# Supplementary material for: A systematic review of impact of person-centred interventions for serious physical illness in terms of outcomes and costs
Source: BMJ Open. 2022 Jul 13;12(7):e054386. doi: 10.1136/bmjopen-2021-054386 (PMC9280891; doi:10.1136/bmjopen-2021-054386)
Supplement: Supplementary data [file bmjopen-2021-054386supp001.pdf]

Supplementary file 1: Searches conducted in electronic databases

Embase Classic+Embase <1947 to 2022 April 08>

1 (patient centred care or patient focused care or person centred care or person focused care or family centred care or family focused care or individualised care or individualized care or holistic care).mp. [mp=title, abstract, heading word, drug trade name, original title, device manufacturer, drug manufacturer, device trade name, keyword heading word, floating subheading word, candidate term word] 15839
2 limit 1 to yr="2020 -Current" 3865
3 (serious illnesse$ or serious illness or chronic disease or chronic illnesse$ or chronic illness or long term conditions or life limiting illnesse$ or life threatening illnesse$).mp. [mp=title, abstract, heading word, drug trade name, original title, device manufacturer, drug manufacturer, device trade name, keyword heading word, floating subheading word, candidate term word] 267608
4 limit 3 to yr="2020 -Current" 28132
5 2 and 4= **208**

https://access.ovid.com/custom/redirector/wayfless.html?idp=https://kclidpdev.kcl.ac.uk/idp/shibboleth&url=http://ovidsp.ovid.com/ovidweb.cgi?T=JS&NEWS=N&PAGE=main&SHAREDSEARCHID=28YQbqmYyLtIarRwfTMEDazD3SF1RTirGR7GVD1pZwebUiJijdYVVfOGwyYvC4Q9F

Ovid MEDLINE(R) ALL <1946 to April 08, 2022>

1 (patient centred care or patient focused care or person centred care or person focused care or family centred care or family focused care or individualised care or individualized care or holistic care).mp. [mp=title, abstract, original title, name of substance word, subject heading word, floating sub-heading word, keyword heading word, organism supplementary concept word, protocol supplementary concept word, rare disease supplementary concept word, unique identifier, synonyms] 9843
2 limit 1 to yr="2022 -Current" 463
3 (serious illnesse$ or serious illness or chronic disease or chronic illnesse$ or chronic illness or long term conditions or life limiting illnesse$ or life threatening illnesse$).mp. [mp=title, abstract, original title, name of substance word, subject heading word, floating sub-heading word, keyword heading word, organism supplementary concept word, protocol supplementary concept word, rare disease supplementary concept word, unique identifier, synonyms] 323614
4 limit 3 to yr="2020 -Current" 22416
5 2 and 4= **21**

https://access.ovid.com/custom/redirector/wayfless.html?idp=https://kclidpdev.kcl.ac.uk/idp/shibboleth&url=http://ovidsp.ovid.com/ovidweb.cgi?T=JS&NEWS=N&PAGE=main&SHAREDSEARCHID=3k81aYpUq8d9oWXfG0tpLyxhYNYL4I8uKFDxDdUBPLBxl0VFpcFdxjgQG9nGHLTIL

AMED (Allied and Complementary Medicine) <1985 to April 2022>

1	patient centred care.mp.	76
2	limit 1 to yr="2020 -Current"	7
3	patient focused care.mp.	15
4	limit 3 to yr="2020 -Current"	0
5	person centred care.mp.	37
6	limit 5 to yr="2020 -Current"	4
7	person focused care.mp.	1
8	limit 7 to yr="2020 -Current"	0
9	family centred care.mp.	35
10	limit 9 to yr="2020 -Current"	3
11	family focused care.mp.	2
12	limit 11 to yr="2020 -Current"	0
13	family focused care.mp.	2
14	individualised care.mp.	10
15	limit 14 to yr="2020 -Current"	0
16	individualized care.mp.	39
17	limit 16 to yr="2020 -Current"	6
18	holistic care.mp.	198
19	limit 18 to yr="2020 -Current"	10
20	2 or 4 or 6 or 8 or 10 or 13 or 15 or 17 or 19	30
21	serious illnesse$.mp.	18
22	limit 21 to yr="2020 -Current"	1
23	chronic illnesse$.mp.	184
24	limit 23 to yr="2020 -Current"	8
25	chronic disease$.mp.	8168
26	limit 25 to yr="2020 -Current"	449
27	life limiting condition$.mp.	67
28	limit 27 to yr="2020 -Current"	2
29	long term condition$.mp.	103
30	limit 29 to yr="2020 -Current"	4
31	22 or 24 or 26 or 28 or 30	461
32	20 and 31	3

https://access.ovid.com/custom/redirector/wayfless.html?idp=https://kclidpdev.kcl.ac.uk/idp/shibboleth&url=http://ovidsp.ovid.com/ovidweb.cgi?T=JS&NEWS=N&PAGE=main&SHAREDSEARCHID=5GZpoqjqkW0mUgtn9svLWQBU36eWXerU3Fd7QGdHRVZTQBRzrvXtT9tbtNMrEBzMp

APA PsycInfo <1806 to April Week 1 2022>

1 (patient centred care or patient focused care or person centred care or person focused care or family centred care or family focused care or individualised care or individualized care or holistic care).mp. [mp=title, abstract, heading word, table of contents, key concepts, original title, tests & measures, mesh word] 3321
2 limit 1 to yr="2020 -Current" 699
3 (serious illnesse$ or serious illness or chronic disease or chronic illnesse$ or chronic illness or long term conditions or life limiting illnesse$ or life threatening illnesse$).mp. [mp=title, abstract, heading word, table of contents, key concepts, original title, tests & measures, mesh word] 45885
4 limit 3 to yr="2020 -Current" 2633
5 2 and 4= **32**

https://access.ovid.com/custom/redirector/wayfless.html?idp=https://kclidpdev.kcl.ac.uk/idp/shibboleth&url=http://ovidsp.ovid.com/ovidweb.cgi?T=JS&NEWS=N&PAGE=main&SHAREDSEARCHID=HpRfZ61FWxXJBuPHZcLglO4z1gqDLHbl79dpT2rMPZwi5gtxtwDxWaR3c9OQEHek

Saturday, April 30, 2022 4:12:13 PM

#	Query	Limiters/Expanders	Last Run Via	Results
S22	S13 AND S21	Expanders - Apply equivalent subjects<br>Search modes - Boolean/Phrase	Interface - EBSCOhost Research Databases<br>Search Screen - Advanced Search<br>Database - CINAHL	303
S21	S14 OR S15 OR S16 OR S17 OR S18 OR S19 OR S20	Expanders - Apply equivalent subjects<br>Search modes - Boolean/Phrase	Interface - EBSCOhost Research Databases<br>Search Screen - Advanced Search<br>Database - CINAHL	87,551
S20	"long term conditions"	Expanders - Apply equivalent subjects<br>Search modes - Boolean/Phrase	Interface - EBSCOhost Research Databases<br>Search Screen - Advanced Search<br>Database - CINAHL	1,903
S19	"long term condition$"	Expanders - Apply equivalent subjects<br>Search modes - Boolean/Phrase	Interface - EBSCOhost Research Databases<br>Search Screen - Advanced Search<br>Database - CINAHL	467
S18	"serious illness"	Expanders - Apply equivalent subjects<br>Search modes - Boolean/Phrase	Interface - EBSCOhost Research Databases<br>Search Screen - Advanced Search<br>Database - CINAHL	1,987
S17	"serious illnesse$"	Expanders - Apply equivalent subjects<br>Search modes - Boolean/Phrase	Interface - EBSCOhost Research Databases<br>Search Screen - Advanced Search<br>Database - CINAHL	0
S16	"chronic illness"	Expanders - Apply equivalent subjects<br>Search modes - Boolean/Phrase	Interface - EBSCOhost Research Databases<br>Search Screen - Advanced Search<br>Database - CINAHL	29,877
S15	"chronic illnesse$"	Expanders - Apply equivalent subjects	Interface - EBSCOhost Research Databases	2

		Search modes - Boolean/Phrase	Search Screen - Advanced Search<br>Database - CINAHL	
S14	"chronic disease$"	Expanders - Apply equivalent subjects<br>Search modes - Boolean/Phrase	Interface - EBSCOhost Research Databases<br>Search Screen - Advanced Search<br>Database - CINAHL	79,620
S13	S1 OR S2 OR S3 OR S4 OR S5 OR S6 OR S7 OR S8 OR S9 OR S10 OR S11 OR S12	Expanders - Apply equivalent subjects<br>Search modes - Boolean/Phrase	Interface - EBSCOhost Research Databases<br>Search Screen - Advanced Search<br>Database - CINAHL	7,546
S12	"holistic care"	Limiters - Published Date: 20200101-20220431<br>Expanders - Apply equivalent subjects<br>Search modes - Boolean/Phrase	Interface - EBSCOhost Research Databases<br>Search Screen - Advanced Search<br>Database - CINAHL	983
S11	(MH "Holistic Care")	Limiters - Published Date: 20200101-20220431<br>Expanders - Apply equivalent subjects<br>Search modes - Boolean/Phrase	Interface - EBSCOhost Research Databases<br>Search Screen - Advanced Search<br>Database - CINAHL	690
S10	"individualized care"	Limiters - Published Date: 20200101-20220431<br>Expanders - Apply equivalent subjects<br>Search modes - Boolean/Phrase	Interface - EBSCOhost Research Databases<br>Search Screen - Advanced Search<br>Database - CINAHL	199
S9	"individualised care"	Limiters - Published Date: 20200101-20220431<br>Expanders - Apply equivalent subjects<br>Search modes - Boolean/Phrase	Interface - EBSCOhost Research Databases<br>Search Screen - Advanced Search<br>Database - CINAHL	67
S8	"family focused care"	Limiters - Published Date: 20200101-	Interface - EBSCOhost Research Databases	9

		20220431 Expanders - Apply equivalent subjects Search modes - Boolean/Phrase	Search Screen - Advanced Search Database - CINAHL	
S7	"family centered care"	Limiters - Published Date: 20200101-20220431 Expanders - Apply equivalent subjects Search modes - Boolean/Phrase	Interface - EBSCOhost Research Databases Search Screen - Advanced Search Database - CINAHL	2,200
S6	(MH "Family Centered Care")	Limiters - Published Date: 20200101-20220431 Expanders - Apply equivalent subjects Search modes - Boolean/Phrase	Interface - EBSCOhost Research Databases Search Screen - Advanced Search Database - CINAHL	898
S5	(MH "Patient Centered Care")	Limiters - Published Date: 20200101-20220431 Expanders - Apply equivalent subjects Search modes - Boolean/Phrase	Interface - EBSCOhost Research Databases Search Screen - Advanced Search Database - CINAHL	4,081
S4	"patient focused care"	Limiters - Published Date: 20200101-20220431 Expanders - Apply equivalent subjects Search modes - Boolean/Phrase	Interface - EBSCOhost Research Databases Search Screen - Advanced Search Database - CINAHL	1,756
S3	"patient centred care"	Limiters - Published Date: 20200101-20220431 Expanders - Apply equivalent subjects Search modes - Boolean/Phrase	Interface - EBSCOhost Research Databases Search Screen - Advanced Search Database - CINAHL	2,006
S2	"person focused care"	Limiters - Published Date: 20200101-20220431 Expanders - Apply	Interface - EBSCOhost Research Databases Search Screen - Advanced	4

Supplemental material
BMJ Publishing Group Limited (BMJ) disclaims all liability and responsibility arising from any reliance placed on this supplemental material which has been supplied by the author(s)
BMJ Open

		equivalent subjects<br>Search modes - Boolean/Phrase	Search<br>Database - CINAHL	
S1	"person centred care"	Limiters - Published Date: 20200101-20220431<br>Expanders - Apply equivalent subjects<br>Search modes - Boolean/Phrase	Interface - EBSCOhost Research Databases<br>Search Screen - Advanced Search<br>Database - CINAHL	453
