# Supplementary material for: A systematic review of impact of person-centred interventions for serious physical illness in terms of outcomes and costs
Source: BMJ Open. 2022 Jul 13;12(7):e054386. doi: 10.1136/bmjopen-2021-054386 (PMC9280891; doi:10.1136/bmjopen-2021-054386)
Supplement: Supplementary data [file bmjopen-2021-054386supp002.pdf]

**Supplementary file 2: Characteristics of studies included in the review N=55**

| Study Number | Author & Year/<br><br>Country | Aim<br>Design<br>Theoretical model | Sample | Intervention(s) | Outcomes/measures and follow-up period | Results |
|---|---|---|---|---|---|---|
| 1 | Fortin et al 2021 (1)<br><br>Canada | To measure the effectiveness of a 4-month interdisciplinary multifaceted intervention based on a change in care delivery for patients with multimorbidity in primary care practices.<br><br>RCT<br><br>Chronic Care Model and Patient-Centred Clinical Method | N=284 patients with multimorbidity (n=144 mean age (SD) 60.8 (10.6) intervention and n=140 mean age (SD) 61.1 (10.3) control) | Consisted of: (1) training the professionals on patient-centered care for persons with multimorbidity, self-management support, interprofessional collaboration, and motivational approach. (2) suggested clinical pathways for patients, with individual visits to health care professionals were developed for each patient. Pathways started with a contact nurse who performed a clinical assessment, elicited patients' goals, and created an individualized care plan. Patients were then referred to the most appropriate professional(s) matching patient goals, including referrals to the nurses themselves. A final visit was with the contact nurse to summarize and plan for sustainability. and (3) | Primary outcomes:<br>1. Health education impact: Health Education Impact Questionnaire<br><br>2. Self-Efficacy: Self-Efficacy for Managing Chronic Diseases<br><br>Secondary outcomes:<br><br>3. Health status: Veterans RAND<br><br>4. Quality of Life: EuroQoL<br><br>5.Psychosocial distress: Kessler Psychological Distress Scale Questionnaire<br><br>6.Health behaviours: Behavioural Risk Surveillance System Outcomes collected at baseline and month 4 | 1-5 No statistically significant differences<br><br>6. Significant differences on physical activity and healthy eating, but not significant on high-risk alcohol |

| Study Number | Author & Year/<br><br>Country | Aim<br>Design<br>Theoretical model | Sample | Intervention(s) | Outcomes/measures and follow-up period | Results |
|---|---|---|---|---|---|---|
| | | | | creating a community of practice within each family medicine group (FMG).<br><br>Patients assigned to the control group were placed on a waiting list to receive the intervention after 4 months.<br>In the meantime, they had access to their usual care including elective appointments with their family doctors or urgent appointments with their heath care professionals for acute reasons (trauma, infection, etc). | | consumption and smoking habit. |
| 2 | de Batlle, 2020 (2)<br><br>Spain | To assess the effectiveness and cost-effectiveness of the implementation of a mobile health (mHealth)-enabled integrated care model for complex chronic patients.<br><br>a prospective, pragmatic, two-arm, | Elderly patients with COPD, heart failure and caregivers<br><br>N=52 integrated care model, mean age (SD): 82(7)<br><br>n=35 usual care, mean age (SD): 82(8). | The combined benefits of the CONNECARE (Personalised Connected Care for Complex Chronic Patients) organizational integrated care model and the eHealth platform supporting it, consisting of a (i) self-management app, with status and performance reports, a | 1. Quality of life (changes in health status): 12-Item Short-Form Survey (SF-12), Barthel index for Activities of Daily Living and, Hospital Anxiety and Depression scale<br><br>2. Use of health care resources and estimated | 1. No significant differences between the two groups (mean change (SD) 5.0 (5.2) p= .10<br><br>2.Unplanned visits were significantly |

| Study Number | Author & Year/ Country | Aim Design Theoretical model | Sample | Intervention(s) | Outcomes/measures and follow-up period | Results |
|---|---|---|---|---|---|---|
| | | parallel implementation trial | | virtual coach with customizable automated feedback, and full communication with the care team; (ii) a Fitbit Flex 2 digital activity tracker and any additional sensor deemed necessary by the care team including a digital pulse-oximeter, digital scale, and digital blood pressure monitor, that were fully integrated into the self-management app; (iii) a patient profile in the SACM (Smart Adaptive Case Management) web-based platform, accessible to all members of the care team (family physicians, hospital specialists, and social workers), that was used for coordination and communication among professionals in the different settings, and to contact the patient when needed; and (iv) assignment of a case manager in charge of supervising the whole process and | associated costs based on Catalan Health Department official data: Unplanned visits and admission<br><br>3. cost-effectiveness, based on the improvement in QoL relative to costs, assessed by means of the incremental cost-effectiveness ratio (ICER);<br><br>Data collected at baseline and a 6-month follow up, | lower in the intervention group (2.3 (3.1) vs 1.0 (1.1) P=0.004).<br><br>3. The integrated care program generated savings from US $584 to $1434 per patient, depending on the scenarios. The integrated care program was cost-effective according to the ICER, performing better in terms of QoL while reducing overall expenses |

| **Study Number** | **Author & Year/ Country** | **Aim Design Theoretical model** | **Sample** | **Intervention(s)** | **Outcomes/measures and follow-up period** | **Results** |
|---|---|---|---|---|---|---|
| | | | | serving as the main patient contact point.<br><br>Control group received usual care (details not provided). | | |
| 3 | Mielenz et al 2020 (3)<br><br>USA | To evaluate the Self-management Resource Center Small Group Programs (SMRCSGP), plus wellness coaching, as a booster intervention in older adults with chronic diseases.<br><br>To evaluate the role of personal health records (PHR) prototype as the linkage between the clinic and community.<br><br>RCT<br><br>Self-efficacy theory | Elderly people >55 years old. N=125 Intervention n=62, mean age (SD) 72 (0.94)<br><br>Control n=63, mean age (SD) 73.1 (0.95) | The intervention: The wellness self-coaching program asked participants to create a “Wellness Vision,” wherein the participants set monthly and weekly behavioural goals that were agreed upon by participant and coach. Class lesson titles were as follows: taming frenzy, self-compassion, focus, mindfulness, strengths (two-part), motivation, legacy, creativity (two-part), body intelligence (two-part), relationships (two-part), positivity (two-part), meaning (two-part), curiosity (two-part), standard setter (two-part), self-leadership, and your plan to thrive. | Primary outcomes<br>1. Physical activity: The Community Health Activities Model Program for Seniors (CHAMPS) was used to collect information on physical activity.<br>-Frequency per week of all exercise-related activities<br>-Hours per week of all exercise-related activities<br><br>2.Behavioral Risk Factor Surveillance System physical activity measures<br>-Met aerobic physical activity guidelines,<br>-Met aerobic and muscle strengthening guidelines, | Across the 6 months of our study the intervention and control groups did not vary significantly on any primary physical activity outcomes of interest (CHAMPS and BRFSS measures) in models.<br><br>The intervention and control groups did vary significantly (p = .03) over time on one secondary outcome: the PROMIS physical function variable. Although both groups reported |

| Study Number | Author & Year/ Country | Aim Design Theoretical model | Sample | Intervention(s) | Outcomes/measures and follow-up period | Results |
|---|---|---|---|---|---|---|
| | | | | Control: Both groups received usual care consisting of self-management Resource Center Small Group Programs (SMRCSGP) (including programs on general chronic disease and specific conditions: arthritis, diabetes, HIV, chronic pain, and cancer) are structured wellness interventions that encourage self-management in older adults living with chronic conditions and are implemented by lay leaders | Secondary outcomes:<br>3. Patient-Reported Outcomes Measurement Information System (PROMIS) v1.0 short form (SF) measures:<br><br>Depressin: Emotional Distress-Depression—SF<br><br>Fatigue: Fatigue—SF 4a,<br><br>Pain behaviour: Pain Behavior—SF 7a,<br><br>Pain intensity: Pain Intensity—SF 3a,<br><br>Pain interference: Pain Interference—SF 4a,<br><br>Physical function: Physical Function—SF20a),<br><br>Sleep: Sleep Disturbance—SF 4a. | improvements on this measure over time (higher scores indicating that participants can do more and feel better), overall improvement was greater for the wellness coaching intervention group (2.6) than for the control (0.6). |

| Study Number | Author & Year/<br><br>Country | Aim<br>Design<br>Theoretical model | Sample | Intervention(s) | Outcomes/measures and follow-up period | Results |
|---|---|---|---|---|---|---|
| | | | | | 4. Medical care questions:<br>- Times visiting a physician<br>- Times visiting a hospital emergency department<br>- Times hospitalized for one night or longer<br>- Total nights spent in the hospital<br><br>-Self-efficacy for exercise was assessed on the Resnick Self- Efficacy for Exercise (SEE)<br>-Falls in the past month<br><br>CHAMPS data were collected at baseline, 3 months, and 6 months. | |
| 4 | Yu et al 2020 (4)<br><br>Canada | To assess the impact of 'MyDiabetesPlan' on decisional conflict, diabetes distress, health-related | N=102 patients<br>n=29 clinicians<br><br>N=111 patients<br>n=24 clinicians | A web-based PtDA in which patients populate their cardiometabolic and psychosocial profiles and general care | Primary outcome:<br>1. Decisional conflict: the Decisional Conflict Scale (DCS),<br><br>Secondary outcomes: | 1. No significant differences between the two groups; mean 0.5; p=0.08 |

| Study Number | Author & Year/ Country | Aim Design Theoretical model | Sample | Intervention(s) | Outcomes/measures and follow-up period | Results |
|---|---|---|---|---|---|---|
| | | quality of life, and patient assessment of chronic illness care at the individual patient level.<br><br>Cluster RCT | | priorities: MyDiabetesPlan then generates individualized diabetes-specific goals and strategies based on these inputs that the patients then select, resulting in an action plan.<br>Clinicians at intervention sites underwent a one-on-one 60-min tutorial in their clinic room by the research coordinator, with access to a one-page how-to guide and 2-min video. During subsequent clinical encounters, a member of the interprofessional team (nurse or dietitian) logged into MyDiabetesPlan and completed it with the patient; the physician subsequently reviewed the resultant action plan with the patient. At 6 months, patients at intervention sites were provided with a patient-directed how-to guide and video and | 2. Diabetes distress: Diabetes Distress Scale (DSS)<br><br>3., Health-related quality of life: SF-12<br><br>4. Chronic illness care: PACIC (Patient Assessment of Chronic Illness Care) Scale<br><br>5. intention to engage in IPSDM (Interprofessional Shared Decision-Making): CPD (Continuing Professional Development.) Reaction Questionnaire<br><br>Outcomes were assessed at the individual participant level, at baseline, and at 6 months and 12 months (after an appointment) through a web-based survey or by mail. | 2. mean change 0.2 p=0.12<br><br>3. mean change 1.2 p=0.57<br><br>4. Mean change 0.15 p<0.001<br><br>5. No significant differences between two groups. |

| Study Number | Author & Year/ Country | Aim Design Theoretical model | Sample | Intervention(s) | Outcomes/measures and follow-up period | Results |
|---|---|---|---|---|---|---|
| | | | | directed to update MyDiabetesPlan according to their progress before the appointment.<br><br>Clinicians in the control sites received paper copies of the executive summary of the Diabetes Canada clinical practice guidelines, and a postcard outlining web-based clinical information resources. After 6 months, patients in the control sites received a Diabetes Canada patient education pamphlet regarding diabetes self-management and a postcard outlining web-based additional patient resources. | | |
| 5 | Bergsten et al 2019 (5)<br><br>Sweden | To evaluate the effect of a nurse-led clinic with frequent visits, treat-to-target and person-centred care of patients with rheumatoid arthritis and moderate-to-high | N=70 patients with moderate to severe symptoms.<br><br>n=36 intervention group, mean age 60.3 (SD 15.9), | 4 nurses attended 2 days' training on principles, philosophy, and delivery of person-centred care.<br>An individual health plan agreed by patient and nurse, including aims for disease activity and | (1) Primary outcome was the difference in the DAS28 change: DAS28 is an index based on the number of tender and swollen joints, patients' global health assessment and the | In the PP analyses, the primary outcome (i.e., the difference in delta-DAS28 between the IG and CG) was not statistically |

| Study Number | Author & Year/ Country | Aim Design Theoretical model | Sample | Intervention(s) | Outcomes/measures and follow-up period | Results |
|---|---|---|---|---|---|---|
| | | disease activity compared with patients receiving regular care.<br><br>RCT<br><br>Gothenburg PCC | n=34 control group, mean age 62.4 (SD 12.2). | participation, tools to achieve these goals.<br><br>Patients in the control group were offered a telephone appointment with their regular physician, in order to discuss their disease activity and whether a physical appointment, and potentially a change in therapy, should be made. All patients were then followed by their treating physician according to regular care, with follow-up visits decided either at this telephone appointment or according to previous plans. In regular care, the patients usually visited the clinic every 6–12 months. As part of regular care, patients also had the possibility of making appointments with the physician in the event of flares. | erythrocyte sedimentation rate.<br><br>Secondary outcomes:<br><br>(2) the proportions with minimal clinical important improvement in DAS28 (>0.6)<br><br>(3) the proportions achieving low disease activity (DAS28 <3.2);<br><br>(4) the proportions achieving a EULAR moderate or good response<br><br>(5) the Health Assessment Questionnaire score, measuring daily function<br><br>(6) the RA impact of disease (RAID) score, measuring the impact of RA from the patient's perspective; | significant (0.43; 95% CI −0.27, 1.13)<br><br>Nonsignificant difference in ITT primary PCC in DAS 26 (mean (95% CI)): 1.39 (0.97 to 1.82) v control 1.04 (0.54 to 1.53).<br><br>In PP PCC 1.50 (1.00 to 2.00) v control 1.07 (0.56 to 1.57). Trial inclusion terminated because more patients in the interventions dropped out |

| Study Number | Author & Year/<br><br>Country | Aim<br>Design<br>Theoretical model | Sample | Intervention(s) | Outcomes/measures and follow-up period | Results |
|---|---|---|---|---|---|---|
| | | | | | (7) Patient Acceptable Symptom State (PASS) score (8) the Beliefs about Medicines Questionnaire (BMQ) responses, measuring patients' attitude to medication split in two domains (BMQ-necessity, BMQ-concerns)<br><br>(9) the EuroQol-5D (EQ-5D) score). | |
| 6 | Berntsen et al (2019)(6)<br><br>Norway | To determine if the Patient-Centred Team Intervention (PACT) causes reduced use of high-level emergency care and increased use of low-level planned care with unchanged mortality risk for the multi-morbid elderly<br><br>Parallel arm study | N=1218 patients >60 years, with multi-morbidity, complex long-term needs and high short-term risk for emergency hospital admission n=439 intervention group, referred to the PACT team. Mean age 80.02 (SD8.72) | Intervention: Patient is assigned to a mini-team of nurse co-ordinator, physician, physiotherapist, occupational therapist and pharmacist. They work with the patient to explore goals using a person-centred approach including a comprehensive geriatric assessment methodology. The team address immediate clinical needs and co-ordinate Average intervention time 30 days. | 1. Number of emergency admissions<br><br>2. Sum of emergency inpatient bed days<br><br>3. Count of emergency re-admissions within 30 days of discharge<br><br>4. Count of planned out-patient visits<br><br>5. Count of emergency outpatient visits | 1. Adjusted RR 0.90 (95%CI: 0.82-0.99)<br><br>2. Adjusted RR 0.68 (95%CI 0.52-0.79)<br><br>3. Adjusted RR 0.72 (95%CI 0.41-1.24)<br><br>4. Adjusted RR 2.27 (95%CI 2.02-2.55)<br><br>5. Adjusted RR 0.90 (95%CI 0.68-1.2) |

| Study Number | Author & Year/<br><br>Country | Aim<br>Design<br>Theoretical model | Sample | Intervention(s) | Outcomes/measures and follow-up period | Results |
|---|---|---|---|---|---|---|
| | | | n=779 control group, mean age 78.8 years (SD 8.68). Patients had an emergency admission but not received PACT intervention. A matched local and distant control was sought for each intervention participant. | Control group: usual care defined as evidence-based care for the cause of the emergency admission to hospital, referral for other diagnoses to GP or specialist care and standard electronic communication. | 6. Mortality risk at 3 and 6 months follow-up<br>Follow up began at first referral to PACT (IG) or time of emergency admission (CG) and ended after 6 months or death. | 6. Adjusted RR 0.39 (95%CI 0.22-0.7) at 3 months and 0.57 (95%CI 0.34-0.94) at 6 months. |
| 7 | Berendonk (2019) (7)<br><br>Germany. | To test the feasibility of a nursing intervention (DEMIAN) in routine care and its effects on care providers' job satisfaction, motivation and work strain.<br><br>Pragmatic two-group cluster RCT | N=20 German long-term care facilities<br>n= 84 care providers (mean age 41.8, SD 10.2) and 42 residents with dementia in intervention group<br>n= 96 care providers (mean age 38.5, SD 11.9) and 42 residents with | Intervention: Registered nurses completed two days of training within a two week period on the DEMIAN intervention. Its objectives are to gather information on meaningful situations for each individual and to use this knowledge to plan and provide care. There was a 6 week implementation phase after training to carry out mini-interventions. Nurses encourages all team members, relatives and | 1. Screening instrument for job strain in human service work (BHD)<br><br>2. Modified Task and Job Analysis Tool- residential LTC version (TAA-A)<br>Baseline assessment and at post intervention follow up | 1. Greater job satisfaction in IG than CG post intervention (p=0.053)<br><br>2. Most TAA-A outcomes did not differ significantly between IG and CG after intervention. Time pressure did decrease in IG compared to CG (p=0.026) |

| Study Number | Author & Year/ Country | Aim Design Theoretical model | Sample | Intervention(s) | Outcomes/measures and follow-up period | Results |
|---|---|---|---|---|---|---|
| | | | dementia in control group | volunteers to be involved in the interventions.<br><br>Control: usual care (details not provided). | | |
| 8 | Bökberg et al (2019) (8)<br><br>Sweden | To evaluate whether an educational intervention had any effect in the staff's perception of providing person-centred palliative care for older persons in nursing homes.<br><br>Pre- and post-test experimental design. | N=365 nursing home staff (nurses, assistant nurses, physiotherapists, occupational therapists, social workers and unit managers) recruited from 20 urban and rural, small (<25 residents) and large (>100 residents) nursing homes in two Swedish counties<br>n=167 intervention group, median age 47<br>n=198 control group, median age 49 years | Intervention: A knowledge-based palliative care intervention consisting of five 2h educational seminars for nursing home staff based on Swedish national documents on the key principles of palliative care intending to improve quality of life for individuals and their families. Participants were provided with a study booklet. The intervention was implemented over 6 months.<br><br>Control: usual training. None of the participating homes had had workplace education or training in palliative care before the intervention. | 1. Person-centred Care Assessment Tool (P-CAT)<br><br>2. Person-Centred Climate Questionnaire (PCQ-S)<br><br>Data collected at baseline and post-intervention | 1. No significant change in total P-CAT score pre- and post intervention in IG (p=0.715)or CG (p=0.601)<br>No statistically significant changes in pre and psot intervention scores on any subscale for either group.<br><br>2. No significant change in total PCQ-S scores pre and post intervention in IG (p=0.685) or CG (p+0.451)<br>No statistically significant changes in pre and post intervention scores on any subscale for either group. |

| **Study Number** | **Author & Year/ Country** | **Aim Design Theoretical model** | **Sample** | **Intervention(s)** | **Outcomes/measures and follow-up period** | **Results** |
|---|---|---|---|---|---|---|
| 9 | Britt et al (2019) (9)<br><br>USA | To assess the effect of the LifeCourse (LC) programme on healthcare utilisations<br><br>Quasi-experimental trial | N=903 patients estimated to be within 3 years of end of life with 1+ serious illness n=450 intervention, mean age 78.1 (SD 12.0)<br>n= 453 control, mean age 74.3 (SD 12.5) recruited from area hospitals or care centres | Intervention: Hour long, monthly home visits for patients and caregivers if the patient desired. Structured visits included setting intentions, discussing goals and guided assessments with the aim of enabling patients to articulate what mattered to them and their goals for living. Visit delivered by a community health worker who had undertaken a 2 week training programme.<br><br>Control: Usual care – standard medical care including palliative, care management, home care, and/or hospice care services | 1. Patient healthcare utilisation<br><br>2. Patient Quality of Life: FACIT-Pal<br><br>3. Patient care experience | 1. Higher proportion of IG completed an advanced directive than CG (173 vs 66, p<0.001). No significant difference in hospice use between dying patients in IG and CG. IG patients spent longer in hospice than CG (88 days vs 44 days, p<0.18). No significant differences between groups in days spent in the ED, hospital or ICU.<br><br>2. No difference between groups (p=0.649)<br><br>3. IG reported greater improvement in the communication domain than CG (p=0.16). No other statistically |

| Study Number | Author & Year/ Country | Aim Design Theoretical model | Sample | Intervention(s) | Outcomes/measures and follow-up period | Results |
|---|---|---|---|---|---|---|
| | | | | | 4. Caregiver experience<br><br>5. Caregiver quality of life: PROMIS-29 Measures collected at baseline then every 3 months until death or 30 months | significant treatment by time effects.<br><br>4. No effect<br><br>5. CG carers had greater increase in anxiety and depression domains compared to IG (B=-0.98, p=0.038 and B=-0.098, p=0.014). No other statistically significant treatment by time effects. |

| Study Number | Author & Year/ Country | Aim Design Theoretical model | Sample | Intervention(s) | Outcomes/measures and follow-up period | Results |
|---|---|---|---|---|---|---|
| 10a | Hedman, et al 2019 (10)<br><br>Sweden | To compare five-year outcomes and changes over time of a client-centred activities of daily living (ADL) intervention versus usual ADL interventions for people with stroke and their significant others.<br><br>RCT<br><br>Gothenburg PCC | People with stroke and significant others.<br><br>N=145 people with stroke (intervention group: n = 71): mean age (SD): 71(9)<br><br>control group: n = 74): mean age (SD): 68 (9)<br><br>N=75 significant others (intervention group: n = 36): mean age (SD) 65 (17)<br><br>(control group: n = 39): mean age (SD) 69 (10). | Intervention: Participants with stroke received an occupational therapist delivered client centred ADL intervention aiming to increase agency in daily activities and participation in everyday life guided by their expressed desires. Occupational therapists had participated in a 5 day workshop on client centredness.<br><br>Control: Rehabilitation in a unit providing usual ADL interventions | Primary outcome<br>1. Perceived participation: Stroke Impact scale<br><br>Secondary outcome:<br>2. Perceived participation: Occupational gaps questionnaire<br><br>3. Frequency of participation in social and complex everyday activities: Frenchay Activities Index<br><br>4. Self-reported use of assistance (yes/no) in six personal and four instrumental ADL: The Katz Extended Scale<br><br>5. Perceived self-efficacy in performing everyday activities: a Self-Efficacy Scale<br><br>6. Overall satisfaction with life: Life Satisfaction Scale | For patients:<br>1. Mean difference –6.5 (–13.3 to 0.3), p= 0.062<br><br>2. Mean difference 0.7 (–0.6 to 2.0), p=0.293<br><br>3. Mean difference –0.2 (–3.2 to 2.7), p=0.885<br><br>4. Odds ratio 0.4 (0.2 to 0.8) p=0.012<br><br>5. Mean difference 2.7 (–8.2 to 13.6), p=0.621<br><br>6. Odds ratio 0.6 (0.2 to 1.3), p= 0.219 |

| Study Number | Author & Year/ Country | Aim Design Theoretical model | Sample | Intervention(s) | Outcomes/measures and follow-up period | Results |
|---|---|---|---|---|---|---|
| | | | | | 7. Globally assess perceived quality of life: Reintegration into normal living index | 7. Mean difference –0.6 (–3.0 to 1.8), p=0.617 |
| | | | | | 8. Mood: Hospital anxiety and depression scale | 8. Anxiety: mean difference –0.3 (–1.6 to 1.0) p=0.611<br>Depression: mean difference –0.4 (–1.6 to 0.7), p=0.474 |
| | | | | | 9. Fatigue severity: fatigue severity scale | 9: Mean difference –2.6 (–6.9 to 1.8), p=0.245 |
| | | | | | For significant others: | : |
| | | | | | 10. Burden of care: caregiver burden scale | 10: Mean difference −4.7 (−12.0 to 2.5), p=0.196 |
| | | | | | 11. Informal care was assessed by the use of the question 'To what extent do you assist your significant other?' | 11: Mean difference −6.0 (−20.1 to 8.1), p=0.402 |

| Study Number | Author & Year/<br><br>Country | Aim<br>Design<br>Theoretical model | Sample | Intervention(s) | Outcomes/measures and follow-up period | Results |
|---|---|---|---|---|---|---|
| | | | | | 12. Mood: HADS as above<br><br>13. The overall satisfaction with life: The 'My life as a whole' item in LiSat-11 was used to assess<br><br>14. Restrictions (gaps) in participation in everyday occupations: The 30-item version of the Occupational Gaps Questionnaire. | 12. Significant differences between two groups −1.7 (–3.0 to −0.5); p=0.005<br><br>13: Odds 1.1 (0.4 to 2.8) p=0.922<br><br>14: Mean difference −0.6 (−2.0 to 0.7), p=0.329 |
| 10 b, c, d | Bertilsson et al (2016) (11)<br><br>Guidetti et al (2015) (12)<br><br>Bertilsson et al (2014) (13)<br><br>(Four papers one study) | a) To determine if a client centred activity of daily living (ADL) group after stroke has an effect on caregiver burden, provision of informal care, perceived participation in everyday occupations and life satisfaction. | N= 183 caregivers of people with stroke attending inpatient or home rehabilitation n=88 intervention group, mean age 60 (SD 14.6) n=95 control group, mean age 64 (SD 13.1) | As above | 1. Caregiver burden: Caregiver Burden Scale.<br><br>2. Informal care: percentage reporting providing assistance with personal ADLs, instrumental ADLs or other activities. | 1. No difference between intervention and control groups at 12 months (42.7 vs 41.8, p=0.75).<br><br>2. No difference between intervention and control groups in for personal ADLs (42 vs 50%, p=0.51), Instrumental ADLs (67 vs 68%, p=0.88) |

| Study Number | Author & Year/<br><br>Country | Aim<br>Design<br>Theoretical model | Sample | Intervention(s) | Outcomes/measures and follow-up period | Results |
|---|---|---|---|---|---|---|
| | Sweden | b) To compare changes regarding perceived participation, independence in activities of daily living (ADL) and life satisfaction between 3, 6 and 12 months after inclusion in a study of a client-centred ADL intervention and usual ADL intervention after stroke.<br><br>c) To study a client-centred activities of daily living (ADL) intervention (CADL) compared with the usual ADL intervention (UADL) in people with stroke regarding: independence in ADL, perceived participation, life satisfaction, use of home-help service, and satisfaction with training.<br><br>Cluster RCT | N=280 people with stroke<br><br>Intervention n=129, mean age (SD) 74 (10)<br><br>Control n=151, mean age (SD) 71 (10.8) | | 3. Participation in everyday occupations: Occupational Gaps Questionnaire (OGQ).<br><br>4. Life satisfaction: Life satisfaction scale (LiSat-11)<br>Outcomes measured at 3 and 12 months | or other support (65 vs 76%, p=0.09) at 12 months.<br><br>3. No difference between intervention and control groups (3.5 vs 4.0, p=0.52) at 12 months.<br><br>4. No difference between intervention and control groups (47 vs 47%, p=0.87) at 12 months<br>No differences between intervention and control groups in changes in outcomes between 3 and 12 months. Except the intervention group had lower General strain at 12 months than 3 months (OR 1.74, p=0.014). |

| Study Number | Author & Year/ Country | Aim Design Theoretical model | Sample | Intervention(s) | Outcomes/measures and follow-up period | Results |
|---|---|---|---|---|---|---|
| | | | | | 5. Independence on ADL: Katz Extended scale (KE)<br><br>6. Perceived participation: Stroke Impact Scale (SIS)<br><br>7. Participation in everyday occupations: Occupational Gaps Questionnaire (OGQ).<br><br>8. Life satisfaction: The Life Satisfaction Scale<br><br>9. Home-help service and satisfaction with training: Self-reported (yes/no) by people with stroke.<br><br>Measures at three, six and twelve months. | 5. Intervention n=38; 29.4% vs control n=52; 34.4% p=0.83<br><br>6. No significant different between groups in all 9 items.<br><br>7. Mean OGQ 9.1 intervention, 107 control; p=0.10<br><br>8. N=47 (36.4%) intervention vs n=56 (37.1%) control; p=0.79<br><br>9. Home help service n=57 (44.2%) intervention vs n=60 (39.7%) control; p=0.54 Satisfaction with training n=94 (72.9%) vs n=105 (69.5%); p=0.33 |

| Study Number | Author & Year/<br><br>Country | Aim<br>Design<br>Theoretical model | Sample | Intervention(s) | Outcomes/measures and follow-up period | Results |
|---|---|---|---|---|---|---|
| 11 | Ohlen et al (2019) (14)<br><br>Sweden | To evaluate whether an intervention with a person-centred approach to information and communication for patients diagnosed with colorectal cancer undergoing surgery can improve the patients' preparedness for surgery, discharge and recovery during six months following diagnosis and initial treatment<br><br>Quasi-experimental longitudinal study. | People undergoing elective surgery for cancer in the colon or rectum n=238 intervention and n=250 control. | Intervention has two components:<br>1) Written interactive patient education materials tool pertaining to phases of care process (examination, diagnosis, surgery, and recovery).<br>2) Person-centred communication in dialogue format using patient education materials. This was the tool used to communicate between the patient and health professionals.<br><br>Control group: Patients received several written patients education materials related to specific parts or procedures related to surgery and recovery. Communication occurred according to standard care. | 1. The Longitudinal Preparedness for Colorectal Cancer Surgery Questionnaire (PCSQ) in Swedish measures preparedness for surgery and recovery | 1. Relative to the control group, patients in the intervention group reported less decline in the domain "searching for and making use of information" (slopes for control and intervention groups were -18.8 and -14.8, respectively, p = 0.01). Relative to the intervention group, the control group participants reported lower scores for the domain "making sense of the recovery process" at time point 1 pre-surgery (intercepts were 80.9 and 84.4 in the control and intervention groups, p = 0.04) but no difference was |

| Study Number | Author & Year/ Country | Aim Design Theoretical model | Sample | Intervention(s) | Outcomes/measures and follow-up period | Results |
|---|---|---|---|---|---|---|
| | | | | | Length of stay | detected in the slope of the trajectory. There were no statistically significant differences in intercepts or slopes between the two groups for "understanding and involvement in the care process" and "support and access to medical care.<br><br>The length of stay patients who were hospitalized in relation to surgery was 8.8 days (median = 8.0) for the control group compared with 8.0 days (median = 7.0) in the intervention group (N = 488, p = 0.033, based on the logarithm of length of stay). |

| Study Number | Author & Year/ Country | Aim Design Theoretical model | Sample | Intervention(s) | Outcomes/measures and follow-up period | Results |
|---|---|---|---|---|---|---|
| | | | | | 2. EORTC QLQ-C30 version 3.0 (30 items) is a widely used measure of HRQOL for patients diagnosed with cancer and the Swedish version was used | 2. Patients also reported a decline in their role function; however, there was a statistically significant difference in the slopes between the two groups (-17.5 versus -7.9 in the control and intervention groups, p = 0.01).<br><br>General health, emotional function, physical function, and cognitive functions were not significant. |
| | | | | | 3. The National Comprehensive Cancer Network (NCCS) Distress Thermometer (DT; Version 1.2013) was used to detect clinically significant distress in patients | 3. No statistically significant differences detected between the two groups |

| Study Number | Author & Year/<br><br>Country | Aim<br>Design<br>Theoretical model | Sample | Intervention(s) | Outcomes/measures and follow-up period | Results |
|---|---|---|---|---|---|---|
| | | | | | Outcomes collected at six weeks, three and six months. | |
| 12a | Pirhonen et al 2019 (15)<br><br>Sweden | To calculate the cost-effectiveness of a person-centred care intervention compared with usual care in patients with acute coronary syndrome (ACS)<br><br>RCT<br><br>Person-centred care according to the framework by the Gothenburg Centre for Person-Centred Care (GPCC) | N=252<br>n=124 intervention,<br><br>n=128 control<br><br>(1) age < 75 years, and (2) were hospitalised for myocardial infarction or unstable angina pectoris. | The intervention group received person-centred care according to the framework developed by the Gothenburg Centre for Person-Centred Care (GPCC), which comprises routines for establishment of a partnership between patients and healthcare professionals.<br>The intervention was provided by designated healthcare professionals (physicians and registered nurses), at each care level, who had received training through lectures, seminars, and workshops on how to apply the intervention. | 1. Quality of life: EQ-5D-3L questionnaire<br><br>2. Direct Costs and Productivity Losses: in and outpatient care visits, diagnosis related costs, pharmaceutical costs productivity losses (indirect costs) associated with temporary and permanent illness, valued according to the human capital method, that is, time units of lost production were valued at their market value. | The base-case calculations showed that person-centred care was more effective and less costly compared with usual care for patients under 65 years of age, while usual care was more effective and less costly in the older age group.<br><br>The cost-effectiveness of the intervention was found to differ between the two age groups (< 65 years with 117 patients |

| Study Number | Author & Year/ Country | Aim Design Theoretical model | Sample | Intervention(s) | Outcomes/measures and follow-up period | Results |
|---|---|---|---|---|---|---|
| | | | | Professionals listened carefully to the patient's narrative in order to include his or her needs and intrinsic personal resources relevant for the treatment and care process. Based on this narrative, a health plan was co-created, which reflects both the perspective of the patient and the expertise of the healthcare professionals. The health plan also contained agreed goals for the recovery period, which were followed-up and revised by the patient together with the designated healthcare professionals at each care level when necessary.<br><br>Control: Both the intervention group and the control group received usual care according to national guidelines for cardiac care | Data collected at baseline, months 1, 2 and 6 (clinical endpoint) and 1 year after the initial hospital discharge. Information on total healthcare utilisation, sickness absenteeism and drug prescriptions were collected for the 1-year period | and ≥ 65 years with 75 patients). In the younger age group, the intervention induced lower total costs and higher quality of life, while the opposite was true in the older age group.<br>Thus, the person-centred care intervention was the cost effective alternative when compared with usual care for those under the age of 65 years, while usual care was the cost-effective alternative in the older age group. |

| Study Number | Author & Year/<br><br>Country | Aim<br>Design<br>Theoretical model | Sample | Intervention(s) | Outcomes/measures and follow-up period | Results |
|---|---|---|---|---|---|---|
| 12b | Pirhonen et al 2017 (16)<br><br>Sweden<br><br>(One study reporting two papers). | To study the effects of person-centred care provided to patients with acute coronary syndrome, using four different health-related outcome measures and to examine the performance of these outcomes when measuring person-centred care.<br><br>RCT<br><br>Person-centred care according to the framework by the Gothenburg Centre for Person-Centred Care (GPCC) | The intervention n= 94 and control n=105 patients.<br><br>All other details as above | 1) Patients and clinician Hansson s identify and discuss problems caused by or related to the patient's condition(s), giving due consideration to both clinical tests and treatments and the practical, social, and emotional effects of their condition(s) and treatment(s) on their daily lives.<br>2) They then engage in a shared decision-making process involving goal setting and action planning, focused on determining priorities, agreeing about realistic objectives, solving specific problems, and identifying relevant sources of support.<br>3) The agreed plan is documented and followed up.<br><br>Both groups received six-months of standard care comprised of a sequence of inpatient care, hospital- | 1. General self-efficacy<br><br>2. Quality of life: EQ-5D<br><br>3. Physical activity: Grimby scale<br><br>4.Return to work | 1. Patients in the intervention group reported significantly higher general self-efficacy than those in the control group six months after intervention start-up.<br><br>2-4. No siggnificant differences between the two groups. |

| Study Number | Author & Year/ Country | Aim Design Theoretical model | Sample | Intervention(s) | Outcomes/measures and follow-up period | Results |
|---|---|---|---|---|---|---|
| | | | | based outpatient care and primary care. | | |
| 12c | Fors et al (2017) (17)<br><br>Sweden | To assess the long-term effect of PCC in patients with acute coronary syndrome (ACS).<br><br>RCT.<br><br>Gothenburg PCC framework | N=199 with diagnosis of ACS and aged <75 years<br><br>n=94 intervention, Mean age (SD) 60.5 (9.3)<br>n=105 control, Mean age (SD) 61.3 (8.9) | PCC according to the Gothenburg PCC framework containing three routines for guiding PCC process to initiate, integrate and safeguard PCC in clinical practice. The PCC teams were trained through lecturers, workshops, and seminars on how to apply the intervention.<br><br>Comparison group received usual care comprising procedures in line with national guidelines. | Primary outcome:<br>1. Self-efficacy: general self-efficacy scale (GSE)<br><br>Measures completed at one month, two months, six months, and 24 months. | 1.The composite score improved in the PCC group compared with the control group at two-year follow-up (18.1% vs 10.5% p=0.127).<br>In the per-protocol analysis, the number of patients improving was significant in favour of the PCC (21.8% vs 10.5%, P=0.039). |
| 12d | Fors (2016)(18)<br><br>Sweden | Evaluating the effects of PCC intervention on self-efficacy after hospitalisations for acute coronary syndrome (ACS).<br><br>RCT. | N=177 patients <75 years hospitalised for ACS<br>n=84 intervention. Mean age 61.0 (SD 9.2)<br>n=93 control. | Provided by a group of health care professionals at the designated hospitals, outpatient clinics, and five primary care centres. Professionals were instructed through lecturers, workshops, seminars on application of PCC through | Patient confidence in managing coronary heart disease: Swedish Cardiac Self-Efficacy Scale (S-CSES). Assessments were conducted at baseline, one month and six months. | PCC improved significantly on the dimension of control symptoms (mean 0.81 vs -0.20; p=0.049) at 1 month. No significant differences were |

| Study Number | Author & Year/<br><br>Country | Aim<br>Design<br>Theoretical model | Sample | Intervention(s) | Outcomes/measures and follow-up period | Results |
|---|---|---|---|---|---|---|
| | | Person-centred care after acute coronary syndrome, from hospital to primary care - A randomised controlled trial"<br>Gothenburg PCC framework | Mean age 61.8 (SD 8.8) years. | teams (patient, physician, and registered nurse). Patients were engaged as partners in their care. Patients and professionals created a collaborative PCC plan within 48 hours of recruitment, then reviewed and revised at 48 hour intervals during admission. After discharge follow-up appointments were held at 4 and 8 weeks with further visits scheduled if required. Comparison received usual care following guidelines previously developed including follow up visits with a nurse at 2-3 weeks and a cardiologist at 6 weeks, then afterwards with their primary care physician at 8-10 weeks. | | seen at six months (p=0.366).<br>No significant difference between IG and CG in global cardiac self-efficacy at one month (p=0.299) or six months (p=0.577) |
| 12e | Fors et al 2016 (19)<br><br>Sweden | The aim of this study was to evaluate the effects of person-centred care (PCC) after acute coronary syndrome (ACS) in | As above (Sub study RCT) | As above | The primary endpoint was a composite of changes combining self-reported general self-efficacy with return to work or previous activity level and clinical | In the group of patients without postsecondary education (n=90) the composite score showed a significant improvement in |

| Study Number | Author & Year/<br><br>Country | Aim<br>Design<br>Theoretical model | Sample | Intervention(s) | Outcomes/measures and follow-up period | Results |
|---|---|---|---|---|---|---|
| | | relation to educational level of participants.<br><br>RCT<br><br>Gothenburg PCC framework | | | outcomes such as re-hospitalisation or death.<br><br>The General Self-Efficacy Scale (GSES) is a 10-item assessed the strength in personal beliefs to cope with and adapt to a variety of daily challenges.<br><br>The Saltin-Grimby Physical Activity Level Scale was used to determine return to previous activity level among those not working. The scale is a self-reported measure of physical activity.<br><br>At 6 months after discharge, each patient was assessed as improved, unchanged, or deteriorated.<br><br>To be classified as improved required | favour of the PCC intervention (n=40) vs. usual care (n=50) at six months (35.0%, n= 14 vs. 16.0%, n = 8; odds ratio (OR) = 2.8, 95% confidence interval (CI): 1.0–7.7, P = 0.041). In patients with postsecondary education (n= 109), a non-significant difference in favour of the PCC intervention (n= 54) vs. usual care (n = 55) was observed in the composite score (13.0%, n = 7 vs 3.6%, n = 2; OR = 3.9, 95% CI: 0.8–19.9, P = 0.097).<br><br>A higher proportion of patients receiving the PCC intervention improved according to the composite |

| Study Number | Author & Year/ Country | Aim Design Theoretical model | Sample | Intervention(s) | Outcomes/measures and follow-up period | Results |
|---|---|---|---|---|---|---|
| | | | | | improvement in the GSES with ≥5 units, return to work or previous activity level (improved from step 1 or at least unchanged from step 2) and no re-hospitalisation or death. A decrease in the GSES with ≥5 units or re-admission for unexpected cardiovascular reasons or death represented a deteriorated condition. Patients were dichotomised into two categories: improved vs. unchanged/deteriorated. | score: 21 of 94 (22%) in the intervention group vs. 10 of 105 (10%) in the controls, p = 0.013. The same outcome applied for the GSES criteria (≥5-point improvement in the GSES): 23 of 94 (24%) vs. 14 of 105 (13%), p = 0.043. A higher proportion of individuals in the intervention group that fulfilled the criteria for GSES also fulfilled the other two criteria included in the composite score: 21 of 23 (91%) vs. 10 of 14 (71%), although the difference was not statistically significant (p = 0.11). This applied to 100% of the patients with low educational |

| **Study Number** | **Author & Year/<br><br>Country** | **Aim<br>Design<br>Theoretical model** | **Sample** | **Intervention(s)** | **Outcomes/measures and follow-up period** | **Results** |
|---|---|---|---|---|---|---|
| | | | | | | level that received the PCC intervention which can be compared with the corresponding figures for patients with high education that received the intervention (7 of 9, 78%) (p = 0.06) or to the controls with a low educational level (8 of 11, 73%) (p= 0.04). |
| 12f | Fors et al 2015 (20)<br><br>Sweden | To evaluate if person-centred care can improve self-efficacy and facilitate return to work or prior activity level in patients after an event of acute coronary syndrome<br><br>RCT<br><br>Gothenburg PCC framework | N=199 patients with acute coronary syndrome <75 years.<br><br>n=94 intervention mean age 60.5 (SD 9.3)<br><br>n=105 control 61.3 (SD 8.9) | In the intervention group a person-centred care process was added to treatment as usual, emphasising the patient as a partner in care. Care was co-created in collaboration between patients, physicians, registered nurses and other health care professionals and documented in a health plan. A team-based partnership across three health care levels included transparent knowledge | 1. Main outcome measure was a composite score of changes in general self-efficacy ≥ 5 units, return to work or prior activity level and re-hospitalisation or death.<br><br>*Self-efficacy: General Self-Efficacy Scale* (GSE scale) a 10-item self-assessment questionnaire designed to measure a broad and stable sense of personal | 1. The composite score showed that more patients (22.3%, n = 21) improved in the intervention group at 6 months compared to the control group (9.5%, n = 10) (odds ratio, 2.7; 95% confidence interval: 1.2–6.2; P = 0.015). The effect was driven by improved self-efficacy ≥ 5 units in the |

| Study Number | Author & Year/<br><br>Country | Aim<br>Design<br>Theoretical model | Sample | Intervention(s) | Outcomes/measures and follow-up period | Results |
|---|---|---|---|---|---|---|
| | | | | about the disease and medical state to achieve agreed goals during recovery<br><br>All gPCC professionals had received training in the theory and practice of gPCC through lectures, seminars and workshops and were given practice in how to formulate and execute gPCC plans. Training emphasised the importance of seeing the patient as a person with needs as well as resources and of a person-centred dialogue as a basis for engaging patients as actively involved partners in their own care. | competence to deal effectively with a variety of stressful situations<br><br>2. Physical activity: Saltin Grimby Physical Activity Level Scale (SGPALS) is a validated measure of self-reported physical activity.<br><br>Questionnaires were completed by patients at baseline in hospital and at four, eight and 24 weeks per post. | intervention group. Overall general self-efficacy improved significantly more in the intervention group compared with the control group (P = 0.026).<br><br>2. There was no difference between groups on re-hospitalisation or death, return to work or prior activity level. |
| 12g | Wolf et al 2016 (21)<br><br>Sweden | To investigate the effect of an eHealth diary and symptom-tracking tool in combination with PCC for patients with acute coronary syndrome (ACS). | This was a sub-study of a RCT investigating the effects of PCC in patients hospitalized with ACS. | Patients in the intervention arm could choose to use a Web-based or mobile-based eHealth tool, or both, for at least 2 months after hospital discharge. | The primary end point was a composite score of changes in general self-efficacy: General Self-Efficacy Scale (GSES) using the Swedish version. | In the intervention arm, n=37 (39%) used the eHealth tool at least once after the index hospitalization. Most of these (24/37, 65%) used the |

| Study Number | Author & Year/<br><br>Country | Aim<br>Design<br>Theoretical model | Sample | Intervention(s) | Outcomes/measures and follow-up period | Results |
|---|---|---|---|---|---|---|
| | | | N=199 patients with ACS aged <75 years were randomly assigned to a PCC intervention (n=94) or standard treatment (control group, n=105)<br><br>Group 1: Person-centred care plus eHealth (n=37)<br><br>Group 2: Person-centred care only (n=57)<br><br>Group 3: Control (n=105) | A registered nurse at the hospital asked all of the patients in the eHealth group if they were interested in using the eHealth tool. Patients had the opportunity to borrow a mobile phone with the eHealth app preinstalled or to download it for use on their own mobile phone. An introductory demonstration, which required the patient to test the eHealth tools, was provided by a registered nurse who was familiar with the study so that patients could start using the tools freely during their hospital stay. Patients also had access to a video demonstration online for further information. The patients themselves decided on the frequency and patterns of use of the eHealth tools. Access to the webpage had no time restriction. | | mobile app and not the Web-based app as the primary source of daily self-rating input. Patients used the eHealth tool a mean of 38 times during the first 8 weeks (range 1–118, SD 33) and 64 times over a 6-month period. Patients who used the eHealth tool in combination with the PCC intervention had a 4-fold improvement in the primary end point compared with the control group (odds ratio 4.0, 95% CI 1.5–10.5; P=.005). This improvement was driven by a significant increase in general self-efficacy compared with the control group (P=.011). |

| Study Number | Author & Year/ Country | Aim Design Theoretical model | Sample | Intervention(s) | Outcomes/measures and follow-up period | Results |
|---|---|---|---|---|---|---|
| | | | | Patients in the control group were managed according to standard rehabilitation, which followed guideline-directed care that was compliant with Swedish standards. | Return to work or prior activity level, and rehospitalization or death 6 months after discharge.<br><br>Patients filled out the GSES instrument at baseline at the hospital, and at 4 weeks, 8 weeks, and 6 months. | Patients in the PCC group who did not use the eHealth tool (n=57) showed a nonsignificant composite score improvement compared with those in the control group (n=105) (odds ratio 2.0, 95% CI 0.8–5.2; P=.14).<br><br>There were 6 events in the PCC + eHealth group (1 death, 5 readmissions), 12 events in the PCC group without eHealth (3 deaths, 9 readmissions), and 16 events in the control group (2 deaths, 14 readmissions). The proportion of patients who returned to work was similar between |

| Study Number | Author & Year/ Country | Aim Design Theoretical model | Sample | Intervention(s) | Outcomes/measures and follow-up period | Results |
|---|---|---|---|---|---|---|
| | | | | | | groups at 6 months (PCC + eHealth 30/34, 88%; PCC no eHealth 47/53, 89%; control 89/98, 91%). |
| 13 | Zakrisson (2019) (22)<br><br>Sweden | To test a self-management intervention in primary health care (PHC) for patients with COPD or chronic heart failure (CHF) on self-efficacy, symptoms, functioning and health<br><br>Multi-centre RCT<br><br>Based on Bandura's theory of self-efficacy | N=150 patients with COPD or CHF from 9 PHC n=73 intervention group, mean age 74.0 (SD 7.4) n=77 control group, mean age 71.4 (SD 8.9) | Intervention: Delivered by a physiotherapist and a nurse who had undertaken a 2-day training programme. Groups of 3 COPD and 3 CHF patients and their relatives attended six 90-minute meetings every other week for a total of 6 meetings. Patients created individual action plans based on personal problems and goal setting discussions. Patients were supported to practice skills and gain knowledge for better self-management and behavioural changes. Further meetings at 6 and 9 months to study long term effects.<br><br>Control: details not provided | 1. Self-efficacy: perceived self-efficacy for fatigue self-management scale (PSEFSM)<br><br>2. Anxiety and depression: Hospital Anxiety and Depression Scale (HADS)<br><br>3. Dyspnoea: modified Medical Research Council dyspnoea scale (mMRC) and New York Heart Association scale (NYHA)<br><br>4. Fatigue Impact Scale (FIS)<br><br>5. Canadian Occupational | 1. No significant change of score at 3 or 12 months for either group.<br><br>2. No significant change of score at 3 or 12 months for either group.<br><br>3. No significant change of score at 3 or 12 months for either group.<br><br>4. No significant change of score at 3 or 12 months for either group.<br><br>5. Significant improvement in IG |

| Study Number | Author & Year/<br><br>Country | Aim<br>Design<br>Theoretical model | Sample | Intervention(s) | Outcomes/measures and follow-up period | Results |
|---|---|---|---|---|---|---|
| | | | | | Performance Measure (COPM)<br><br>6. Six-minute walking distance test (6MWD)<br><br>7. 36 Item Short Form Survey (SF-36)<br>COPM assessed at baseline and 3 months. All other measures collected at baseline, 3 months and 1 year. | group from baseline to 3 months (performance scores 4.7 and 5.3, p=0.04, satisfaction scores 4.5 and 5.1, p=0.03)<br><br>6. No significant change of score at 3 or 12 months for either group<br><br>7. Statistically significant improvement on social function subscale for IG between baseline and 1 year for IG (-8.3 vs 2.6, p=0.005). All other subscales no significant change. |

| Study Number | Author & Year/ Country | Aim Design Theoretical model | Sample | Intervention(s) | Outcomes/measures and follow-up period | Results |
|---|---|---|---|---|---|---|
| 14 | Arian (2018) (23)<br><br>Iran | To investigate the effect of a holistic care programme (HCP) on the reduction of iron overload in patients with beta-thalassaemia major<br><br>RCT | N=90 patients with beta-thalassaemia major referred to a large thalassaemia centre in Iran<br>n=45 intervention, mean age 25.58 (SD 3.92)<br>n=45 control, mean age 23.91 (SD 5.03) | Intervention: Patients attended the HCP over 8 weeks. This comprised individual counselling for four 45-60 min sessions, group training for four 60-90 min sessions and rehabilitation for 20 sessions<br><br>Control: Routine care at the clinic for 8 weeks | Primary outcomes:<br>1. Change in serum ferritin at three months (mg/L)<br><br>2. Change in iron level at three months (micrograms/dL)<br><br>Secondary outcomes:<br>3. Change in serum ferritin 1 year and 2 years post intervention<br><br>4. Total iron binding capacity at three months<br><br>5. Six-minute walk test (6MWT) at three months (metres) | 1. Significantly greater reduction in IG (mean difference between groups -1180.84mg/L, p=0.001)<br><br>2.Significantly greater reduction in IG (mean difference -65.555micrograms/dL, p=0.002)<br><br>3. No significant difference comparing IG and CG (p=0.07). Significant reduction within IG at 1 year (p=0.001) and 2 years (p=0.001).<br><br>4. Not significant (mean difference 8.33, p=0.724)<br><br>5. Significant improvement in IG compared to CG |

| **Study Number** | **Author & Year/<br><br>Country** | **Aim<br>Design<br>Theoretical model** | **Sample** | **Intervention(s)** | **Outcomes/measures and follow-up period** | **Results** |
|---|---|---|---|---|---|---|
| | | | | | 6. Haemoglobin (Hb) at three months | (mean difference 99.95m, p=0.001)<br><br>6. No significant difference (mean difference -0.27, p=0.425) |
| 15 | Eggers et al 2018 (24)<br><br>Germany | To assess whether a community-based, open-label, integrated approach improves QoL in PD patients.<br><br>RCT | N=150 Intervention group (IG), mean age (SD) 69.8 ( 8.4)<br><br>and 150 Control group (CG), mean age (SD) 69.9 (7.8) | The interventional group (IG) received an individually tailored therapy plan and additional home visits.<br><br>Patients randomly assigned to a control group (CG), received standard German neurological treatment | Primary outcome<br>1. QoL: compared the differential change of Parkinson's Disease Questionnaire (PDQ-39) from baseline to 6-month follow-up between CG and IG.<br><br>2. Mood: Beck Depression Inventory (BDI-2)<br><br>3. Motor: (United Parkinson's Disease Rating scale, Part III, UPDRS-III) | 1. PDQ-39 significantly improved in the IG compared to the CG over the 6-month period The mean group difference as a change from baseline over 6 months was 2.20 points (95% CI − 4.4 to − 0.1), p = 0.044.<br><br>2. No significant differences<br><br>3. For motor symptoms, there was a significant reduction in |

| Study Number | Author & Year/<br><br>Country | Aim<br>Design<br>Theoretical model | Sample | Intervention(s) | Outcomes/measures and follow-up period | Results |
|---|---|---|---|---|---|---|
| | | | | | 4. Non-motor functioning: Nonmotor Symptom Score, NMS-Score<br><br>5. Cognition: Parkinson | UPDRS part III over the first 3 months in the IG ($p < 0.001$), and a significant between-group difference ($p = 0.003$). Over the 6-month period, UPDRS-III significantly improved in the IG compared to the CG ($p \leq 0.001$). The mean group difference as a change from baseline over 6 months was 3.3 points (95% CI − 4.9 to − 1.7; p<0.001).<br><br>4. The scores of the PD-NMS improved after 6 months in favour of the IG (mean change 11.3, 95% CI − 17.1 to − 5.5; p<0.001). |

| Study Number | Author & Year/<br><br>Country | Aim<br>Design<br>Theoretical model | Sample | Intervention(s) | Outcomes/measures and follow-up period | Results |
|---|---|---|---|---|---|---|
| | | | | | Neuropsychometric Dementia Assessment, (PANDA)<br><br>Data collected at baseline, three and six months. | 5. No significant differences |
| 16 | Fors et al (2018) (25)<br><br>Sweden | To evaluate the effects of person-centred support via telephone in two chronically ill patient groups, chronic obstructive pulmonary disease (COPD) and/or chronic heart failure (CHF).<br><br>RCT<br><br>Person-centred care according to the framework by the Gothenburg Centre for Person-Centred Care (GPCC) | N=221 patients ≥50 years with COPD and/or CHF<br><br>n=103 intervention Mean age (SD) 78.3 (9.5)<br><br>n=118 control Mean age (SD) 76.9 (8.3) | Patients in the intervention group were telephoned one to four weeks after discharge by a registered nurse initially to co-create a person-centred health plan with the patient and subsequently to discuss and evaluate the plan.<br><br>Nurse's initially received extensive training in person-centred communication and a two day dedicated education about CHF and COPD.<br><br>Patients in the control care group received usual care and were managed using existing guidelines for the | 1. compost score in general self-efficacy: General Self-Efficacy (GSE) | 1. No significant differences between the two groups (57.6%, n = 68 vs. 46.6%, n = 48; OR = 1.6, 95% CI: 0.9±2.7; P = 0.102).<br><br>Significantly more patients in the control group had deteriorated in self-efficacy (GSE scores ≥5 units) than in the intervention group at three months (23.7%, n = 28 vs. 11.7%, n = 12; OR = 2.4, 95% CI: 1.1±4.9; P = 0.022) and at six months follow-up (22.9%, n |

| Study Number | Author & Year/ Country | Aim Design Theoretical model | Sample | Intervention(s) | Outcomes/measures and follow-up period | Results |
|---|---|---|---|---|---|---|
| | | | | diagnosis and treatment of acute and chronic heart failure. | | = 27 vs. 9.7%, n = 10; OR = 2.8, 95% CI: 1.3±6.0; P = 0.011).<br><br>Improvement in GSE was significantly greater in favour of the intervention group at both three months (0.7 (mean) ± 5.8 (SD); n = 79 vs. -2.2 (mean) ± 6.1 (SD); n = 89; P = 0.010) and six months (0.9 (mean) ± 6.4 (SD); n = 69 vs. -2.0 (mean) ± 6.8 (SD); n = 85; P = 0.006 |
| | | | | | 2. Re-hospitalization and death<br><br>Each patient classified as deteriorated, improved or unchanged: Deteriorated: if GSE had decreased by ≥5 units | 2. There were 49 clinical events (14 deaths, 35 re-admissions) in the control group and 41 in the intervention group (9 deaths, 32 re-admissions). |

| Study Number | Author & Year/<br><br>Country | Aim<br>Design<br>Theoretical model | Sample | Intervention(s) | Outcomes/measures and follow-up period | Results |
|---|---|---|---|---|---|---|
| | | | | | OR re-admitted to hospital for unscheduled reasons related to COPD and/or CHF OR had died;<br><br>-Improved: if GSE had increased by≥5 units AND the patient had not been hospitalized for unscheduled reasons related to COPD and/or CHF AND not died.<br>-Unchanged: neither deteriorated nor improved according to the above criteria.<br><br>GSE completed at baseline, three and at six months. | Per-protocol analysis (n = 202) of the composite score showed that more patients deteriorated in the control group than in the intervention group (57.6%, n = 68 vs. 42.9%, n = 36; OR = 1.8, 95% CI 1.0±3.2; P = 0.039). |
| 17 | Reed et al (2018) (26)<br><br>Australia | To determine whether a clinician-led chronic disease self-management support (CDSMS) program improves the overall self-rated health level of older Australians | N=254 patients over 60 years with at least 2 chronic conditions from 5 general practices<br>n=127 intervention, of which 48% 60-75 | Intervention: CDSMS program which uses a set of tools and structured process that enables clinicians and patients to collaboratively assess self-management behaviour, identify problems, set goals | Primary outcome measure:<br>1. Self-rated health measured with 5-point likert scale | 1.IG more likely to report better health than CG (OR 2.5, p=0.023) at 6 months. Most participants in both IG and CG reported no change to self-reported health from |

| Study Number | Author & Year/<br><br>Country | Aim<br>Design<br>Theoretical model | Sample | Intervention(s) | Outcomes/measures and follow-up period | Results |
|---|---|---|---|---|---|---|
| | | with multiple chronic health conditions<br><br>RCT | years, 36% 76-85 years and 16% >85<br>n=127 control, of which 46% 60-75 years, 40% 76-85 years and 14% >85 years | and develop individual care plans.<br><br>Control: Semi-structured positive attention program. Participants receive information relevant to their condition and scheduled contact with their clinician who was instructed to provide positive attention. All participants received 3 home visits and four follow up phone calls over 6 months from a clinician. | Secondary outcome measures:<br>2. Health status<br><br>3. Health behaviours<br><br>4. Self-efficacy<br><br>5. Health Education Impact Questionnaire (heiQ)<br><br>6. Health care utilisation Assessed at baseline and 6 months. | baseline to 6 months (57% IG and 69% CG). Improved health from baseline to 6 months reported in 34% of IG and 19% CG.<br><br>Secondary outcomes: 2-6 No statistically significant between group differences for any outcome |

| Study Number | Author & Year/ Country | Aim Design Theoretical model | Sample | Intervention(s) | Outcomes/measures and follow-up period | Results |
|---|---|---|---|---|---|---|
| 18 | Schäfer et al. (2018) (27)<br><br>Germany | To determine if patient-centred communication leads to a reduction of the number of medications taken without reducing health-related quality of life<br>Two-arm cluster-randomised controlled trial | N=604 patients aged 65-84 with at least three chronic conditions recruited from 55 primary care practices<br>n=299 Intervention group, mean age 73.3 (SD 4.8)<br>n=305 control group, mean age 73.5 (SD 5.0) | Intervention: Three 30-minute PC talks with a GP over 12 months to identify treatment targets and priorities of the patient, review of all medications and discuss goal attainment and future treatment targets<br><br>Control: care as usual (details not provided) | Primary outcomes:<br>1. Change in number of medications taken by the patient<br><br>2. Health related quality of life: EQ-5D<br><br>Secondary outcomes:<br><br>3. Patient satisfaction<br><br>4. Patient empowerment<br><br>5. GP's knowledge about medication taken by the patient<br><br>6. Healthcare use | 1. No statistically significant difference between IG and CG for change in number of medications (p=0.43)<br><br>2. No significant difference between groups (p=0.34)<br><br>3. No effect<br><br>4. No effect<br><br>5. No effect (p=0.772)<br><br>6. IG had greater contact with GPs than CG (p=0.010) but fewer days in hospital (p=0.006) and fewer attendances at |

| Study Number | Author & Year/<br><br>Country | Aim<br>Design<br>Theoretical model | Sample | Intervention(s) | Outcomes/measures and follow-up period | Results |
|---|---|---|---|---|---|---|
| | | | | | | physical, occupational or speech therapy units (p=0.044) |
| 19 | Thom et al 2018 (28)<br><br><br>USA | To determine the benefit of health coaching for patients with moderate to severe COPD relative to usual care.<br><br>RCT | N=192 COPD patients: n=100 intervention, mean age (SD) 60.7 (8.0).and n= 92 control mean age (SD) 61.9 (7.2). | Patients randomized to the health coaching arm received health coaching for 9 months. Each health coach worked with a total of 50 patients with a maximum caseload of 30 patients at any given time. Health coaches were expected to complete an initial visit within 2–3 weeks of enrollment; to meet in person with the patient at least three additional times over the course of the study; and to have a phone check-in call at least every 3 weeks, including within 2 weeks after each medical visit (minimum of 13 phone check-ins over 9 mo). In-person visits could be at the | Primary outcomes:<br><br>1. COPD quality of life: Chronic Respiratory Disease Questionnaire (CRQ-SF)<br><br>2.dyspnoea: CRQ-SF dyspnoea subscale score<br><br>3. Number of COPD exacerbations: a standardized 6-minute walk test<br><br>4. Self-efficacy for COPD management | 1-9 There were no significant differences between study arms for any of the primary outcomes or for the secondary outcomes |

| Study Number | Author & Year/<br><br>Country | Aim<br>Design<br>Theoretical model | Sample | Intervention(s) | Outcomes/measures and follow-up period | Results |
|---|---|---|---|---|---|---|
| | | | | clinic, at the patient's home, or at a public location that afforded sufficient privacy. Additional contacts were guided by patient needs and preferences. Coaches were also expected to conduct at least one in-depth consultation with the study pulmonary nurse practitioner specialist and to attend medical visits between the patient and their PCP when possible. Health coaching focused on helping patients identify and achieve self-care goals for their COPD using techniques from motivational interviewing and adult learning models. Specific content included COPD education, action planning for exacerbations, teaching proper inhaler use, and facilitating consultation with a pulmonary nurse practitioner specialist.<br><br>Patients randomised to usual care continued to | 5. COPD symptoms and functional capacity: COPD Assessment Test<br><br>6. Lung function: spirometry as the percent predicted $FEV_1$,<br><br>7. Current smoking status: defined as any self-reported cigarette use in the past 30 days,<br><br>8. Number of bed days owing to respiratory problems in the past 4 weeks.<br><br>9. Knowledge of COPD: the percentage of correct responses to four questions developed for the present study.<br><br>10. Patient-reported quality of care: Patient Assessment of Chronic Illness Care | 10: Statistically significant differences between coaching and usual care (0.07 to 0.68 p=0.02). |

| Study Number | Author & Year/ Country | Aim Design Theoretical model | Sample | Intervention(s) | Outcomes/measures and follow-up period | Results |
|---|---|---|---|---|---|---|
| | | | | have visits with their PCP over the course of the 9-month period. They received any resources their provider and their clinic offered as part of standard care, including access to COPD educators, respiratory therapists, COPD education classes, pulmonary rehabilitation, smoking cessation classes, and pulmonary specialist referrals by the primary care clinician. | Outcomes at baseline, 3, 6, and 9 months. | |
| 20 | Armstrong et al (2017) (29)<br><br>Canada | To determine whether follow-up care delivered via a mobile app can be used to avert in-person follow-up care visits compared with conventional, in-person follow-up care in the first 30 days following ambulatory surgery<br><br>RCT | N=65 women undergoing elective breast reconstruction surgery<br>n=32 intervention, mean age 50.3 (SD12.3)<br>n=33 control, mean age 45.1 (SD 14.1) | Intervention: Planned clinic follow up replaced with daily use of QoC Health Inc mobile app. Allows users to submit photographs and responses to validated quality of recovery questionnaire and visual analogue scale for first 30 days post operatively. Surgeons follow patient reports on a web portal. | Primary outcome:<br>1. Total number of follow-up visits associated with the surgery at 30 days post-op.<br><br>Secondary outcomes:<br><br>2. Total number of telephone calls and emails to the healthcare team associated with the surgery at 30 days post-op. | 1. IG had fewer follow up visits than CG (mean 0.66 vs 1.64) IG 0.4 times less likely to attend in person (p<0.001)<br><br>2. No significant difference between IG and CG in telephone calls (mean 0.31 vs 0.3, IRR 1.03, p=0.95). |

| Study Number | Author & Year/<br><br>Country | Aim<br>Design<br>Theoretical model | Sample | Intervention(s) | Outcomes/measures and follow-up period | Results |
|---|---|---|---|---|---|---|
| | | | | Control: planned clinic follow up at 1 and 4 weeks post operatively | 3.Patient reported satisfaction and convenience scores: 5 point Likert scale<br><br>4. Post-operative complications: adverse events attributed to the surgery requiring a medical or surgical intervention<br>All outcomes measured at 30 days. | IG sent more emails than CG (mean 0.65 vs 0.15, IRR 4.13, p=0.05)<br><br>3. No significant difference between groups in satisfaction scores (IRR 0.95, p=0.7). IG had higher convenience scores than CG (IRR 1.39, p=0.08)<br><br>4. No difference in rates of complications between groups (p=0.42). |

| Study Number | Author & Year/<br><br>Country | Aim<br>Design<br>Theoretical model | Sample | Intervention(s) | Outcomes/measures and follow-up period | Results |
|---|---|---|---|---|---|---|
| 21 | Feldthusen et al 2017 (30) | To examine effects of person-centered physical therapy on fatigue and related variables in persons with rheumatoid arthritis (RA).<br><br>RCT<br><br>Gothenburg | Rheumatoid arthritis patients recruited at outpatient rheumatology clinic<br><br>(N=70): intervention group (n=36) mean age 54.2 (SD 8.5) and control group (n=34) mean age 52.7 (SD 10.9). | Each participant in the intervention group participated in the 12-week intervention of person-centered physical therapy. The goal of the intervention was, in partnership between participant and physical therapist, to devise a mutually agreed self-care plan that guided the participant in managing his or her fatigue and to effectively do so over time. The same physical therapist, experienced and specialized in RA management and person-centered care, conducted the intervention.<br>The intervention was initiated with an individual person-centered meeting. A self-care plan was jointly developed and focused on tailoring health-enhancing physical activity and balancing life activities | 1. Primary outcome was general fatigue (visual analog scale).<br><br>Secondary outcomes:<br>2. Multidimensional fatigue<br>(Bristol Rheumatoid Arthritis Fatigue Multi-Dimensional Questionnaire)<br><br>3. Fatigue-related variables (ie, disease, health, function).<br><br>Data collected at baseline, three and six months. | 1.General fatigue improved more in the intervention group than the reference group (P=.042). Improvement in median general fatigue reached minimal clinically important differences between and within groups at post test and follow-up.<br><br>2-3 Improvement was also observed for anxiety (P=.0099), and trends toward improvements were observed for most multidimensional aspects of fatigue (P=.023-.048), leg strength/endurance (P=.024), and physical activity (P=.023). Compared |

| **Study Number** | **Author & Year/ Country** | **Aim Design Theoretical model** | **Sample** | **Intervention(s)** | **Outcomes/measures and follow-up period** | **Results** |
|---|---|---|---|---|---|---|
| | | | | The reference group continued with regular activities; both groups received usual health care | | with the control group at follow-up, the intervention group improvement was observed for leg strength/endurance (P=001), and the trends toward improvements persisted for physical (P=041) and living related (P=031) aspects of fatigue, physical activity (P=019), anxiety (P=015), self-rated health (P=.010), and self-efficacy (P=046). |
| 22a | Hansson et al 2017 (31)<br><br>Sweden | To compare a person-centred care intervention in terms of health-related quality of life, disease-specific symptoms or problems, with traditional care as a control group for patients with head and neck cancer. | N=96 patients with head and neck cancer (HNC) attending oncology care<br><br>n=54 intervention mean age 61 (SD 7.8) | Patients attended meetings with the intervention nurse, oncology specialist. The first meeting included a description of the study as well as information needed about the health-care plan. The plan was designed and developed according to a basic model from Gothenburg PCC | Health related Quality of Life (HRQoL): European Organization for Research and Treatment of Cancer (EORTC) QLQ-C30 and the EORTC QLQ-35 version 3.0. | HRQoL was nonsignificant in all instruments. gPCC-group tended, from the 10th week, to be better than those in the control group (CG) and were, from the 18th week, statistically significantly better in |

| Study Number | Author & Year/<br><br>Country | Aim<br>Design<br>Theoretical model | Sample | Intervention(s) | Outcomes/measures and follow-up period | Results |
|---|---|---|---|---|---|---|
| | | <br>RCT<br><br>Gothenburg PCC | n=42 control mean age 62 (SD 10.9) | (gPCC) and further adapted to suit patients with HNC and scheduled by the nurse and patient together. The health-care plan comprised self-management goals that were formed in partnership between the patient and the nurse. Each patient was encouraged to reflect on their self-management goals, how to reach them, and to anticipate barriers; and to refine the plan. The health plan includes both short- and long-term goals for the patient along with the actions needed to reach each goal.<br>The plan is a "living" document specific to each patient, in which the goals and actions are tracked and revised over time. The patient was also given a direct telephone number to reach the nurse specialist if they had any questions about anything relating to their treatment and | Data collected at baseline, weeks 4, 10, 18 and 52. | the gPCC-group in terms of HNC-specific problems (QLQ-35), swallowing (p = 0.014), social eating (p = 0.048) and feeling ill (p = 0.021). |

| Study Number | Author & Year/<br><br>Country | Aim<br>Design<br>Theoretical model | Sample | Intervention(s) | Outcomes/measures and follow-up period | Results |
|---|---|---|---|---|---|---|
| | | | | wellbeing. The nurse documented the health-care plan in the medical record.<br><br>Patients randomized to the control group received usual care and return visits were scheduled according to the treatment procedure based on the Regional care program for patients with HNC which included post-treatment follow up visits to an oncologist at 6–8 weeks and from then on every third month for 2 years. | | |
| 22b | Gyllensten et al 2019 | The aim was to examine the cost-effectiveness, including healthcare and productivity costs, of a person-centred care intervention versus standard medical care among patients with Head and Neck Care. | As above | As above | Health-related quality of life: EuroQol (Group's five-dimension health state questionnaire (EQ-5D™), | No significant differences<br><br>(The average total cost was Euro (EUR) 55,544 (95% confidence interval: EUR 48,474–62,614) in the intervention group |

| Study Number | Author & Year/ Country | Aim Design Theoretical model | Sample | Intervention(s) | Outcomes/measures and follow-up period | Results |
|---|---|---|---|---|---|---|
| | | RCT<br><br>Gothenburg PCC | | | At baseline, 4 weeks, 10 weeks, 18 weeks, and 52 weeks. | and EUR 57,443 (EUR 48,607–66,279) among controls, with similar health-related quality of life) |
| 23 | Ko et al (2017) (32)<br>Hong Kong | To evaluate whether comprehensive care programme with multidisciplinary input will decrease hospital readmissions and length of hospital stay for patients with COPD<br><br>RCT. | N=180 COPD patients admitted with an acute exacerbation.<br>n=90 intervention. Mean age 74.9 (SD=7.9) years,<br><br>n=90 control. Mean age 74.6 (SD=8.6). | Individualised education sessions including anatomy and physiology, pathophysiology of COPD, smoking cessation, techniques of using medication, management of dyspnoea, self-management of exacerbations, coping, relaxation techniques, social and community support.<br>Patients were provided with telephone number to call and seek advice from respiratory nurse during office hours. | Primary Outcome:<br>1. Hospital readmission rate at one year.<br><br>Secondary outcomes:<br>2. Length of stay (LOS)<br><br>3. Dyspnoea: Modified Medical Research | 1. At 12 months relative risk of readmission was 0.668, p=0.047 for the intervention group compared with the control group.<br><br>2. at 12 months IG had a shorter LOS 4.59 vs 8.86, p<0.001<br><br>3. IG had greater improvement on |

| Study Number | Author & Year/<br><br>Country | Aim<br>Design<br>Theoretical model | Sample | Intervention(s) | Outcomes/measures and follow-up period | Results |
|---|---|---|---|---|---|---|
| | | | | Subsequently patients received three monthly telephone calls from respiratory nurse for one year to assess their condition and answer queries.<br><br>Comparison group received usual care, the attending physician determined the patient's medication and follow-up as normal practice. | Council Dyspnoea Scale (MMRC)<br><br>4. QoL: St George's Respiratory Questionnaire.<br><br>5. Lung function $FEV_1$/FVC ratio<br><br>6. Exercise capacity: 6 minute walk test<br><br>7. Mortality | MMRC -0.1 vs 0.2, p=0.003<br><br>4. SGRQ: Improvement for IG at 12 months, -6.9 vs -0.1, p=0.003<br><br>5. No significant difference between groups in change in lung function at 12 months (p=0.653)<br><br>6. No significant difference between groups in change in exercise capacity at 12 months (-10m vs -22.5m, p=0.233)<br><br>7. Ten patients in IG and 12 in CG had died at 12 months. |
| 25 | Low et al (2017) (33)<br><br>Singapore | Evaluate the effectiveness of an integrated practice unit and modified virtual ward model in reducing readmission rates in | N=840 patients with one or more unscheduled readmissions in last 90 days and at high risk of | Intervention: Hospital care transferred to Integrated Practice Unit MDT on randomisation. Intensive discharge planning including identifying and | Primary outcome:<br>1. Unplanned readmissions within 30 days of discharge | Primary outcome:<br>1. Readmission at 30 days was lower in the intervention group than the |

| Study Number | Author & Year/<br><br>Country | Aim<br>Design<br>Theoretical model | Sample | Intervention(s) | Outcomes/measures and follow-up period | Results |
|---|---|---|---|---|---|---|
| | | patients at highest risk of readmission.<br><br>RCT | readmission (LACE score >/=10)<br>n=420 intervention group, mean age 70.5 (SD 13.5)<br>n=420 control group, mean age 70.3 (SD 13.7) | addressing risk factors for readmission. All patients provided with individualised care plan on discharge. Phone call from nurse case manager within 72 hours of discharge and home assessment within 1 week plus review at Virtual Ward MDT.<br><br>Control: Standard hospital care | Secondary outcomes:<br>2. Unplanned readmissions within 90 and 180 days of discharge (visits/patient/month)<br><br>3. Emergency department attendance rate within 30, 90 and 180 days of discharge (visits/patient/month).<br><br>4. Probability of death up to 180 days | control group (0.25 vs 0.38, p=0.001)<br><br>2. Readmissions at 90 (0.67 vs 0.90, p=0.001) and 180 (1.05 vs 1.46, p=<0.001) days were lower in the intervention group than the control group.<br><br>3. ED visits were lower in the intervention group than the control group at 30 (0.26 vs 0.43, p=<0.001), 90 (0.66 vs 0.92, p=0.001) and 180 (1.14 vs 1.60, p<0.001) days.<br><br>4. 28% reduction in mortality in intervention group compared to control (HR 0.72, p<0.001). |

| Study Number | Author & Year/<br><br>Country | Aim<br>Design<br>Theoretical model | Sample | Intervention(s) | Outcomes/measures and follow-up period | Results |
|---|---|---|---|---|---|---|
| 25 | Wichit et al (2017) (34)<br><br>Thailand | To evaluate a theoretically driven family-oriented intervention to improve self-efficacy, self-management, glycaemic control and quality of life in T2D<br><br>RCT.<br><br>Bandura's self-efficacy theory | N=140 T2D patients.<br><br>n=70 experimental group, mean age 61.3 (SD=11.6) years;<br>n=70 control group, mean age 55.5 (SD=10.5) years. | Family-oriented programme (patients/family dyads) consisting of education classes, group discussions, home visit, and telephone follow-up. Participants learned specialised skills such as meal planning, physical activities, managing complications. Education sessions were delivered at baseline, week 5 and week 9.<br><br>Control received usual care consisting of blood sugar testing, physical examinations and medication follow-up | Primary outcome<br>1. Type 2 Diabetes (T2D) self-management: Summary of Diabetes Self-Care Activities Scale (SDSCA)<br><br>Secondary outcomes:<br><br>2. T2D self-efficacy: Diabetes Management Self-Efficacy Scale (DMSES) and Perceived Therapeutic Self-Efficacy Scale (PTES) | 1. At week 5 SDSCA increased from 80.9 to 96.5 in the intervention and decreased from 80.5 to 80.2 in the control, the results were significant between the two groups (p<0.001). At week 13 SDSCA was 1.2.8 in the intervention and 80.4 in the control (p<0.001).<br><br>2. At week 5 DMSES increased from 55.6 to 69.8 in the intervention, but decreased from 58.7 to 58.2 in the control (p<0.001)<br>At week 13 DMSES further increased to 76.0 in the intervention and slightly increased in |

| Study Number | Author & Year/<br><br>Country | Aim<br>Design<br>Theoretical model | Sample | Intervention(s) | Outcomes/measures and follow-up period | Results |
|---|---|---|---|---|---|---|
| | | | | | 3. Quality of life: Thai Version short-form Health Survey (SF-12) | the control to 60.7 (p<0.001).<br>At week 5 PTES increased from 32.4 in the intervention to 37.9 but decreased from 34.8 to 33.7 in the control group (p<0.001). at week 13 PTES increased in both groups to 40.8 in the intervention and 35.3 in the control group (p<0.001).<br><br>3. At week 5, Physical aspect of QoL increased in both groups from 46.7 to 50.0 in the intervention and 48.2 to 49.2 in the control (p=0.2), similar pattern occurred at week 13.<br><br>Mental aspect of QoL increased from 54.1 to 56.0 in the intervention group. |

| Study Number | Author & Year/ Country | Aim Design Theoretical model | Sample | Intervention(s) | Outcomes/measures and follow-up period | Results |
|---|---|---|---|---|---|---|
| | | | | | 4. Diabetes Knowledge: Diabetes Knowledge Questionnaire (DKQ)<br><br>5. HbA1c: extracted from patient's health records<br><br>Outcomes conducted at baseline and 3 weeks and 13 weeks (HbA1c | In the control group it remained at 54.3. (p=0.2). At week 13 QoL was 58.4 in the intervention and 54.7 in the control (p<0.001).<br><br>4. At week 5 DKQ was 17.1 from 10.7 in the intervention, while it was 11.7 from 10.6 in the control (p<0.001). At week 13 DKQ was 16.5 in the intervention group and 13.2 in the control group (p<0.001)<br><br>5. At baseline HbA1c was 7.0 in the intervention and 6.3 in the control. At week 13 it was 7.0 in the intervention and 7.3 in the control (p=0.2) |

| Study Number | Author & Year/<br><br>Country | Aim<br>Design<br>Theoretical model | Sample | Intervention(s) | Outcomes/measures and follow-up period | Results |
|---|---|---|---|---|---|---|
| | | | | | was assessed at baseline and week 13). | |
| 26a | Larsson et al 2015 (35)<br><br><br><br>Sweden | To examine the effects of a progressive resistance exercise program on muscle strength, health status, and current pain intensity in women with Fibromyalgia (FM).<br><br>RCT<br><br>Gothenburg PCC | N=130 women with FM, n=67 resistance exercise, n=63 mean age 50.8 (SD 9.05) relaxation therapy mean age 52 (SD 9.08) | The intervention: The resistance exercise program was performed twice a week for 15 weeks and was supervised by experienced physiotherapists. It was conducted at physiotherapy premises and at a local gym at four different sites in groups comprising five to seven participants to promote interaction between participants and to facilitate physiotherapeutic guidance. The intervention was preceded by an individual introductory meeting.<br>The meeting was commenced with a dialogue between the participant and the physiotherapist about the participant's earlier experiences and thoughts of exercise. | 1. The primary outcome was isometric knee-extension force (N) measured with a dynamometer (Steve Strong: Stig Starke HBI, Göteborg, Sweden) using a standard protocol.<br><br>Secondary outcomes were:<br>2. Fibromyalgia impact: the fibromyalgia impact questionnaire (FIQ) a disease-specific self-reported questionnaire that comprises ten subscales of disabilities and symptoms.<br><br><br><br>3. Current pain intensity: rated on a plastic 0-100 visual analogue scale | 1. Significantly greater improvement (p = 0.010) was found for isometric knee-extension force in favor of the resistance exercise group as compared to the active control group<br><br><br>2. Significantly greater improvement was observed in health status (FIQ total score) (p = 0.038) in the resistance exercise group compared to the active control group<br><br><br>3. Significantly greater improvement was observed |

| Study Number | Author & Year/ Country | Aim Design Theoretical model | Sample | Intervention(s) | Outcomes/measures and follow-up period | Results |
|---|---|---|---|---|---|---|
| | | | | The meeting also included exercise instructions, testing and adjustment of loads and modifications of specific exercises according to individual conditions and according to self-efficacy principles. The meeting resulted in a written protocol with descriptions of specific exercises and loads, which was used by each participant as an exercise program at each exercise session. The exercise was initiated at low loads, and possibilities for progressions of loads were evaluated every 3−4 weeks in dialogue between the physiotherapist and participant.<br><br>The control group was the relaxation therapy was performed twice a week for 15 weeks and was guided by experienced physiotherapists. It was conducted at physiotherapy premises at four different | with a moveable cursor along a line and anchors at the extremes.<br><br>4. The six-minute walk test (6MWT), a performance-based test that measures total walking distance (m) during a period of 6 minutes | in current pain intensity (VAS) (p = 0.033) in the resistance exercise group compared to the active control group<br><br>4. Significantly greater improvement was observed in the 6MWT (p = 0.003) in the resistance exercise group compared to the active control group |

| Study Number | Author & Year/ Country | Aim Design Theoretical model | Sample | Intervention(s) | Outcomes/measures and follow-up period | Results |
|---|---|---|---|---|---|---|
| | | | | sites in groups comprising five to eight participants and was preceded by an individual introductory meeting at the premises, which included instructions and allowed for preparations and modifications of practical matter such as positioning and the use of mattresses and pillows to reach a good level of comfort. The relaxation therapy performed a series of mental exercises including relaxation and autosuggestion. The physiotherapist guided the participants through their bodies, during approximately 25 minutes, by focusing their minds on the bodily experience of relaxation and letting the body part in focus rest on the ground. This was repeated for each specific body-part, aiming at feeling as relaxed as possible in | | |

| Study Number | Author & Year/ Country | Aim Design Theoretical model | Sample | Intervention(s) | Outcomes/measures and follow-up period | Results |
|---|---|---|---|---|---|---|
| | | | | the whole of the body at the end of the session. Participants were invited to share experiences and ask each other and the physiotherapist questions and continued thereafter with the stretching exercises. | | |
| 26b | Ericsson et al 2016 (36) | This sub-study aimed to examine the effects of a person-centered progressive resistance exercise program on multiple dimensions of fatigue in women with fibromyalgia (FM), and to investigate predictors of the potential change in fatigue. | As above | as above | Outcomes were:<br><br>1. Five dimensions of fatigue measured with the Multidimensional Fatigue Inventory (MFI-20). | 1.A higher improvement was found at the post-treatment examination for change in the resistance exercise group, as compared to change in the active control group in the MFI-20 subscale of physical fatigue (resistance group change –1.7, SD 4.3, controls change 0.0, SD 2.7, p = 0.013), with an effect size of 0.33. |

| Study Number | Author & Year/ Country | Aim Design Theoretical model | Sample | Intervention(s) | Outcomes/measures and follow-up period | Results |
|---|---|---|---|---|---|---|
| | | | | | 2. FIQ fatigue (0–100) The VAS for fatigue included in the Fibromyalgia Impact Questionnaire (FIQ) was used as a one-dimensional measure of fatigue.<br><br>3. Pittsburgh Sleep Quality Index (PSQI) (0–21) The PSQI assesses sleep quality and disturbances over a 1-month period.<br><br>4. Pain catastrophizing scale (PCS) (0–52) The | 2. The resistance exercise group improved in the FIQ for fatigue over time from baseline to post treatment (mean difference −8.6, SD 21.2, p = 0.002).<br><br>3. The resistance exercise group improved over time in the PSQI subscale for sleep quality (mean difference −0.2, SD 0.8, p = 0.047), while the active control group improved in the PSQI subscale for need of medications to sleep (mean difference 0.3 SD 1.0, p = 0.036)<br><br>4. The resistance exercise group |

| Study Number | Author & Year/ Country | Aim Design Theoretical model | Sample | Intervention(s) | Outcomes/measures and follow-up period | Results |
|---|---|---|---|---|---|---|
| | | | | | PCS assesses pain-related catastrophic thinking.<br><br>5. Hospital Anxiety and Depression Scale (HADS) (0–21) | improved significantly over time in all three PCS subscales and the PCS total score (mean difference in PCS total score −2.7 SD 7.6, p = 0.004). In the active control group there was a tendency towards improvement in two PCS subscales and the PCS total score (p = 0.051–0.056).<br><br>5. No significant changes during the study period were found within any of the groups for HADS anxiety or HADS depression. |

| Study Number | Author & Year/<br><br>Country | Aim<br>Design<br>Theoretical model | Sample | Intervention(s) | Outcomes/measures and follow-up period | Results |
|---|---|---|---|---|---|---|
| 27a | Hansson et al 2016 (37)<br><br>Sweden | To estimate the cost–utility of PCC when compared with conventional care in patients hospitalized for worsening chronic heart failure.<br><br>A controlled before and after design<br><br>Gothenburg PCC framework | N=248 CHF patients<br>n=125 intervention, mean age 77 (SD 11)<br><br>n= 123 control, mean age 80 (SD 9) | Larsson Larsson Larsson | Costs of care:<br>An assessment of health-related quality of life used the EQ-5D 3L instrument at baseline and at three months after discharge to usual care.<br><br>The quality of life weight was then used to calculate<br>QALYs. This measure combines years of life with quality of life so that the QALY, as a result of a treatment, can consist in increasing life expectancy and/or increased quality of life. QALY calculations were made on an individual level, reflecting the change from baseline to three months, assuming a linear increase in quality of life (QoL) between the two measurements. | We found that PCC resulted in lower costs (€863 per patient, p=0.026) and generated marginally more health benefits than conventional care.<br><br>The costs for those who actually received PCC, per protocol (PP) (63%) were significantly (p=0.026) lower than for those in the conventional care group, with an incremental cost-saving of €863. For the first three months, patients in the conventional care group showed decreasing health-related quality of life, with a corresponding improvement in the PCC(PP) group. |

| **Study Number** | **Author & Year/ Country** | **Aim Design Theoretical model** | **Sample** | **Intervention(s)** | **Outcomes/measures and follow-up period** | **Results** |
|---|---|---|---|---|---|---|
| 27b | Ulin et al 2016 (38)<br><br>Sweden | To evaluate whether proactive care-planning based on the Gothenburg person-centred care (gPCC) model leads to improved efficiency in discharge procedures compared with usual care in patients hospitalized for worsening chronic heart failure.<br><br>A controlled before and after design<br><br>Gothenburg PCC framework | As above | The gPCC health plan starts with the patient narrative, which includes information regarding everyday life and symptoms prior to and during the worsening of the condition. In addition, the patient's resources are identified, including motivations and goals. The social situation and the possible need for additional support at home after discharge from hospital are also of importance. Finally, within 24–48 hours, all information and facts are summarized and written in the gPCC health plan, which also includes planned investigations, treatment goals and length of stay at hospital.<br><br>Thereafter, the first notification can be sent to the patient's municipal home care service and to the primary healthcare | The first endpoint was the number of days from admission to Step 1, the first notice to the municipality, including the municipal home care service and the primary healthcare service.<br><br>The second endpoint was the number of days from admission to the second notice to the municipal home care service and to the primary healthcare service confirming the discharge planning conference, or Step 2. | During hospitalization, first notifications (Step 1) to the patients' municipal home-care services and/or round-the-clock home nursing care services were more frequent in the per-protocol gPCC group (33.8%) compared with the usual care group (12.1%), but not significant.<br><br>During hospitalization, the number of days from admission to notices to the patients' municipal homecare services and/or round-the-clock home nursing care services for confirmed discharge planning conferences (the second notification |

| Study Number | Author & Year/<br><br>Country | Aim<br>Design<br>Theoretical model | Sample | Intervention(s) | Outcomes/measures and follow-up period | Results |
|---|---|---|---|---|---|---|
| | | | | service, which is Step 1. The patient and healthcare professionals discuss the gPCC health plan and reach an agreement. The gPCC health plan is regularly evaluated (and if necessary, revised) in all aspects of care (such as symptoms, resources, management and treatment) by the patient and the healthcare professionals during the hospitalization. The gPCC health plan forms the basis for the second notice to the municipal home care service and to the primary healthcare service with an accurate and detailed description of the patient's anticipated status (including for example symptoms and resources) at discharge, as well as any anticipated discharge planning conference in the hospital, which is Step 2. The third notice is recorded when the patient is ready for | The third endpoint, Step 3, was the number of days from admission to the notice to the municipality that the patient was ready for discharge from hospital. | or Step 2) was significantly decreased (p=0.03) in the per-protocol gPCC group compared with the usual care group.<br><br>The length of stay in hospital and the time to the third notification (Step 3) to the patients' municipal home-care services and/or round-the-clock home nursing care services were significantly decreased: 6.77 days in the per-protocol gPCC group compared with 9.22 days in the usual care group (p<0.01), and 11 days in the per-protocol gPCC group |

| Study Number | Author & Year/ Country | Aim Design Theoretical model | Sample | Intervention(s) | Outcomes/measures and follow-up period | Results |
|---|---|---|---|---|---|---|
| | | | | discharge, also in concordance with the gPCC health plan projected number of days of hospitalization, which is Step 3. | | compared with 35 days in the usual care group (p=0.01), respectively |
| 27c | Ekman et al (2012) (39)<br><br>Sweden | To evaluate outcomes of PCC in hospitalized patients with chronic heart failure (CHF) with respect to the length of hospital stay (LOS), activities of daily living (ADL), health-related quality of life (HRQL) and 6-month readmission rate<br><br>Controlled before and after design<br><br>Gothenburg PCC | As above | As above | Primary outcome:<br>1. Length of stay (LOS) computed as number of whole inpatient days from admission to discharge<br><br>Secondary outcomes: | 1. The mean LOS in the Usual care group was 9.22 days (SD 7.4, median 7, IQR 5, range 2–44 days) compared with 8.22 days (SD 4.4, median 8, IQR 5, range 2–31 days) in the PCC group (P . 0.16). In the PP analysis, LOS was significantly shorter (2.5 days) in the PCC group (6.77 days, SD 3.2, median 6.5, IQR 3, range 2–25; P . 0.01),<br><br>2. Physical functional |

| Study Number | Author & Year/ Country | Aim Design Theoretical model | Sample | Intervention(s) | Outcomes/measures and follow-up period | Results |
|---|---|---|---|---|---|---|
| | | | | | 2. Activities of daily living (ADL) using the Katz-ADL index<br><br>3. Quality of life (HRQL) assessed using the Swedish version of the Kansas City Cardiomyopathy Questionnaire (KCCQ)<br><br>Data collected at baseline, three months, and six months. | performance as assessed with the Katz–ADL index was similar at baseline between the two groups in the analysis of all patients as well as in the PP analysis. At discharge, ADL levels were better in the PCC group (all patients, P . 0.07; the PP group, P . 0.04).<br><br>3. There were no differences in the KCCQ Overall Summary Score or the Clinical Summary score after 3 months. |

| Study Number | Author & Year/ Country | Aim Design Theoretical model | Sample | Intervention(s) | Outcomes/measures and follow-up period | Results |
|---|---|---|---|---|---|---|
| 27d | Dudas et al 2012 (40) | To evaluate whether PCC is associated with less self-reported uncertainty in illness compared with usual care in patients hospitalized for worsening chronic heart failure (CHF).<br><br>A controlled before and after design<br><br>Gothenburg PCC framework | As above | As above | The Swedish version of the Cardiovascular Population Scale (CPS) CPS consists of two dimensions:1) ambiguity (10 items), which covers the perception of patients concerning the severity of their illness; and 2) complexity (six items), which covers the perception of patients concerning their dignity, treatment and system of care. | The PCC group had better scores than the usual care group in the CPS domains complexity (M=15.2, SD=4.7 vs. M=16.8, SD=4.7; p=0.020) and ambiguity (M=27.8, SD=6.6 vs. M=29.8, SD=6.9; p=0.041).<br><br>The PCC group reported lower scores in the dimension of ambiguity, which measures patients' self-reported experiences about uncertainty in their illness, in both the ITT analysis and in the PP analysis (M = 28.2 (SD = 6.5) and 27.8 (SD = 6.6), respectively) than the usual care group (M |

| Study Number | Author & Year/ Country | Aim Design Theoretical model | Sample | Intervention(s) | Outcomes/measures and follow-up period | Results |
|---|---|---|---|---|---|---|
| | | | | | | = 29.8 (SD = 6.9)). There was a significant difference in the dimension of ambiguity in the PP analysis between the groups for patients in the PCC group (p = 0.067). |

| | | | | | | |
|---|---|---|---|---|---|---|
| 28 | Jutterström et al (2016) (41)<br><br>Sweden | To evaluate the effect of a nurse led patient-centered self-management support in T2D with regard to metabolic changes.<br><br>RCT<br><br>Theory of Hernandez | N=182 people aged 40-80 with T2DM<br>n=70 Group Intervention (GI)<br>n=35 Individual Intervention (II)<br>n=36 Internal control group<br><br>n=54 External Control | Ten Diabetes Specialists Nurses (DSNs) from nine health care centres participated in a preparatory workshop of approximately 20 hrs that emphasised the patients understanding of illness. DSNs received a theoretical and practical preparation and motivating patient-centred communication aimed at supporting illness integration and how to strengthen patient's self-efficacy for self-management.<br>In the patient intervention, participants in the GI and II groups were invited to six sessions of 45-90 minutes each over a period of up to six months.<br>In the GI groups, the patients reflected aspects of living with T2D together and DSNs acted as a moderator.<br>The intervention consisted of either discussions in groups or patients or individual conversations with the DSN, depending on the arm of allocation. During the six sessions, the | 1. HbA1c<br><br>2. Body mass index<br><br>3. Systolic and diastolic blood pressure | 1. HbA1c significantly decreased at 12 months follow-up by 5 mmol/mol in the GI (p<0.001) and 4 mmol/mol (p=0.004)in the individual intervention (II), in the internal control group there was no change (p=0.878), while in the external control group it increased with 2 mmol/mol (p=0.213). The results were significant between intervention groups (GI and II) and external control group.<br><br>2. Body mass index was not significant between groups<br><br>3. Both systolic and diastolic blood pressure were not significant between groups |

| | | | | | | |
|---|---|---|---|---|---|---|
| | | | | participants were free to discuss issues they considered important in relation to their experiences with the disease.<br><br>Control: IC and EC groups received standard care which normally included 1-2 visits per year as per national guidelines. | | |

| Study Number | Author & Year/<br><br>Country | Aim<br>Design<br>Theoretical model | Sample | Intervention(s) | Outcomes/measures and follow-up period | Results |
|---|---|---|---|---|---|---|
| 29a | Olsson et al 2016 (42)<br><br>Two papers one study | The study had two aims:<br>(1) to identify vulnerable patients using the general self-efficacy scale (GSES) and the Tampa scale for Kinesiophobia (TSK), and (2) to evaluate if person-centred care including the responses of the instruments made rehabilitation more effective in terms of shortening hospital length of stay.<br><br>A quasi-experimental design | Patients scheduled for total hip arthroplasty (THA), an intervention group (n = 128), mean age 68 and a control group (n = 138), mean age 66. | Intervention group received evidence-based information based on their own prerequisites. Evidence-based guidelines, clinical knowledge and patients' individual prerequisites were combined with forming a partnership with professionals.<br><br>The first step in establishing the partnership was for a RN specialized in surgical care to obtain a narrative from each patient, covering the patient's everyday life, resources, motivation, and goals; patients were also asked to fill out the General Self-efficacy (GSES) and Tampa scale of kinesiophobia (TSK) questionnaires.<br><br>The RN then made a tentative, detailed gPCC health plan based on the narrative, the medical examination, and the self- | The primary endpoint of the study was the number of days spent in the hospital relative to the self-rated GSES and TSK scores. The hospital Length of Stay was compared between the control group and the intervention group for patients scoring ≤ 29 on the GSES and/or ≥ 40 on the TSK. The relation between Length of Stay and American Society of Anesthesiologists" classification system (ASA) category was also studied.<br><br>1. Self-Efficacy: General self-efficacy scale (GSES)<br><br>2. Fear of Movement: Tampa Scale for Kinesiophobia (TSK)<br><br>3.Length of Stay | Significantly shorter stay in intervention group: 5.3 days (SD 2.2) vs control 7 days (SD 5.0); P<0.0005.<br><br>Patients with low GSES in the intervention group had shorter length of stay (LoS) by 1.6 days (95 % CI 0.16–3.15) p=0.03.<br><br>Patients with high TSK in the intervention group had shorter LoS by 2.43 days (95 % CI 0.76–4.12) p= 0.005. For patients who had both, the reduction of LoS was 2.15 days (95 % CI 0.24–4.04) p=0.028. |

| Study Number | Author & Year/ Country | Aim Design Theoretical model | Sample | Intervention(s) | Outcomes/measures and follow-up period | Results |
|---|---|---|---|---|---|---|
| | | | | reported results of the GSES and TSK surveys. The gPCC health plan specified each patient's short-and long-term goals, resources, special needs, and plan for recovery after discharge.<br>The tentative health care plan was included in the letter provided to the patient at the outpatient clinic appointment 2 weeks before surgery. The health plan was discussed with the patient and finalized when an agreement was reached between the professionals and the patient.<br><br>The patients were helped to familiarise themselves in the situation and to achieve their personal goal by emphasising their personal resources and capabilities documented in the health plan. | 4. American Society of Anesthesiologists" classification system (ASA): Patients scheduled for planned surgery commonly belong to one of three categories: (1) healthy, (2) mild systemic disease, or (3) severe systemic disease. The patients in this study were classified by the anaesthesiologist responsible for anaesthetising patients during the surgical procedure. | |

| **Study Number** | **Author & Year/<br><br>Country** | **Aim<br>Design<br>Theoretical model** | **Sample** | **Intervention(s)** | **Outcomes/measures and follow-up period** | **Results** |
|---|---|---|---|---|---|---|
| | | | | Control group received Standard care consisted of:<br><br>Completing questionnaires about their living circumstances, physical abilities and filled out surveys such as the GSES, TSK. Standardised information including peri-operative routines and postoperative training based on hip replacement patients in general. Patients also got a written booklet containing details from the oral information about pre and postoperative care. | | |
| 29b | Olsson et al 2014 (43) | To investigate if person-centred care intervention would improve patients' recovery as measured by Length of stay LoS following hip surgery | As above | As above | 1. The primary outcome measure was Length of Stay LoS, calculated as the number of whole inpatient days from admission to discharge.<br><br>2. Secondary outcomes included physical function | 1. The mean LoS in the control group was 7 days (SD 5.0) compared to 5.3 days in the gPCC group (SD 2.2) (p <0.0005)<br><br>2. Physical functional performance: At |

| Study Number | Author & Year/ Country | Aim Design Theoretical model | Sample | Intervention(s) | Outcomes/measures and follow-up period | Results |
|---|---|---|---|---|---|---|
| | | | | | at both discharge and 3 months later, measured with Activity of Daily Living (ADL) and Functional Recovery Scale (FRS). ADL was self-assessed by the patients at admission and measured by a nurse at discharge.<br><br>3. Readmission: Any hospital readmission within 3 months was obtained from the patient records. | discharge, 84% in the control group had regained ADL level A compared with 72% in the intervention group, the difference was not significant.<br><br>For FRS: Three months after surgery, 12% in the control group scored under 80% compared with 8.5% in the gPCC group and the difference was not significant.<br><br>3. Readmissions within 3 months were similar between the two groups; two patients in the control group and three in the gPCC group were readmitted and the |

| Study Number | Author & Year/<br><br>Country | Aim<br>Design<br>Theoretical model | Sample | Intervention(s) | Outcomes/measures and follow-up period | Results |
|---|---|---|---|---|---|---|
| | | | | | | difference was not significant. |
| 30 | Or and Tao (2016) Hong (44)<br><br>Hong Kong | Evaluate the effects of a person-centred tablet computer-based self-monitoring system for chronic disease (T2D and/or hypertension).<br><br>RCT | N=63 patients with T2D and/or hypertension<br>n=33 intervention, mean age 69.3 (SD 9.7)<br>n=30 control, mean age 69.7 (SD 10.2) | Tablet computer-based disease self-monitoring system. The system was interactive with 10 inch tablet computer, blood glucose and blood pressure monitor (2 in 1). The system would indicate Vital signs values. Abnormal values were measured in red, normal values in green. The system also had video-based educational materials that allowed patients to learn how to self-manage their chronic conditions, e.g. how to measure glucose, BP, diet, and exercises.<br><br>Comparison group received a 2-in-1 blood glucose and blood pressure monitor for self-monitoring and a logbook for recording the vital signs measured and the dates and times of measurements. | 1.Systolic and diastolic blood pressures | 1. Significant improvements were seen in systolic blood pressure in the intervention group from baseline to 1 month (-16.7 mm Hg), 2 months (-10.3 mm Hg) and 3 months (-13.0 mm Hg). Non-significant differences were seen in the control group (-2.1 mm Hg) at month one, 6.2 at 2 months, and -5.4 mm Hg at 3 months. The differences were significant between the two groups after 1 month (p<0.001) and month 3 (p=0.043). Similarly significant differences were seen in diastolic pressure in the |

| Study Number | Author & Year/ Country | Aim Design Theoretical model | Sample | Intervention(s) | Outcomes/measures and follow-up period | Results |
|---|---|---|---|---|---|---|
| | | | | | | intervention group (-8.0 mm Hg) at 1 month, -6.6 mm Hg at month 2, and -5.7 mm Hg at month 3. Non-significant decline were seen in the control group -0.3 mm Hg at 1 month, -1.9 mm Hg at 2 months, and -2.0 mm Hg at 3 months. The decline in diastolic pressure were significantly greater in the intervention group than control group after 1 ($p<0.001$) and 2 months ($p=0.028$). |
| | | | | | 2.Fasting blood glucose | 2. After 3 months non-significant decline in FBG was seen in the intervention group (-1.0 mmol/dL) and an increase in the control group (0.4 mmol/dL), the trend |

| **Study Number** | **Author & Year/<br><br>Country** | **Aim<br>Design<br>Theoretical model** | **Sample** | **Intervention(s)** | **Outcomes/measures and follow-up period** | **Results** |
|---|---|---|---|---|---|---|
| | | | | | 3. HbA1c<br><br>4. Patient's knowledge of T2D and hypertension: Modified Michigan Diabetes knowledge Scale and the hypertension knowledge questionnaire.<br>Measured at baseline, months 1,2, and 3. | was not statistically different between groups (p=0.407).<br><br>3. HbA1c<br>Both decreased at 3 months -0.2 in the intervention and control groups. No between group differences.<br><br>4. No significant differences on knowledge of hypertension and T2D. |
| 31a | Sahlen et al (2016) (45)<br><br>Sweden | To assess the cost-effectiveness of person-centred care integrated heart failure and palliative home care.<br><br>RCT | N=72 participants with NYHA class III-IV heart failure<br><br>n=36 intervention<br>n=36 control | Person-centred integrated intervention. Structured PCC (partnership between patients/carers and professional caregivers and includes initiating, working on and documenting partnership) with a collaborative approach | 1.Quality adjusted life years (QALYS) EQ-5D | 1.QALY was 0.569 in the intervention and 0.538 in the control group as baseline. Slight improvement was seen in the intervention (+0.006), but |

| Study Number | Author & Year/<br><br>Country | Aim<br>Design<br>Theoretical model | Sample | Intervention(s) | Outcomes/measures and follow-up period | Results |
|---|---|---|---|---|---|---|
| | | Person-centred palliative care model. Six S: self-image, self-determination, social relationships, symptom control, synthesis and surrender. | | between palliative and heart failure care specialists involving rounds with all team members every 2 weeks. Care delivered at home with easy access to care with frequency and duration of calls dependent on patient need. The team was responsible for total care including co-morbidities.<br><br>Comparison group received usual care consisting of nurse-led heart failure clinic at the hospital or primary health care centre. | 2. Costs of health care: multiplying the allocated time for given services by the average salaries.<br><br>Data collected at baseline, and month six. | declined in the control group (-0.024), p=0.026.<br><br>2. Cost of intervention SEK (Swedish krona) 1.4 million (140,000 Euros). The control costed SEK 2 million (205,000 euros). The intervention reduced costs of SEK 600,000 over the 6 month intervention period. |
| 31b | Brännstrom & Boman (2014) (46)<br><br>Sweden. | To evaluate the effect of a PCC and integrated palliative advanced home care and heart failure care.<br><br>RCT.<br><br>Person-centred palliative care model. Six S: self-image, self-determination, social | N=72 patients with CHF class III-IV.<br>n=36 intervention<br>n=36 control | Multi-disciplinary approach involving collaboration between specialists in palliative care and heart failure care (specialised nurses, palliative care nurses, cardiologists, palliative care physician, physiotherapists and occupational therapists. Patients also received structured PCC at home. | 1. Symptom burden: Edmond Symptom Assessment Scale (ESAS)<br><br>2. Health related QoL-Euro QoL (EQ-5D) | 1. ESAS was not significant between the groups (data not provided).<br><br>2. No significant differences in QoL between the two groups (47.7 to 60.4 in the intervention group and 48.2 to 52.3 in the control |

| Study Number | Author & Year/<br><br>Country | Aim<br>Design<br>Theoretical model | Sample | Intervention(s) | Outcomes/measures and follow-up period | Results |
|---|---|---|---|---|---|---|
| | | relationships, symptom control, synthesis and surrender. | | The model used the six S as Sahlen et al (2016) above<br><br>Control: usual care as described above (Sahlen et al; 2016). | 3. Kansas City Cardiomyopathy Questionnaire (KCCQ)<br><br>Assessments were conducted at baseline, 3 and 6 months. | group), P=0.10. Age-adjusted analysis between groups showed delta value of HRQL from baseline to 6 months was significantly better in the intervention compared to control (p=0.02).<br><br>3. No significant differences were found between the two groups (data not provided). |
| 32 | Slok et al. (2016) (47)<br><br>The Netherlands | To assess the effectiveness of the Assessment of Burden of COPD (ABC) toll on disease specific quality of life in patients with COPD<br><br>A Cluster RCT. | N=39 primary care practices, 17 hospitals<br>N=357 COPD patients<br>n=175 intervention, mean age 64.8 (SD 8.7) | Applied the ABC tool consisting of a short validated questionnaire assessing the experienced burden of COPD, parameters of COPD lung function, and treatment algorithm including visual display and treatment advice. | Primary outcomes:<br>1. Improvement in disease-specific quality of life at 18 months; St George's Respiratory Questionnaire (SGRQ) | 1. At 18-months 34% of the 146 patients from 27 health care providers in the intervention group had a clinically significant improvement in the SGRQ (at least 4 points) compared |

| Study Number | Author & Year/<br><br>Country | Aim<br>Design<br>Theoretical model | Sample | Intervention(s) | Outcomes/measures and follow-up period | Results |
|---|---|---|---|---|---|---|
| | | | n=182 control, mean age 65.8 (SD 8.8) | GPs, nurses, pulmonologists were instructed to use the ABC tool during their routine consultations. Patients visited health care professionals at least four times in 18 months. Patients were asked to fill out the ABC scale, report their dyspnoea using the MRC dyspnoea scale and self-report level of physical activity. Patients and providers could decide on treatment plan together. Patients formulated personal treatment goals.<br><br>Health care professionals in the control group provided usual care according to Dutch COPD guidelines. | Secondary outcomes:<br>2. Disease-specific quality of life; COPD Assessment Test (CAT)<br><br>3. Perceived QoL: Patient Assessment of Chronic Illness Care (PACIC)<br>Collected at four time points: baseline, 6 months, 12 months and 18 months. | with 22% of the 146 patients from the 29 healthcare providers in the control group (OR 1.85; p=0.02).<br><br>2. No significant differences in the CAT between the two groups (-0.26; p=0.68).<br><br>3. PACIC improved significantly in the intervention group compared with the control group at 18 months (0.32; p<0.01). |
| 33 | Windrum et al (2016) (48)<br><br>UK | To examine the relative impacts of alternative patient education programmes for people newly diagnosed with type 2 diabetes. | N=203 patients with Type 2 Diabetes from 6 General Practices in a city | Intervention: Patient centred education based on mediated learning. Delivered by health care professionals who attended a two-day course. Discussions were mediated | Fasting HbA1c at diagnosis and at 12 months after education programme in mmol/l. | 1. HbA1c significantly lower in IG than CG after 12 months (6.838 vs 7.163, p<0.05) |

| Study Number | Author & Year/<br><br>Country | Aim<br>Design<br>Theoretical model | Sample | Intervention(s) | Outcomes/measures and follow-up period | Results |
|---|---|---|---|---|---|---|
| | | RCT | n=94 intervention, mean age 65.8 (SD 9.69)<br>n=109 control, mean age 65.35 (SD 8.45) | between patients on key areas of health and self-management. Patients learnt to use and critically appraise information, translating it to their own individual circumstances. Patients received an 'education pack' with the same basic information as the control group and were encouraged to reflect on their own behaviour and health choices. Finally patients created a personal action plan with key goals for diet, exercise and lifestyle.<br><br>Control: Didactic course of diabetes education including causes of the condition, symptoms, diet and exercise and foot care. Patients also received NHS and Diabetes UK information leaflets. | | |
| 34 | Yu (2016) (49)<br><br>Hong Kong | To develop an innovative geriatric practice, a health and | N=60 family caregivers co-residing with frail | Intervention: A comprehensive health and social assessment of | 1. Caregiver perceived burden: Caregiver | 1. IG had significantly greater reduction in |

| Study Number | Author & Year/<br><br>Country | Aim<br>Design<br>Theoretical model | Sample | Intervention(s) | Outcomes/measures and follow-up period | Results |
|---|---|---|---|---|---|---|
| | | social collaborative case management (HSC-CM) for family caregivers of older adults and conduct a pilot RCT<br><br>Pilot RCT | older adults and providing 6 or more hours of care daily recruited from an elderly community centre run by the YWCA<br>n=30 carers in intervention group, mean age 61.5 (SD 15.5)<br>n=30 carers in control group, mean age 61.2 (SD 17.1) | caregiver and care recipient conducted in the first 4 weeks by two case managers, a registered nurse and a social worker. A case manager was assigned to provide integrated, coordinated continued care from week 5-16. Caregivers were invited to attend group workshops according to their needs to optimise informational, emotional and social support between peers.<br><br>Control: usual care. | burden inventory (CBI, Chinese version).<br><br>2. Caregiver and health-related quality of life: Medical Outcomes Study 36-item Short Form Health Survey (SF-36 Chinese version) | perceived burden (p=0.03) than CG<br><br>2. IG had significant improvement in vitality (p=0.049), social role functioning (p=0.47) and general well-being (p=0.49). |
| 35 | Hernandez et al, (2015) (50)<br><br>USA | Explore the effectiveness of a community-based integrated care (IC) service in preventing hospitalisations and emergency department visits in stable frail COPD patients<br><br>RCT | N=155 COPD patients.<br>n=71 intervention. Mean age 73 (SD=8) years.<br>n=84 control, mean age 75 (SD=9) years. | A 2-h educational programme administered by nurse covering disease knowledge, non-pharmacological treatments, techniques for pharmacological administration, and self-management of the disease and co-morbid conditions and strategies to adopt with future exacerbations. A joint | 1. Hospital admission and visit to emergency department<br><br>2. Mortality | 1. IC group showed decline in risk of emergency room visits; OR: 0.33 p=0.02. Hospital admissions did not differ significantly OR: 2.17; p=0.237<br><br>2. Mortality reduced in the IC group OR:0.36; p=0.034 |

| Study Number | Author & Year/ Country | Aim Design Theoretical model | Sample | Intervention(s) | Outcomes/measures and follow-up period | Results |
|---|---|---|---|---|---|---|
| | | | | visit of the specialist nurse and the primary care team (physician, nurse, social worker) at patient's home within 72 hours after study entry.<br>Community care team received 2 h face-to-face educational training and 1 day stay at the hospital ward, aiming at enhancing home-based management of frail COPD patients. Number of home visits individually tailored to patient needs.<br><br>Usual care: Comparison group received conventional treatment being managed by their physician without any support from specialised nurses. Visits were every 6 months in the out-patient clinic. | 3. Dyspnoea: MRC dyspnoea scale<br><br>4. Anxiety and depression: HADS<br><br>5. QoL: St George's Respiratory Questionnaire<br><br>6. COPD knowledge and self-management | 3. No difference between groups (p=0.96) at 12 months<br><br>4. No differences on anxiety between the groups (p=0.13), but depression significantly improved in the IC group (p<0.01) at 12 months<br><br>5. Symptoms score significantly reduced in the IC group compared with the control group 32 vs 42 p=0.02, activity and impacts scores did not change significantly 63 vs 69; p=0.20, 36 vs 40; p=0.28 respectively.<br><br>6. knowledge significantly increased in the IC |

| Study Number | Author & Year/<br><br>Country | Aim<br>Design<br>Theoretical model | Sample | Intervention(s) | Outcomes/measures and follow-up period | Results |
|---|---|---|---|---|---|---|
| | | | | | 7. Percentage of current smokers | group compared with the control group 40 vs 25; p=0.02<br><br>7. Lower percentage of current smokers in the intervention group (3% vs 16%, p=0.002. |
| 36 | Kikkenborg et al (51)(2015)<br><br>Denmark | To examine the potential effects of a short psychoeducational nursing intervention on primary emotions and describe the trajectory of primary emotions over time in patients with implantable cardioverter defibrillators (ICD).<br>RCT<br><br>Theory of nursing, Rosemary Rizzo Parses Human Becoming Practice | N=196 adults with first time ICD implantation<br>n=99 intervention group, mean age 58<br>n=97 control group, mean age 58 | Intervention: Three monthly, one hour nurse led psychosocial support and education sessions commencing on discharge.<br><br>Control: Usual care plus an invitation to attend a single 2 hour group session with information and sharing of experiences but no individual psycho-educational follow-up. | 1. Primary Emotions using The Emotions and Health Scale<br>Measured at baseline and 3 months | 1. No significant differences in primary emotions between intervention and control groups at 3 months.<br>Joy (11 vs 10.8, p=0.76),<br>Agreeableness (10.4 vs 10.2, p=0.64),<br>Surprise 77 vs 80, p=0.67, Fear 6.76 vs 6.94, p=0.42,<br>Sadness (8.15 vs 7.64, p=0.06)<br>Disgust (4.62 vs 4.96, p=0.83), Anger (5.68 vs 6.04, p=0.97, Anticipation |

| Study Number | Author & Year/<br><br>Country | Aim<br>Design<br>Theoretical model | Sample | Intervention(s) | Outcomes/measures and follow-up period | Results |
|---|---|---|---|---|---|---|
| | | | | | | 8.34 vs 8.83, p=0.35). |
| 37a | Larsson et al (2015) (52)<br><br>Sweden | To compare the costs of rheumatology care between a nurse-led rheumatology clinic (NLC) based on person-centred care (PCC), versus a rheumatologist-led clinic (RLC) in monitoring patients with chronic inflammatory arthritis (CIA) undergoing biological therapy.<br><br>RCT<br><br>Gothenburg PCC | N=97 patients with CIA undergoing biological therapy and a disease activity score (DAS28 </=3.2) recruited from a rheumatology clinic in Southern Sweden<br>n=47 intervention group, mean age 55.0 (SD 12.3)<br>n=50 control group, mean age 55.8 (SD 13.2) | Intervention: Patients randomised to attend a NLC based on the principles of patient centred care. In addition to assessing disease activity and medication, visits focussed on patients needs and global health. Patients could contact their nurse when needed between appointments.<br>Control: attending a Rheumatologist led clinic. Visits to both clinics lasted about 30 minutes. | Total annual use of resources and direct costs of care monitoring biological therapy over 12 months<br>Secondary outcome measures:<br>Annual use of resources and direct costs for the components of the primary outcome (fixed monitoring, variable monitoring, rehabilitation, specialist consultations, radiography and pharmacological therapy). | Statistically significant lower costs in IG than CG (€14107.7 vs €16274.9 per patient, p=0.004)<br><br>Statistically significant cost reductions in total fixed monitoring (-€116.7, p=0.001), total (fixed and variable) monitoring (-€155.0, p=0.001) and pharmacological therapy (-€1444.5, p=0.029). No statistically significant reduction in monitoring visits, blood tests, additional phone consultations, inpatient and outpatient rehabilitation, physiotherapy, |

| Study Number | Author & Year/<br><br>Country | Aim<br>Design<br>Theoretical model | Sample | Intervention(s) | Outcomes/measures and follow-up period | Results |
|---|---|---|---|---|---|---|
| | | | | | | occupational therapy, psychosocial treatment, specialist consultations or radiography. |
| 37b | Larsson et al (2013) (53)<br><br>Sweden | To compare and evaluate the treatment outcomes of a nurse-led rheumatology clinic and a rheumatologist clinic in patients with low disease activity or undergoing remission who are undergoing biological therapy<br><br>RCT<br><br>Gothenburg PCC | n= 107 patients with chronic inflammatory arthritis undergoing biological therapy and a disease activity score (DAS28 </=3.2) recruited from a rheumatology clinic in Southern Sweden<br>n=53 intervention, mean age 55 (SD 12.3)<br>n=54 control, mean age 55.8 (SD 13.2) | Intervention: Patients randomised to attend a NLC based on the principles of patient centred care. In addition to assessing disease activity and medication, visits focussed on patients needs and global health. Patients could contact their nurse when needed between appointments.<br><br>Control: attending a Rheumatologist led clinic. Visits to both clinics lasted about 30 minutes | Primary outcome:<br><br>1. Disease activity: DAS28 and DAS28-CRP<br><br>Secondary outcomes:<br>2. Performing Activities of Daily Living (ADLs): Health Assessment Questionnaire (HAQ)<br><br>3. Pain assessed by Visual Analogue Scale<br><br>4. Satisfaction in obtaining rheumatology | Mean difference of change (IG-CG) between groups not statistically significant for any primary or secondary outcome<br>1. DAS28 (-0.06, p=0.66) or DAS28-CRP (0.05, p=0.70)<br><br>2. 0.02, p=0.79<br><br>3. Non-significant -0.24, p=0.95<br><br>4. Non-significant 0.25, p=0.43 |

| **Study Number** | **Author & Year/<br><br>Country** | **Aim<br>Design<br>Theoretical model** | **Sample** | **Intervention(s)** | **Outcomes/measures and follow-up period** | **Results** |
|---|---|---|---|---|---|---|
| | | | | | care: Numerical Rating Scale<br><br>5. Confidence in obtaining rheumatology care: Numerical Rating Scale | 5. Non-significant 0.2, p=0.42 |
| 38 | Lowther et al (2015) (54)<br><br>Kenya | To evaluate the effectiveness of a nurse-led palliative care intervention among people with HIV<br><br>RCT | N=120 participants with HIV<br><br>n=60 intervention, mean age 38.3 (SD 8.2)<br>n=60 control, mean age 40.5 (SD9.2) | Patients in the intervention arm<br>received clinical care from a nurse who has received two weeks' training in palliative care and ongoing clinical<br>support and supervision from experienced palliative care providers.<br><br>Control group received care from nurse's who had no exposure to palliative care training. | Primary Outcome:<br>1.Pain severity: African Palliative Care Outcomes (APOS)<br><br>Secondary Outcomes:<br>2.Psychiatric morbidity: GHQ-12<br><br>3. Quality of Life (mental and physical: Medical Outcomes Study (MOS)-HIV | 1.Mean change was +3.5 in the intervention and +4.0 in the control (p=0.83)<br>Total APOS mean change was +12 in the intervention and +7.5 in the control (p=0.04).<br><br>2. Significant difference was seen between intervention and control (-0.50; p=0.04).<br><br>3. Significant differences between groups on mental health subscale |

| Study Number | Author & Year/ Country | Aim Design Theoretical model | Sample | Intervention(s) | Outcomes/measures and follow-up period | Results |
|---|---|---|---|---|---|---|
| | | | | | Outcomes assessed at baseline, one, two, three and four months. | (0.61; p=0.01) but no significant differences between groups on physical aspects of QoL(0.44; p=0.06). |
| 39 | Kelechi et al. (2014) (55)<br><br>USA | To test the feasibility and efficacy of a motivational enhancement and conditioning activity for leg function (MECALF) in patients with critically colonized/infected chronic leg ulcers.<br><br>Comparative study Motivational Enhancement | N=21 patients with critically colonised or infected leg or foot ulcers.<br>n=12 intervention<br>n= 9 control | Intervention: MECALF. Specialist nurses received 8 hours of training in motivational enhancement (ME). They used 10 minutes of each weekly wound visit to engage in ME over 6 weeks. Patients were given a brochure detailing an exercise programme (CALF) to promote walking and other physical activities developed by a physical therapist.<br><br>Control: CALF. Usual wound care as per protocols. Patients received the CALF exercise brochure but no ME. | Data collected at baseline and week 8 (2 weeks post intervention)<br>1. Pain : Leg Pain Questionnaire (LPQ)<br><br>2. Strength: dyanometer for ankle dorsiflexion and plantar flexion in $lb/in^2$<br><br>3. Ankle range of motion: goniometry for dorsiflexion, plantar flexion, inversion and eversion in degrees<br><br>4. Motivation: readiness ruler | 1. Reduced pain at 8 weeks in CG compared to IG (p=0.046)<br><br>2. No statistically significant difference between groups.<br><br>3. No statistically significant difference between groups at 8 weeks (p=0.748)<br><br>4. No statistically significant difference between groups (p=0.641) |

| Study Number | Author & Year/<br><br>Country | Aim<br>Design<br>Theoretical model | Sample | Intervention(s) | Outcomes/measures and follow-up period | Results |
|---|---|---|---|---|---|---|
| | | | | | 5. Self-efficacy/confidence: Questionnaire for Physical Activity and Exercise<br><br>6. Functional physical activity: Timed chair rise test, timed up and go, community healthy activities model for program for seniors (CHAMPS). | 5. No statistically significant difference between groups (p=0.643)<br><br>6. No statistically significant difference between groups in any measure. |
| 40 | Young et al (2013) (56)<br><br>Australia | To investigate the effectiveness of a centralised, nurse-delivered telephone based service to improve care coordination and patient reported outcomes after surgery for colorectal cancer.<br><br>RCT | N= 756<br>n=387 intervention group, mean age 86.9 (SD 12.2)<br>n=369 control group, mean age 67 (SD 12.1) | Five scheduled, structured telephone calls from a nurse on days 3 and 10 then at 1,3 and 6 months after hospital discharge. Identified needs were addressed by the nurse using detailed standardized clinical protocols.<br><br>Control group received usual care. | Primary and secondary outcomes not specified.<br>1. Total care coordination score at 3 and 6 months<br><br>2. Global assessment of care coordination at 3 and 6 months | 1. No significant differences between intervention and control groups at 3 (79.5 vs 78.7, p=0.3) or 6 months (80 vs 80.3, p=0.8).<br><br>2. No significant differences between intervention and control groups median scores at 3 (9 vs 9, p=1.0) or 6 months (10 vs 10, p=0.1). |

| Study Number | Author & Year/<br><br>Country | Aim<br>Design<br>Theoretical model | Sample | Intervention(s) | Outcomes/measures and follow-up period | Results |
|---|---|---|---|---|---|---|
| | | | | | 3. Global assessment of quality of care at 3 and 6 months | 3. No difference in intervention and control groups median scores at 3 (10 vs 10, p=1.0) or 6 months (10 vs 10, p=1.0) |
| | | | | | 4. Supportive Care Needs Survey Short Form (SCNS-SF34) at 3 and 6 months | 4. No difference in intervention and control group unmet needs median score at 3 (59.9 vs 56.8, p=0.6) or 6 months (50.0 vs 46.6, p=0.7) |
| | | | | | 5. Unplanned readmissions at 1 and 6 months | 5. No difference between intervention and control group in unplanned admissions at 1 (8.6 vs 10.5%, p=0.4) or 6 months (25.6 vs 27.9%, p=0.5) |
| | | | | | 6. Emergency room presentations at 1 and 6 months | 6. No difference between intervention and control group in emergency room presentations at 1 (10.8 vs 13.8%, |

| Study Number | Author & Year/ Country | Aim Design Theoretical model | Sample | Intervention(s) | Outcomes/measures and follow-up period | Results |
|---|---|---|---|---|---|---|
| | | | | | | p=0.2) or 6 months (25.9 vs 25.4%, p=0.9) |
| | | | | | 7. Proportion receiving postoperative chemotherapy | 7. No significant difference between intervention and control groups in proportion receiving postoperative chemotherapy (73 vs 78%, p=0.5) |
| | | | | | 8. Distress at baseline, 1, 3 and 6 months | 8. No difference in intervention and control groups in mean distress scores at 1 (2.3 vs 2.4, p=0.1), 3 (2.0 vs 2.0, p=0.3) or 6 months (1.8 vs 1.8, p=0.2) |
| | | | | | 9. Functional Assessment of Cancer Therapy- Colorectal (FACT-C) total score at baseline, 1, 3 and 6 months | 9. No significant difference between intervention and control groups in FACT-C total score at 1 (100.61 vs 100.40, p=0.4, 3 (103.48 vs103.26, |

| Study Number | Author & Year/<br><br>Country | Aim<br>Design<br>Theoretical model | Sample | Intervention(s) | Outcomes/measures and follow-up period | Results |
|---|---|---|---|---|---|---|
| | | | | | | p=0.4) or 6 months (105.10 vs 105.35, p=0.5) |
| 41 | Chochinov (2011) (57)<br><br>USA, Canada and Australia | To determine if dignity therapy could mitigate stress and/or bolster end-of-life experience for patients nearing death<br><br>Multi centre RCT | N=326 patients receiving hospital or community based palliative care<br>n=108 dignity therapy, mean age 64.2 (SD 14.6)<br>n=107 client centred care, mean age 64.3 (SD 14.3)<br>n=111 standard palliative care, mean age 66.7 (SD 14.2) | Dignity Therapy: novel brief (30 min) psychotherapy session providing an opportunity to speak about things that matter most to the patient often relating to meaning and purpose. Sessions were transcribed to produce a document that could be bequeathed to a recipient of patient's choice. Therapists undertook 3 day training.<br>Client Centred Care: Supportive psychotherapeutic approach focussing on 'here and now' issues such as symptoms and their illness. No permanent record of conversation given to patient.<br>Standard Palliative Care: access to MDT palliative care support services. | Primary outcomes:<br>1. Mean change in baseline and end of intervention<br><br>2. Palliative Performance Scale<br><br>3. FACIT spiritual well-being scale<br><br>4. Patient dignitary inventory (PDI)<br><br>5. Hospital anxiety and depression scale (HADS)<br><br>6. Items from Structured Interview for Symptoms and Concerns (SISC) including dignity, desire for death, suffering, hopelessness, depression, suicidal ideation and sense of burden to others. | Primary outcomes:<br>1-7. No significant differences found in change from baseline to end of intervention between the three groups in any outcome measure. |

| **Study Number** | **Author & Year/ Country** | **Aim Design Theoretical model** | **Sample** | **Intervention(s)** | **Outcomes/measures and follow-up period** | **Results** |
|---|---|---|---|---|---|---|
| | | | | Control group: Participants assigned to the control group received Standard Palliative Care which included access to the full range of palliative care support services available to all study patients, including specialist palliative care physicians and nurses (i.e. experts in pain and symptom management), social workers, chaplains, and psychologists and/or psychiatrists. No participating site provided a formal approach to addressing generativity issues; as such, a program comparable to Dignity Therapy was not available to patients who were not randomized to the Dignity Therapy arm of this trial. | 7. Two item quality of life scale<br><br>Secondary outcome:<br>8. Detailed survey of experience of study | 8. Dignity therapy group more likely to have found the study helpful (p<0.001), that it improved their quality of life (p<0.001), sense of dignity (p=0.002), spiritual wellbeing (p=0.006), lessened sadness or depression (p=0.009) and felt satisfied with the study arm assignment (p<0.001). The Dignity Therapy group were likely to report that being in the study changed how their family appreciate and see them (p<0.001) and |

| Study Number | Author & Year/<br><br>Country | Aim<br>Design<br>Theoretical model | Sample | Intervention(s) | Outcomes/measures and follow-up period | Results |
|---|---|---|---|---|---|---|
| | | | | | | that it will help their family p<0.001). |
| 42 | Goelz et al (2011) (58)<br><br>Germany | To demonstrate that COM-ON-p concise and individualized communication skills training (CST) improves oncologists communication skills in consultations focussing on the transition to palliative care<br><br>RCT | N=41 physicians in charge of patients with cancer and practising at a University Medical Centre in Germany<br>n=22 physicians in intervention group<br>n=19 physicians in control group | Intervention: Participants undertook the COM-ON-p training programme including pre-assessment with an actor patient (1 hour), a 1.5 day workshop and an individual coaching workshop (30 mins) 2 weeks after the workshop and post assessment with an actor patient (1 hour). Facilitators were experienced in oncology and CST and helped physicians focus on individual learning goals which they had developed with video analysis.<br><br>Control: No additional training.<br>All physicians undertook 2 video recorded consultations with actor patients at baseline and 5 weeks later. | COM-ON-Checklist: Participants were ranked on 5 point scale for relevant behavioural domains.<br>Primary outcome:<br><br>1. Section A average score for 6 items specific to the transition to palliative care<br><br>2.Section B average score for 9 general communication items<br><br>Secondary outcome:<br>3. Involving significant others: Section C average score of 4 items on the involvement of significant others and global item 2. | 1. IG had significantly higher scores than CG after intervention (Effect size 0.78, p=0.0026)<br><br>2. IG had significantly higher scores than CG after intervention (Effect size 0.78, p=0.0078.<br><br>3. IG had significantly higher scores than CG after intervention (Effect size 0.65, p=0.0070). |

| Study Number | Author & Year/<br><br>Country | Aim<br>Design<br>Theoretical model | Sample | Intervention(s) | Outcomes/measures and follow-up period | Results |
|---|---|---|---|---|---|---|
| 43 | Murphy et al (2010) (59)<br><br>USA | To examine whether tailored activity pacing intervention was more effective than general activity pacing intervention for managing pain and fatigue in adults with osteoarthritis.<br><br>RCT | n=13 intervention group with OA, mean age 63.9 (SD=7.8)<br>n=11 control group with OA, mean age 59.5 (SD= 6,6) | Intervention: Education module on activity pacing tailored to the individual delivered by an occupational therapist. Participants undertook 5 days of home monitoring of activity levels with an accelerometer and a log of symptoms and activity. A personalised report detailing the relationship between activity and symptoms was the basis for pacing recommendations. Second session focussing on individual progress.<br><br>Control: Education module on generalised activity pacing delivered by an occupational therapist with advice to implement the strategies. Second session focussing on individual progress. | Primary outcomes:<br>1. Pain: WOMAC<br><br>2. Fatigue: Brief Fatigue Inventory<br>Data collected at baseline and 10 week follow up | 1. WOMAC pain score decreased from baseline to week 10 in the control group (9.4 to 7.6) and the intervention group (7.9 to 6.7). The difference between groups was not statistically significant (p=0.35) with small effect size d=0.38.<br><br>2. BFI Fatigue Severity reduced in the control group (4.3 to 4.8) and the intervention group (4.1 to 3.3). The difference between groups was not statistically significant (p=0.09) with a moderate to large effect size (d=0.79) BFI Fatigue Interference increased in the control group (3.6 to |

| **Study Number** | **Author & Year/<br><br>Country** | **Aim<br>Design<br>Theoretical model** | **Sample** | **Intervention(s)** | **Outcomes/measures and follow-up period** | **Results** |
|---|---|---|---|---|---|---|
| | | | | | | 4.2) and decreased in the intervention group (3.1 to 1.6). The difference between groups was statistically significant (p=0.02) with a large effect size (d=1.10) |
| 44 | Wolff et al (2010) (60)<br><br>USA | Determine whether guided care (GC) improves patients' primary caregivers' depressive symptoms, strain, productivity and perceptions of quality of care for care recipients.<br><br>Clustered RCT | N=308 primary caregivers/patient dyads<br>n= 156 intervention caregivers (mean age 60.9 years)/patient (mean age 78.0 years) dyads randomised to Guided Care (GC)<br>n=152 usual care caregiver (mean age 61.6)/patient (mean age 77.9) dyads (UC)<br>n=22 usual care, mean age 31.91 (SD=6.52), male | Guided Care (GC)provided by nurses: included training and supporting patient's family caregivers. Designed to address deficiencies in the quality of chronic care delivery by facilitating coordinated, comprehensive, evidence-based heath care for multimorbid adults.<br><br>GC nurses collaborated with patients PCP to provide clinical processes: assessing the patient at home, creating an evidence-based care plan, promoting patient self-management, proactively monitoring patient condition, | Primary outcomes:<br><br>1. Caregiver depressive symptoms: Centre for Epidemiological Studies (CES-D)<br><br>2. Caregiver strain: Modified Caregiver Strain Index (CSI) | At 18 months follow-up:<br><br>1. CES-D changed from 6.4 to 6.8 in the GC compared with 7.1 to 5.8 in the UC. The results were not statistically significant between groups<br><br>2. CSI increased from 6.5 to 6.7 in the GC group and 6.6 to 7.7 in the UC group. These results were not statistically significant between the two groups. |

| Study Number | Author & Year/<br><br>Country | Aim<br>Design<br>Theoretical model | Sample | Intervention(s) | Outcomes/measures and follow-up period | Results |
|---|---|---|---|---|---|---|
| | | | gender n=21 (95.5%).<br>Participants recruited within 14 primary care physician teams (PCP) | coaching the patient to practice healthy behaviours, coordinating patients transition between sites and providers of care, facilitating access to community resources, and educating and supporting patients family caregivers.<br><br>Comparison group received usual care (details not provided). | 3. Quality of Chronic Illness Care: modified version of the Patient Assessment of Chronic Illness Care (PACIC)<br><br>4.Caregiver Productivity Loss: Work Productivity and Activity Impairment questionnaire (WPAI:CG)<br><br>Baseline and 18-month follow-ups. | 3.Aggregate QoL was higher in the GC group compared with the usual care group (0.40; p<0.001)<br><br>4. Work productivity loss was more substantial in the GC group compared with the UC group (14.6% to 8.4% vs 18.2% to 16.1%). Presentism declined from 16.7% to 11.9% in the UC group compared with 12.9% to 5.3% in the GC group. |
| 45 | Dobscha et al (2009) (61)<br><br>USA | To assess whether a collaborative intervention can improve chronic pain-related outcomes in a Department of Veteran Affairs (VA) primary care setting.<br><br>Cluster RCT | N=401 patients at 5 primary care clinics with moderate or severe chronic pain<br>n=187 intervention group, mean age 62.1 (SD 11.2) | Intervention: clinicians in intervention practices undertook two 90 minute workshops including abbreviated training in shared decision making skills and chronic pain education. Patients received an assessment with a care manager to | Primary Outcome:<br>1. Self-reported pain disability: Roland Morris Disability Questionnaire for pain (RMDQ) score<br>Additional main outcomes:<br><br>2. Depression severity: PHQ-9 | 1. Greater improvement from baseline to 12 months in intervention group than control (-1.4 vs -0.2, p=0.004).<br><br>2. Greater improvement from |

| Study Number | Author & Year/<br><br>Country | Aim<br>Design<br>Theoretical model | Sample | Intervention(s) | Outcomes/measures and follow-up period | Results |
|---|---|---|---|---|---|---|
| | | | n= 214 control group, mean age 61.3 (SD 12.3) | develop individualised functional goals and a treatment plan was communicated to the clinician. Patients were invited to a four session workshop based on the brief activating approach. Care managers contacted patients every 2 months for 12 months to provide support and reassess goals and activities.<br><br>Control: treatment as usual including referral to speciality pain clinic, ancillary services such as physiotherapy and occupational therapy. | 3. Pain intensity: CPG Pain Intensity subscale<br><br>Secondary outcomes:<br>4. CPG Pain interference subscale<br><br>5. Patient rated global impression of change<br><br>6. Global VA health care satisfaction | baseline to 12 months in IG than CG (-3.7 vs -1.2, p=0.003).<br><br>3. Greater improvement from baseline to 12 months in IG than CG (-4.7 vs -0.6, p=0.01).<br><br>Secondary outcomes:<br>4. Improvement from baseline to 12 months in IG and worsening in CG (-5.7 vs 2.3, p=0.03)<br><br>5. Greater improvement in IG than CG at 12 months (3.7 vs 4.4, p<0.01)<br><br>6. No difference in change from baseline to 12 months in IG and |

| Study Number | Author & Year/<br><br>Country | Aim<br>Design<br>Theoretical model | Sample | Intervention(s) | Outcomes/measures and follow-up period | Results |
|---|---|---|---|---|---|---|
| | | | | | <br><br>7. Health related quality of life: EQ-5D<br><br>8. Effectiveness of VA chronic pain treatment Outcomes collected at baseline, 3, 6 and 12 months. | CG (-0.27 vs -0.36, p=0.44)<br><br>7. No difference between IG and CG in change from baseline to 12 months (-0.02 vs -0.04, p=0.17)<br><br>8. No difference in change from baseline to 12 months in IG and CG (0.33 vs 0.2, p=0.64) |
| 46 | Machado et al, (2007) (62)<br><br>Brazil | To compare effectiveness of psychotherapy based on client-centred therapy and exercise for patients with chronic nonspecific low back pain<br><br>RCT. | N=33 participants with nonspecific low back pain (LBP)<br>n=16 intervention, mean age 44.6 (SD=12.1) years.<br>n=17 control, mean age 42.4 (SD=13.2) years. | Psychotherapy based on the principles of nondirective counselling. Patients in groups attended 80 minute treatment sessions twice a week for 9 weeks. Therapists provided support as patients discussed life stressors, including chronic pain.<br><br>Control group received Physiotherapists-led exercise therapy. General | 1. Disability: Brazil Roland-Morris Questionnaire (BRM)<br><br>2. Pain: Visual Analogue Scale (VAS) | 1. Exercise group showed lower disability at 9 weeks compared with the psychotherapy group (-4.9 points difference; p=0.02), at 6 months (4 points difference; p=0.13)<br><br>2. Pain scores were not significantly lower in the exercise |

| Study Number | Author & Year/<br><br>Country | Aim<br>Design<br>Theoretical model | Sample | Intervention(s) | Outcomes/measures and follow-up period | Results |
|---|---|---|---|---|---|---|
| | | | | exercise consisting of 20 minute walking, general stretching, and strengthening of the bridge (lying supine with knees flexed, raising hips and hold for 5 seconds, repeating the procedure for 15 minutes). Patients attended the 40 minute sessions in groups, twice a week for 9 weeks. | 3. Depressive symptoms: Beck Depression Inventory (BDI)<br><br>Assessments conducted at baseline, 9 weeks and 6 months (depression was not assessed at 6 months). | group compared with psychotherapy group at nine weeks (-1.8; p=0.27)<br>At six months the exercise group again scored lower compared with the psychotherapy group (-1.3; p=0.38).<br><br>3. Exercise group showed less depressive symptoms compared with the psychotherapy group at nine week (-6.3 points difference; p=0.29). |
| 47 | Glasgow et al (2005) (63)<br><br>USA | To determine if an interactive computer technology intervention designed to improve patient centred communication | N=886 adults with Type 2 Diabetes under the care of 52 primary care physicians<br>n=469 intervention | Intervention: Before two appointments, 6 months apart, patients completed computerized touch screen assessments including recall of clinical interventions and | Primary outcome:<br>1. Patient reports of receiving American Diabetes Association recommended laboratory screenings and | Primary outcome:<br>1. intervention group had greater improvement in laboratory screenings completed than |

| Study Number | Author & Year/<br><br>Country | Aim<br>Design<br>Theoretical model | Sample | Intervention(s) | Outcomes/measures and follow-up period | Results |
|---|---|---|---|---|---|---|
| | | improves diabetes care.<br><br>Cluster RCT | group, mean age 62 (SD 1.4)<br>n=417 control group, mean age 64 (SD 1.3) | developing a self-management action plan. Received detailed personalised printout of results. Patients met a Care manager trained in patient centred self-management approaches to review care needs and self-care goals followed by a follow-up call after each visit.<br><br>Control: Completed the same touch screen computer assessment but received a print- out of general health risks. No meetings or calls from care manager but same number of physician appointments. | recommended patient centred care activities Secondary outcomes.<br><br>2. Diabetes quality of life (The revised Problem Area in Diabetes 2 Scale, PAID-2)<br><br>3. HbA1c<br><br>4. Total cholesterol to HDL cholesterol ratio.<br><br>5. Depression (Patient Health Questionnaire, PHQ-9, % with 10 or higher).<br><br>Outcomes measured at baseline and 12 months. | controls (F=11.6, p<0.001) and patient centred activities (F=39.5, p<0.001).<br><br>2. No significant difference between intervention and control groups at 12 months (27.4 VS 27.5, p=0.964).<br><br>3. No difference in HbA1c between intervention and control groups (7.11 vs 7.17%, p=0.571.<br><br>4. No difference between intervention and control groups (4.11 vs 4.15, p=0.733).<br><br>5. No difference between intervention and control groups (12.3 vs 13.9%). |

| Study Number | Author & Year/<br><br>Country | Aim<br>Design<br>Theoretical model | Sample | Intervention(s) | Outcomes/measures and follow-up period | Results |
|---|---|---|---|---|---|---|
| | | | | | | |
| 48 | Mills et al (2003) (64)<br><br>Australia | Geographically controlled study | N=509 people with Type 2 Diabetes in rural Australia<br>n=398 intervention<br>n=111 control | Intervention: Care planning using a patient centred care planning model. Emotions, thoughts and behaviours translated into patient specific problem statements then goals. Care plans created and reviewed annually. Relevant health services were scheduled in line with best practice. Patients were followed for two years at minimum 6 month intervals.<br><br>Control: usual care in rural Southern Australia | 1.Problem and goal scores recorded on linear analogue scale recorded by patients and service co-ordinators<br><br>2.Work and social adjustment: Work and Social Adjustment Scale (WASAS) at each visit.<br><br>3.Medical Outcomes Study 36-Short Form (SF36).<br><br>4.Emergency and elective admission rates | 1.Up to 60% of IG felt their main problem improved by the end of the trial. 40-60% of patients made some progress toward achieving their first goal.<br><br>2. The WASAS scores between the two groups were statistically significant (P < 0.01) over time, with mean scores improving 10%.<br><br>3.Statistically significant difference (p<0.01) between IG and CG in SF 36.<br><br>4. IG group hospital admission rate fell 18.2% compared to CG. |

| Study Number | Author & Year/<br><br>Country | Aim<br>Design<br>Theoretical model | Sample | Intervention(s) | Outcomes/measures and follow-up period | Results |
|---|---|---|---|---|---|---|
| 49 | Kennedy, et al (2003) (65)<br><br>UK | To evaluate the effects of a PC intervention on clinical outcomes and health service use among patients with inflammatory bowel disease (IBD).<br><br>Multicentre cluster RCT. | N=19 hospitals, outpatient (n=9 treatment, n=10 control).<br>n=635 patients with inflammatory bowel disease (IBD)<br>n=270 intervention (mean age 44.4, sd=14.9)<br>n=365 control (mean age 46.3, sd 15.1) | Clinicians at the intervention sites received a 2-hr training session led by an expert in postgraduate medical education using role play and video feedback titled 'patient-centred consultation in gastroenterology'. Training focused in PC medicine principles and applied to self-management in IBD. Patients at the intervention sites participated in PC consultations conducted by clinicians. A self-management plan was negotiated and written into the guidebook. Patients were instructed to call a specified number if they needed to schedule an appointment according to circumstances listed in the guidebook.<br><br>Patients at the control sites received management processes deemed appropriate by hospital specialists. | 1. Hospital appointments<br><br>2. Quality of life: Inflammatory bowel disease questionnaire (IBDQ)<br><br>3. Anxiety and depression: Hospital Anxiety and Depression Scale (HADS) | 1. The number of kept appointments reduced by app. one third in the intervention group compared with the control group (difference -1.4; p<0.001). The mean number of clinic non-attendances per person during the trial was also lower for the intervention group (difference -0.08; p=0.034).<br><br>2. IBDQ did not differ significantly between the two groups (difference 1.94; p=0.45)<br><br>3. HADS did not differ significantly between two groups (difference -0.35; p=0.40) |

| Study Number | Author & Year/<br><br>Country | Aim<br>Design<br>Theoretical model | Sample | Intervention(s) | Outcomes/measures and follow-up period | Results |
|---|---|---|---|---|---|---|
| | | | | | 4. Patient enablement: patient enablement instrument (PEI)<br><br>5. Satisfaction : Consultation satisfaction questionnaire (CSQ). | 4. the intervention group showed a higher enablement score (difference 0.90; p=0.026)<br><br>5. satisfaction did not differ significantly between the two groups (3.47; p=0.09). |
| 50 | Martin et al, (2004) (66)<br><br>New Zealand | To test whether individualised care plan for patients experiencing acute exacerbations of COPD result in reduced health care utilisation and improved quality of life<br><br>RCT. | N=93 COPD patients<br>n=44 intervention group, mean age 71.1 years.<br>n=49 control group, mean age 61.9 years. | Individualised care plan based on an interview between patient and respiratory nurse, review of hospital records by respiratory specialist and by patient's own GP. Each patient was given instructions about how to use the plan by the respiratory nurse. Copies of the plan were held by patient, GP, ambulance service, emergency department and after hour's surgery.<br><br>Control group received usual care. They did not | Primary outcome:<br>1.Utilisation of primary care services and hospital admissions | 1. Intervention group called out the ambulance service more frequent (2.8 vs 1.1) calls per 12 months (p=0.03).<br>Intervention group had more GP visits compared with control group (15.6 vs 11.6) in 12 months; p=0.08<br>The intervention group has more hospital admissions compared with the control group (1.1 vs 0.7); p=0.17. |

| **Study Number** | **Author & Year/ Country** | **Aim Design Theoretical model** | **Sample** | **Intervention(s)** | **Outcomes/measures and follow-up period** | **Results** |
|---|---|---|---|---|---|---|
| | | | | have an individualised care plan.<br>All participants remained under the care of their own GP. | 2. Quality of Life: St George's Respiratory Questionnaire (SGRQ)<br>Outcomes assessed at baseline, three, six and 12 months. | 2. SGRQ did not differ significantly between groups (57.3,sd=13.5 for intervention and 55.1, sd=14.6) for control. |
| 51 | Alamo, et al, (2002) (67)<br><br>Spain | To assess whether patient-centred consultations are more effective than usual care style of consultations among patient with chronic musculoskeletal pain and fibromyalgia<br><br>Clustered RCT | N=20 GP's in 13 health centres.<br>N=110 patients n=10 GP's intervention, n=10 GP's control.<br>N=63 (mean age 39.2; sd=7.6 years) patients intervention<br>N=47 (mean age 42.3; sd=10) patients control | GP's in the intervention received training on communication skills necessary to undertake PC approach. These focused on active listening, asking patients' to express their fears and concerns, offering reassurance, coming up with a management plan together with the patient.<br><br>Control group GP's provided usual care | 1. Pain intensity: VAS and pain scale of the Nottingham health profile (NHP) questionnaire<br><br>2. Number of tender points and subjective health status: NHP questionnaire | Pain reduced in the intervention group (mean pain at baseline 3.4 (sd=1.2), at 6 months 3.3 (sd=1.0) and at 12 months 3.1 (sd=1.0). Mean pain in the control group was 4.1 (sd=0.8), at 6 months 3.9 (sd=0.8) and at 12 months 3.9 (sd=0.8). The difference between the two groups was not statistically significant (p=0.73)<br><br>2.Number of tender points reduced significantly in the intervention group |

| Study Number | Author & Year/<br><br>Country | Aim<br>Design<br>Theoretical model | Sample | Intervention(s) | Outcomes/measures and follow-up period | Results |
|---|---|---|---|---|---|---|
| | | | | | 3. Psychological disturbance: Goldberg Scale of anxiety and depression (GHQ)<br><br>Participants were followed-up at 6 and 12 months. | compared with the control group (p=0.05)<br><br>3.GHQ anxiety significantly reduced in the intervention compared with the control group (p=0.04)<br>GHQ depression was not statistically significant (p=0.33) |
| 52 | Sommers et al. (2000) (68)<br><br>USA | To examine the impact of an interdisciplinary, collaborative practice intervention involving a primary care physician, a nurse, and a social worker for community-dwelling seniors with chronic illnesses<br><br>Concurrent, controlled cohort study | N=543 patients aged 65 or older under treatment for at least 2 chronic conditions. Recruited from 18 private primary care physician offices<br>n=280 intervention group, mean age 78 (SD 6.8) | Intervention: home assessment from a nurse or social worker including listening to health concerns, home safety check and functional assessment. Creation of risk reduction plans and treatment plans based on chronic disease self-management strategies. Follow up sessions at least every 6 weeks including telephone, home visit, small group sessions or office or hospital visit. | Utilisation of medical services at baseline, 1 and 2 years<br>1. Change in number of hospital admissions per patient per year<br><br>2. Change in percentage of patients with 1 or more hospital readmissions within 60 days<br><br>3. Change mean number of visits to all physicians | 1. Statistically significant reduction in admissions in IG vs CG (-0.02 vs 0.18, p=0.03)<br><br>2. Statistically significant reduction in readmissions in IG vs CG (-2.0 vs 5.4, p=0.03)<br><br>3. Statistically significant reduction in visits in IG vs CG |

| Study Number | Author & Year/ Country | Aim Design Theoretical model | Sample | Intervention(s) | Outcomes/measures and follow-up period | Results |
|---|---|---|---|---|---|---|
| | | | n=263 control group, mean age 77 (SD 6.6) | Control: usual care from the primary physician | 4. Change in percentage of patients with 1 or more visits to the emergency department<br><br>5. Change in proportion of patients with 1 or more home care visits<br><br>6. Change in number of patients with 1 or more nursing home placements<br>Patient reported health status at baseline, 1 and 2 years.<br><br>7. Change in Health Activities Questionnaire<br><br>8. Geriatric Depression Scale | (-1.5 vs 0.5, p=0.003)<br><br>4. No difference in change between IG and CG (1.2 vs -0.66, p=0.77)<br><br>5. No difference in change between IG and CG (1.8 vs -2.6, p=0.81)<br><br>6. No difference in change between IG and CG (5.0 vs -5.4, p=0.59)<br><br>7. No difference in change between IG and CG (0.03 vs 0.08, p=0.14<br><br>8. No difference in change between IG and CG (0.3 vs 0.5, p=0.52) |

| Study Number | Author & Year/<br><br>Country | Aim<br>Design<br>Theoretical model | Sample | Intervention(s) | Outcomes/measures and follow-up period | Results |
|---|---|---|---|---|---|---|
| | | | | | 9. Medications count<br><br>10. Social activities count<br><br>11. Symptom scale<br><br>12. SF-36 self-rated health<br><br>13. Nutrition checklist | 9. No difference in change between IG and CG (0.3 vs 0, p=0.26)<br><br>10. Significant increase in IG vs reduction in CG (0.2 vs -0.3, p=0.04)<br><br>11. No significant change in IG vs CG (-0.5 vs 1.0, p=0.08)<br><br>12. No significant change in IG vs CG (0 vs 0.1, p=0.08)<br><br>13. No significant change in IG or CG (0.3 vs 0, p=0.12) |
| 53 | Gustafson et al (1994) (69)<br><br>USA | Test the impact of an interactive, computerised, personal health support system on adults with HIV<br><br>RCT | N=107 in intervention group, mean age 34.8 years<br>n=97 in control group, mean age 34.5 years | Intervention: Participants were given a PC based Comprehensive Health Enhancement Support System (CHESS) in their homes for 6 or 3 months. This enables access to health information, asking | 1.Quality of life scores: Medical Outcomes Survey (MOS) at baseline, 2 and 5 months | 1.At 2 months the intervention group reported significantly improved cognitive functioning (p=0.053), more active lives (p=0.013), |

| Study Number | Author & Year/ Country | Aim Design Theoretical model | Sample | Intervention(s) | Outcomes/measures and follow-up period | Results |
|---|---|---|---|---|---|---|
| | | | | experts questions anonymously and reading personal accounts of others with similar problems.<br><br>Control: no details provided | <br><br><br><br><br><br><br><br><br><br><br><br><br><br><br><br><br><br><br><br><br><br><br><br><br><br><br><br>2.Use of ambulatory care services in 2 months | decreased negative emotion (p=0.013) and better social support (p=0.074) than controls. Depression, physical function, energy and participation in healthcare did not show significant differences between groups.<br>At 5 months the intervention group reported more active life (p=0.034), improved social support (p=0.017) and more active participation in their healthcare (p=0.020). There was no difference between groups in cognitive function, negative emotions, depression, physical function, or energy.<br><br>2. No difference in frequency of visits to |

| Study Number | Author & Year/ Country | Aim Design Theoretical model | Sample | Intervention(s) | Outcomes/measures and follow-up period | Results |
|---|---|---|---|---|---|---|
| | | | | | before and after intervention implementation | ambulatory care services between groups. Intervention group reported shorter visits than controls during the intervention (p=0.043) and were more likely to telephone providers both during (p=0.013) and after (p=0.094) the intervention. |
| | | | | | 3.Hospitalisation before, during and after intervention implementation | 3.Hospitalisations were lower for the intervention group than controls during the intervention (p=0.020) and shorter (p=0.009). These differences were not maintained after the intervention. |

| Study Number | Author & Year/<br><br>Country | Aim<br>Design<br>Theoretical model | Sample | Intervention(s) | Outcomes/measures and follow-up period | Results |
|---|---|---|---|---|---|---|
| 54 | Kinmonth et al (1998) (70) , UK | To assess the effect of additional training of practice nurses and general practitioners in patient centred care on lifestyle, psychological and physiological status of patients with type 2 diabetes.<br>Pragmatic parallel group design, randomisation between practice teams to routine care.<br>RCT. | N=41 practices<br>n=21 intervention practices and 142 patients<br>n=20 usual care practices and 108 patients.<br>250/360 patients (30-70 years)<br>Mean age 41.54(SD=9.83) years. | 1.5 days group training for the nurses and 0.5 days for doctors:<br><br>Reviewed evidence-based person-centred consulting and practised the skills they learnt with an experienced facilitator. Skills included active listening and negotiation of behavioural change. They produced materials including a booklet for patients, 'Diabetes in your hands' which encouraged patients to ask questions.<br><br>Comparison group nurses were offered similar support sessions focusing on use of guidelines and materials. | 1. Quality of life: Audit of diabetes dependent quality of life (ADDQoL)<br><br>2. Communication and satisfaction with treatment<br><br>3.Wellbeing: The wellbeing questionnaire<br><br>4. Blood pressure<br><br>5. Body mass index (kg/m$^2$) | 1. QoL mean in the intervention -1.09 and -1.23 in the control group (p=0.27).<br><br>2. Intervention showed better communication with doctors (odds 2.8 p<0.001), satisfaction with treatment (1.6 p=0.05)<br><br>3. Wellbeing: mean difference 2.8 (p=0.03)<br><br>4. Mean systolic BP 144.3 in the intervention and 142.8 in the control groups p=0.18 Diastolic BP 89.0 in the intervention and 87.2 in the control p=0.10<br><br>5. Mean BMI 31.3 in the intervention and |

| Study Number | Author & Year/<br><br>Country | Aim<br>Design<br>Theoretical model | Sample | Intervention(s) | Outcomes/measures and follow-up period | Results |
|---|---|---|---|---|---|---|
| | | | | | 6. Haemoglobin A1c % | 29.5 in the control p=0.03.<br><br>6. Mean HbA1c 7.07 in the IF and 7.17 in the control group (p=0.31). |

| Study Number | Author & Year/<br><br>Country | Aim<br>Design<br>Theoretical model | Sample | Intervention(s) | Outcomes/measures and follow-up period | Results |
|---|---|---|---|---|---|---|
| 55 | Landefeld (1995) (71), USA | To compare outcomes of people admitted to a unit especially designed to improve the functional outcomes of acutely ill older patients with standard care<br><br>RCT | n=651 people aged 70 or older admitted for general medical care at a teaching hospital<br>n=327 intervention group, mean age 80.2 (SD)<br>n=324 control group, mean age 80.1 (SD 6.6) | Intervention: Admission to a unit practising the Acute Care for Elders programme including a specially prepared environment, patient-centred care emphasizing independence, discharge planning aiming to discharge patients home and intensive review of medical care to minimise adverse effects of interventions and procedures.<br><br>Usual care: admission to acute care medical unit.<br>In both groups patients were assigned a primary nurse, two resident physicians and an attending physician. Staffing ratios and access to hospital support services including social work, physiotherapy, and nutrition. | Primary outcome:<br>1. Change from admission to discharge in the number of basic activities of daily living (ADLs) that the patient could perform independently<br><br>Secondary outcomes<br>2. Patients admitted from own home being discharged to a long-term care institution<br><br>3. Overall health status at discharge<br><br>4. Mean length of hospital stay<br><br>5. Mean total hospital charges | 1. IG had greater improvement compared to CG (p=0.009)<br>The mean ADLs performed independently at discharge were 3.6 for IG and 3.3 for CG (p=0.05)<br><br>2. Fewer IG patients discharged to institution than CG (14% vs 22%, p=0.01)<br><br>3.Better health status in IG than CG (p<0.001)<br><br>4. Not significant<br><br>5. Not significant |

1. Fortin M, Stewart M, Ngangue P, Almirall J, Bélanger M, Brown JB, et al. Scaling Up Patient-Centered Interdisciplinary Care for Multimorbidity: A Pragmatic Mixed-Methods Randomized Controlled Trial. Annals of Family Medicine. 2021;19(2):126-34.
2. de Batlle J, Massip M, Vargiu E, Nadal N, Fuentes A, Ortega Bravo M, et al. Implementing Mobile Health-Enabled Integrated Care for Complex Chronic Patients: Intervention Effectiveness and Cost-Effectiveness Study. JMIR Mhealth Uhealth. 2021;9(1):e22135.
3. Mielenz TJ, Tracy M, Jia H, Durbin LL, Allegrante JP, Arniella G, et al. Creation of the Person-Centered Wellness Home in Older Adults. Innovation in Aging. 2020;4(1).
4. Yu C, Choi D, Bruno BA, Thorpe KE, Straus SE, Cantarutti P, et al. Impact of MyDiabetesPlan, a Web-Based Patient Decision Aid on Decisional Conflict, Diabetes Distress, Quality of Life, and Chronic Illness Care in Patients With Diabetes: Cluster Randomized Controlled Trial. Journal of Medical Internet Research. 2020;22(9):N.PAG-N.PAG.
5. Bergsten U, Almehed K, Baigi A, Jacobsson LTH. A randomized study comparing regular care with a nurse-led clinic based on tight disease activity control and person-centred care in patients with rheumatoid arthritis with moderate/high disease activity: A 6-month evaluation. Musculoskeletal Care. 2019;17(3):215-25.
6. Berntsen GKR, Dalbakk M, Hurley JS, Bergmo T, Solbakken B, Spansvoll L, et al. Person-centred, integrated and pro-active care for multi-morbid elderly with advanced care needs: a propensity score-matched controlled trial. BMC health services research. 2019;19(1):682-.
7. Berendonk C, Kaspar R, Bär M, Hoben M. Improving Quality of Work life for Care Providers by Fostering the Emotional well-being of Persons with Dementia: A Cluster-randomized Trial of a Nursing Intervention in German long-term Care Settings. Dementia (London). 2019;18(4):1286-309.
8. Bökberg C, Behm L, Wallerstedt B, Ahlström G. Evaluation of person-centeredness in nursing homes after a palliative care intervention: pre- and post-test experimental design. BMC Palliative Care. 2019;18(1):44.
9. Britt HR, JaKa, M. M., Fernstrom, K. M., Bingham, P. E., Betzner, A. E., Taghon, J. R., Shippee, N. D., Shippee, T. P., Schellinger, S. E., & Anderson, E. W. . Quasi-Experimental Evaluation of LifeCourse on Utilization and Patient and Caregiver Quality of Life and Experience. . The American journal of hospice & palliative care 2019;36(5):408-16.
10. Hedman A, Eriksson G, von Koch L, Guidetti S. Five-year follow-up of a cluster-randomized controlled trial of a client-centred activities of daily living intervention for people with stroke. Clin Rehabil. 2019;33(2):262-76.
11. Bertilsson AS, Eriksson G, Ekstam L, Tham K, Andersson M, von Koch L, et al. A cluster randomized controlled trial of a client-centred, activities of daily living intervention for people with stroke: one year follow-up of caregivers. Clinical rehabilitation. 2016;30(8):765-75.
12. Guidetti S, Ranner M, Tham K, Andersson M, Ytterberg C, von Koch L. A “Client-Centred Activities of Daily Living” Intervention for Persons with Stroke: One-Year Follow-up of a Randomized Controlled Trial. Journal of Rehabilitation Medicine. 2015;47(7):605-11.
13. Bertilsson A-S, Ranner M, von Koch L, Eriksson G, Johansson U, Ytterberg C, et al. A client-centred ADL intervention: three-month follow-up of a randomized controlled trial. Scandinavian journal of occupational therapy. 2014;21(5):377-91.
14. Öhlén J, Sawatzky R, Pettersson M, Sarenmalm EK, Larsdotter C, Smith F, et al. Preparedness for colorectal cancer surgery and recovery through a person-centred

information and communication intervention - A quasi-experimental longitudinal design. PLoS One. 2019;14(12):e0225816.
15. Pirhonen L, Bolin K, Olofsson EH, Fors A, Ekman I, Swedberg K, et al. Person-Centred Care in Patients with Acute Coronary Syndrome: Cost-Effectiveness Analysis Alongside a Randomised Controlled Trial. PharmacoEconomics - Open. 2019;3(4):495-504.
16. Pirhonen L, Olofsson EH, Fors A, Ekman I, Bolin K. Effects of person-centred care on health outcomes-A randomized controlled trial in patients with acute coronary syndrome. Health Policy. 2017;121(2):169-79.
17. Fors A, Swedberg K, Ulin K, Wolf A, Ekman I. Effects of person-centred care after an event of acute coronary syndrome: Two-year follow-up of a randomised controlled trial. International Journal of Cardiology. 2017;249:42-7.
18. Fors A, Taft C, Ulin K, Ekman I. Person-centred care improves self-efficacy to control symptoms after acute coronary syndrome: A randomized controlled trial. European Journal of Cardiovascular Nursing. 2016;15(2):186-94.
19. Fors A, Gyllensten H, Swedberg K, Ekman I. Effectiveness of person-centred care after acute coronary syndrome in relation to educational level: Subgroup analysis of a two-armed randomised controlled trial. Int J Cardiol. 2016;221:957-62.
20. Fors A, Ekman I, Taft C, Björkelund C, Frid K, Larsson ME, et al. Person-centred care after acute coronary syndrome, from hospital to primary care - A randomised controlled trial. Int J Cardiol. 2015;187:693-9.
21. Wolf A, Fors A, Ulin K, Thorn J, Swedberg K, Ekman I. An eHealth Diary and Symptom-Tracking Tool Combined With Person-Centered Care for Improving Self-Efficacy After a Diagnosis of Acute Coronary Syndrome: A Substudy of a Randomized Controlled Trial. J Med Internet Res. 2016;18(2):e40.
22. Zakrisson AB, Arne M, Hasselgren M, Lisspers K, Ställberg B, Theander K. A complex intervention of self-management for patients with COPD or CHF in primary care improved performance and satisfaction with regard to own selected activities; A longitudinal follow-up. J Adv Nurs. 2019;75(1):175-86.
23. Arian M, Memarian R, Oghazian MB, Vakilian F, Badiee Z. The effect of a holistic care program on the reduction of iron over load in patients with beta-thalassemia major: A randomized clinical trial. Iranian Red Crescent Medical Journal. 2018;20 (4) (no pagination)(e60820).
24. Eggers C, Dano R, Schill J, Fink GR, Hellmich M, Timmermann L. Patient-centered integrated healthcare improves quality of life in Parkinson's disease patients: a randomized controlled trial. J Neurol. 2018;265(4):764-73.
25. Fors A, Blanck E, Ali L, Swedberg K, Ekman I. Person-centred telephone-support is effective in patients with chronic obstructive pulmonary disease and/or chronic heart failure-six-month follow-up of a randomized controlled trial. European Journal of Heart Failure. 2018;20 (Supplement 1):194.
26. Reed RL, Roeger L, Howard S, Oliver-Baxter JM, Battersby MW, Bond M, et al. A self-management support program for older Australians with multiple chronic conditions: A randomised controlled trial. Medical Journal of Australia. 2018;208(2):69-74.
27. Schafer I, Kaduszkiewicz H, Mellert C, Loffler C, Mortsiefer A, Ernst A, et al. Narrative medicine-based intervention in primary care to reduce polypharmacy: results from the cluster-randomised controlled trial MultiCare AGENDA. BMJ Open. 2018;8(1):e017653.
28. Thom DH, Willard-Grace R, Tsao S, Hessler D, Huang B, DeVore D, et al. Randomized Controlled Trial of Health Coaching for Vulnerable Patients with Chronic Obstructive Pulmonary Disease. Annals of the American Thoracic Society. 2018;15(10):1159-68.

29. Armstrong KA, Coyte PC, Brown M, Beber B, Semple JL. Effect of home monitoring via mobile app on the number of in-person visits following ambulatory surgery a randomized clinical trial. JAMA Surgery. 2017;152(7):622-7.
30. Feldthusen C, Dean E, Forsblad-d'Elia H, Mannerkorpi K. Effects of Person-Centered Physical Therapy on Fatigue-Related Variables in Persons With Rheumatoid Arthritis: A Randomized Controlled Trial. Arch Phys Med Rehabil. 2016;97(1):26-36.
31. Hansson E, Carlström E, Olsson LE, Nyman J, Koinberg I. Can a person-centred-care intervention improve health-related quality of life in patients with head and neck cancer? A randomized, controlled study. BMC Nurs. 2017;16:9.
32. Ko FWS, Cheung NK, Rainer TH, Lum C, Wong I, Hui DSC. Comprehensive care programme for patients with chronic obstructive pulmonary disease: A randomised controlled trial. Thorax. 2017;72(2):122-8.
33. Low LL, Tan SY, Ng MJM, Tay WY, Ng LB, Balasubramaniam K, et al. Applying the Integrated Practice Unit Concept to a Modified Virtual Ward Model of Care for Patients at Highest Risk of Readmission: A Randomized Controlled Trial. PloS one. 2017;12(1):e0168757-e.
34. Wichit N, Mnatzaganian G, Courtney M, Schulz P, Johnson M. Randomized controlled trial of a family-oriented self-management program to improve self-efficacy, glycemic control and quality of life among Thai individuals with Type 2 diabetes. Diabetes Research and Clinical Practice. 2017;123:37-48.
35. Larsson A, Palstam A, Löfgren M, Ernberg M, Bjersing J, Bileviciute-Ljungar I, et al. Resistance exercise improves muscle strength, health status and pain intensity in fibromyalgia--a randomized controlled trial. Arthritis Res Ther. 2015;17(1):161.
36. Ericsson A, Palstam A, Larsson A, Löfgren M, Bileviciute-Ljungar I, Bjersing J, et al. Resistance exercise improves physical fatigue in women with fibromyalgia: a randomized controlled trial. Arthritis Res Ther. 2016;18:176.
37. Hansson E, Ekman I, Swedberg K, Wolf A, Dudas K, Ehlers L, et al. Person-centred care for patients with chronic heart failure - a cost-utility analysis. Eur J Cardiovasc Nurs. 2016;15(4):276-84.
38. Ulin K, Olsson LE, Wolf A, Ekman I. Person-centred care - An approach that improves the discharge process. European Journal of Cardiovascular Nursing. 2016;15(3):e19-26.
39. Ekman I, Wolf A, Olsson LE, Taft C, Dudas K, Schaufelberger M, et al. Effects of person-centred care in patients with chronic heart failure: the PCC-HF study. European Heart Journal. 2012;33(9):1112-9.
40. Dudas K, Olsson LE, Wolf A, Swedberg K, Taft C, Schaufelberger M, et al. Uncertainty in illness among patients with chronic heart failure is less in person-centred care than in usual care. European Journal of Cardiovascular Nursing. 2013;12(6):521-8.
41. Jutterstrom L, Hornsten A, Sandstrom H, Stenlund H, Isaksson U. Nurse-led patient-centered self-management support improves HbA1c in patients with type 2 diabetes-A randomized study. Patient Education and Counseling. 2016;99(11):1821-9.
42. Olsson LE, Hansson E, Ekman I. Evaluation of person-centred care after hip replacement-a controlled before and after study on the effects of fear of movement and self-efficacy compared to standard care. BMC Nurs. 2016;15(1):53.
43. Olsson L-E, Karlsson J, Berg U, Kärrholm J, Hansson E. Person-centred care compared with standardized care for patients undergoing total hip arthroplasty—a quasi-experimental study. Journal of Orthopaedic Surgery and Research. 2014;9(1):95.
44. Or C, Tao D. A 3-Month Randomized Controlled Pilot Trial of a Patient-Centered, Computer-Based Self-Monitoring System for the Care of Type 2 Diabetes Mellitus and Hypertension. Journal of Medical Systems. 2016;40(4):81.

45. Sahlen K-G, Boman K, Brannstrom M. A cost-effectiveness study of person-centered integrated heart failure and palliative home care: Based on a randomized controlled trial. Palliative Medicine. 2016;30(3):296-302.
46. Brannstrom M, Boman K. Effects of person-centred and integrated chronic heart failure and palliative home care. PREFER: a randomized controlled study. European Journal of Heart Failure. 2014;16(10):1142-51.
47. Slok AH, Kotz D, van Breukelen G, Chavannes NH, Rutten-van Molken MP, Kerstjens HA, et al. Effectiveness of the Assessment of Burden of COPD (ABC) tool on health-related quality of life in patients with COPD: a cluster randomised controlled trial in primary and hospital care. BMJ Open. 2016;6(7):e011519.
48. Windrum P, Garcia-Goni M, Coad H. The Impact of Patient-Centered versus Didactic Education Programs in Chronic Patients by Severity: The Case of Type 2 Diabetes Mellitus. Value in Health. 2016;19(4):353-62.
49. Yu DSF. Effects of a Health and Social Collaborative Case Management Model on Health Outcomes of Family Caregivers of Frail Older Adults: Preliminary Data from a Pilot Randomized Controlled Trial. Journal of the American Geriatrics Society. 2016;64(10):2144-8.
50. Hernandez C, Alonso A, Garcia-Aymerich J, Serra I, Marti D, Rodriguez-Roisin R, et al. Effectiveness of community-based integrated care in frail COPD patients: A randomised controlled trial. npj Primary Care Respiratory Medicine. 2015;25 (no pagination)(15022).
51. Kikkenborg Berg S, Stoier L, Moons P, Zwisler AD, Winkel P, Ulrich Pedersen P. Emotions and health: findings from a randomized clinical trial on psychoeducational nursing to patients with implantable cardioverter defibrillator. The Journal of cardiovascular nursing. 2015;30(3):197-204.
52. Larsson I, Fridlund B, Arvidsson B, Teleman A, Svedberg P, Bergman S. A nurse-led rheumatology clinic versus rheumatologist-led clinic in monitoring of patients with chronic inflammatory arthritis undergoing biological therapy: A cost comparison study in a randomised controlled trial. BMC Musculoskeletal Disorders. 2015;16 (1) (no pagination)(817).
53. Larsson I, Fridlund B, Arvidsson B, Teleman A, Bergman S. Treatment outcomes from a nurse-led rheumatology clinic in monitoring of anti-TNF therapy-a randomised controlled trial. Arthritis and Rheumatism. 2012;10):S667.
54. Lowther K, Selman L, Simms V, Gikaara N, Ahmed A, Ali Z, et al. Nurse-led palliative care for HIV-positive patients taking antiretroviral therapy in Kenya: a randomised controlled trial. Lancet HIV. 2015;2(8):e328-34.
55. Kelechi TJ, Mueller M, Spencer C, Rinard B, Loftis G. The effect of a nurse-directed intervention to reduce pain and improve behavioral and physical outcomes in patients with critically colonized/infected chronic leg ulcers. Journal of Wound, Ostomy, & Continence Nursing. 2014;41(2):111-21.
56. Young JM, Butow PN, Walsh J, Durcinoska I, Dobbins TA, Rodwell L, et al. Multicenter randomized trial of centralized nurse-led telephone-based care coordination to improve outcomes after surgical resection for colorectal cancer: the CONNECT intervention. Journal of Clinical Oncology. 2013;31(28):3585-91.
57. Chochinov HM, Kristjanson LJ, Breitbart W, McClement S, Hack TF, Hassard T, et al. Effect of dignity therapy on distress and end-of-life experience in terminally ill patients: a randomised controlled trial. Lancet Oncology. 2011;12(8):753-62.
58. Goelz T, Wuensch A, Stubenrauch S, Ihorst G, de Figueiredo M, Bertz H, et al. Specific training program improves oncologists' palliative care communication skills in a randomized controlled trial. Journal of Clinical Oncology. 2011;29(25):3402-7.

59. Murphy SL, Lyden AK, Smith DM, Dong Q, Koliba JF. Effects of a tailored activity pacing intervention on pain and fatigue for adults with osteoarthritis. American Journal of Occupational Therapy. 2010;64(6):869-76.
60. Wolff JL, Giovannetti ER, Boyd CM, Reider L, Palmer S, Scharfstein D, et al. Effects of guided care on family caregivers. The Gerontologist. 2010;50(4):459-70.
61. Dobscha SK, Corson K, Perrin NA, Hanson GC, Leibowitz RQ, Doak MN, et al. Collaborative care for chronic pain in primary care: a cluster randomized trial. JAMA. 2009;301(12):1242-52.
62. Machado LA, Azevedo DC, Capanema MB, Neto TN, Cerceau DM. Client-Centered Therapy vs Exercise Therapy for Chronic Low Back Pain: A Pilot Randomized Controlled Trial in Brazil. Pain Medicine. 2007;8(3):251-8.
63. Glasgow RE, Nutting PA, King DK, Nelson CC, Cutter G, Gaglio B, et al. Randomized effectiveness trial of a computer-assisted intervention to improve diabetes care. Diabetes Care. 2005;28(1):33-9.
64. Mills PD, Harvey PW. Beyond community-based diabetes management and the COAG coordinated care trial. Australian Journal of Rural Health. 2003;11(3):131-7.
65. Kennedy A, Nelson E, Reeves D, Richardson G, Roberts C, Robinson A, et al. A randomised controlled trial to assess the impact of a package comprising a patient-orientated, evidence-based self-help guidebook and patient-centred consultations on disease management and satisfaction in inflammatory bowel disease. Health Technology Assessment. 2003;7(28).
66. Martin IR, McNamara D, Sutherland FR, Tilyard MW, Taylor DR. Care plans for acutely deteriorating COPD: a randomized controlled trial. Chronic respiratory disease. 2004;1(4):191-5.
67. Alamo MM, Moral RR, Perula de Torres LA. Evaluation of a patient-centred approach in generalized musculoskeletal chronic pain/fibromyalgia patients in primary care. Patient Education & Counseling. 2002;48(1):23-31.
68. Sommers LS, Marton KI, Barbaccia JC, Randolph J. Physician, nurse, and social worker collaboration in primary care for chronically ill seniors. Archives of Internal Medicine. 2000;160(12):1825-33.
69. Gustafson DH, Hawkins RP, Boberg EW, Bricker E, Pingree S, Chan CL. The use and impact of a computer-based support system for people living with AIDS and HIV infection. Proc Annu Symp Comput Appl Med Care. 1994:604-8.
70. Kinmonth AL, Woodcock A, Griffin S, Spiegal N, Campbell MJ. Randomised controlled trial of patient centred care of diabetes in general practice: Impact on current wellbeing and future disease risk. British Medical Journal. 1998;317(7167):1202-8.
71. Landefeld CS, Palmer RM, Kresevic DM, Fortinsky RH, Kowal J. A randomized trial of care in a hospital medical unit especially designed to improve the functional outcomes of acutely ill older patients. New England Journal of Medicine. 1995;332(20):1338-44.
