# Supplementary material for: A systematic review of impact of person-centred interventions for serious physical illness in terms of outcomes and costs
Source: BMJ Open. 2022 Jul 13;12(7):e054386. doi: 10.1136/bmjopen-2021-054386 (PMC9280891; doi:10.1136/bmjopen-2021-054386)
Supplement: Supplementary data [file bmjopen-2021-054386supp003.pdf]

Supplementary file 3: Risk of Bias in the studies (Joanna Briggs Institute Critical Appraisal checklist)

Yes: means good and no risk of bias, No: means there was risk of bias, ITTA: Intention to treat analysis, IG: Intervention group, CG: control group

	Author	Random allocation	Allocation concealment	Baseline similarity/Comparable at entry	Blinding of participants	Blinding of those delivering treatment	Blinding of outcome assessors	Identical except intervention	Relatively complete follow-up achieved	Participants analysed in the groups originally randomised	Outcomes measures same between group	Outcomes assessed in reliable way	Statistical analysis appropriate	Total YES scores
1	Fortin et al 2021	Yes	Yes	Yes	No	No	Unclear	Yes	Yes	Yes	Yes	Yes	Yes	9/12
2	Yu et al 2020	Yes	Yes	No	No	No	Yes	Yes	No, 71%	Yes	Yes	Yes	Yes	8/12
3	de Batlle, 2020	Unclear	Unclear	No	No	Unclear	NA assessed through a web-based survey/mail	Yes	Yes, 87%	Yes, Modified ITTA	Yes	Yes	Yes	6/12
4	Mielenz et al 2020	Yes	Unclear	Yes	No	No	Yes	Yes	Yes, 97%	Yes, Modified	Yes	Yes	Yes	9/12
5	Bergsten et al 2019	Yes	Unclear	No	No	No	Yes	Yes	Yes, 83%	Yes	Yes	Yes	Yes	8/12
6	Berntsen et al (2019)	No	N/A	No	No	No	Unclear	Yes	Unclear	Yes	Yes	Yes	Yes	5/12
7	Berondonk (2019)	Yes	Yes	Yes	No	No	Unclear	Yes	Yes, 82%	Yes, Modified ITTA	Yes	Yes	Yes	9/12
8	Bokberg et al (2019)	No	N/A	No	No	No	Yes	Yes	No	Yes, Modified ITTA	Yes	Yes	Yes	6/12
9	Britt et al (2019)	No, patients recruited at separate hospitals. Those declining participation in IG were offered inclusion in CG	No	CG were younger, more likely to be married and more likely to live at home. CG were more likely to have a cardiovascular, and less likely to have dementia as a primary diagnosis.	No	No	No	Yes	No, 51% completed 12 months	Unclear	Yes	Yes	Yes	4/12

	Author	Random allocation	Allocation concealment	Baseline similarity/Comparable at entry	Blinding of participants	Blinding of those delivering treatment	Blinding of outcome assessors	Identical except intervention	Relatively complete follow-up achieved	Participants analysed in the groups originally randomised	Outcomes measures same between group	Outcomes assessed in reliable way	Statistical analysis appropriate	Total YES scores
10	Hedman, et al 2019 Bertilsson et al (2016), Guidenti et al (2015) and Bertilsson et al (2014) One study reporting three papers	Unclear	Unclear	Unclear	No	No	Unclear	Yes	Yes, 81%	Yes, Modified ITTA	Yes	Yes	Yes	6/12
11	Ohlen et al (2019)	No	N/A	Yes	No	No	N/A	Yes	Yes, 82%	Yes, ITTA	Yes	Yes	Yes	7/12
12	Pirhonen et al 2019 Pirhonen et al 2016, Fors et al (2017); Fors et al (2016a)a Fors et al (2016b) Wolf et al 2016 and Fors et al 2015 Seven papers one study	Yes	Yes	Yes	No	No	Not clear	Yes	Yes, 91%	Yes	Yes	Yes	Yes	9/12
13	Zakrisson et al (2019)	Yes	Yes	No	No	No	Yes	Yes	No	Yes	Yes	Yes	Yes	8/12
14	Arian et al (2018)	Yes	Unclear	Yes	No	No	Yes	Yes	No	Yes	Yes	Yes	Yes	8/12

	Author	Random allocation	Allocation concealment	Baseline similarity/Comparable at entry	Blinding of participants	Blinding of those delivering treatment	Blinding of outcome assessors	Identical except intervention	Relatively complete follow-up achieved	Participants analysed in the groups originally randomised	Outcomes measures same between group	Outcomes assessed in reliable way	Statistical analysis appropriate	Total YES scores
15	Eggers, 2018	Yes	Yes	Yes	No	No	Unclear	Yes	Yes, 86%	Yes, Modified ITTA	Yes	Yes	Yes	9/12
16	Fors et al (2018)	Yes	Not clear	Yes	No	No	N/A: patients self-completed and sent by post	Yes	Yes, 91%	Yes	Yes	Yes	Yes	8/12
17	Reed et al 2018)	Yes	Yes	Yes	Yes	No	Yes	Yes	Yes, 91%	Yes	Yes	Yes	Yes	11/12
18	Schäfer et al (2018)	Yes	Yes	No difference in characteristics of GPs and practices between groups.<br>More female patients in the control group.	No	No	No	Yes	Yes, 93% completed	Yes, Modified ITTA	No	Yes	Yes	7/12
19	Thom et al 2018	Yes	Yes	Yes	No	No	Unclear	Yes	Yes, 82%	Yes	Yes	Yes	Yes	9/12
20	Armstrong et al (2017)	Unclear	Yes	Yes	No	No	Unclear	Yes	Yes, 93%	Yes	Yes	Yes	Yes	8/12
21	Feldthusen et al 2017	Yes	Yes	Yes	No	Yes	Yes	Yes	Yes, 96%	Yes	Yes	Yes	Yes	11/12
22	Hansson et al 2017<br><br>Gyllensten et al 2019	Yes	Yes	No	No	No	No	Yes	Yes, 92%	Yes	Yes	Yes	Yes	8/12
23	Ko et al (2017)	Yes	Yes	IG had higher FEV_1% of predicted	No	No	Yes	Yes	No, 79%	Yes	Yes	Yes	Yes	8/12
24	Low et al (2017)	Yes	Yes	Yes	No	No	Yes	Yes	Yes, 87%	Yes	Yes	Yes	Yes	10/12
25	Wichit et al (2017)	Yes	Yes	IG were older	Yes	No	Yes	Yes	Yes, 96%	Yes	Yes	Yes	Yes	10/12
26	Ericsson et al 2016	Yes	Yes	Yes	No	No	Yes	Yes	No, 70%	Yes, ITTA	Yes	Yes	Yes	9/12

	Author	Random allocation	Allocation concealment	Baseline similarity/Comparable at entry	Blinding of participants	Blinding of those delivering treatment	Blinding of outcome assessors	Identical except intervention	Relatively complete follow-up achieved	Participants analysed in the groups originally randomised	Outcomes measures same between group	Outcomes assessed in reliable way	Statistical analysis appropriate	Total YES scores
	Larsson et al 2015<br><br>Two papers one study													
27	Hansson et al 2016; Ulin et al 2016; Ekman et al (2012) and Dudas et al 2012 Four papers one study	No	N/A	No	No	No	Unclear	Yes	Yes, 80%	Yes, ITTA	Yes	Yes	Yes	6/12
28	Jutterström et al (2016)	Yes	Yes	No, Greater numbers in the Group intervention group. Group intervention had greater HbA1c than External control group, and lower total cholesterol than internal control. External control group were more likely to have diet and/or insulin treatment	No	No	No	Yes	Yes, 88%	Yes, Modified ITTA	Yes	Yes	Yes	8/12
29	Olsson et al 2016 and	No	N/A	No	No	No	Unclear	Yes	Yes, 99%	Yes	Yes	Yes	Yes	6/12

	Author	Random allocation	Allocation concealment	Baseline similarity/Comparable at entry	Blinding of participants	Blinding of those delivering treatment	Blinding of outcome assessors	Identical except intervention	Relatively complete follow-up achieved	Participants analysed in the groups originally randomised	Outcomes measures same between group	Outcomes assessed in reliable way	Statistical analysis appropriate	Total YES scores
	Olsson et al 2014<br><br>Two papers one study													
30	Or and Tao (2016)	Yes	Yes	Yes	No	No	No	Yes	Yes, 87%	Yes, Modified ITTA	No, IG self monitoring data captured on a tablet, CG from log book records.	Yes	Yes	8/12
31	Sahlen et al (2016); Brännstrom & Boman (2014) One study two papers	Unclear	Unclear	IG older	No	No	Unclear	Yes	Yes, 83%	Yes, Modified ITTA	Yes	Yes	Yes	6/12
32	Slok et al (2016)	Yes	Yes	IG more likely to be a current smoker	No	No	No	Yes	Yes, 82%	Yes, Modified ITTA	Yes	Yes	Yes	8/12
33	Windrum et al (2016)	Unclear	Unclear	Yes	No	Yes	Unclear	Yes	Unclear	Unclear	Unclear	Unclear	Yes	4/12
34	Yu (2016)	Yes	Unclear	Yes	No	No	Yes	Yes	Yes	Yes, Modified ITTA	Yes	Yes	Yes	9/12
35	Hernández et al (2015)	Yes	Yes	CG more likely to have had influenza and pneumococccal vaccines	No	No	Yes	Yes	No, 71%	Yes, Modified ITTA	Yes	Yes	Yes	8/12
36	Kikkenborg et al (2015)	Unclear	Unclear	Yes	No	No	Yes	Yes	Yes, 84%	Yes, Modified ITTA	Yes	Yes	Yes	8/12

	Author	Random allocation	Allocation concealment	Baseline similarity/Comparable at entry	Blinding of participants	Blinding of those delivering treatment	Blinding of outcome assessors	Identical except intervention	Relatively complete follow-up achieved	Participants analysed in the groups originally randomised	Outcomes measures same between group	Outcomes assessed in reliable way	Statistical analysis appropriate	Total YES scores
37	Larsson et al (2015) and 2013 One study two papers	Yes	Yes	Yes	No	No	No	Yes	Yes	Yes	Yes	Yes	Yes	9/12
38	Lowther et al (2015)	Yes	Yes	CG had been diagnosed with HIV for longer and been taking ART for longer than IG.	No	No	No	Yes	Yes, 95%	Yes, Modified ITTA	Yes	Yes	Yes	8/12
39	Kelechi et al (2014)	Yes	Not clear	Greater motivation in IG Patient baseline demographic characteristics not reported by group	No	No	Not clear	Yes	Yes, 88%	Yes, Modified ITTA	Yes	Not stated	Yes	6/12
40	Young et al (2013)	Yes	Yes	IG more likely to have private health insurance, were admitted to a private hospital and had a stoma created.	No	No	Unclear	Yes	Yes, 88%	Yes, Modified ITTA	Yes	Yes	Yes	8/12
41	Chochinov et al (2011)	Yes	Yes	Yes	No	No	Yes	Yes	No, 74%	Yes, Modified ITTA	Yes	Unclear	Yes	8/12
42	Goelz et al (2011)	Yes	Yes	No	No	Unclear	Unclear	Yes	Yes	Yes	Yes	Yes	Yes	8/12
43	Murphy et al (2010)	Yes	Unclear	Yes	No	No	Yes	Yes	No, 74%	Yes, Modified ITTA	Yes	Unclear	Yes	7/12
44	Wolff et al (2010)	Unclear, Cluster	Unclear	CG more likely to be female	Unclear	No	Yes	Yes	No, 69% of patients and	Yes, Modified ITTA	Yes	Yes	Yes	6/12

	Author	Random allocation	Allocation concealment	Baseline similarity/Comparable at entry	Blinding of participants	Blinding of those delivering treatment	Blinding of outcome assessors	Identical except intervention	Relatively complete follow-up achieved	Participants analysed in the groups originally randomised	Outcomes measures same between group	Outcomes assessed in reliable way	Statistical analysis appropriate	Total YES scores
		randomization		and less educated					64% of caregivers					
45	Dobscha et al (2009)	Unclear	Yes	Yes	Yes	Yes	Yes	Yes	Yes, 90%	Yes, ITTA	Yes	Yes	Yes	11/12
46	Machado et al (2007)	Yes	Yes	IG had longer duration of symptoms, more likely to be female and more likely to not be working due to lower back pain	No	No	Yes	Yes	Yes, 81%	Yes, Modified ITTA	Yes	Yes	Yes	9/12
47	Glasgow et al 2005	Yes	Yes	Yes	No	No	unclear	Yes	Yes, 83%	Yes	Yes	Yes	Yes	9/12
48	Mills and Harvey (2003)	Unclear	Unclear	No	No	No	Unclear	Yes	No	Yes, modified ITTA	Yes	Yes	Yes	5/12
49	Kennedy et al (2004)	Unclear	Unclear	IG more likely to be off work with long term sickness	No	No	Unclear	Differences in discharge policies between centres.	Yes, 87%	Yes, ITTA	Yes	Unclear	Yes	4/12
50	Martin et al, (2004),	Unclear	Unclear	CG were more likely to be male and have greater cigarette consumption.	No	No	Unclear	Yes	Yes, 83%	Yes, Modified ITTA	Yes	Yes	Yes	6/12
51	Alamo et al (2002)	Unclear	Unclear	IG more tender points and more likely to describe pain as never/hardly ever a problem.	No	No	Yes	Yes	No, 74%	Yes	Yes	Yes	Yes	6/12
52	Sommers et al (2000)	Yes	Unclear	No	No	No	Unclear	Yes	No	Yes, Modified ITTA	Yes	Yes	Yes	6/12

	Author	Random allocation	Allocation concealment	Baseline similarity/Comparable at entry	Blinding of participants	Blinding of those delivering treatment	Blinding of outcome assessors	Identical except intervention	Relatively complete follow-up achieved	Participants analysed in the groups originally randomised	Outcomes measures same between group	Outcomes assessed in reliable way	Statistical analysis appropriate	Total YES scores
53	Gustafson et al (1994)	Yes	Yes	Yes	No	No	Unclear	Yes	Yes, 84%	Yes, Modified ITTA	Yes	Unclear	Yes	8/12
54	Kinmonth et al (1998)	Yes	Yes	Yes	Unclear	No	Unclear	Yes	No, 69%	Yes, Modified ITTA	Yes	Yes	Yes	8/12
55	Landefeld et al 1995	Yes	Unclear	Yes	No	No	No	Yes	Yes	Yes, Modified ITTA	Yes	Yes	Yes	8/12
