# Supplementary material for: A systematic review of impact of person-centred interventions for serious physical illness in terms of outcomes and costs
Source: BMJ Open. 2022 Jul 13;12(7):e054386. doi: 10.1136/bmjopen-2021-054386 (PMC9280891; doi:10.1136/bmjopen-2021-054386)
Supplement: Author's manuscript [file bmjopen-2021-054386.draft_revisions.pdf]

BMJ Open is committed to open peer review. As part of this commitment we make the peer review history of every article we publish publicly available.

When an article is published we post the peer reviewers' comments and the authors' responses online. We also post the versions of the paper that were used during peer review. These are the versions that the peer review comments apply to.

The versions of the paper that follow are the versions that were submitted during the peer review process. They are not the versions of record or the final published versions. They should not be cited or distributed as the published version of this manuscript.

BMJ Open is an open access journal and the full, final, typeset and author-corrected version of record of the manuscript is available on our site with no access controls, subscription charges or pay-per-view fees (http://bmjopen.bmj.com).

If you have any questions on BMJ Open's open peer review process please email info.bmjopen@bmj.com

BMJ Open

A systematic review of impact of person-centred interventions for serious physical illness in terms of outcomes and costs.

Journal:	*BMJ Open*
Manuscript ID	bmjopen-2021-054386
Article Type:	Original research
Date Submitted by the Author:	11-Jun-2021
Complete List of Authors:	Bashan Nkhoma, Kennedy; King's College London, Florence Nightingale Faculty of Nursing Midwifery and Palliative Care<br>Cook, Amelia; King's College London, Cicely Saunders Institute for Palliative Care, Policy and Rehabilitation<br>Giusti, Alessandra; King's College London, Cicely Saunders Institute<br>Farrant , Lindsay ; University of Cape Town Faculty of Health Sciences, School of Public Health and Family Medicine<br>Petrus , Ruwayda; University of KwaZulu-Natal College of Humanities, School of Applied Human Sciences<br>Petersen, I.; Univ KwaZulu Natal, Centre for Rural Health<br>Gwyther, Liz; University of Cape Town Faculty of Health Sciences, School of Public Health and Family Medicine<br>Venkatapuram, Sridhar; King's College London,<br>Harding, Richard; King's College London, of Palliative Care, Policy and Rehabilitation ;
Keywords:	Health policy < HEALTH SERVICES ADMINISTRATION & MANAGEMENT, Quality in health care < HEALTH SERVICES ADMINISTRATION & MANAGEMENT, Clinical trials < THERAPEUTICS, Health economics < HEALTH SERVICES ADMINISTRATION & MANAGEMENT

SCHOLARONE™
Manuscripts

I, the Submitting Author has the right to grant and does grant on behalf of all authors of the Work (as defined in the below author licence), an exclusive licence and/or a non-exclusive licence for contributions from authors who are: i) UK Crown employees; ii) where BMJ has agreed a CC-BY licence shall apply, and/or iii) in accordance with the terms applicable for US Federal Government officers or employees acting as part of their official duties; on a worldwide, perpetual, irrevocable, royalty-free basis to BMJ Publishing Group Ltd ("BMJ") its licensees and where the relevant Journal is co-owned by BMJ to the co-owners of the Journal, to publish the Work in this journal and any other BMJ products and to exploit all rights, as set out in our licence.

The Submitting Author accepts and understands that any supply made under these terms is made by BMJ to the Submitting Author unless you are acting as an employee on behalf of your employer or a postgraduate student of an affiliated institution which is paying any applicable article publishing charge ("APC") for Open Access articles. Where the Submitting Author wishes to make the Work available on an Open Access basis (and intends to pay the relevant APC), the terms of reuse of such Open Access shall be governed by a Creative Commons licence – details of these licences and which Creative Commons licence will apply to this Work are set out in our licence referred to above.

Other than as permitted in any relevant BMJ Author's Self Archiving Policies, I confirm this Work has not been accepted for publication elsewhere, is not being considered for publication elsewhere and does not duplicate material already published. I confirm all authors consent to publication of this Work and authorise the granting of this licence.

For peer review only

A systematic review of impact of person-centred interventions for serious physical illness in terms of outcomes and costs.

Kennedy Bashan Nkhoma[1], Amelia Cook[1], Alessandra Giusti[1], Lindsay Farrant[2], Ruwayda Petrus[3], Inge Petersen[4], Liz Gwyther[2], Sridhar Venkatapuram[5], Richard Harding[1]

Affiliations

1. King's College London, Cicely Saunders Institute of Palliative Care, Policy and Rehabilitation, London, SE5 9PJ, United Kingdom

2. University of Cape Town, School of Public Health and Family Medicine (Palliative Medicine) Falmouth Building, Cape Town, Western Cape, South Africa

3. University of KwaZulu-Natal, College of Humanities, School of Applied Human Sciences Durban, ZA 4001, South Africa

4. University of KwaZulu Natal, Centre for Rural Health, Howard College, Durban, ZA 4001, South Africa

5. King's College London, King's Global Health Institute, Franklin Wilkins Building, 150 Stamford Street, London, UK SE1 9NH

Correspondence: Dr Kennedy B. Nkhoma

Email: kennedy.nkhoma@kcl.ac.uk Telephone: +44 (0)207 848 5566

For peer review only

Abstract

Introduction

Person-centred care is being internationally recognised as a critical attribute of high-quality healthcare. However, the concept has been criticised for being poorly theorized and operationalised. Focusing on delivery, we aimed to review and evaluate the evidence from interventions that aimed to deliver person-centred care (PCC) for people with serious physical illness.

Methods

Systematic review of literature using PRISMA guidelines. We searched Amed, Assian, CINAHL, Cochrane Library, Embase, Medline, PsycInfo, Scopus and Web of Science data bases, using the following key concepts: Patient/person-centred care, family-centred care, family-based care, individualised care, holistic care, value-based care. Due to heterogeneity of interventions and populations studied, narrative synthesis was conducted. Study quality was appraised using the Joanna Briggs checklist.

Results

We screened n=4796 papers and n=70 papers (reporting n=54 different studies) were retained in the review. Most of these studies n=45 studies were RCT's. We synthesised findings across two main models: 1) Studies with self-management components and 2) technology-based interventions.

Self-management component. The interventions consisted of training of patients and/or caregivers or staff. Some studies reported that interventions had effect in reduction hospital admissions, improving quality of life and reducing costs of care, while some studies reported no effects on quality of life, self-efficacy, and health utilisation.

For peer review only - http://bmjopen.bmj.com/site/about/guidelines.xhtml

Technology based interventions: consisted of mobile phone, mobile app, tablet/computer, and video. Although some interventions showed improvements for self-efficacy, hospitalisations and length of stay, quality of life did not improve across most studies.

Conclusions

PCC interventions using self-management have some effects in reducing health utilisation, costs of care, and improving quality of life. Technology based interventions also reduces health utilisation and improves self-efficacy but has no effect on quality of life. However very few studies used self-management and technology approaches. Further work is needed to identify how self-management and technology approaches can be used manage serious illness.

Word count: 6580

Review Registration number: PROSPERO CRD42018108302

Key words: Person-centred care, Serious physical illness, Systematic Reviews, Self-management interventions, technology-based interventions

For peer review only - http://bmjopen.bmj.com/site/about/guidelines.xhtml

Strengths and limitations of this study

- A study provides a systematic review of the evidence on the impact of person-centred interventions for serious physical illness in terms of outcomes and costs.
- We used robust procedures for systematic reviewing and quality assessment of the studies included, in line with Preferred Reporting Items for Systematic Reviews and Meta-Analyses (PRISMA) reporting guidelines.
- Most of the studies identified and included were conducted in high income countries.
- We conducted a narrative synthesis due to heterogenicity of the studies included (different disease population, different outcome measures and different trial end points).
- Most of the studies included did not state the theoretical framework underpinning the person-centred interventions. However, many studies that reported the theoretical framework used the Goldenberg theory of person-centred care and were conducted in Sweden across various clinical settings.

What is already known?

- Person-centred care is internationally recognised as an important component of achieving high-quality healthcare and is essential to achieving the Universal Health Coverage goals.
- Patients with serious physical illness need person-centred care; due to complex clinical needs associated with serious illness. They also require involvement of family or primary caregivers throughout the disease trajectory as they navigate the health care delivery system to help with day-to-day care and decision making about patient management.

For peer review only - http://bmjopen.bmj.com/site/about/guidelines.xhtml

- Qualitative evidence supports the Santana et al model of PCC and suggest that additional domains of PCC should be given visibility: family and friend involvement and support; promoting continuation of normality and self-identity; structuring service organisation to enable continuity of care and patient navigation.
- PCC emphasises the need to respect, listen to, and understand patients and families. Furthermore, provision of honest, complete, clear, and comprehensive information to patient and families to enable them to make appropriate decision about their care.

What are the new findings?

- Person-centred care can be implemented across different settings: primary, secondary, and tertiary (for example in-patient, out-patient, emergency care, surgery, residential homes etc) and across different disease conditions (for example cancer, COPD, heart failure, HIV etc).
- PCC interventions reduce costs of care in heart failure, COPD, acute coronary syndrome, and rheumatology populations, however few studies reported data on this outcome.
- There is evidence to suggest that PCC interventions can be implemented using self-management or technology approaches. PCC interventions using self-management have some effects in reducing health utilisation, costs of care, and improving quality of life. Technology based interventions also reduces health utilisation and improves self-efficacy but has no effect on quality of life. However very few studies used self-management and technology approaches.
- Most PCC interventions were conducted in high-income countries. All technology-based interventions were conducted in high income and middle-income countries.
- Most studies did not state the theoretical assumption/framework which informed the PCC intervention. Studies which stated the theoretical framework were

For peer review only - http://bmjopen.bmj.com/site/about/guidelines.xhtml

predominantly informed by the Gothenburg person-centred care and were conducted in Sweden.

- Evidence on effectiveness of PCC interventions among health professionals and family caregivers is inconclusive.

What do the new findings imply?

- Institutions should consider implementing person-centred care interventions using available resources.
- Health service researchers should consider incorporating costs of person-centred care as an outcome when designing and evaluating complex interventions.
- There is a need for PCC interventions to be conducted in low-and middle-income countries across various settings.
- There is a need for more work on PCC interventions delivered to health care professionals and family caregivers of patients with serious illness.
- There is a need to consider the theoretical underpinnings of PCC when designing, developing, and evaluating complex interventions in serious illness.
- Further work is needed to demonstrate effectiveness of self-management and technology based PPC interventions across different disease conditions.

For peer review only - http://bmjopen.bmj.com/site/about/guidelines.xhtml

Introduction

World Health Organization (WHO) guidance emphasise person-centredness as a core component of health professionals' skills and quality health-care (1). Integrated, person-centred care (PCC) is essential to achieving Universal Health Coverage (UHC) (2, 3). The core elements of PCC in health policy, medicine and nursing have been described as: patient participation and involvement, patient relationship with the healthcare professionals and context where care is delivered (4). Person-centred care aims to give meaningful assessment and equal weight to a patient's subjective understanding of their illness, including their needs, concerns, and expectations. This occurs, alongside a biomedical diagnosis; PCC also promote their equal participation in treatment decision-making and empowers them to take greater control of their own health and management of their condition (5).

Our first systematic review identified and appraised the empirical evidence underpinning conceptualisations of 'person centredness' for serious illness (6). Serious illness, as defined in that review, includes conditions that carry a high degree of clinical uncertainty, may require high care dependency because of decreased function, but may not be advanced (7). The review concluded that PCC (through valuing the social needs of patients, promoting quality of life, and reform of health structures) will improve patients' experience of interaction with healthcare systems (6). The review also concluded that primary data are needed that investigate the meaning and practice of PCC in a diverse diagnostic groups and settings (6). Re-engineering health systems to deliver PCC has particular relevance to low- and middle-income countries (LMIC) (8, 9). Serious health-related suffering places a huge burden on health systems, with the greatest burden in LMIC. Projections from WHO mortality data estimate that low-income countries face the largest proportional increase, largely due to ageing (155% increase in people with serious health related suffering in the last year of life by 2060 to 5.14 million people) (10). In such contexts, serious illness also places huge psychological, social, economic, physical, and spiritual burdens on patients and (largely

For peer review only - http://bmjopen.bmj.com/site/about/guidelines.xhtml

female) family caregivers. (11-13). It carries a high risk of mortality, negatively impacts quality of life and daily function, and is burdensome in symptoms, treatments and or caregiver stress (14).

PCC has great potential for patients, families, staff and the health care system in terms of engagement, enablement, management of symptoms and reduction in re-referrals, reducing readmission, frequent visits to primary care and/or emergency visits (15). Identification, refinement, adaptation, and implementation of effective PCC interventions may thus help to achieve the WHO and Universal Health Coverage goals. However, no review to date has aimed to identify and synthesise the evidence for the outcomes and cost of PCC across serious physical illness. We aimed to review the evidence (in terms of outcomes and costs) for interventions that aim to deliver person-centred care to, or enhance person-centredness of care for, adults with serious physical illness.

Methods

Design

Systematic review of peer-reviewed literature drawing on PRISMA guidelines, with quality appraisal using the Joanna Briggs Institute critical appraisal checklist, and narrative synthesis of findings. A full protocol is registered with PROSPERO, CRD42018108302 (16).

Objectives

The objectives of this review were to i) identify models of person-centred care interventions for adults with serious illness; ii) determine which outcomes have been measured as endpoints; iii) appraise intervention effectiveness in terms of outcomes and costs, using narrative synthesis; iv) evaluate the quality of the evidence.

Search strategy

The following databases were searched in January 2020: Amed, Assian, Cumulative Index to Nursing and Allied Health Literature (CINAHL), Cochrane Library, Embase, Medline,

For peer review only

PsycInfo, Scopus and Web of Science. Key journals and reference lists from included studies and relevant review articles were hand searched.

The search strategy (Table 1) was developed in consultation with an information specialist. We used the following key concepts, drawing on our prior review of the concepts and primary data underpinning person-centred care (6): person/patient-centred care, family-centred care, family-based care, individualised care, holistic care, value-based care. Data bases were searched from inception.

Table 1: Search strategy

Search strategy number	Key concepts	Key words
1	Patient centred<br>Family centred<br>Person centred<br>Individualised<br>Holistic<br>Value based	Patient-centered care or patient-centred care or client-centred care or client-centered care or client-focused care or person-centred care or person-centered care or person-focused care or family-centred care or family-focused care or family-centered care or individuali?ed or holistic care or holistic health or value based care
2	Serious illness<br>Chronic illness<br>Long term illness	chronic diseases or serious illness or chronic illness or long term conditions or long term illness

Subject headings and word truncations were entered according to requirements of each database to map all potential keywords. Group 1 concepts were combined using the 'OR' function. Likewise group 2 concepts were combined using OR function. Finally search strategies 1 and 2 were intersected using the 'AND' function

For peer review only - http://bmjopen.bmj.com/site/about/guidelines.xhtml

Eligibility criteria

The inclusion and exclusion criteria are summarised in table 2 below:

Table 2: Inclusion and exclusion criteria

	**Inclusion**	**Exclusion**
Participants	All serious physical illness as defined by Kelly et al 2014; 2016: *Serious illness is a health condition that carries a high risk of mortality AND either negatively impacts a person's daily function or quality of life, OR excessively strains their caregivers.*<br><br>Caregivers of patients with serious physical illness defined above.<br><br>Health care professionals (doctors, nurses, social workers etc) caring for patients with serious physical illness.	Patients with conditions considered risk factors to develop serious illness such as hypertension.
Interventions	Any interventions delivered using a person-centred approach such as involving patients in decision-making about their care, setting goals and plans, patient being involved managing their own disease, interventions focused on the whole person, holistic approach. Interventions delivered in any format that is focused on the needs of the patients.	Any interventions delivered without patient involvement or decision making about their care or helping them take actions to support themselves.
Studies and comparator	Published intervention studies<br>Written in English language only<br>Evaluations using a comparator.<br>The comparison group should either be usual care/standard care, or a	Unpublished studies, studies not written in English language, conference proceedings, conference abstracts,<br>Non-randomised trials

For peer review only

	comparator intervention.	No comparison group.
Outcomes	Patient and family caregiver self-report outcomes, e.g.:<br>-pain and symptom prevalence and intensity/severity, interference with daily activities, knowledge and practice of self-management, quality of life;<br>-psychosocial outcomes such as stress, anxiety, depression, burnout, distress.<br>-social, practical, and spiritual;<br>knowledge of pain and/or symptom management, quality of life, psychological outcomes (anxiety, stress, depression, distress) and caregiver motivation to provide care.<br><br>Formal and informal health service use<br>Costs of services.	Outcomes not related to person-centred care (outcomes not focusing on physical, psychological social and spiritual aspects of care).

Selection of studies, data collection and management

We report the search strategy process using the PRISMA flow chart (17). All references identified by the search strategy were exported to Endnote software and deduplicated. One reviewer (KN) independently appraised all titles and abstracts against the inclusion and exclusion criteria. If the title and abstract was obviously irrelevant, the reference was excluded at this stage. Full text retained references were obtained and appraised against inclusion and exclusion criteria, and if the decision was unclear this was discussed with a second reviewer (AC) and if necessary adjudicated by a third (RH).

For peer review only

Data extraction

KN and AC extracted study data using methods described in the Cochrane handbook for systematic reviews of interventional studies (18). A standardised data extraction form was used to ensure consistency in the review (19). AC extracted n=26 papers and KN extracted n=44 papers, then both authors peer reviewed data extraction. Any queries were resolved through discussion. RH reviewed the final data extraction.

The following variables were extracted: authors, year of publication, aims and objectives, setting and country, study design, selection of participants, sample characteristics, randomisation procedures, blinding of participants and outcome assessors, assessment of outcomes and measures used, description of the intervention and comparison group, intervention delivery, sample size, data analysis, loss to follow-up, findings for outcomes and costs, and study conclusions. We have summarised data extraction in table 4.

Assessment of methodological quality of the studies

We applied the Joanna Briggs Institute Critical Appraisal checklist for Randomised and non-Randomised trials to assess methodological quality of the studies (20). These are summarised in table 3. This was conducted at individual study level. AC and KN assessed each study independently, and thereafter discussed critical appraisal. Discrepancies in the assessment of quality between AC and KN were resolved by discussion, and RH checked the critical appraisals of the papers.

Synthesis of the evidence

Due to heterogeneity of the studies, interventions, participants, and outcomes a meta-synthesis was not conducted. We therefore present a narrative synthesis of the studies included in the review. We performed narrative synthesis to synthesize the findings of the different studies.

For peer review only

We conducted narrative synthesis using the Guidance on the Conduct of Narrative Synthesis in Systematic Reviews (21).

We developed two a logic models (Figures 2 and 3) to summarise the context, study population, to describe the intervention components, mechanism of action, and outcomes. Figure 2 contains studies which reported a theory or conceptual framework which informed the development of the intervention. Figure 3 reports studies which did not state a theory or conceptual framework of the intervention.

A preliminary synthesis was undertaken in form of a thematic analysis involving listing and presenting results in tabular form. The results of the included studies were summarised in a narrative synthesis within a framework (participants, study aims, intervention description, usual care description, outcomes and measures used as presented on table 4. For each study the effects of the intervention on the outcomes tested in provided.

We explored relationships in the data, for example similar study design use (RCT vs non-RCT), similar methods of randomisation, similar intervention components and mode of delivery and similar outcomes. We then made conclusions based on the robustness of the synthesis and the quality of evidence.

Patient and public involvement

Patient and public involvement was not conducted as part of this review.

For peer review only

Table 3: Risk of Bias in the studies (Joanna Briggs Institute Critical Appraisal checklist)

Yes: means good and no risk of bias, No: means there was risk of bias, ITTA: Intention to treat analysis, IG: Intervention group, CG: control group

	Author	Random allocation	Allocation concealment	Baseline similarity/Comparable at entry	Blinding of participants	Blinding of those delivering treatment	Blinding of outcome assessors	Identical except intervention	Relatively complete follow-up achieved	Participants analysed in the groups originally randomised	Outcomes measures same between group	Outcomes assessed in reliable way	Statistical analysis appropriate	Total YES scores
1	de Batlle, 2020	Unclear	Unclear	No	No	Unclear	Unclear	Yes	Yes, 87%	Yes, Modified ITTA	Yes	Yes	Yes	6/12
2	Mielenz et al 2020	Yes	Unclear	Yes	No	No	Yes	Yes	Yes, 97%	Yes, Modified	Yes	Yes	Yes	9/12
3	Yu et al 2020	Yes	Unclear	Yes	No	No	N/A	Yes	No, 71%	Yes, Modified ITTA	Yes	Yes	Yes	7/12
4	Bergsten et al 2019	Yes	Unclear	No	No	No	Yes	Yes	Yes, 83%	Yes	Yes	Yes	Yes	9/12
5	Berntsen et al (2019)	No	N/A	No	No	No	Unclear	Yes	Unclear	Yes	Yes	Yes	Yes	5/12
6	Berondonk (2019)	Yes	Yes	Yes	No	No	Unclear	Yes	Yes, 82%	Yes, Modified ITTA	Yes	Yes	Yes	9/12
7	Bokberg et al (2019)	No	N/A	No	No	No	Yes	Yes	No	Yes, Modified ITTA	Yes	Yes	Yes	6/12
8	Britt et al (2019)	No, patients recruited at separate hospitals. Those declining participation in IG were offered inclusion in CG	No	CG were younger, more likely to be married and more likely to live at home. CG were more likely to have a cardiovascular, and less likely to have dementia as a primary diagnosis.	No	No	No	Yes	No, 51% completed 12 months	Unclear	Yes	Yes	Yes	4/12

For peer review only

	Author	Random allocation	Allocation concealment	Baseline similarity/Comparable at entry	Blinding of participants	Blinding of those delivering treatment	Blinding of outcome assessors	Identical except intervention	Relatively complete follow-up achieved	Participants analysed in the groups originally randomised	Outcomes measures same between group	Outcomes assessed in reliable way	Statistical analysis appropriate	Total YES scores
9	Hedman, et al 2019 Bertilsson et al (2016), Guidenti et al (2015) and Bertilsson et al (2014) One study reporting three papers	Unclear	Unclear	Unclear	No	No	Unclear	Yes	Yes, 81%	Yes, Modified ITTA	Yes	Yes	Yes	6/12
10	Ohlen et al (2019)	No	N/A	Yes	No	No	N/A	Yes	Yes, 82%	Yes, ITTA	Yes	Yes	Yes	6/12
11	Pirhonen et al 2019 and Pirhonen et al 2016 Two papers one study	Yes	Not clear	Yes	No	No	Not clear	Yes	Yes, 91%	Yes	Yes	Yes	Yes	8/12
12	Zakrisson et al (2019)	Yes	Yes	No	No	No	Yes	Yes	No	Yes	Yes	Yes	Yes	8/12
13	Arian et al (2018)	Yes	Unclear	Yes	No	No	Yes	Yes	No	Yes	Yes	Yes	Yes	8/12
14	Eggers, 2018	Yes	Yes	Yes	No	No	Unclear	Yes	Yes, 86%	Yes, Modified ITTA	Yes	Yes	Yes	9/12
15	Fors et al (2018)	Yes	Not clear	Yes	No	No	N/A: patients self-completed and sent by post	Yes	Yes, 91%	Yes	Yes	Yes	Yes	8/12
16	Reed et al 2018)	Yes	Yes	Yes	Yes	No	Yes	Yes	Yes, 91%	Yes	Yes	Yes	Yes	11/12

For peer review only - http://bmjopen.bmj.com/site/about/guidelines.xhtml

	Author	Random allocation	Allocation concealment	Baseline similarity/Comparable at entry	Blinding of participants	Blinding of those delivering treatment	Blinding of outcome assessors	Identical except intervention	Relatively complete follow-up achieved	Participants analysed in the groups originally randomised	Outcomes measures same between group	Outcomes assessed in reliable way	Statistical analysis appropriate	Total YES scores
17	Schäfer et al (2018)	Yes	Yes	No difference in characteristics of GPs and practices between groups. More female patients in the control group.	No	No	No	Yes	Yes, 93% completed	Yes, Modified ITTA	IG data collected at baseline and after intervention (mean 441 ±66) CG data collected at baseline and 14 months (mean 376 ±27 days).	Yes	Yes	7/12
18	Armstrong et al (2017)	Unclear	Yes	Yes	No	No	Unclear	Yes	Yes, 93%	Yes	Yes	Yes	Yes	8/12
19	Feldthusen et al 2017	Yes	Yes	Yes	No	Yes	Yes	Yes	Yes, 96%	Yes	Yes	Yes	Yes	11/12
20	Fors et al (2017); Fors et al (2016a)a Fors et al (2016b) Wolf et al 2016 and Fors et al 2015 One study reporting five papers	Yes	Yes	Yes	No	No	Unclear	Yes	Yes, 95%	Yes, Modified ITTA	Yes	Yes	Yes	9/12
21	Hansson et al 2017<br><br>Gyllensten et al 2019	Yes	Yes	No	No	No	No	Yes	Yes, 92%	Yes	Yes	Yes	Yes	8/12
22	Ko et al (2017)	Yes	Yes	IG had higher FEV_1% of predicted	No	No	Yes	Yes	No, 79%	Yes	Yes	Yes	Yes	8/12
23	Low et al (2017)	Yes	Yes	Yes	No	No	Yes	Yes	Yes, 87%	Yes	Yes	Yes	Yes	10/12

For peer review only

	Author	Random allocation	Allocation concealment	Baseline similarity/Comparable at entry	Blinding of participants	Blinding of those delivering treatment	Blinding of outcome assessors	Identical except intervention	Relatively complete follow-up achieved	Participants analysed in the groups originally randomised	Outcomes measures same between group	Outcomes assessed in reliable way	Statistical analysis appropriate	Total YES scores
24	Wichit et al (2017)	Yes	Yes	IG were older	Yes	No	Yes	Yes	Yes, 96%	Yes	Yes	Yes	Yes	10/12
25	Ericsson et al 2016 Larsson et al 2015<br><br>Two papers one study	Yes	Yes	Yes	No	No	Yes	Yes	No, 70%	Yes, ITTA	Yes	Yes	Yes	9/12
26	Hansson et al 2016; Ulin et al 2016; Ekman et al (2012) and Dudas et al 2012 Four papers one study	No	N/A	No	No	No	Unclear	Yes	Yes, 80%	Yes, ITTA	Yes	Yes	Yes	6/12
27	Jutterström et al (2016)	Yes	Yes	No, Greater numbers in the Group intervention group. Group intervention had greater HbA1c than External control group, and lower total cholesterol than internal control. External control group were more likely to have diet and/or insulin treatment	No	No	No	Yes	Yes, 88%	Yes, Modified ITTA	Yes	Yes	Yes	8/12

For peer review only

	Author	Random allocation	Allocation concealment	Baseline similarity/Comparable at entry	Blinding of participants	Blinding of those delivering treatment	Blinding of outcome assessors	Identical except intervention	Relatively complete follow-up achieved	Participants analysed in the groups originally randomised	Outcomes measures same between group	Outcomes assessed in reliable way	Statistical analysis appropriate	Total YES scores
28	Olsson et al 2016 and<br><br>Olsson et al 2014<br><br>Two papers one study	No	N/A	No	No	No	Unclear	Yes	Yes, 99%	Yes	Yes	Yes	Yes	6/12
29	Or and Tao (2016)	Yes	Yes	Yes	No	No	No	Yes	Yes, 87%	Yes, Modified ITTA	No, IG self monitoring data captured on a tablet, CG from log book records.	Yes	Yes	8/12
30	Sahlen et al (2016); Brännstrom & Boman (2014) One study two papers	Unclear	Unclear	IG older	No	No	Unclear	Yes	Yes, 83%	Yes, Modified ITTA	Yes	Yes	Yes	6/12
31	Slok et al (2016)	Yes	Yes	IG more likely to be a current smoker	No	No	No	Yes	Yes, 82%	Yes, Modified ITTA	Yes	Yes	Yes	8/12
32	Windrum et al (2016)	Unclear	Unclear	Yes	No	Yes	Unclear	Yes	Unclear	Unclear	Unclear	Unclear	Yes	4/12
33	Yu (2016)	Yes	Unclear	Yes	No	No	Yes	Yes	Yes	Yes, Modified ITTA	Yes	Yes	Yes	9/12
34	Hernández et al (2015)	Yes	Yes	CG more likely to have had influenza and	No	No	Yes	Yes	No, 71%	Yes, Modified ITTA	Yes	Yes	Yes	8/12

For peer review only

	Author	Random allocation	Allocation concealment	Baseline similarity/Comparable at entry	Blinding of participants	Blinding of those delivering treatment	Blinding of outcome assessors	Identical except intervention	Relatively complete follow-up achieved	Participants analysed in the groups originally randomised	Outcomes measures same between group	Outcomes assessed in reliable way	Statistical analysis appropriate	Total YES scores
				pneumococcal vaccines										
35	Kikkenborg et al (2015)	Unclear	Unclear	Yes	No	No	Yes	Yes	Yes, 84%	Yes, Modified ITTA	Yes	Yes	Yes	8/12
36	Larsson et al (2015) and 2013 One study two papers	Yes	Yes	Yes	No	No	No	Yes	Yes	Yes	Yes	Yes	Yes	9/12
37	Lowther et al (2015)	Yes	Yes	CG had been diagnosed with HIV for longer and been taking ART for longer than IG.	No	No	No	Yes	Yes, 95%	Yes, Modified ITTA	Yes	Unclear	Yes	7/12
38	Kelechi et al (2014)	Yes	Not clear	Greater motivation in IG Patient baseline demographic characteristics not reported by group	No	No	Not clear	Yes	Yes, 88%	Yes, Modified ITTA	Yes	Not stated	Yes	6/12
39	Young et al (2013)	Yes	Yes	IG more likely to have private health insurance, were admitted to a private hospital and had a stoma created.	No	No	Unclear	Yes	Yes, 88%	Yes, Modified ITTA	Yes	Yes	Yes	8/12
40	Chochinov et al (2011)	Yes	Yes	Yes	No	No	Yes	Yes	No, 74%	Yes, Modified ITTA	Yes	Unclear	Yes	8/12
41	Goelz et al (2011)	Yes	Yes	No	No	Unclear	Unclear	Yes	Yes	Yes	Yes	Yes	Yes	8/12

For peer review only - http://bmjopen.bmj.com/site/about/guidelines.xhtml

	Author	Random allocation	Allocation concealment	Baseline similarity/Comparable at entry	Blinding of participants	Blinding of those delivering treatment	Blinding of outcome assessors	Identical except intervention	Relatively complete follow-up achieved	Participants analysed in the groups originally randomised	Outcomes measures same between group	Outcomes assessed in reliable way	Statistical analysis appropriate	Total YES scores
42	Murphy et al (2010)	Yes	Unclear	Yes	No	No	Yes	Yes	No, 74%	Yes, Modified ITTA	Yes	Unclear	Yes	7/12
43	Wolff et al (2010)	Unclear, Cluster randomization	Unclear	CG more likely to be female and less educated	Unclear	No	Yes	Yes	No, 69% of patients and 64% of caregivers	Yes, Modified ITTA	Yes	Yes	Yes	6/12
44	Dobscha et al (2009)	Unclear	Yes	Yes	Yes	Yes	Yes	Yes	Yes, 90%	Yes, ITTA	Yes	Yes	Yes	11/12
45	Machado et al (2007)	Yes	Yes	IG had longer duration of symptoms, more likely to be female and more likely to not be working due to lower back pain	No	No	Yes	Yes	Yes, 81%	Yes, Modified ITTA	Yes	Yes	Yes	9/12
46	Glasgow et al 2005	Yes	Yes	Yes	No	No	unclear	Yes	Yes, 83%	Yes	Yes	Yes	Yes	9/12
47	Mills and Harvey (2003)	Unclear	Unclear	No	No	No	Unclear	Yes	No	Yes, modified ITTA	Yes	Yes	Yes	5/12
48	Kennedy et al (2004)	Unclear	Unclear	IG more likely to be off work with long term sickness	No	No	Unclear	Differences in discharge policies between centres.	Yes, 87%	Yes, ITTA	Yes	Unclear	Yes	4/12
49	Martin et al, (2004),	Unclear	Unclear	CG were more likely to be male and have greater cigarette consumption.	No	No	Unclear	Yes	Yes, 83%	Yes, Modified ITTA	Yes	Yes	Yes	6/12
50	Alamo et al (2002)	Unclear	Unclear	IG more tender points and more likely to describe pain as never/hardly	No	No	Yes	Yes	No, 74%	Yes	Yes	Yes	Yes	6/12

For peer review only

	Author	Random allocation	Allocation concealment	Baseline similarity/Comparable at entry	Blinding of participants	Blinding of those delivering treatment	Blinding of outcome assessors	Identical except intervention	Relatively complete follow-up achieved	Participants analysed in the groups originally randomised	Outcomes measures same between group	Outcomes assessed in reliable way	Statistical analysis appropriate	Total YES scores
				ever a problem.										
51	Sommers et al (2000)	Yes	Unclear	No	No	No	Unclear	Yes	No	Yes, Modified ITTA	Yes	Yes	Yes	6/12
52	Gustafson et al (1998)	Yes	Yes	Yes	No	No	Unclear	Yes	Yes, 84%	Yes, Modified ITTA	Yes	Unclear	Yes	8/12
53	Kinmonth et al (1998)	Yes	Yes	Yes	Unclear	No	Unclear	Yes	No, 69%	Yes, Modified ITTA	Yes	Yes	Yes	8/12
54	Landefeld et al 1995	Yes	Unclear	Yes	No	No	No	Yes	Yes	Yes, Modified ITTA	Yes	Yes	Yes	8/12

For peer review only

Results

The PRISMA flow diagram (figure 1) below presents the results of the search strategy. After deduplication, we screened n=4796 papers (title, abstract) and n=91 papers were retained for full text screening. Of these, n=21 were excluded (reasons are reported in the flow chart) and n=70 papers (reporting 54 different studies) were retained in the review.

For peer review only

For peer review only - http://bmjopen.bmj.com/site/about/guidelines.xhtml

Table 4: Characteristics of studies included in the review N=54

Study Number	Author & Year/ Country	Aim Design Theoretical model	Sample	Intervention(s)	Outcomes/measures and follow-up period	Results
1	de Batlle, 2020<br><br>Spain	To assess the effectiveness and cost-effectiveness of the implementation of a mobile health (mHealth)-enabled integrated care model for complex chronic patients.<br><br>a prospective, pragmatic, two-arm, parallel implementation trial	Elderly patients with COPD, heart failure and caregivers<br><br>N=52 integrated care model, mean age (SD): 82(7)<br><br>n=35 usual care, mean age (SD): 82(8).	The combined benefits of the CONNECARE (Personalised Connected Care for Complex Chronic Patients) organizational integrated care model and the eHealth platform supporting it, consisting of a (i) self-management app, with status and performance reports, a virtual coach with customizable automated feedback, and full communication with the care team; (ii) a Fitbit Flex 2 digital activity tracker and any additional sensor deemed necessary by the care team including a digital pulse-oximeter, digital scale, and digital blood pressure monitor, that were fully integrated into the self-management app; (iii)	1. Quality of life (changes in health status): 12-Item Short-Form Survey (SF-12), Barthel index for Activities of Daily Living and, Hospital Anxiety and Depression scale<br><br>2. Use of health care resources and estimated associated costs based on Catalan Health Department official data: Unplanned visits and admission<br><br>3. cost-effectiveness, based on the improvement in QoL relative to costs, assessed by means of the incremental cost-	1. No significant differences between the two groups (mean change (SD) 5.0 (5.2) p= .10<br><br>2.Unplanned visits were significantly lower in the intervention group (2.3 (3.1) vs 1.0 (1.1) P=0.004).<br><br>3. The integrated care program generated savings from US $584 to $1434 per patient, depending on the scenarios. The integrated care program

For peer review only

Study Number	Author & Year/ Country	Aim Design Theoretical model	Sample	Intervention(s)	Outcomes/measures and follow-up period	Results
				a patient profile in the SACM (Smart Adaptive Case Management) web-based platform, accessible to all members of the care team (family physicians, hospital specialists, and social workers), that was used for coordination and communication among professionals in the different settings, and to contact the patient when needed; and (iv) assignment of a case manager in charge of supervising the whole process and serving as the main patient contact point.<br><br>Control group received usual care (details not provided).	effectiveness ratio (ICER);<br><br>Data collected at baseline and a 6-month follow up,	was cost-effective according to the ICER, performing better in terms of QoL while reducing overall expenses
2	Mielenz et al 2020<br><br>USA	To evaluate the Self-management Resource Center Small Group Programs (SMRCSGP), plus wellness coaching, as a booster intervention in older	Elderly people >55 years old.<br>N=125<br>Intervention n=62, mean age (SD) 72 (0.94)	The intervention: The wellness self-coaching program asked participants to create a "Wellness Vision," wherein the participants set monthly and weekly behavioural goals	Primary outcomes<br>1. Physical activity: The Community Health Activities Model Program for Seniors (CHAMPS) was used to collect	Across the 6 months of our study the intervention and control groups did not vary significantly on any primary physical activity outcomes of interest (CHAMPS and

For peer review only - http://bmjopen.bmj.com/site/about/guidelines.xhtml

Study Number	Author & Year/ Country	Aim Design Theoretical model	Sample	Intervention(s)	Outcomes/measures and follow-up period	Results
		adults with chronic diseases.<br><br>To evaluate the role of personal health records (PHR) prototype as the linkage between the clinic and community.<br><br>RCT<br><br>Self-efficacy theory	Control n=63, mean age (SD) 73.1 (0.95)	that were agreed upon by participant and coach. Class lesson titles were as follows: taming frenzy, self-compassion, focus, mindfulness, strengths (two-part), motivation, legacy, creativity (two-part), body intelligence (two-part), relationships (two-part), positivity (two-part), meaning (two-part), curiosity (two-part), standard setter (two-part), self-leadership, and your plan to thrive.<br><br>Control: Both groups received usual care consisting of self-management Resource Center Small Group Programs (SMRCSGP) (including programs on general chronic disease and specific conditions: arthritis, diabetes, HIV, chronic pain, and cancer) are structured wellness interventions that encourage self-management in older	information on physical activity.<br>-Frequency per week of all exercise-related activities<br>-Hours per week of all exercise-related activities<br><br>2.Behavioral Risk Factor Surveillance System physical activity measures<br>-Met aerobic physical activity guidelines,<br>-Met aerobic and muscle strengthening guidelines,<br><br>Secondary outcomes:<br>3. Patient-Reported Outcomes Measurement Information System (PROMIS) v1.0 short form (SF) measures:<br><br>Depressin: Emotional Distress-Depression—SF<br><br>Fatigue: Fatigue—SF 4a,	BRFSS measures) in models.<br><br>The intervention and control groups did vary significantly (p = .03) over time on one secondary outcome: the PROMIS physical function variable. Although both groups reported improvements on this measure over time (higher scores indicating that participants can do more and feel better), overall improvement was greater for the wellness coaching intervention group (2.6) than for the control (0.6).

Study Number	Author & Year/<br><br>Country	Aim<br>Design<br>Theoretical model	Sample	Intervention(s)	Outcomes/measures and follow-up period	Results
				adults living with chronic conditions and are implemented by lay leaders	Pain behaviour: Pain Behavior—SF 7a,<br><br>Pain intensity: Pain Intensity—SF 3a,<br><br>Pain interference: Pain Interference—SF 4a,<br><br>Physical function: Physical Function—SF20a),<br><br>Sleep: Sleep Disturbance—SF 4a.<br><br>4. Medical care questions:<br>- Times visiting a physician<br>- Times visiting a hospital emergency department<br>- Times hospitalized for one night or longer<br>- Total nights spent in the hospital<br><br>-Self-efficacy	

For peer review only

Study Number	Author & Year/<br><br>Country	Aim<br>Design<br>Theoretical model	Sample	Intervention(s)	Outcomes/measures and follow-up period	Results
					for exercise was assessed on the Resnick Self- Efficacy for Exercise (SEE)<br>-Falls in the past month<br><br>CHAMPS<br>data were collected at baseline, 3 months, and 6 months.	
3	Yu et al 2020<br><br>Canada	To assess the impact of 'MyDiabetesPlan' on decisional conflict, diabetes distress, health-related quality of life, and patient assessment of chronic illness care at the individual patient level.<br><br><br>Cluster RCT	N=102 patients<br>n=29 clinicians<br><br>N=111 patients<br>n=24 clinicians	A web-based PtDA in which patients populate their cardiometabolic and psychosocial profiles and general care priorities: MyDiabetesPlan then generates individualized diabetes-specific goals and strategies based on these inputs that the patients then select, resulting in an action plan.<br>Clinicians at intervention sites underwent a one-on-one 60-min tutorial in their clinic room by the research coordinator, with access to a one-page how-to guide and 2-min	Primary outcome:<br>1. Decisional conflict: the Decisional Conflict Scale (DCS),<br><br>Secondary outcomes:<br>2. Diabetes distress: Diabetes Distress Scale (DSS)<br><br><br>3., Health-related quality of life: SF-12<br><br>4. Chronic illness care: PACIC (Patient Assessment of Chronic Illness Care) Scale<br><br>5. intention to engage in	1. No significant differences between the two groups; mean 0.5; p=0.08<br><br>2. mean change 0.2 p=0.12<br><br><br><br>3. mean change 1.2 p=0.57<br><br>4. Mean change 0.15 p<0.001

For peer review only - http://bmjopen.bmj.com/site/about/guidelines.xhtml

Study Number	Author & Year/ Country	Aim Design Theoretical model	Sample	Intervention(s)	Outcomes/measures and follow-up period	Results
				video. During subsequent clinical encounters, a member of the interprofessional team (nurse or dietitian) logged into MyDiabetesPlan and completed it with the patient; the physician subsequently reviewed the resultant action plan with the patient. At 6 months, patients at intervention sites were provided with a patient-directed how-to guide and video and directed to update MyDiabetesPlan according to their progress before the appointment.<br><br>Clinicians in the control sites received paper copies of the executive summary of the Diabetes Canada clinical practice guidelines, and a postcard outlining web-based clinical information resources. After 6 months, patients in the control sites received a	IPSDM (Interprofessional Shared Decision-Making): CPD (Continuing Professional Development.) Reaction Questionnaire<br><br>Outcomes were assessed at the individual participant level, at baseline, and at 6 months and 12 months (after an appointment) through a web-based survey or by mail.	5. No significant differences between two groups.

For peer review only - http://bmjopen.bmj.com/site/about/guidelines.xhtml

Study Number	Author & Year/ Country	Aim Design Theoretical model	Sample	Intervention(s)	Outcomes/measures and follow-up period	Results
				Diabetes Canada patient education pamphlet regarding diabetes self-management and a postcard outlining web-based additional patient resources.		
4	Bergsten et al 2019 (22)<br><br>Sweden	To evaluate the effect of a nurse-led clinic with frequent visits, treat-to-target and person-centred care of patients with rheumatoid arthritis and moderate-to-high disease activity compared with patients receiving regular care.<br><br>RCT<br><br>Gothenburg PCC	N=70 patients with moderate to severe symptoms.<br><br>n=36 intervention group, mean age 60.3 (SD 15.9), n=34 control group, mean age 62.4 (SD 12.2).	4 nurses attended 2 days' training on principles, philosophy, and delivery of person-centred care. An individual health plan agreed by patient and nurse, including aims for disease activity and participation, tools to achieve these goals.<br><br>Patients in the control group were offered a telephone appointment with their regular physician, in order to discuss their disease activity and whether a physical appointment, and potentially a change in therapy, should be made. All patients were then followed by their treating physician according to	(1) Primary outcome was the difference in the DAS28 change: DAS28 is an index based on the number of tender and swollen joints, patients' global health assessment and the erythrocyte sedimentation rate.<br><br>Secondary outcomes:<br><br>(2) the proportions with minimal clinical important improvement in DAS28 (>0.6)<br><br>(3) the proportions achieving low disease activity (DAS28 <3.2);	In the PP analyses, the primary outcome (i.e., the difference in delta-DAS28 between the IG and CG) was not statistically significant (0.43; 95% CI −0.27, 1.13)<br><br>NS difference in ITT primary PCC in DAS 26 (mean (95% CI)): 1.39 (0.97 to 1.82) v control 1.04 (0.54 to 1.53).<br><br>In PP PCC 1.50 (1.00 to 2.00) v control 1.07 (0.56 to 1.57). Trial inclusion

For peer review only - http://bmjopen.bmj.com/site/about/guidelines.xhtml

Study Number	Author & Year/ Country	Aim Design Theoretical model	Sample	Intervention(s)	Outcomes/measures and follow-up period	Results
				regular care, with follow-up visits decided either at this telephone appointment or according to previous plans. In regular care, the patients usually visited the clinic every 6–12 months. As part of regular care, patients also had the possibility of making appointments with the physician in the event of flares.	(4) the proportions achieving a EULAR moderate or good response<br><br>(5) the Health Assessment Questionnaire score, measuring daily function<br><br>(6) the RA impact of disease (RAID) score, measuring the impact of RA from the patient's perspective;<br><br>(7) Patient Acceptable Symptom State (PASS) score (8) the Beliefs about Medicines Questionnaire (BMQ) responses, measuring patients' attitude to medication split in two domains (BMQ-necessity, BMQ-concerns)<br><br>(9) the EuroQol-5D (EQ-5D) score).	terminated because more patients in the interventions dropped out

For peer review only

Study Number	Author & Year/ Country	Aim Design Theoretical model	Sample	Intervention(s)	Outcomes/measures and follow-up period	Results
5	Berntsen et al (2019)(23)<br><br>Norway	To determine if the Patient-Centred Team Intervention (PACT) causes reduced use of high-level emergency care and increased use of low-level planned care with unchanged mortality risk for the multi-morbid elderly<br><br>Parallel arm study	N=1218 patients >60 years, with multi-morbidity, complex long-term needs and high short-term risk for emergency hospital admission n=439 intervention group, referred to the PACT team. Mean age 80.02 (SD8.72) n=779 control group, mean age 78.8 years (SD 8.68). Patients had an emergency admission but not received PACT intervention. A matched local and distant control was sought for each intervention participant.	Intervention: Patient is assigned to a mini-team of nurse co-ordinator, physician, physiotherapist, occupational therapist and pharmacist. They work with the patient to explore goals using a person-centred approach including a comprehensive geriatric assessment methodology. The team address immediate clinical needs and co-ordinate Average intervention time 30 days.<br><br>Control group: usual care defined as evidence-based care for the cause of the emergency admission to hospital, referral for other diagnoses to GP or specialist care and standard electronic communication.	1. Number of emergency admissions<br><br>2. Sum of emergency inpatient bed days<br><br>3. Count of emergency re-admissions within 30 days of discharge<br><br>4. Count of planned out-patient visits<br><br>5. Count of emergency outpatient visits<br><br>6. Mortality risk at 3 and 6 months follow-up Follow up began at first referral to PACT (IG) or time of emergency admission (CG) and ended after 6 months or death.	1. Adjusted RR 0.90 (95%CI: 0.82-0.99)<br><br>2. Adjusted RR 0.68 (95%CI 0.52-0.79)<br><br>3. Adjusted RR 0.72 (95%CI 0.41-1.24)<br><br>4. Adjusted RR 2.27 (95%CI 2.02-2.55)<br><br>5. Adjusted RR 0.90 (95%CI 0.68-1.2)<br><br>6. Adjusted RR 0.39 (95%CI 0.22-0.7) at 3 months and 0.57 (95%CI 0.34-0.94) at 6 months.

For peer review only - http://bmjopen.bmj.com/site/about/guidelines.xhtml

Study Number	Author & Year/<br><br>Country	Aim<br>Design<br>Theoretical model	Sample	Intervention(s)	Outcomes/measures and follow-up period	Results
6	Berendonk (2019) (24)<br><br>Germany.	To test the feasibility of a nursing intervention (DEMIAN) in routine care and its effects on care providers' job satisfaction, motivation and work strain.<br><br>Pragmatic two-group cluster RCT	N=20 German long-term care facilities<br>n= 84 care providers (mean age 41.8, SD 10.2) and 42 residents with dementia in intervention group<br>n= 96 care providers (mean age 38.5, SD 11.9) and 42 residents with dementia in control group	Intervention: Registered nurses completed two days of training within a two week period on the DEMIAN intervention. Its objectives are to gather information on meaningful situations for each individual and to use this knowledge to plan and provide care. There was a 6 week implementation phase after training to carry out mini-interventions. Nurses encourages all team members, relatives and volunteers to be involved in the interventions.<br><br>Control: usual care (details not provided).	1. Screening instrument for job strain in human service work (BHD)<br><br>2. Modified Task and Job Analysis Tool- residential LTC version (TAA-A)<br>Baseline assessment and at post intervention follow up	1. Greater job satisfaction in IG than CG post intervention (p=0.053)<br><br>2. Most TAA-A outcomes did not differ significantly between IG and CG after intervention. Time pressure did decrease in IG compared to CG (p=0.026)
7	Bökberg et al (2019) (25)<br><br>Sweden	To evaluate whether an educational intervention had any effect in the staff's perception of providing person-centred palliative care for older persons in nursing homes.	N=365 nursing home staff (nurses, assistant nurses, physiotherapists, occupational therapists, social workers and unit managers) recruited from 20	Intervention: A knowledge-based palliative care intervention consisting of five 2h educational seminars for nursing home staff based on Swedish national documents on the key principles of palliative care intending to improve quality of life for individuals	1. Person-centred Care Assessment Tool (P-CAT)	1. No significant change in total P-CAT score pre- and post intervention in IG (p=0.715)or CG (p=0.601)<br>No statistically significant changes in pre and psot intervention scores on any subscale for either group.

Study Number	Author & Year/ Country	Aim Design Theoretical model	Sample	Intervention(s)	Outcomes/measures and follow-up period	Results
		Pre- and post-test experimental design.	urban and rural, small (<25 residents) and large (>100 residents) nursing homes in two Swedish counties n=167 intervention group, median age 47 n=198 control group, median age 49 years	and their families. Participants were provided with a study booklet. The intervention was implemented over 6 months.<br><br>Control: usual training. None of the participating homes had had workplace education or training in palliative care before the intervention.	2. Person-Centred Climate Questionnaire (PCQ-S)<br><br>Data collected at baseline and post-intervention	2. No significant change in total PCQ-S scores pre and post intervention in IG (p=0.685) or CG (p+0.451) No statistically significant changes in pre and post intervention scores on any subscale for either group.
8	Britt et al (2019) (26)<br><br>USA	To assess the effect of the LifeCourse (LC) programme on healthcare utilisations<br><br>Quasi-experimental trial	N=903 patients estimated to be within 3 years of end of life with 1+ serious illness n=450 intervention, mean age 78.1 (SD 12.0) n= 453 control, mean age 74.3 (SD 12.5) recruited from area hospitals or care centres	Intervention: Hour long, monthly home visits for patients and caregivers if the patient desired. Structured visits included setting intentions, discussing goals and guided assessments with the aim of enabling patients to articulate what mattered to them and their goals for living. Visit delivered by a community health worker who had undertaken a 2 week training programme.	1. Patient healthcare utilisation	1. Higher proportion of IG completed an advanced directive than CG (173 vs 66, p<0.001). No significant difference in hospice use between dying patients in IG and CG. IG patients spent longer in hospice than CG (88 days vs 44 days, p<0.18). No significant differences between groups in days spent in the ED, hospital or ICU.

For peer review only - http://bmjopen.bmj.com/site/about/guidelines.xhtml

Study Number	Author & Year/<br><br>Country	Aim<br>Design<br>Theoretical model	Sample	Intervention(s)	Outcomes/measures and follow-up period	Results
				Control: Usual care – standard medical care including palliative, care management, home care, and/or hospice care services	2. Patient Quality of Life: FACIT-Pal<br><br>3. Patient care experience<br><br>4. Caregiver experience<br><br>5. Caregiver quality of life: PROMIS-29<br>Measures collected at baseline then every 3 months until death or 30 months	2. No difference between groups (p=0.649)<br><br>3. IG reported greater improvement in the communication domain than CG (p=0.16). No other statistically significant treatment by time effects.<br><br>4. No effect<br><br>5. CG carers had greater increase in anxiety and depression domains compared to IG (B=-0.98, p=0.038 and B=-0.098, p=0.014). No other statistically significant treatment by time effects.

For peer review only

Study Number	Author & Year/ Country	Aim Design Theoretical model	Sample	Intervention(s)	Outcomes/measures and follow-up period	Results
9a	Hedman, et al 2019 (27)<br><br>Sweden	To compare five-year outcomes and changes over time of a client-centred activities of daily living (ADL) intervention versus usual ADL interventions for people with stroke and their significant others.<br><br>RCT<br><br>Gothenburg PCC	People with stroke and significant others.<br><br>N=145 people with stroke (intervention group: n = 71): mean age (SD): 71(9)<br><br>control group: n = 74): mean age (SD): 68 (9)<br><br>N=75 significant others (intervention group: n = 36): mean age (SD) 65 (17)<br><br>(control group: n = 39): mean age (SD) 69 (10).	Intervention: Participants with stroke received an occupational therapist delivered client centred ADL intervention aiming to increase agency in daily activities and participation in everyday life guided by their expressed desires. Occupational therapists had participated in a 5 day workshop on client centredness.<br><br>Control: Rehabilitation in a unit providing usual ADL interventions	Primary outcome<br>1. Perceived participation: Stroke Impact scale<br><br>Secondary outcome:<br>2. Perceived participation: Occupational gaps questionnaire<br><br>3. Frequency of participation in social and complex everyday activities: Frenchay Activities Index<br><br>4. Self-reported use of assistance (yes/no) in six personal and four instrumental ADL: The Katz Extended Scale<br><br>5. Perceived self-efficacy in performing everyday activities: a Self-Efficacy Scale<br><br>6. Overall satisfaction with life: Life Satisfaction Scale	For patients:<br>1. Mean difference –6.5 (–13.3 to 0.3), p= 0.062<br><br>2. Mean difference 0.7 (–0.6 to 2.0), p=0.293<br><br>3. Mean difference –0.2 (–3.2 to 2.7), p=0.885<br><br>4. Odds ratio 0.4 (0.2 to 0.8) p=0.012<br><br>5. Mean difference 2.7 (–8.2 to 13.6), p=0.621<br><br>6. Odds ratio 0.6 (0.2 to 1.3), p= 0.219

For peer review only - http://bmjopen.bmj.com/site/about/guidelines.xhtml

Study Number	Author & Year/<br><br>Country	Aim<br>Design<br>Theoretical model	Sample	Intervention(s)	Outcomes/measures and follow-up period	Results
					7. Globally assess perceived quality of life: Reintegration into normal living index<br><br>8. Mood: Hospital anxiety and depression scale<br><br>9. Fatigue severity: fatigue severity scale<br><br>For significant others:<br><br>10. Burden of care: caregiver burden scale<br><br>11. Informal care was assessed by the use of the question 'To what extent do you assist your significant other?'	7. Mean difference –0.6 (–3.0 to 1.8), p=0.617<br><br>8. Anxiety: mean difference –0.3 (–1.6 to 1.0) p=0.611<br>Depression: mean difference –0.4 (–1.6 to 0.7), p=0.474<br><br>9: Mean difference –2.6 (–6.9 to 1.8), p=0.245<br><br>For significant others:<br><br>10: Mean difference −4.7 (−12.0 to 2.5), p=0.196<br><br>11: Mean difference −6.0 (−20.1 to 8.1), p=0.402

For peer review only

Study Number	Author & Year/ Country	Aim Design Theoretical model	Sample	Intervention(s)	Outcomes/measures and follow-up period	Results
					12. Mood: HADS as above<br><br>13. The overall satisfaction with life: The ‘My life as a whole’ item in LiSat-11 was used to assess<br><br>14. Restrictions (gaps) in participation in everyday occupations: The 30-item version of the Occupational Gaps Questionnaire.	12. Significant differences between two groups −1.7 (–3.0 to −0.5); p=0.005<br><br>13: Odds 1.1 (0.4 to 2.8) p=0.922<br><br>14: Mean difference −0.6 (−2.0 to 0.7), p=0.329
9 b, c, d	Bertilsson et al (2016) (28)<br><br>Guidetti et al (2015) (29)<br><br>Bertilsson et al (2014) (30)<br><br>(Four papers one study)	a) To determine if a client centred activity of daily living (ADL) group after stroke has an effect on caregiver burden, provision of informal care, perceived participation in everyday occupations and life satisfaction.	N= 183 caregivers of people with stroke attending inpatient or home rehabilitation n=88 intervention group, mean age 60 (SD 14.6) n=95 control group, mean age 64 (SD 13.1)	As above	1. Caregiver burden: Caregiver Burden Scale.<br><br>2. Informal care: percentage reporting providing assistance with personal ADLs, instrumental ADLs or other activities.	1. No difference between intervention and control groups at 12 months (42.7 vs 41.8, p=0.75).<br><br>2. No difference between intervention and control groups in for personal ADLs (42 vs 50%, p=0.51), Instrumental ADLs (67 vs 68%, p=0.88) or other support

For peer review only

Study Number	Author & Year/<br><br>Country	Aim<br>Design<br>Theoretical model	Sample	Intervention(s)	Outcomes/measures and follow-up period	Results
	Sweden	b) To compare changes regarding perceived participation, independence in activities of daily living (ADL) and life satisfaction between 3, 6 and 12 months after inclusion in a study of a client-centred ADL intervention and usual ADL intervention after stroke.<br><br>c) To study a client-centred activities of daily living (ADL) intervention (CADL) compared with the usual ADL intervention (UADL) in people with stroke regarding: independence in ADL, perceived participation, life satisfaction, use of home-help service, and satisfaction with training.<br><br>Cluster RCT	N=280 people with stroke<br><br>Intervention n=129, mean age (SD) 74 (10)<br><br>Control n=151, mean age (SD) 71 (10.8)		3. Participation in everyday occupations: Occupational Gaps Questionnaire (OGQ).<br><br>4. Life satisfaction: Life satisfaction scale (LiSat-11)<br>Outcomes measured at 3 and 12 months<br><br>5. Independence on ADL: Katz Extended scale (KE)	(65 vs 76%, p=0.09) at 12 months.<br><br>3. No difference between intervention and control groups (3.5 vs 4.0, p=0.52) at 12 months.<br><br>4. No difference between intervention and control groups (47 vs 47%, p=0.87) at 12 months<br>No differences between intervention and control groups in changes in outcomes between 3 and 12 months. Except the intervention group had lower General strain at 12 months than 3 months (OR 1.74, p=0.014).<br><br>5. Intervention n=38; 29.4% vs control n=52; 34.4% p=0.83

For peer review only

Study Number	Author & Year/ Country	Aim Design Theoretical model	Sample	Intervention(s)	Outcomes/measures and follow-up period	Results
					6. Perceived participation: Stroke Impact Scale (SIS)<br><br>7. Participation in everyday occupations: Occupational Gaps Questionnaire (OGQ).<br><br>8. Life satisfaction: The Life Satisfaction Scale<br><br>9. Home-help service and satisfaction with training: Self-reported (yes/no) by people with stroke.<br><br>Measures at three, six and twelve months.	6. No significant different between groups in all 9 items.<br><br>7. Mean OGQ 9.1 intervention, 107 control; p=0.10<br><br>8. N=47 (36.4%) intervention vs n=56 (37.1%) control; p=0.79<br><br>9. Home help service n=57 (44.2%) intervention vs n=60 (39.7%) control; p=0.54 Satisfaction with training n=94 (72.9%) vs n=105 (69.5%); p=0.33

For peer review only

Study Number	Author & Year/<br><br>Country	Aim<br>Design<br>Theoretical model	Sample	Intervention(s)	Outcomes/measures and follow-up period	Results
10	Ohlen et al (2019) (31)<br><br>Sweden	To evaluate whether an intervention with a person-centred approach to information and communication for patients diagnosed with colorectal cancer undergoing surgery can improve the patients' preparedness for surgery, discharge and recovery during six months following diagnosis and initial treatment<br><br>Quasi-experimental longitudinal study.	People undergoing elective surgery for cancer in the colon or rectum n=238 intervention and n=250 control.	Intervention has two components:<br>1) Written interactive patient education materials tool pertaining to phases of care process (examination, diagnosis, surgery, and recovery).<br>2) Person-centred communication in dialogue format using patient education materials. This was the tool used to communicate between the patient and health professionals.<br><br>Control group: Patients received several written patients education materials related to specific parts or procedures related to surgery and recovery. Communication occurred according to standard care.	1. The Longitudinal Preparedness for Colorectal Cancer Surgery Questionnaire (PCSQ) in Swedish measures preparedness for surgery and recovery	1. Relative to the control group, patients in the intervention group reported less decline in the domain "searching for and making use of information"<br>(slopes for control and intervention groups were -18.8 and -14.8, respectively,<br>p = 0.01). Relative to the intervention group, the control group participants reported lower scores for the domain "making sense of the recovery process" at time point 1 pre-surgery (intercepts were 80.9 and 84.4 in the control and intervention groups, p = 0.04) but no difference was detected in the slope of the trajectory. There were no statistically significant differences in intercepts or slopes between the two groups for

For peer review only - http://bmjopen.bmj.com/site/about/guidelines.xhtml

Study Number	Author & Year/ Country	Aim Design Theoretical model	Sample	Intervention(s)	Outcomes/measures and follow-up period	Results
						"understanding and involvement in the care process" and "support and access to medical care.
					Length of stay	The length of stay patients who were hospitalized in relation to surgery was 8.8 days (median = 8.0) for the control group compared with 8.0 days (median = 7.0) in the intervention group (N = 488, p = 0.033, based on the logarithm of length of stay).
					2. EORTC QLQ-C30 version 3.0 (30 items) is a widely used measure of HRQOL for patients diagnosed with cancer and the Swedish version was used	2. Patients also reported a decline in their role function; however, there was a statistically significant difference in the slopes between the two groups (-17.5 versus -7.9 in the control and intervention groups, p = 0.01).

For peer review only - http://bmjopen.bmj.com/site/about/guidelines.xhtml

Study Number	Author & Year/ Country	Aim Design Theoretical model	Sample	Intervention(s)	Outcomes/measures and follow-up period	Results
					3. The National Comprehensive Cancer Network (NCCS) Distress Thermometer (DT; Version 1.2013) was used to detect clinically significant distress in patients<br><br>Outcomes collected at six weeks, three and six months.	General health, emotional function, physical function, and cognitive functions were not significant.<br><br>3. No statistically significant differences detected between the two groups
11a	Pirhonen et al 2019 (32)<br><br>Sweden	To calculate the cost-effectiveness of a person-centred care intervention compared with usual care in patients with acute coronary syndrome (ACS)<br><br>RCT	N=252<br>n=124 intervention,<br><br>n=128 control<br><br>(1) age < 75 years, and (2) were hospitalised for	The intervention group received person-centred care according to the framework developed by the Gothenburg Centre for Person-Centred Care (GPCC), which comprises routines for establishment of a partnership between	1. Quality of life: EQ-5D-3L questionnaire<br><br>2. Direct Costs and Productivity Losses: in and outpatient care visits, diagnosis related costs, pharmaceutical costs productivity losses (indirect costs) associated	The base-case calculations showed that person-centred care was more effective and less costly compared with usual care for patients under 65 years of age, while usual care was more effective and less costly in the older age group.

For peer review only

Study Number	Author & Year/ Country	Aim Design Theoretical model	Sample	Intervention(s)	Outcomes/measures and follow-up period	Results
		Person-centred care according to the framework by the Gothenburg Centre for Person-Centred Care (GPCC)	myocardial infarction or unstable angina pectoris.	patients and healthcare professionals. The intervention was provided by designated healthcare professionals (physicians and registered nurses), at each care level, who had received training through lectures, seminars, and workshops on how to apply the intervention.<br><br>Professionals listened carefully to the patient's narrative in order to include his or her needs and intrinsic personal resources relevant for the treatment and care process. Based on this narrative, a health plan was co-created, which reflects both the perspective of the patient and the expertise of the healthcare professionals. The health plan also contained agreed goals for the recovery period, which were followed-up and revised by the patient together with the	with temporary and permanent illness, valued according to the human capital method, that is, time units of lost production were valued at their market value.<br><br>Data collected at baseline, months 1, 2 and 6 (clinical endpoint) and 1 year after the initial hospital discharge. Information on total healthcare utilisation, sickness absenteeism and drug prescriptions were collected for the 1-year period	The cost-effectiveness of the intervention was found to differ between the two age groups (< 65 years with 117 patients and ≥ 65 years with 75 patients). In the younger age group, the intervention induced lower total costs and higher quality of life, while the opposite was true in the older age group. Thus, the person-centred care intervention was the cost effective alternative when compared with usual care for those under the age of 65 years, while usual care was the cost-effective alternative in the older age group.

For peer review only

Study Number	Author & Year/<br><br>Country	Aim<br>Design<br>Theoretical model	Sample	Intervention(s)	Outcomes/measures and follow-up period	Results
				designated healthcare professionals at each care level when necessary.<br><br><br>Control: Both the intervention group and the control group received usual care according to national guidelines for cardiac care		
11b	Pirhonen et al 2017 (33)<br><br><br>Sweden<br><br>(One study reporting two papers).	To study the effects of person-centred care provided to patients with acute coronary syndrome, using four different health-related outcome measures and to examine the performance of these outcomes when measuring person-centred care.<br><br>RCT<br><br>Person-centred care according to the framework by the Gothenburg Centre for Person-Centred	The intervention n= 94 and control n=105 patients.<br><br>All other details as above	1) Patients and clinicians identify and discuss problems caused by or related to the patient's condition(s), giving due consideration to both clinical tests and treatments and the practical, social, and emotional effects of their condition(s) and treatment(s) on their daily lives.<br>2) They then engage in a shared decision-making process involving goal setting and action planning, focused on determining priorities, agreeing about realistic objectives, solving specific problems, and	1. General self-efficacy<br><br><br><br><br><br><br><br><br>2. Quality of life: EQ-5D<br><br>3. Physical activity: Grimby scale<br><br>4.Return to work	1. Patients in the intervention group reported significantly higher general self-efficacy than those in the control group six months after intervention start-up.<br><br>2-4. No siggnificant differences between the two groups.

For peer review only - http://bmjopen.bmj.com/site/about/guidelines.xhtml

Study Number	Author & Year/ Country	Aim Design Theoretical model	Sample	Intervention(s)	Outcomes/measures and follow-up period	Results
		Care (GPCC)		identifying relevant sources of support.<br>3) The agreed plan is documented and followed up.<br><br>Both groups received six-months of standard care comprised of a sequence of inpatient care, hospital-based outpatient care and primary care.		
12	Zakrisson (2019) (34)<br><br>Sweden	To test a self-management intervention in primary health care (PHC) for patients with COPD or chronic heart failure (CHF) on self-efficacy, symptoms, functioning and health<br><br>Multi-centre RCT<br><br>Based on Bandura's theory of self-efficacy	N=150 patients with COPD or CHF from 9 PHC<br>n=73 intervention group, mean age 74.0 (SD 7.4)<br>n=77 control group, mean age 71.4 (SD 8.9)	Intervention: Delivered by a physiotherapist and a nurse who had undertaken a 2-day training programme. Groups of 3 COPD and 3 CHF patients and their relatives attended six 90-minute meetings every other week for a total of 6 meetings. Patients created individual action plans based on personal problems and goal setting discussions. Patients were supported to practice skills and gain knowledge for better self-management and behavioural changes.	1. Self-efficacy: perceived self-efficacy for fatigue self-management scale (PSEFSM)<br><br>2. Anxiety and depression: Hospital Anxiety and Depression Scale (HADS)<br><br>3. Dyspnoea: modified Medical Research Council dyspnoea scale (mMRC) and New York Heart Association scale (NYHA)	1. No significant change of score at 3 or 12 months for either group.<br><br>2. No significant change of score at 3 or 12 months for either group.<br><br>3. No significant change of score at 3 or 12 months for either group.

For peer review only - http://bmjopen.bmj.com/site/about/guidelines.xhtml

Study Number	Author & Year/ Country	Aim Design Theoretical model	Sample	Intervention(s)	Outcomes/measures and follow-up period	Results
				Further meetings at 6 and 9 months to study long term effects.<br><br>Control: details not provided	4. Fatigue Impact Scale (FIS)<br><br>5. Canadian Occupational Performance Measure (COPM)<br><br>6. Six-minute walking distance test (6MWD)<br><br>7. 36 Item Short Form Survey (SF-36)<br>COPM assessed at baseline and 3 months. All other measures collected at baseline, 3 months and 1 year.	4. No significant change of score at 3 or 12 months for either group.<br><br>5. Significant improvement in IG group from baseline to 3 months (performance scores 4.7 and 5.3, p=0.04, satisfaction scores 4.5 and 5.1, p=0.03)<br><br>6. No significant change of score at 3 or 12 months for either group<br><br>7. Statistically significant improvement on social function subscale for IG between baseline and 1 year for IG (-8.3 vs 2.6, p=0.005). All other subscales no significant change.

For peer review only - http://bmjopen.bmj.com/site/about/guidelines.xhtml

Study Number	Author & Year/ Country	Aim Design Theoretical model	Sample	Intervention(s)	Outcomes/measures and follow-up period	Results
13	Arian (2018) (35)<br><br>Iran	To investigate the effect of a holistic care programme (HCP) on the reduction of iron overload in patients with beta-thalassaemia major<br><br>RCT	N=90 patients with beta-thalassaemia major referred to a large thalassaemia centre in Iran<br>n=45 intervention, mean age 25.58 (SD 3.92)<br>n=45 control, mean age 23.91 (SD 5.03)	Intervention: Patients attended the HCP over 8 weeks. This comprised individual counselling for four 45-60 min sessions, group training for four 60-90 min sessions and rehabilitation for 20 sessions<br><br>Control: Routine care at the clinic for 8 weeks	Primary outcomes:<br>1. Change in serum ferritin at three months (mg/L)<br><br>2. Change in iron level at three months (micrograms/dL)<br><br>Secondary outcomes:<br>3. Change in serum ferritin 1 year and 2 years post intervention<br><br>4. Total iron binding capacity at three months<br><br>5. Six-minute walk test (6MWT) at three months (metres)	1. Significantly greater reduction in IG (mean difference between groups -1180.84mg/L, p=0.001)<br><br>2.Significantly greater reduction in IG (mean difference - 65.555micrograms/dL, p=0.002)<br><br>3. No significant difference comparing IG and CG (p=0.07). Significant reduction within IG at 1 year (p=0.001) and 2 years (p=0.001).<br><br>4. Not significant (mean difference 8.33, p=0.724)<br><br>5. Significant improvement in IG compared to CG (mean difference 99.95m, p=0.001)

Study Number	Author & Year/<br><br>Country	Aim<br>Design<br>Theoretical model	Sample	Intervention(s)	Outcomes/measures and follow-up period	Results
					6. Haemoglobin (Hb) at three months	6. No significant difference (mean difference -0.27, p=0.425)
14	Eggers et al 2018<br><br>Germany	To assess whether a community-based, open-label, integrated approach improves QoL in PD patients.<br><br><br>RCT	N=150 Intervention group (IG), mean age (SD) 69.8 (8.4)<br><br>and 150 Control group (CG), mean age (SD) 69.9 (7.8)	The interventional group (IG) received an individually tailored therapy plan and additional home visits.<br><br>Patients randomly assigned to a control group (CG), received standard German neurological treatment	Primary outcome<br><br>1. QoL: compared the differential change of Parkinson's Disease Questionnaire (PDQ-39) from baseline to 6-month follow-up between CG and IG.<br><br><br>2. Mood: Beck Depression Inventory (BDI-2)<br><br>3. Motor: (United Parkinson's Disease Rating scale, Part III, UPDRS-III)	1. PDQ-39 significantly improved in the IG compared to the CG over the 6-month period The mean group difference as a change from baseline over 6 months was 2.20 points (95% CI − 4.4 to − 0.1), p = 0.044.<br><br>2. No significant differences<br><br><br>3. For motor symptoms, there was a significant reduction in UPDRS part III over the first 3 months in the IG (p < 0.001), and a significant between-group difference (p =

For peer review only - http://bmjopen.bmj.com/site/about/guidelines.xhtml

Study Number	Author & Year/ Country	Aim Design Theoretical model	Sample	Intervention(s)	Outcomes/measures and follow-up period	Results
						0.003). Over the 6-month period, UPDRS-III significantly improved in the IG compared to the CG (p ≤ 0.001). The mean group difference as a change from baseline over 6 months was 3.3 points (95% CI − 4.9 to − 1.7; p<0.001).
					4. Non-motor functioning: Nonmotor Symptom Score, NMS-Score	4. The scores of the PD-NMS improved after 6 months in favour of the IG (mean change 11.3, 95% CI − 17.1 to − 5.5; p<0.001).
					5. Cognition: Parkinson Neuropsychometric Dementia Assessment, (PANDA)<br><br>Data collected at baseline, three and six months.	5. No significant differences

For peer review only

Study Number	Author & Year/<br><br>Country	Aim<br>Design<br>Theoretical model	Sample	Intervention(s)	Outcomes/measures and follow-up period	Results
15	Fors et al (2018) (36)<br><br>Sweden	To evaluate the effects of person-centred support via telephone in two chronically ill patient groups, chronic obstructive pulmonary disease (COPD) and/or chronic heart failure (CHF).<br><br>RCT<br><br>Person-centred care according to the framework by the Gothenburg Centre for Person-Centred Care (GPCC)	N=221 patients ≥50 years with COPD and/or CHF<br><br>n=103 intervention Mean age (SD) 78.3 (9.5)<br><br>n=118 control Mean age (SD) 76.9 (8.3)	Patients in the intervention group were telephoned one to four weeks after discharge by a registered nurse initially to co-create a person-centred health plan with the patient and subsequently to discuss and evaluate the plan.<br><br>Nurse's initially received extensive training in person-centred communication and a two day dedicated education about CHF and COPD.<br><br>Patients in the control care group received usual care and were managed using existing guidelines for the diagnosis and treatment of acute and chronic heart failure.	1. compost score in general self-efficacy: General Self-Efficacy (GSE)	1. No significant differences between the two groups (57.6%, n = 68 vs. 46.6%, n = 48; OR = 1.6, 95% CI: 0.9±2.7; P = 0.102).<br><br>Significantly more patients in the control group had deteriorated in self-efficacy (GSE scores ≥5 units) than in the intervention group at three months (23.7%, n = 28 vs. 11.7%, n = 12; OR = 2.4, 95% CI: 1.1±4.9; P = 0.022) and at six months follow-up (22.9%, n = 27 vs. 9.7%, n = 10; OR = 2.8, 95% CI: 1.3±6.0; P = 0.011).<br><br>Improvement in GSE was significantly greater in favour of the intervention group at both three months (0.7 (mean) ± 5.8 (SD); n = 79 vs. -2.2 (mean) ± 6.1

For peer review only - http://bmjopen.bmj.com/site/about/guidelines.xhtml

Study Number	Author & Year/ Country	Aim Design Theoretical model	Sample	Intervention(s)	Outcomes/measures and follow-up period	Results
						(SD); n = 89; P = 0.010) and six months (0.9 (mean) ± 6.4 (SD); n = 69 vs. -2.0 (mean) ± 6.8 (SD); n = 85; P = 0.006
					2. Re-hospitalization and death<br><br>Each patient classified as deteriorated, improved or unchanged:<br>Deteriorated: if GSE had decreased by ≥5 units OR re-admitted to hospital for unscheduled reasons related to COPD and/or CHF OR had died;<br><br>-Improved: if GSE had increased by≥5 units AND the patient had not been hospitalized for unscheduled reasons related to COPD and/or CHF AND not died.<br>-Unchanged: neither deteriorated nor	2. There were 49 clinical events (14 deaths, 35 re-admissions) in the control group and 41 in the intervention group (9 deaths, 32 re-admissions).<br><br>Per-protocol analysis (n = 202) of the composite score showed that more patients deteriorated in the control group than in the intervention group (57.6%, n = 68 vs. 42.9%, n = 36; OR = 1.8, 95% CI 1.0±3.2; P = 0.039).

For peer review only

Study Number	Author & Year/ Country	Aim Design Theoretical model	Sample	Intervention(s)	Outcomes/measures and follow-up period	Results
					improved according to the above criteria.<br><br>GSE completed at baseline, three and at six months.	
16	Reed et al (2018) (37)<br><br>Australia	To determine whether a clinician-led chronic disease self-management support (CDSMS) program improves the overall self-rated health level of older Australians with multiple chronic health conditions<br><br>RCT	N=254 patients over 60 years with at least 2 chronic conditions from 5 general practices<br>n=127 intervention, of which 48% 60-75 years, 36% 76-85 years and 16% >85<br>n=127 control, of which 46% 60-75 years, 40% 76-85 years and 14% >85 years	Intervention: CDSMS program which uses a set of tools and structured process that enables clinicians and patients to collaboratively assess self-management behaviour, identify problems, set goals and develop individual care plans.<br><br>Control: Semi-structured positive attention program. Participants receive information relevant to their condition and scheduled contact with their clinician who was instructed to provide positive attention. All participants received 3 home visits and four follow up phone calls over 6 months from a clinician.	Primary outcome measure:<br>1. Self-rated health measured with 5-point likert scale<br><br>Secondary outcome measures:<br>2. Health status<br><br>3. Health behaviours<br><br>4. Self-efficacy	1.IG more likely to report better health than CG (OR 2.5, p=0.023) at 6 months. Most participants in both IG and CG reported no change to self-reported health from baseline to 6 months (57% IG and 69% CG). Improved health from baseline to 6 months reported in 34% of IG and 19% CG.<br><br>Secondary outcomes: 2-6 No statistically significant between group differences for any outcome

For peer review only

Study Number	Author & Year/<br><br>Country	Aim<br>Design<br>Theoretical model	Sample	Intervention(s)	Outcomes/measures and follow-up period	Results
					5. Health Education Impact Questionnaire (heiQ)<br><br>6. Health care utilisation<br>Assessed at baseline and 6 months.	
17	Schäfer et al. (2018) (38)<br><br>Germany	To determine if patient-centred communication leads to a reduction of the number of medications taken without reducing health-related quality of life<br>Two-arm cluster-randomised controlled trial	N=604 patients aged 65-84 with at least three chronic conditions recruited from 55 primary care practices<br>n=299 Intervention group, mean age 73.3 (SD 4.8)<br>n=305 control group, mean age 73.5 (SD 5.0)	Intervention: Three 30-minute PC talks with a GP over 12 months to identify treatment targets and priorities of the patient, review of all medications and discuss goal attainment and future treatment targets<br><br>Control: care as usual (details not provided)	Primary outcomes:<br>1. Change in number of medications taken by the patient<br><br>2. Health related quality of life: EQ-5D<br><br>Secondary outcomes:<br><br>3. Patient satisfaction<br><br>4. Patient empowerment<br><br>5. GP's knowledge about medication taken by the patient<br><br>6. Healthcare use	1. No statistically significant difference between IG and CG for change in number of medications (p=0.43)<br><br>2. No significant difference between groups (p=0.34)<br><br>3. No effect<br><br>4. No effect<br><br>5. No effect (p=0.772)

For peer review only - http://bmjopen.bmj.com/site/about/guidelines.xhtml

Study Number	Author & Year/<br><br>Country	Aim<br>Design<br>Theoretical model	Sample	Intervention(s)	Outcomes/measures and follow-up period	Results
						6. IG had greater contact with GPs than CG (p=0.010) but fewer days in hospital (p=0.006) and fewer attendances at physical, occupational or speech therapy units (p=0.044)
18	Armstrong et al (2017) (39)<br><br>Canada	To determine whether follow-up care delivered via a mobile app can be used to avert in-person follow-up care visits compared with conventional, in-person follow-up care in the first 30 days following ambulatory surgery<br><br>RCT	N=65 women undergoing elective breast reconstruction surgery<br>n=32 intervention, mean age 50.3 (SD12.3)<br>n=33 control, mean age 45.1 (SD 14.1)	Intervention: Planned clinic follow up replaced with daily use of QoC Health Inc mobile app. Allows users to submit photographs and responses to validated quality of recovery questionnaire and visual analogue scale for first 30 days post operatively. Surgeons follow patient reports on a web portal.<br><br>Control: planned clinic follow up at 1 and 4 weeks post operatively	Primary outcome:<br>1. Total number of follow-up visits associated with the surgery at 30 days post-op.<br><br>Secondary outcomes:<br><br>2. Total number of telephone calls and emails to the healthcare team associated with the surgery at 30 days post-op.<br><br>3.Patient reported satisfaction and convenience scores: 5 point Likert scale	1. IG had fewer follow up visits than CG (mean 0.66 vs 1.64) IG 0.4 times less likely to attend in person (p<0.001)<br><br>2. No significant difference between IG and CG in telephone calls (mean 0.31 vs 0.3, IRR 1.03, p=0.95). IG sent more emails than CG (mean 0.65 vs 0.15, IRR 4.13, p=0.05)<br><br>3. No significant difference between groups in satisfaction scores (IRR 0.95, p=0.7).

For peer review only

**Study Number**	**Author & Year/ Country**	**Aim Design Theoretical model**	**Sample**	**Intervention(s)**	**Outcomes/measures and follow-up period**	**Results**
					4. Post-operative complications: adverse events attributed to the surgery requiring a medical or surgical intervention<br>All outcomes measured at 30 days.	IG had higher convenience scores than CG (IRR 1.39, p=0.08)<br><br>4. No difference in rates of complications between groups (p=0.42).
19	Feldthusen et al 2017 (40)	To examine effects of person-centered physical therapy on fatigue and related variables in persons with rheumatoid arthritis (RA).<br><br>RCT<br><br>Gothenburg	Rheumatoid arthritis patients recruited at outpatient rheumatology clinic<br><br>(N=70): intervention group (n=36) mean age 54.2 (SD 8.5) and control group (n=34) mean age 52.7 (SD 10.9).	Each participant in the intervention group participated in the 12-week intervention of person-centered physical therapy. The goal of the intervention was, in partnership between participant and physical therapist, to devise a mutually agreed self-care plan that guided the participant in managing his or her fatigue and to effectively do so over time. The same physical therapist, experienced and specialized in RA	1. Primary outcome was general fatigue (visual analog scale).<br><br>Secondary outcomes:<br>2. Multidimensional fatigue (Bristol Rheumatoid Arthritis Fatigue Multi-	1.General fatigue improved more in the intervention group than the reference group (P=.042). Improvement in median general fatigue reached minimal clinically important differences between and within groups at post test and follow-up.<br><br>2-3 Improvement was also observed for anxiety (P=.0099), and trends toward improvements

Study Number	Author & Year/ Country	Aim Design Theoretical model	Sample	Intervention(s)	Outcomes/measures and follow-up period	Results
				management and person-centered care, conducted the intervention. The intervention was initiated with an individual person-centered meeting. A self-care plan was jointly developed and focused on tailoring health-enhancing physical activity and balancing life activities<br><br>The reference group continued with regular activities; both groups received usual health care	Dimensional Questionnaire)<br><br>3. Fatigue-related variables (ie, disease, health, function).<br><br>Data collected at baseline, three and six months.	were observed for most multidimensional aspects of fatigue (P=.023-.048), leg strength/endurance (P=.024), and physical activity (P=.023). Compared with the control group at follow-up, the intervention group improvement was observed for leg strength/endurance (P=001), and the trends toward improvements persisted for physical (P=041) and living related (P=031) aspects of fatigue, physical activity (P=019), anxiety (P=015), self-rated health (P=.010), and self-efficacy (P=046).

For peer review only

Study Number	Author & Year/ Country	Aim Design Theoretical model	Sample	Intervention(s)	Outcomes/measures and follow-up period	Results
20a	Fors et al (2017) (41)<br><br>Sweden	To assess the long-term effect of PCC in patients with acute coronary syndrome (ACS).<br><br>RCT.<br><br>Gothenburg PCC framework	N=199 with diagnosis of ACS and aged <75 years<br><br>n=94 intervention, Mean age (SD) 60.5 (9.3)<br>n=105 control, Mean age (SD) 61.3 (8.9)	PCC according to the Gothenburg PCC framework containing three routines for guiding PCC process to initiate, integrate and safeguard PCC in clinical practice. The PCC teams were trained through lecturers, workshops, and seminars on how to apply the intervention.<br><br>Comparison group received usual care comprising procedures in line with national guidelines.	Primary outcome:<br>1. Self-efficacy: general self-efficacy scale (GSE)<br><br>Measures completed at one month, two months, six months, and 24 months.	1.The composite score improved in the PCC group compared with the control group at two-year follow-up (18.1% vs 10.5% p=0.127).<br>In the per-protocol analysis, the number of patients improving was significant in favour of the PCC (21.8% vs 10.5%, P=0.039).
20b	Fors (2016)(42)<br><br>Sweden	Evaluating the effects of PCC intervention on self-efficacy after hospitalisations for acute coronary syndrome (ACS).<br><br>RCT.<br><br>Person-centred care after acute coronary syndrome, from hospital to	N=177 patients <75 years hospitalised for ACS<br>n=84 intervention. Mean age 61.0 (SD 9.2)<br>n=93 control. Mean age 61.8 (SD 8.8) years.	Provided by a group of health care professionals at the designated hospitals, outpatient clinics, and five primary care centres. Professionals were instructed through lecturers, workshops, seminars on application of PCC through teams (patient, physician, and registered nurse). Patients were engaged as partners in their care. Patients and professionals	Patient confidence in managing coronary heart disease: Swedish Cardiac Self-Efficacy Scale (S-CSES). Assessments were conducted at baseline, one month and six months.	PCC improved significantly on the dimension of control symptoms (mean 0.81 vs -0.20; p=0.049) at 1 month. No significant differences were seen at six months (p=0.366). No significant difference between IG and CG in global cardiac self-efficacy at one month (p=0.299) or six months (p=0.577)

For peer review only - http://bmjopen.bmj.com/site/about/guidelines.xhtml

Study Number	Author & Year/<br><br>Country	Aim<br>Design<br>Theoretical model	Sample	Intervention(s)	Outcomes/measures and follow-up period	Results
		primary care - A randomised controlled trial"<br>Gothenburg PCC framework		created a collaborative PCC plan within 48 hours of recruitment, then reviewed and revised at 48 hour intervals during admission. After discharge follow-up appointments were held at 4 and 8 weeks with further visits scheduled if required. Comparison received usual care following guidelines previously developed including follow up visits with a nurse at 2-3 weeks and a cardiologist at 6 weeks, then afterwards with their primary care physician at 8-10 weeks.		
20c	Fors et al 2016 (43)<br><br>Sweden	The aim of this study was to evaluate the effects of person-centred care (PCC) after acute coronary syndrome (ACS) in relation to educational level of participants.<br><br>RCT	As above (Sub study RCT)	As above	The primary endpoint was a composite of changes combining self-reported general self-efficacy with return to work or previous activity level and clinical outcomes such as re-hospitalisation or death.	In the group of patients without postsecondary education (n=90) the composite score showed a significant improvement in favour of the PCC intervention (n=40) vs. usual care (n=50) at six months (35.0%, n=<br>14 vs. 16.0%, n = 8;<br>odds ratio (OR) = 2.8,

For peer review only - http://bmjopen.bmj.com/site/about/guidelines.xhtml

Study Number	Author & Year/ Country	Aim Design Theoretical model	Sample	Intervention(s)	Outcomes/measures and follow-up period	Results
		Gothenburg PCC framework			The General Self-Efficacy Scale (GSES) is a 10-item assessed the strength in personal beliefs to cope with and adapt to a variety of daily challenges.<br><br>The Saltin-Grimby Physical Activity Level Scale was used to determine return to previous activity level among those not working. The scale is a self-reported measure of physical activity.<br><br>At 6 months after discharge, each patient was assessed as improved, unchanged, or deteriorated.<br><br>To be classified as improved required improvement in the GSES with ≥5 units,	95% confidence interval (CI): 1.0–7.7, P = 0.041). In patients with postsecondary education (n= 109), a non-significant difference in favour of the PCC intervention (n= 54) vs. usual care (n = 55) was observed in the composite score (13.0%, n = 7 vs 3.6%, n = 2; OR = 3.9, 95% CI: 0.8–19.9, P = 0.097).<br><br>A higher proportion of patients receiving the PCC intervention improved according to the composite score: 21 of 94 (22%) in the intervention group vs. 10 of 105 (10%) in the controls, p = 0.013. The same outcome applied for the GSES criteria (≥5-point improvement in the GSES): 23 of 94 (24%) vs. 14 of 105 (13%), p = 0.043. A higher proportion of individuals

For peer review only

Study Number	Author & Year/ Country	Aim Design Theoretical model	Sample	Intervention(s)	Outcomes/measures and follow-up period	Results
					return to work or previous activity level (improved from step 1 or at least unchanged from step 2) and no re-hospitalisation or death. A decrease in the GSES with ≥5 units or re-admission for unexpected cardiovascular reasons or death represented a deteriorated condition. Patients were dichotomised into two categories: improved vs. unchanged/deteriorated.	in the intervention group that fulfilled the criteria for GSES also fulfilled the other two criteria included in the composite score: 21 of 23 (91%) vs. 10 of 14 (71%), although the difference was not statistically significant (p = 0.11). This applied to 100% of the patients with low educational level that received the PCC intervention which can be compared with the corresponding figures for patients with high education that received the intervention (7 of 9, 78%) (p = 0.06) or to the controls with a low educational level (8 of 11, 73%) (p= 0.04).

For peer review only

Study Number	Author & Year/ Country	Aim Design Theoretical model	Sample	Intervention(s)	Outcomes/measures and follow-up period	Results
20d	Fors et al 2015 (44)<br><br>Sweden	To evaluate if person-centred care can improve self-efficacy and facilitate return to work or prior activity level in patients after an event of acute coronary syndrome<br><br>RCT<br><br>Gothenburg PCC framework	N=199 patients with acute coronary syndrome <75 years.<br><br>n=94 intervention mean age 60.5 (SD 9.3)<br><br>n=105 control 61.3 (SD 8.9)	In the intervention group a person-centred care process was added to treatment as usual, emphasising the patient as a partner in care. Care was co-created in collaboration between patients, physicians, registered nurses and other health care professionals and documented in a health plan. A team-based partnership across three health care levels included transparent knowledge about the disease and medical state to achieve agreed goals during recovery<br><br>All gPCC professionals had received training in the theory and practice of gPCC through lectures, seminars and workshops and were given practice in how to formulate and execute gPCC plans. Training emphasised the importance of seeing the	1. Main outcome measure was a composite score of changes in general self-efficacy ≥ 5 units, return to work or prior activity level and re-hospitalisation or death.<br><br>*Self-efficacy: General Self-Efficacy Scale* (GSE scale) a 10-item self-assessment questionnaire designed to measure a broad and stable sense of personal competence to deal effectively with a variety of stressful situations<br><br>2. Physical activity: Saltin Grimby Physical Activity Level Scale (SGPALS) is a validated measure of self-reported physical activity.<br><br>Questionnaires were completed by patients at	1. The composite score showed that more patients (22.3%, n = 21) improved in the intervention group at 6 months compared to the control group (9.5%, n = 10) (odds ratio, 2.7; 95% confidence interval: 1.2–6.2; P = 0.015). The effect was driven by improved self-efficacy ≥ 5 units in the intervention group. Overall general self-efficacy improved significantly more in the intervention group compared with the control group (P = 0.026).<br><br>2. There was no difference between groups on re-hospitalisation or death, return to work or prior activity level.

For peer review only - http://bmjopen.bmj.com/site/about/guidelines.xhtml

Study Number	Author & Year/ Country	Aim Design Theoretical model	Sample	Intervention(s)	Outcomes/measures and follow-up period	Results
				patient as a person with needs as well as resources and of a person-centred dialogue as a basis for engaging patients as actively involved partners in their own care.	baseline in hospital and at four, eight and 24 weeks per post.	
20e	Wolf et al 2016 (45)<br><br>Sweden	To investigate the effect of an eHealth diary and symptom-tracking tool in combination with PCC for patients with acute coronary syndrome (ACS).	This was a sub-study of a RCT investigating the effects of PCC in patients hospitalized with ACS.<br><br>N=199 patients with ACS aged <75 years were randomly assigned to a PCC intervention (n=94) or standard treatment (control group, n=105)<br><br>Group 1: Person-centred care plus eHealth (n=37)	Patients in the intervention arm could choose to use a Web-based or mobile-based eHealth tool, or both, for at least 2 months after hospital discharge.<br><br>A registered nurse at the hospital asked all of the patients in the eHealth group if they were interested in using the eHealth tool. Patients had the opportunity to borrow a mobile phone with the eHealth app preinstalled or to download it for use on their own mobile phone. An introductory demonstration, which required the patient to test the eHealth tools, was provided by a registered nurse who was	The primary end point was a composite score of changes in general self-efficacy: General Self-Efficacy Scale (GSES) using the Swedish version.	In the intervention arm, n=37 (39%) used the eHealth tool at least once after the index hospitalization. Most of these (24/37, 65%) used the mobile app and not the Web-based app as the primary source of daily self-rating input. Patients used the eHealth tool a mean of 38 times during the first 8 weeks (range 1–118, SD 33) and 64 times over a 6-month period. Patients who used the eHealth tool in combination with the PCC intervention had a 4-fold improvement in the primary end point compared with the

For peer review only - http://bmjopen.bmj.com/site/about/guidelines.xhtml

Study Number	Author & Year/ Country	Aim Design Theoretical model	Sample	Intervention(s)	Outcomes/measures and follow-up period	Results
			Group 2: Person-centred care only (n=57)<br><br>Group 3: Control (n=105)	familiar with the study so that patients could start using the tools freely during their hospital stay. Patients also had access to a video demonstration online for further information. The patients themselves decided on the frequency and patterns of use of the eHealth tools. Access to the webpage had no time restriction.<br><br>Patients in the control group were managed according to standard rehabilitation, which followed guideline-directed care that was compliant with Swedish standards.	Return to work or prior activity level, and rehospitalization or death 6 months after discharge.	control group (odds ratio 4.0, 95% CI 1.5–10.5; P=.005). This improvement was driven by a significant increase in general self-efficacy compared with the control group (P=.011). Patients in the PCC group who did not use the eHealth tool (n=57) showed a nonsignificant composite score improvement compared with those in the control group (n=105) (odds ratio 2.0, 95% CI 0.8–5.2; P=.14).<br><br>There were 6 events in the PCC + eHealth group (1 death, 5 readmissions), 12 events in the PCC group without eHealth (3 deaths, 9 readmissions), and 16 events in the control group (2 deaths, 14 readmissions). The proportion of patients who returned to work

For peer review only - http://bmjopen.bmj.com/site/about/guidelines.xhtml

Study Number	Author & Year/<br><br>Country	Aim<br>Design<br>Theoretical model	Sample	Intervention(s)	Outcomes/measures and follow-up period	Results
					Patients filled out the GSES instrument at baseline at the hospital, and at 4 weeks, 8 weeks, and 6 months.	was similar between groups at 6 months (PCC + eHealth 30/34, 88%; PCC no eHealth 47/53, 89%; control 89/98, 91%).
21a	Hansson et al 2017 (46)<br><br>Sweden	To compare a person-centred care intervention in terms of health-related quality of life, disease-specific symptoms or problems, with traditional care as a control group for patients with head and neck cancer.<br><br>RCT<br><br>Gothenburg PCC	N=96 patients with head and neck cancer (HNC) attending oncology care<br><br>n=54 intervention mean age 61 (SD 7.8)<br><br>n=42 control mean age 62 (SD 10.9)	Patients attended meetings with the intervention nurse, oncology specialist. The first meeting included a description of the study as well as information needed about the health-care plan. The plan was designed and developed according to a basic model from Gothenburg PCC (gPCC) and further adapted to suit patients with HNC and scheduled by the nurse and patient together. The health-care plan comprised self-management goals that were formed in partnership between the patient and the	Health related Quality of Life (HRQoL): European Organization for Research and Treatment of Cancer (EORTC) QLQ-C30 and the EORTC QLQ-35 version 3.0.<br><br>Data collected at baseline, weeks 4, 10, 18 and 52.	HRQoL was nonsignificant in all instruments. gPCC-group tended, from the 10th week, to be better than those in the control group (CG) and were, from the 18th week, statistically significantly better in the gPCC-group in terms of HNC-specific problems (QLQ-35), swallowing (p = 0.014), social eating (p = 0.048) and feeling ill (p = 0.021).

For peer review only - http://bmjopen.bmj.com/site/about/guidelines.xhtml

Study Number	Author & Year/ Country	Aim Design Theoretical model	Sample	Intervention(s)	Outcomes/measures and follow-up period	Results
				nurse. Each patient was encouraged to reflect on their self-management goals, how to reach them, and to anticipate barriers; and to refine the plan. The health plan includes both short- and long-term goals for the patient along with the actions needed to reach each goal.<br>The plan is a “living” document specific to each patient, in which the goals and actions are tracked and revised over time. The patient was also given a direct telephone number to reach the nurse specialist if they had any questions about anything relating to their treatment and wellbeing. The nurse documented the health-care plan in the medical record.<br><br>Patients randomized to the control group received usual		

For peer review only

Study Number	Author & Year/ Country	Aim Design Theoretical model	Sample	Intervention(s)	Outcomes/measures and follow-up period	Results
				care and return visits were scheduled according to the treatment procedure based on the Regional care program for patients with HNC which included post-treatment follow up visits to an oncologist at 6–8 weeks and from then on every third month for 2 years.		
21b	Gyllensten et al 2019	The aim was to examine the cost-effectiveness, including healthcare and productivity costs, of a person-centred care intervention versus standard medical care among patients with Head and Neck Care.<br><br>RCT<br><br>Gothenburg PCC	As above	As above	Health-related quality of life: EuroQol (Group's five-dimension health state questionnaire (EQ-5D™),	No significant differences<br><br>(The average total cost was Euro (EUR) 55,544 (95% confidence interval: EUR 48,474–62,614) in the intervention group and EUR 57,443 (EUR 48,607–66,279) among controls, with similar health-related quality of life)

For peer review only

Study Number	Author & Year/ Country	Aim Design Theoretical model	Sample	Intervention(s)	Outcomes/measures and follow-up period	Results
					At baseline, 4 weeks, 10 weeks, 18 weeks, and 52 weeks.	
22	Ko et al (2017) (47) Hong Kong	To evaluate whether comprehensive care programme with multidisciplinary input will decrease hospital readmissions and length of hospital stay for patients with COPD<br><br>RCT.	N=180 COPD patients admitted with an acute exacerbation. n=90 intervention. Mean age 74.9 (SD=7.9) years,<br><br>n=90 control. Mean age 74.6 (SD=8.6).	Individualised education sessions including anatomy and physiology, pathophysiology of COPD, smoking cessation, techniques of using medication, management of dyspnoea, self-management of exacerbations, coping, relaxation techniques, social and community support.<br>Patients were provided with telephone number to call and seek advice from respiratory nurse during office hours.<br>Subsequently patients received three monthly telephone calls from respiratory nurse for one year to assess their condition and answer queries.	Primary Outcome:<br>1. Hospital readmission rate at one year.<br><br>Secondary outcomes:<br>2. Length of stay (LOS)<br><br>3. Dyspnoea: Modified Medical Research Council Dyspnoea Scale (MMRC)<br><br>4. QoL: St George's Respiratory Questionnaire.<br><br>5. Lung function FEV_1/FVC ratio	1. At 12 months relative risk of readmission was 0.668, p=0.047 for the intervention group compared with the control group.<br><br>2. at 12 months IG had a shorter LOS 4.59 vs 8.86, p<0.001<br><br>3. IG had greater improvement on MMRC -0.1 vs 0.2, p=0.003<br><br>4. SGRQ: Improvement for IG at 12 months, -6.9 vs -0.1, p=0.003<br><br>5. No significant difference between groups in change in lung

For peer review only - http://bmjopen.bmj.com/site/about/guidelines.xhtml

Study Number	Author & Year/ Country	Aim Design Theoretical model	Sample	Intervention(s)	Outcomes/measures and follow-up period	Results
				Comparison group received usual care, the attending physician determined the patient's medication and follow-up as normal practice.	6. Exercise capacity: 6 minute walk test<br><br>7. Mortality	function at 12 months (p=0.653)<br><br>6. No significant difference between groups in change in exercise capacity at 12 months (-10m vs -22.5m, p=0.233)<br><br>7. Ten patients in IG and 12 in CG had died at 12 months.
23	Low et al (2017) (48)<br><br>Singapore	Evaluate the effectiveness of an integrated practice unit and modified virtual ward model in reducing readmission rates in patients at highest risk of readmission.<br><br>RCT	N=840 patients with one or more unscheduled readmissions in last 90 days and at high risk of readmission (LACE score >/=10)<br>n=420 intervention group, mean age 70.5 (SD 13.5)<br>n=420 control group, mean age 70.3 (SD 13.7)	Intervention: Hospital care transferred to Integrated Practice Unit MDT on randomisation. Intensive discharge planning including identifying and addressing risk factors for readmission. All patients provided with individualised care plan on discharge. Phone call from nurse case manager within 72 hours of discharge and home assessment within 1 week plus review at Virtual Ward MDT.	Primary outcome:<br>1. Unplanned readmissions within 30 days of discharge<br><br>Secondary outcomes:<br>2. unplanned readmissions within 90 and 180 days of discharge (visits/patient/month)<br><br>3. emergency department attendance	Primary outcome:<br>1. Readmission at 30 days was lower in the intervention group than the control group (0.25 vs 0.38, p=0.001)<br><br>2. Readmissions at 90 (0.67 vs 0.90, p=0.001) and 180 (1.05 vs 1.46, p=<0.001) days were lower in the intervention group than the control group.<br><br>3. ED visits were lower in the intervention group

For peer review only - http://bmjopen.bmj.com/site/about/guidelines.xhtml

Study Number	Author & Year/ Country	Aim Design Theoretical model	Sample	Intervention(s)	Outcomes/measures and follow-up period	Results
				Control: Standard hospital care	rate within 30, 90 and 180 days of discharge (visits/patient/month).<br><br>4. Probability of death up to 180 days	than the control group at 30 (0.26 vs 0.43, p=<0.001), 90 (0.66 vs 0.92, p=0.001) and 180 (1.14 vs 1.60, p<0.001) days.<br><br>4. 28% reduction in mortality in intervention group compared to control (HR 0.72, p<0.001).
24	Wichit et al (2017) (49)<br><br>Thailand	To evaluate a theoretically driven family-oriented intervention to improve self-efficacy, self-management, glycaemic control and quality of life in T2D<br><br>RCT.<br><br>Bandura's self-efficacy theory	N=140 T2D patients.<br><br>n=70 experimental group, mean age 61.3 (SD=11.6) years;<br>n=70 control group, mean age 55.5 (SD=10.5) years.	Family-oriented programme (patients/family dyads) consisting of education classes, group discussions, home visit, and telephone follow-up. Participants learned specialised skills such as meal planning, physical activities, managing complications. Education sessions were delivered at baseline, week 5 and week 9.<br><br>Control received usual care consisting of blood sugar testing, physical	Primary outcome<br>1. Type 2 Diabetes (T2D) self-management: Summary of Diabetes Self-Care Activities Scale (SDSCA)<br><br>Secondary outcomes:<br><br>2. T2D self-efficacy: Diabetes Management	1. At week 5 SDSCA increased from 80.9 to 96.5 in the intervention and decreased from 80.5 to 80.2 in the control, the results were significant between the two groups (p<0.001). At week 13 SDSCA was 1.2.8 in the intervention and 80.4 in the control (p<0.001).<br><br>2. At week 5 DMSES increased from 55.6 to 69.8 in the intervention, but decreased from 58.7

For peer review only - http://bmjopen.bmj.com/site/about/guidelines.xhtml

Study Number	Author & Year/ Country	Aim Design Theoretical model	Sample	Intervention(s)	Outcomes/measures and follow-up period	Results
				examinations and medication follow-up	Self-Efficacy Scale (DMSES) and Perceived Therapeutic Self-Efficacy Scale (PTES)<br><br>3. Quality of life: Thai Version short-form Health Survey (SF-12)	to 58.2 in the control (p<0.001) At week 13 DMSES further increased to 76.0 in the intervention and slightly increased in the control to 60.7 (p<0.001). At week 5 PTES increased from 32.4 in the intervention to 37.9 but decreased from 34.8 to 33.7 in the control group (p<0.001). at week 13 PTES increased in both groups to 40.8 in the intervention and 35.3 in the control group (p<0.001).<br><br>3. At week 5, Physical aspect of QoL increased in both groups from 46.7 to 50.0 in the intervention and 48.2 to 49.2 in the control (p=0.2), similar pattern occurred at week 13.<br><br>Mental aspect of QoL increased from 54.1 to 56.0 in the intervention

For peer review only - http://bmjopen.bmj.com/site/about/guidelines.xhtml

Study Number	Author & Year/ Country	Aim Design Theoretical model	Sample	Intervention(s)	Outcomes/measures and follow-up period	Results
						group. In the control group it remained at 54.3. (p=0.2). At week 13 QoL was 58.4 in the intervention and 54.7 in the control (p<0.001).
					4. Diabetes Knowledge: Diabetes Knowledge Questionnaire (DKQ)	4. At week 5 DKQ was 17.1 from 10.7 in the intervention, while it was 11.7 from 10.6 in the control (p<0.001). At week 13 DKQ was 16.5 in the intervention group and 13.2 in the control group (p<0.001)
					5. HbA1c: extracted from patient's health records	5. At baseline HbA1c was 7.0 in the intervention and 6.3 in the control. At week 13 it was 7.0 in the intervention and 7.3 in the control (p=0.2)
					Outcomes conducted at baseline and 3 weeks and 13 weeks (HbA1c was assessed at baseline and week 13).	

For peer review only

Study Number	Author & Year/<br><br>Country	Aim<br>Design<br>Theoretical model	Sample	Intervention(s)	Outcomes/measures and follow-up period	Results
25a	Larsson et al 2015 (50)<br><br><br><br>Sweden	To examine the effects of a progressive resistance exercise program on muscle strength, health status, and current pain intensity in women with Fibromyalgia (FM).<br><br>RCT<br><br>Gothenburg PCC	N=130 women with FM, n=67 resistance exercise, n=63 mean age 50.8 (SD 9.05) relaxation therapy mean age 52 (SD 9.08)	The intervention: The resistance exercise program was performed twice a week for 15 weeks and was supervised by experienced physiotherapists. It was conducted at physiotherapy premises and at a local gym at four different sites in groups comprising five to seven participants to promote interaction between participants and to facilitate physiotherapeutic guidance. The intervention was preceded by an individual introductory meeting.<br>The meeting was commenced with a dialogue between the participant and the physiotherapist about the participant's earlier experiences and thoughts of exercise.<br>The meeting also included exercise instructions, testing and adjustment of loads and modifications of	1. The primary outcome was isometric knee-extension force (N) measured with a dynamometer (Steve Strong: Stig Starke HBI, Göteborg, Sweden) using a standard protocol.<br><br>Secondary outcomes were:<br>2. Fibromyalgia impact: the fibromyalgia impact questionnaire (FIQ) a disease-specific self-reported questionnaire that comprises ten subscales of disabilities and symptoms.<br><br>3. Current pain intensity: rated on a plastic 0-100 visual analogue scale with a moveable cursor along a line and anchors at the extremes.	1. Significantly greater improvement (p = 0.010) was found for isometric knee-extension force in favor of the resistance exercise group as compared to the active control group<br><br><br>2. Significantly greater improvement was observed in health status (FIQ total score) (p = 0.038) in the resistance exercise group compared to the active control group<br><br><br>3. Significantly greater improvement was observed in current pain intensity (VAS) (p = 0.033) in the resistance exercise group compared to the active control group

For peer review only

Study Number	Author & Year/ Country	Aim Design Theoretical model	Sample	Intervention(s)	Outcomes/measures and follow-up period	Results
				specific exercises according to individual conditions and according to self-efficacy principles. The meeting resulted in a written protocol with descriptions of specific exercises and loads, which was used by each participant as an exercise program at each exercise session. The exercise was initiated at low loads, and possibilities for progressions of loads were evaluated every 3−4 weeks in dialogue between the physiotherapist and participant.<br><br>The control group was the relaxation therapy was performed twice a week for 15 weeks and was guided by experienced physiotherapists. It was conducted at physiotherapy premises at four different sites in groups comprising five to eight participants and was preceded by an individual introductory	4. The six-minute walk test (6MWT), a performance-based test that measures total walking distance (m) during a period of 6 minutes	4. Significantly greater improvement was observed in the 6MWT (p = 0.003) in the resistance exercise group compared to the active control group

For peer review only

Study Number	Author & Year/ Country	Aim Design Theoretical model	Sample	Intervention(s)	Outcomes/measures and follow-up period	Results
				meeting at the premises, which included instructions and allowed for preparations and modifications of practical matter such as positioning and the use of mattresses and pillows to reach a good level of comfort. The relaxation therapy performed a series of mental exercises including relaxation and autosuggestion. The physiotherapist guided the participants through their bodies, during approximately 25 minutes, by focusing their minds on the bodily experience of relaxation and letting the body part in focus rest on the ground. This was repeated for each specific body-part, aiming at feeling as relaxed as possible in the whole of the body at the end of the session. Participants were invited to share experiences and ask each other and the		

Study Number	Author & Year/ Country	Aim Design Theoretical model	Sample	Intervention(s)	Outcomes/measures and follow-up period	Results
				physiotherapist questions and continued thereafter with the stretching exercises.		
25b	Ericsson et al 2016 (51)	This sub-study aimed to examine the effects of a person-centered progressive resistance exercise program on multiple dimensions of fatigue in women with fibromyalgia (FM), and to investigate predictors of the potential change in fatigue.	As above	as above	Outcomes were:<br><br>1. Five dimensions of fatigue measured with the Multidimensional Fatigue Inventory (MFI-20).<br><br>2. FIQ fatigue (0–100) The VAS for fatigue included in the Fibromyalgia Impact Questionnaire (FIQ) was used as a one-dimensional measure of fatigue.	1.A higher improvement was found at the post-treatment examination for change in the resistance exercise group, as compared to change in the active control group in the MFI-20 subscale of physical fatigue (resistance group change –1.7, SD 4.3, controls change 0.0, SD 2.7, p = 0.013), with an effect size of 0.33.<br><br>2. The resistance exercise group improved in the FIQ for fatigue over time from baseline to post treatment (mean difference −8.6, SD 21.2, p = 0.002).

For peer review only - http://bmjopen.bmj.com/site/about/guidelines.xhtml

Study Number	Author & Year/<br><br>Country	Aim<br>Design<br>Theoretical model	Sample	Intervention(s)	Outcomes/measures and follow-up period	Results
					3. Pittsburgh Sleep Quality Index (PSQI) (0–21) The PSQI assesses sleep quality and disturbances over a 1-month period.	3. The resistance exercise group improved over time in the PSQI subscale for sleep quality (mean difference −0.2, SD 0.8, p = 0.047), while the active control group improved in the PSQI subscale for need of medications to sleep (mean difference 0.3 SD 1.0, p = 0.036)
					4. Pain catastrophizing scale (PCS) (0–52) The PCS assesses pain-related catastrophic thinking.	4. The resistance exercise group improved significantly over time in all three PCS subscales and the PCS total score (mean difference in PCS total score −2.7 SD 7.6, p = 0.004). In the active control group there was a tendency towards improvement in two PCS subscales and the PCS total score (p = 0.051–0.056).

For peer review only

Study Number	Author & Year/<br><br>Country	Aim<br>Design<br>Theoretical model	Sample	Intervention(s)	Outcomes/measures and follow-up period	Results
					5. Hospital Anxiety and Depression Scale (HADS) (0–21)	5. No significant changes during the study period were<br>found within any of the groups for HADS anxiety or<br>HADS depression.
26a	Hansson et al 2016 (52)<br><br>Sweden	To estimate the cost–utility of PCC when compared with conventional care in patients hospitalized for worsening chronic heart failure.<br><br>A controlled before and after design<br><br>Gothenburg PCC framework	N=248 CHF patients<br>n=125 intervention, mean age 77 (SD 11)<br><br>n= 123 control, mean age 80 (SD 9)	The intervention focused on working partnership between the patient and health professionals. it consisted of three steps:<br>1) initiating partnership: a comprehensive narrative was obtained from the patient about their symptoms and concerns to guide assessment and plan of care 2) working the partnership: encouraging active participation from the patients in their care e.g getting out of bed, patients rating their symptoms and concerns using a five-step Likert scale. This acted as a process indicator which further helped the process of decision making, 3)	Costs of care:<br>An assessment of health-related quality of life used the EQ-5D 3L instrument at baseline and at three months after discharge to usual care.<br><br>The quality of life weight was then used to calculate<br>QALYs. This measure combines years of life with quality of life so that the QALY, as a result of a treatment, can consist in increasing life expectancy and/or increased quality of life. QALY calculations were made on an individual level, reflecting the	We found that PCC resulted in lower costs (€863 per<br>patient, p=0.026) and generated marginally more health benefits than conventional care.<br><br>The costs for those who actually received PCC, per protocol (PP) (63%) were significantly (p=0.026) lower than for those in the conventional care group, with an incremental cost-saving of €863. For the first three months, patients in the conventional care group showed decreasing health-related quality of life, with

For peer review only - http://bmjopen.bmj.com/site/about/guidelines.xhtml

Study Number	Author & Year/<br><br>Country	Aim<br>Design<br>Theoretical model	Sample	Intervention(s)	Outcomes/measures and follow-up period	Results
				Safeguarding partnership: the PCC plan stipulated that decisions and assessments be documented throughout the care process in the record form.<br><br>Usual care patients were treated according to usual routines for CHF patients (details not provided).	change from baseline to three months, assuming a linear increase in quality of life (QoL) between the two measurements.	a corresponding improvement in the PCC(PP) group.
26b	Ulin et al 2016 (53)<br><br>Sweden	To evaluate whether proactive care-planning based on the Gothenburg person-centred care (gPCC) model leads to improved efficiency in discharge procedures compared with usual care in patients hospitalized for worsening chronic heart failure.<br><br>A controlled before and after design<br><br>Gothenburg PCC framework	As above	The gPCC health plan starts with the patient narrative, which includes information regarding everyday life and symptoms prior to and during the worsening of the condition. In addition, the patient's resources are identified, including motivations and goals. The social situation and the possible need for additional support at home after discharge from hospital are also of importance. Finally, within 24–48 hours, all information and facts are summarized and written in the gPCC	The first endpoint was the number of days from admission to Step 1, the first notice to the municipality, including the municipal home care service and the primary healthcare service.<br><br>The second endpoint was the number of days from admission to the second notice to the municipal home care	During hospitalization, first notifications (Step 1) to the patients' municipal home-care services and/or round-the-clock home nursing care services were more frequent in the per-protocol gPCC group (33.8%) compared with the usual care group (12.1%), but not significant.<br><br>During hospitalization, the number of days from admission to notices to the patients' municipal homecare services

For peer review only - http://bmjopen.bmj.com/site/about/guidelines.xhtml

Study Number	Author & Year/ Country	Aim Design Theoretical model	Sample	Intervention(s)	Outcomes/measures and follow-up period	Results
				health plan, which also includes planned investigations, treatment goals and length of stay at hospital.<br><br>Thereafter, the first notification can be sent to the patient's municipal home care service and to the primary healthcare service, which is Step 1. The patient and healthcare professionals discuss the gPCC health plan and reach an agreement. The gPCC health plan is regularly evaluated (and if necessary, revised) in all aspects of care (such as symptoms, resources, management and treatment) by the patient and the healthcare professionals during the hospitalization. The gPCC health plan forms the basis for the second notice to the municipal home care service and to the primary healthcare service with an	service and to the primary healthcare service confirming the discharge planning conference, or Step 2.<br><br>The third endpoint, Step 3, was the number of days from admission to the notice to the municipality that the patient was ready for discharge from hospital.	and/or round-the-clock home nursing care services for confirmed discharge planning conferences (the second notification or Step 2) was significantly decreased (p=0.03) in the per-protocol gPCC group compared with the usual care group.<br><br>The length of stay in hospital and the time to the third notification (Step 3) to the patients' municipal home-care services and/or round-the-clock home nursing care services were significantly decreased: 6.77 days in the per-protocol gPCC group compared with 9.22 days in the usual care group (p<0.01), and 11 days in the per-protocol gPCC group

For peer review only - http://bmjopen.bmj.com/site/about/guidelines.xhtml

Study Number	Author & Year/ Country	Aim Design Theoretical model	Sample	Intervention(s)	Outcomes/measures and follow-up period	Results
				accurate and detailed description of the patient's anticipated status (including for example symptoms and resources) at discharge, as well as any anticipated discharge planning conference in the hospital, which is Step 2. The third notice is recorded when the patient is ready for discharge, also in concordance with the gPCC health plan projected number of days of hospitalization, which is Step 3.		compared with 35 days in the usual care group (p=0.01), respectively
26c	Ekman et al (2012) (54)<br><br>Sweden	To evaluate outcomes of PCC in hospitalized patients with chronic heart failure (CHF) with respect to the length of hospital stay (LOS), activities of daily living (ADL), health-related quality of life (HRQL) and 6-month readmission rate	As above	As above	Primary outcome:<br>1. Length of stay (LOS) computed as number of whole inpatient days from admission to discharge	1. The mean LOS in the Usual care group was 9.22 days (SD 7.4, median 7, IQR 5, range 2–44 days) compared with 8.22 days (SD 4.4, median 8, IQR 5, range 2–31 days) in the PCC group (P . 0.16). In the PP analysis, LOS was significantly shorter (2.5

For peer review only - http://bmjopen.bmj.com/site/about/guidelines.xhtml

Study Number	Author & Year/ Country	Aim<br>Design<br>Theoretical model	Sample	Intervention(s)	Outcomes/measures and follow-up period	Results
		Controlled before and after design<br><br>Gothenburg PCC			Secondary outcomes:<br><br>2. Activities of daily living (ADL) using the Katz-ADL index<br><br>3. Quality of life (HRQL) assessed using the Swedish version of the Kansas City Cardiomyopathy Questionnaire (KCCQ)<br><br>Data collected at baseline, three months, and six months.	days) in the PCC group (6.77 days, SD 3.2, median 6.5, IQR 3, range 2–25; P . 0.01),<br><br>2. Physical functional performance as assessed with the Katz–ADL index was similar at baseline between the two groups in the analysis of all patients as well as in the PP analysis. At discharge, ADL levels were better in the PCC group (all patients, P . 0.07; the PP group, P . 0.04).<br><br>3. There were no differences in the KCCQ Overall Summary Score or the Clinical Summary score after 3 months.

For peer review only

Study Number	Author & Year/ Country	Aim Design Theoretical model	Sample	Intervention(s)	Outcomes/measures and follow-up period	Results
26d	Dudas et al 2012 (55)	To evaluate whether PCC is associated with less self-reported uncertainty in illness compared with usual care in patients hospitalized for worsening chronic heart failure (CHF).<br><br>A controlled before and after design<br><br>Gothenburg PCC framework	As above	As above	The Swedish version of the Cardiovascular Population Scale (CPS) CPS consists of two dimensions:1) ambiguity (10 items), which covers the perception of patients concerning the severity of their illness; and 2) complexity (six items), which covers the perception of patients concerning their dignity, treatment and system of care.	The PCC group had better scores than the usual care group in the CPS domains complexity (M=15.2, SD=4.7 vs. M=16.8, SD=4.7; p=0.020) and ambiguity (M=27.8, SD=6.6 vs. M=29.8, SD=6.9; p=0.041).<br><br>The PCC group reported lower scores in the dimension of ambiguity, which measures patients' self-reported experiences about uncertainty in their illness, in both the ITT analysis and in the PP analysis (M = 28.2 (SD = 6.5) and 27.8 (SD = 6.6), respectively) than the usual care group (M = 29.8 (SD = 6.9)). There was a significant difference in the dimension of ambiguity in the PP analysis between the groups for

Study Number	Author & Year/ Country	Aim Design Theoretical model	Sample	Intervention(s)	Outcomes/measures and follow-up period	Results
						patients in the PCC group (p = 0.067).
27	Jutterström et al (2016) (56)<br><br>Sweden	To evaluate the effect of a nurse led patient-centered self-management support in T2D with regard to metabolic changes.<br><br>RCT<br><br>Theory of Hernandez	N=182 people aged 40-80 with T2DM<br>n=70 Group Intervention (GI)<br>n=35 Individual Intervention (II)<br>n=36 Internal control group<br><br>n=54 External Control	Ten Diabetes Specialists Nurses (DSNs) from nine health care centres participated in a preparatory workshop of approximately 20 hrs that emphasised the patients understanding of illness. DSNs received a theoretical and practical preparation and motivating patient-centred communication aimed at supporting illness integration and how to strengthen patient's self-efficacy for self-management.<br>In the patient intervention, participants in the GI and II groups were invited to six sessions of 45-90 minutes each over a period of up to six months.<br>In the GI groups, the patients reflected aspects of living with T2D together and DSNs acted as a moderator.	1. HbA1c<br><br>2. Body mass index<br><br>3. Systolic and diastolic blood pressure	1. HbA1c significantly decreased at 12 months follow-up by 5 mmol/mol in the GI (p<0.001) and 4 mmol/mol (p=0.004)in the individual intervention (II), in the internal control group there was no change (p=0.878), while in the external control group it increased with 2 mmol/mol (p=0.213). The results were significant between intervention groups (GI and II) and external control group.<br><br>2. Body mass index was not significant between groups<br><br>3. Both systolic and diastolic blood pressure were not significant between groups

For peer review only - http://bmjopen.bmj.com/site/about/guidelines.xhtml

Study Number	Author & Year/<br><br>Country	Aim<br>Design<br>Theoretical model	Sample	Intervention(s)	Outcomes/measures and follow-up period	Results
				The intervention consisted of either discussions in groups or patients or individual conversations with the DSN, depending on the arm of allocation. During the six sessions, the participants were free to discuss issues they considered important in relation to their experiences with the disease.<br><br>Control: IC and EC groups received standard care which normally included 1-2 visits per year as per national guidelines.		
28a	Olsson et al 2016 (57)<br><br>Two papers one study	The study had two aims:<br>(1) to identify vulnerable patients using the general self-efficacy scale (GSES) and the Tampa scale for Kinesiophobia (TSK), and (2) to evaluate if person-centred care including the responses of the	Patients scheduled for total hip arthroplasty (THA), an intervention group (n = 128), mean age 68 and a control group (n = 138), mean age 66.	Intervention group received evidence-based information based on their own prerequisites. Evidence-based guidelines, clinical knowledge and patients' individual prerequisites were combined with forming a partnership with professionals.	The primary endpoint of the study was the number of days spent in the hospital relative to the self-rated GSES and TSK scores. The hospital Length of Stay was compared between the control group and the intervention group for patients scoring ≤ 29 on the GSES and/or ≥ 40 on	Significantly shorter stay in intervention group: 5.3 days (SD 2.2)<br>vs control 7 days (SD 5.0);<br>P<0.0005.<br><br>Patients with low GSES in the intervention group had shorter length of stay (LoS) by 1.6 days

For peer review only - http://bmjopen.bmj.com/site/about/guidelines.xhtml

Study Number	Author & Year/ Country	Aim Design Theoretical model	Sample	Intervention(s)	Outcomes/measures and follow-up period	Results
		instruments made rehabilitation more effective in terms of shortening hospital length of stay.<br><br>A quasi-experimental design		The first step in establishing the partnership was for a RN specialized in surgical care to obtain a narrative from each patient, covering the patient's everyday life, resources, motivation, and goals; patients were also asked to fill out the General Self-efficacy (GSES) and Tampa scale of kinesiophobia (TSK) questionnaires.<br><br>The RN then made a tentative, detailed gPCC health plan based on the narrative, the medical examination, and the self-reported results of the GSES and TSK surveys. The gPCC health plan specified each patient's short-and long-term goals, resources, special needs, and plan for recovery after discharge. The tentative health care plan was included in the patient letter provided to the patient at the outpatient clinic	the TSK. The relation between Length of Stay and American Society of Anesthesiologists" classification system (ASA) category was also studied.<br><br>1. Self-Efficacy: General self-efficacy scale (GSES)<br><br>2. Fear of Movement: Tampa Scale for Kinesiophobia (TSK)<br><br>3.Length of Stay<br><br>4. American Society of Anesthesiologists" classification system (ASA): Patients scheduled for planned surgery commonly belong to one of three categories: (1) healthy, (2) mild systemic disease, or (3) severe systemic disease. The patients in this study	(95 % CI 0.16–3.15) p=0.03.<br><br>Patients with high TSK in the intervention group had shorter LoS by 2.43 days (95 % CI 0.76–4.12) p= 0.005. For patients who had both, the reduction of LoS was 2.15 days (95 % CI 0.24–4.04) p=0.028.

For peer review only - http://bmjopen.bmj.com/site/about/guidelines.xhtml

Study Number	Author & Year/ Country	Aim Design Theoretical model	Sample	Intervention(s)	Outcomes/measures and follow-up period	Results
				appointment 2 weeks before surgery. The health plan was discussed with the patient and finalized when an agreement was reached between the professionals and the patient.<br><br>The patients were helped to familiarise themselves in the situation and to achieve their personal goal by emphasising their personal resources and capabilities documented in the health plan.<br><br>Control group received Standard care consisted of:<br><br>Completing questionnaires about their living circumstances, physical abilities and filled out surveys such as the GSES, TSK. Standardised information including peri-operative routines and postoperative training based on hip replacement	were classified by the anaesthesiologist responsible for anaesthetising patients during the surgical procedure.	

For peer review only - http://bmjopen.bmj.com/site/about/guidelines.xhtml

Study Number	Author & Year/ Country	Aim Design Theoretical model	Sample	Intervention(s)	Outcomes/measures and follow-up period	Results
				patients in general. Patients also got a written booklet containing details from the oral information about pre and postoperative care.		
28b	Olsson et al 2014 (58)	To investigate if person-centred care intervention would improve patients' recovery as measured by Length of stay LoS following hip surgery	As above	As above	1. The primary outcome measure was Length of Stay LoS, calculated as the number of whole inpatient days from admission to discharge.<br><br>2. Secondary outcomes included physical function at both discharge and 3 months later, measured with Activity of Daily Living (ADL) and Functional Recovery Scale (FRS). ADL was self-assessed by the patients at admission and measured by a nurse at discharge.	1. The mean LoS in the control group was 7 days (SD 5.0) compared to 5.3 days in the gPCC group (SD 2.2) (p <0.0005)<br><br>2. Physical functional performance: At discharge, 84% in the control group had regained ADL level A compared with 72% in the intervention group, the difference was not significant.<br><br>For FRS: Three months after surgery, 12% in the control group scored under 80% compared

For peer review only - http://bmjopen.bmj.com/site/about/guidelines.xhtml

Study Number	Author & Year/ Country	Aim Design Theoretical model	Sample	Intervention(s)	Outcomes/measures and follow-up period	Results
					3. Readmission: Any hospital readmission within 3 months was obtained from the patient records.	with 8.5% in the gPCC group and the difference was not significant.<br><br>3. Readmissions within 3 months were similar between the two groups; two patients in the control group and three in the gPCC group were readmitted and the difference was not significant.
29	Or and Tao (2016) Hong (59)<br><br>Hong Kong	Evaluate the effects of a person-centred tablet computer-based self-monitoring system for chronic disease (T2D and/or hypertension).<br><br>RCT	N=63 patients with T2D and/or hypertension n=33 intervention, mean age 69.3 (SD 9.7) n=30 control, mean age 69.7 (SD 10.2)	Tablet computer-based disease self-monitoring system. The system was interactive with 10 inch tablet computer, blood glucose and blood pressure monitor (2 in 1). The system would indicate Vital signs values. Abnormal values were measured in red, normal values in green. The system also had video-based educational materials that allowed patients to learn how to self-manage their chronic conditions, e.g.	1.Systolic and diastolic blood pressures	1. Significant improvements were seen in systolic blood pressure in the intervention group from baseline to 1 month (-16.7 mm Hg), 2 months (-10.3 mm Hg) and 3 months (-13.0 mm Hg). Non-significant differences were seen in the control group (-2.1 mm Hg) at month one, 6.2 at 2 months, and -5.4 mm Hg at 3 months. The differences were significant between the

For peer review only - http://bmjopen.bmj.com/site/about/guidelines.xhtml

Study Number	Author & Year/ Country	Aim Design Theoretical model	Sample	Intervention(s)	Outcomes/measures and follow-up period	Results
				how to measure glucose, BP, diet, and exercises.<br><br>Comparison group received a 2-in-1 blood glucose and blood pressure monitor for self-monitoring and a logbook for recording the vital signs measured and the dates and times of measurements.	 <br><br><br><br><br><br><br><br><br><br><br><br><br><br><br><br><br><br><br><br><br><br>2.Fasting blood glucose	two groups after 1 month (p<0.001) and month 3 (p=0.043).<br>Similarly significant differences were seen in diastolic pressure in the intervention group (-8.0 mm Hg) at 1 month, -6.6 mm Hg at month 2, and -5.7 mm Hg at month 3. Non-significant decline were seen in the control group -0.3 mm Hg at 1 month, -1.9 mm Hg at 2 months, and -2.0 mm Hg at 3 months. The decline in diastolic pressure were significantly greater in the intervention group than control group after 1 (p<0.001) and 2 months (p=0.028).<br><br>2. After 3 months non-significant decline in FBG was seen in the intervention group (-1.0 mmol/dL) and an increase in the control group (0.4 mmol/dL), the trend was not statistically

For peer review only

Study Number	Author & Year/<br><br>Country	Aim<br>Design<br>Theoretical model	Sample	Intervention(s)	Outcomes/measures and follow-up period	Results
					3. HbA1c<br><br>4. Patient's knowledge of T2D and hypertension: Modified Michigan Diabetes knowledge Scale and the hypertension knowledge questionnaire.<br>Measured at baseline, months 1,2, and 3.	different between groups (p=0.407).<br><br>3. HbA1c<br>Both decreased at 3 months -0.2 in the intervention and control groups. No between group differences.<br><br>4. No significant differences on knowledge of hypertension and T2D.
30a	Sahlen et al (2016) (60)<br><br>Sweden	To assess the cost-effectiveness of person-centred care integrated heart failure and palliative home care.<br><br>RCT<br><br>Person-centred palliative care model.	N=72 participants with NYHA class III-IV heart failure<br><br>n=36 intervention<br>n=36 control	Person-centred integrated intervention. Structured PCC (partnership between patients/carers and professional caregivers and includes initiating, working on and documenting partnership) with a collaborative approach between palliative and heart failure care specialists	1.Quality adjusted life years (QALYS) EQ-5D	1.QALY was 0.569 in the intervention and 0.538 in the control group as baseline. Slight improvement was seen in the intervention (+0.006), but declined in the control group (-0.024), p=0.026.

For peer review only

Study Number	Author & Year/ Country	Aim Design Theoretical model	Sample	Intervention(s)	Outcomes/measures and follow-up period	Results
		Six S: self-image, self-determination, social relationships, symptom control, synthesis and surrender.		involving rounds with all team members every 2 weeks. Care delivered at home with easy access to care with frequency and duration of calls dependent on patient need. The team was responsible for total care including co-morbidities.<br><br>Comparison group received usual care consisting of nurse-led heart failure clinic at the hospital or primary health care centre.	2. Costs of health care: multiplying the allocated time for given services by the average salaries.<br><br>Data collected at baseline, and month six.	2. Cost of intervention SEK (Swedish krona) 1.4 million (140,000 Euros). The control costed SEK 2 million (205,000 euros). The intervention reduced costs of SEK 600,000 over the 6 month intervention period.
30b	Brännstrom & Boman (2014) (61)<br><br>Sweden.	To evaluate the effect of a PCC and integrated palliative advanced home care and heart failure care.<br><br>RCT.<br><br>Person-centred palliative care model. Six S: self-image, self-determination, social relationships, symptom control, synthesis and surrender.	N=72 patients with CHF class III-IV.<br>n=36 intervention<br>n=36 control	Multi-disciplinary approach involving collaboration between specialists in palliative care and heart failure care (specialised nurses, palliative care nurses, cardiologists, palliative care physician, physiotherapists and occupational therapists. Patients also received structured PCC at home. The model used the six S as Sahlen et al (2016) above	1. Symptom burden: Edmond Symptom Assessment Scale (ESAS)<br><br>2. Health related QoL-Euro QoL (EQ-5D)	1. ESAS was not significant between the groups (data not provided).<br><br>2. No significant differences in QoL between the two groups (47.7 to 60.4 in the intervention group and 48.2 to 52.3 in the control group), P=0.10. Age-adjusted analysis between groups showed delta value of HRQL

For peer review only - http://bmjopen.bmj.com/site/about/guidelines.xhtml

Study Number	Author & Year/ Country	Aim Design Theoretical model	Sample	Intervention(s)	Outcomes/measures and follow-up period	Results
				Control: usual care as described above (Sahlen et al; 2016).	3. Kansas City Cardiomyopathy Questionnaire (KCCQ)<br><br>Assessments were conducted at baseline, 3 and 6 months.	from baseline to 6 months was significantly better in the intervention compared to control (p=0.02).<br><br>3. No significant differences were found between the two groups (data not provided).
31	Slok et al. (2016) (62)<br><br>The Netherlands	To assess the effectiveness of the Assessment of Burden of COPD (ABC) toll on disease specific quality of life in patients with COPD<br><br>A Cluster RCT.	N=39 primary care practices, 17 hospitals<br>N=357 COPD patients<br>n=175 intervention, mean age 64.8 (SD 8.7)<br>n=182 control, mean age 65.8 (SD 8.8)	Applied the ABC tool consisting of a short validated questionnaire assessing the experienced burden of COPD, parameters of COPD lung function, and treatment algorithm including visual display and treatment advice.<br>GPs, nurses, pulmonologists were instructed to use the ABC tool during their routine consultations. Patients visited health care	Primary outcomes:<br>1. Improvement in disease-specific quality of life at 18 months; St George's Respiratory Questionnaire (SGRQ)<br><br>Secondary outcomes:<br>2. Disease-specific quality of life; COPD Assessment Test (CAT)	1. At 18-months 34% of the 146 patients from 27 health care providers in the intervention group had a clinically significant improvement in the SGRQ (at least 4 points) compared with 22% of the 146 patients from the 29 healthcare providers in the control group (OR 1.85; p=0.02).<br><br>2. No significant differences in the CAT between the two groups (-0.26; p=0.68).

For peer review only - http://bmjopen.bmj.com/site/about/guidelines.xhtml

Study Number	Author & Year/ Country	Aim Design Theoretical model	Sample	Intervention(s)	Outcomes/measures and follow-up period	Results
				professionals at least four times in 18 months. Patients were asked to fill out the ABC scale, report their dyspnoea using the MRC dyspnoea scale and self-report level of physical activity. Patients and providers could decide on treatment plan together. Patients formulated personal treatment goals.<br><br>Health care professionals in the control group provided usual care according to Dutch COPD guidelines.	3. Perceived QoL: Patient Assessment of Chronic Illness Care (PACIC)<br>Collected at four time points: baseline, 6 months, 12 months and 18 months.	3. PACIC improved significantly in the intervention group compared with the control group at 18 months (0.32; p<0.01).
32	Windrum et al (2016) (63)<br><br>UK	To examine the relative impacts of alternative patient education programmes for people newly diagnosed with type 2 diabetes.<br><br>RCT	N=203 patients with Type 2 Diabetes from 6 General Practices in a city<br>n=94 intervention, mean age 65.8 (SD 9.69)<br>n=109 control, mean age 65.35 (SD 8.45)	Intervention: Patient centred education based on mediated learning. Delivered by health care professionals who attended a two-day course. Discussions were mediated between patients on key areas of health and self-management. Patients learnt to use and critically appraise information, translating it to their own	Fasting HbA1c at diagnosis and at 12 months after education programme in mmol/l.	1. HbA1c significantly lower in IG than CG after 12 months (6.838 vs 7.163, p<0.05)

For peer review only - http://bmjopen.bmj.com/site/about/guidelines.xhtml

Study Number	Author & Year/ Country	Aim Design Theoretical model	Sample	Intervention(s)	Outcomes/measures and follow-up period	Results
				individual circumstances. Patients received an 'education pack' with the same basic information as the control group and were encouraged to reflect on their own behaviour and health choices. Finally patients created a personal action plan with key goals for diet, exercise and lifestyle.<br><br>Control: Didactic course of diabetes education including causes of the condition, symptoms, diet and exercise and foot care. Patients also received NHS and Diabetes UK information leaflets.		
33	Yu (2016) (64)<br><br>Hong Kong	To develop an innovative geriatric practice, a health and social collaborative case management (HSC-CM) for family caregivers of older adults and conduct a pilot RCT	N=60 family caregivers co-residing with frail older adults and providing 6 or more hours of care daily recruited from an elderly	Intervention: A comprehensive health and social assessment of caregiver and care recipient conducted in the first 4 weeks by two case managers, a registered nurse and a social worker. A case manager was	1. Caregiver perceived burden: Caregiver burden inventory (CBI, Chinese version).<br><br>2. Caregiver and health-related quality of life: Medical Outcomes Study 36-item Short Form	1. IG had significantly greater reduction in perceived burden (p=0.03) than CG<br><br>2. IG had significant improvement in vitality (p=0.049), social role functioning (p=0.47) and

For peer review only

Study Number	Author & Year/ Country	Aim Design Theoretical model	Sample	Intervention(s)	Outcomes/measures and follow-up period	Results
		Pilot RCT	community centre run by the YWCA n=30 carers in intervention group, mean age 61.5 (SD 15.5) n=30 carers in control group, mean age 61.2 (SD 17.1)	assigned to provide integrated, coordinated continued care from week 5-16. Caregivers were invited to attend group workshops according to their needs to optimise informational, emotional and social support between peers.<br><br>Control: usual care.	Health Survey (SF-36 Chinese version)	general well-being (p=0.49).
34	Hernandez et al, (2015) (65)<br><br>USA	Explore the effectiveness of a community-based integrated care (IC) service in preventing hospitalisations and emergency department visits in stable frail COPD patients<br><br>RCT	N=155 COPD patients. n=71 intervention. Mean age 73 (SD=8) years. n=84 control, mean age 75 (SD=9) years.	A 2-h educational programme administered by nurse covering disease knowledge, non-pharmacological treatments, techniques for pharmacological administration, and self-management of the disease and co-morbid conditions and strategies to adopt with future exacerbations. A joint visit of the specialist nurse and the primary care team (physician, nurse, social worker) at patient's home within 72 hours after study entry.	1. Hospital admission and visit to emergency department<br><br>2. Mortality<br><br>3. Dyspnoea: MRC dyspnoea scale<br><br>4. Anxiety and depression: HADS	1. IC group showed decline in risk of emergency room visits; OR: 0.33 p=0.02. Hospital admissions did not differ significantly OR: 2.17; p=0.237<br><br>2. Mortality reduced in the IC group OR:0.36; p=0.034<br><br>3. No difference between groups (p=0.96) at 12 months<br><br>4. No differences on anxiety between the

For peer review only - http://bmjopen.bmj.com/site/about/guidelines.xhtml

Study Number	Author & Year/<br><br>Country	Aim<br>Design<br>Theoretical model	Sample	Intervention(s)	Outcomes/measures and follow-up period	Results
				Community care team received 2 h face-to-face educational training and 1 day stay at the hospital ward, aiming at enhancing home-based management of frail COPD patients. Number of home visits individually tailored to patient needs.<br><br>Usual care: Comparison group received conventional treatment being managed by their physician without any support from specialised nurses. Visits were every 6 months in the out-patient clinic.	5. QoL: St George's Respiratory Questionnaire<br><br>6. COPD knowledge and self-management<br><br>7. Percentage of current smokers	groups (p=0.13), but depression significantly improved in the IC group (p<0.01) at 12 months<br><br>5. Symptoms score significantly reduced in the IC group compared with the control group 32 vs 42 p=0.02, activity and impacts scores did not change significantly 63 vs 69; p=0.20, 36 vs 40; p=0.28 respectively.<br><br>6. knowledge significantly increased in the IC group compared with the control group 40 vs 25; p=0.02<br><br>7. Lower percentage of current smokers in the intervention group (3% vs 16%, p=0.002.
35	Kikkenborg et al (66)(2015)<br><br>Denmark	To examine the potential effects of a short psychoeducational nursing intervention on	N=196 adults with first time ICD implantation	Intervention: Three monthly, one hour nurse led psychosocial support and education sessions commencing on discharge.	1. Primary Emotions using The Emotions and Health Scale Measured at baseline and 3 months	1. No significant differences in primary emotions between intervention and control groups at 3 months.

For peer review only - http://bmjopen.bmj.com/site/about/guidelines.xhtml

**Study Number**	**Author & Year/ Country**	**Aim Design Theoretical model**	**Sample**	**Intervention(s)**	**Outcomes/measures and follow-up period**	**Results**
		primary emotions and describe the trajectory of primary emotions over time in patients with implantable cardioverter defibrillators (ICD).<br>RCT<br><br>Theory of nursing, Rosemary Rizzo Parses Human Becoming Practice	n=99 intervention group, mean age 58<br>n=97 control group, mean age 58	Control: Usual care plus an invitation to attend a single 2 hour group session with information and sharing of experiences but no individual psycho-educational follow-up.		Joy (11 vs 10.8, p=0.76), Agreableness (10.4 vs 10.2, p=0.64), Surprise 77 vs 80, p=0.67, Fear 6.76 vs 6.94, p=0.42, Sadness (8.15 vs 7.64, p=0.06) Disgust (4.62 vs 4.96, p=0.83), Anger (5.68 vs 6.04, p=0.97, Anticipation 8.34 vs 8.83, p=0.35).
36a	Larsson et al (2015) (67)<br><br>Sweden	To compare the costs of rheumatology care between a nurse-led rheumatology clinic (NLC) based on person-centred care (PCC), versus a rheumatologist-led clinic (RLC) in monitoring patients with chronic inflammatory arthritis (CIA) undergoing biological therapy.<br><br>RCT	N=97 patients with CIA undergoing biological therapy and a disease activity score (DAS28 </=3.2) recruited from a rheumatology clinic in Southern Sweden<br>n=47 intervention group, mean age 55.0 (SD 12.3)<br>n=50 control group, mean age 55.8 (SD 13.2)	Intervention: Patients randomised to attend a NLC based on the principles of patient centred care. In addition to assessing disease activity and medication, visits focussed on patients needs and global health. Patients could contact their nurse when needed between appointments.<br>Control: attending a Rheumatologist led clinic. Visits to both clinics lasted about 30 minutes.	Total annual use of resources and direct costs of care monitoring biological therapy over 12 months<br>Secondary outcome measures:<br>Annual use of resources and direct costs for the components of the primary outcome (fixed monitoring, variable monitoring, rehabilitation, specialist consultations, radiography and pharmacological therapy).	Statistically significant lower costs in IG than CG (€14107.7 vs €16274.9 per patient, p=0.004)<br><br>Statistically significant cost reductions in total fixed monitoring (-€116.7, p=0.001), total (fixed and variable) monitoring (-€155.0, p=0.001) and pharmacological therapy (-€1444.5, p=0.029). No statistically significant reduction in monitoring

For peer review only

Study Number	Author & Year/ Country	Aim Design Theoretical model	Sample	Intervention(s)	Outcomes/measures and follow-up period	Results
		Gothenburg PCC				visits, blood tests, additional phone consultations, inpatient and outpatient rehabilitation, physiotherapy, occupational therapy, psychosocial treatment, specialist consultations or radiography.
36b	Larsson et al (2013) (68) Sweden	To compare and evaluate the treatment outcomes of a nurse-led rheumatology clinic and a rheumatologist clinic in patients with low disease activity or undergoing remission who are undergoing biological therapy<br><br>RCT<br><br>Gothenburg PCC	n= 107 patients with chronic inflammatory arthritis undergoing biological therapy and a disease activity score (DAS28 </=3.2) recruited from a rheumatology clinic in Southern Sweden<br>n=53 intervention, mean age 55 (SD 12.3)<br>n=54 control, mean age 55.8 (SD 13.2)	Intervention: Patients randomised to attend a NLC based on the principles of patient centred care. In addition to assessing disease activity and medication, visits focussed on patients needs and global health. Patients could contact their nurse when needed between appointments.<br><br>Control: attending a Rheumatologist led clinic. Visits to both clinics lasted about 30 minutes	Primary outcome:<br><br>1. Disease activity: DAS28 and DAS28-CRP<br><br>Secondary outcomes:<br>2. Performing Activities of Daily Living (ADLs): Health Assessment Questionnaire (HAQ)	Mean difference of change (IG-CG) between groups not statistically significant for any primary or secondary outcome<br>1. DAS28 (-0.06, p=0.66) or DAS28-CRP (0.05, p=0.70)<br><br>2. 0.02, p=0.79

For peer review only - http://bmjopen.bmj.com/site/about/guidelines.xhtml

Study Number	Author & Year/ Country	Aim Design Theoretical model	Sample	Intervention(s)	Outcomes/measures and follow-up period	Results
					3. Pain assessed by Visual Analogue Scale<br><br>4. Satisfaction in obtaining rheumatology care: Numerical Rating Scale<br><br>5. Confidence in obtaining rheumatology care: Numerical Rating Scale	3. Non-significant -0.24, p=0.95<br><br>4. Non-significant 0.25, p=0.43<br><br>5. Non-significant 0.2, p=0.42
37	Lowther et al (2015) (69)<br><br>Kenya	To evaluate the effectiveness of a nurse-led palliative care intervention among people with HIV<br><br>RCT	N=120 participants with HIV<br><br>n=60 intervention, mean age 38.3 (SD 8.2)<br>n=60 control, mean age 40.5 (SD9.2)	Patients in the intervention arm received clinical care from a nurse who has received two weeks' training in palliative care and ongoing clinical support and supervision from experienced palliative care providers.<br><br>Control group received care from nurse's who had no exposure to palliative care training.	Primary Outcome:<br>1.Pain severity: African Palliative Care Outcomes (APOS)<br><br>Secondary Outcomes:<br>2.Psychiatric morbidity: GHQ-12	1.Mean change was +3.5 in the intervention and +4.0 in the control (p=0.83)<br>Total APOS mean change was +12 in the intervention and +7.5 in the control (p=0.04).<br><br>2. Significant difference was seen between intervention and control (-0.50; p=0.04).

For peer review only - http://bmjopen.bmj.com/site/about/guidelines.xhtml

Study Number	Author & Year/<br><br>Country	Aim<br>Design<br>Theoretical model	Sample	Intervention(s)	Outcomes/measures and follow-up period	Results
					3. Quality of Life (mental and physical: Medical Outcomes Study (MOS)-HIV<br><br>Outcomes assessed at baseline, one, two, three and four months.	3. Significant differences between groups on mental health subscale (0.61; p=0.01) but no significant differences between groups on physical aspects of QoL(0.44; p=0.06).
38	Kelechi et al. (2014) (70)<br><br>USA	To test the feasibility and efficacy of a motivational enhancement and conditioning activity for leg function (MECALF) in patients with critically colonized/infected chronic leg ulcers.<br><br>Comparative study<br>Motivational Enhancement	N=21 patients with critically colonised or infected leg or foot ulcers.<br>n=12 intervention<br>n= 9 control	Intervention: MECALF. Specialist nurses received 8 hours of training in motivational enhancement (ME). They used 10 minutes of each weekly wound visit to engage in ME over 6 weeks. Patients were given a brochure detailing an exercise programme (CALF) to promote walking and other physical activities developed by a physical therapist.<br><br>Control: CALF. Usual wound care as per protocols. Patients received the CALF exercise brochure but no ME.	Data collected at baseline and week 8 (2 weeks post intervention)<br>1. Pain : Leg Pain Questionnaire (LPQ)<br><br>2. Strength: dyanometer for ankle dorsiflexion and plantar flexion in lb/in^2<br><br>3. Ankle range of motion: goniometry for dorsiflexion, plantar flexion, inversion and eversion in degrees<br><br>4. Motivation: readiness ruler	1. Reduced pain at 8 weeks in CG compared to IG (p=0.046)<br><br>2. No statistically significant difference between groups.<br><br>3. No statistically significant difference between groups at 8 weeks (p=0.748)<br><br>4. No statistically significant difference between groups (p=0.641)

For peer review only - http://bmjopen.bmj.com/site/about/guidelines.xhtml

Study Number	Author & Year/<br><br>Country	Aim<br>Design<br>Theoretical model	Sample	Intervention(s)	Outcomes/measures and follow-up period	Results
					5. Self-efficacy/confidence: Questionnaire for Physical Activity and Exercise<br><br>6. Functional physical activity: Timed chair rise test, timed up and go, community healthy activities model for program for seniors (CHAMPS).	5. No statistically significant difference between groups (p=0.643)<br><br>6. No statistically significant difference between groups in any measure.
39	Young et al (2013) (71)<br><br>Australia	To investigate the effectiveness of a centralised, nurse-delivered telephone based service to improve care coordination and patient reported outcomes after surgery for colorectal cancer.<br><br>RCT	N= 756<br>n=387 intervention group, mean age 86.9 (SD 12.2)<br>n=369 control group, mean age 67 (SD 12.1)	Five scheduled, structured telephone calls from a nurse on days 3 and 10 then at 1,3 and 6 months after hospital discharge. Identified needs were addressed by the nurse using detailed standardized clinical protocols.<br><br>Control group received usual care.	Primary and secondary outcomes not specified.<br>1. Total care coordination score at 3 and 6 months<br><br>2. Global assessment of care coordination at 3 and 6 months	1. No significant differences between intervention and control groups at 3 (79.5 vs 78.7, p=0.3) or 6 months (80 vs 80.3, p=0.8).<br><br>2. No significant differences between intervention and control groups median scores at 3 (9 vs 9, p=1.0) or 6 months (10 vs 10, p=0.1).

For peer review only

Study Number	Author & Year/ Country	Aim Design Theoretical model	Sample	Intervention(s)	Outcomes/measures and follow-up period	Results
					3. Global assessment of quality of care at 3 and 6 months	3. No difference in intervention and control groups median scores at 3 (10 vs 10, p=1.0) or 6 months (10 vs 10, p=1.0)
					4. Supportive Care Needs Survey Short Form (SCNS-SF34) at 3 and 6 months	4. No difference in intervention and control group unmet needs median score at 3 (59.9 vs 56.8, p=0.6) or 6 months (50.0 vs 46.6, p=0.7)
					5. Unplanned readmissions at 1 and 6 months	5. No difference between intervention and control group in unplanned admissions at 1 (8.6 vs 10.5%, p=0.4) or 6 months (25.6 vs 27.9%, p=0.5)
					6. Emergency room presentations at 1 and 6 months	6. No difference between intervention and control group in emergency room presentations at 1 (10.8 vs 13.8%, p=0.2) or 6 months (25.9 vs 25.4%, p=0.9)

For peer review only

Study Number	Author & Year/ Country	Aim Design Theoretical model	Sample	Intervention(s)	Outcomes/measures and follow-up period	Results
					7. Proportion receiving postoperative chemotherapy<br><br>8. Distress at baseline, 1, 3 and 6 months<br><br>9. Functional Assessment of Cancer Therapy- Colorectal (FACT-C) total score at baseline, 1, 3 and 6 months	7. No significant difference between intervention and control groups in proportion receiving postoperative chemotherapy (73 vs 78%, p=0.5)<br><br>8. No difference in intervention and control groups in mean distress scores at 1 (2.3 vs 2.4, p=0.1), 3 (2.0 vs 2.0, p=0.3) or 6 months (1.8 vs 1.8, p=0.2)<br><br>9. No significant difference between intervention and control groups in FACT-C total score at 1 (100.61 vs 100.40, p=0.4, 3 (103.48 vs103.26, p=0.4) or 6 months (105.10 vs 105.35, p=0.5)
40	Chochinov (2011) (72)<br><br>USA, Canada and Australia	To determine if dignity therapy could mitigate stress and/or bolster end-of-life experience	N=326 patients receiving hospital or community based palliative care	Dignity Therapy: novel brief (30 min) psychotherapy session providing an opportunity to speak about things that matter most to	Primary outcomes:<br>1. Mean change in baseline and end of intervention	Primary outcomes:<br>1-7. No significant differences found in change from baseline to end of intervention

For peer review only

Study Number	Author & Year/ Country	Aim Design Theoretical model	Sample	Intervention(s)	Outcomes/measures and follow-up period	Results
		for patients nearing death<br><br>Multi centre RCT	n=108 dignity therapy, mean age 64.2 (SD 14.6)<br>n=107 client centred care, mean age 64.3 (SD 14.3)<br>n=111 standard palliative care, mean age 66.7 (SD 14.2)	the patient often relating to meaning and purpose. Sessions were transcribed to produce a document that could be bequeathed to a recipient of patient's choice. Therapists undertook 3 day training.<br>Client Centred Care: Supportive psychotherapeutic approach focussing on 'here and now' issues such as symptoms and their illness. No permanent record of conversation given to patient.<br>Standard Palliative Care: access to MDT palliative care support services.<br><br>Control group: Participants assigned to the control group received Standard Palliative Care which included access to the full range of palliative care support services available to all study patients, including specialist palliative care physicians and nurses	2. Palliative Performance Scale<br><br>3. FACIT spiritual well-being scale<br><br>4. Patient dignitary inventory (PDI)<br><br>5. Hospital anxiety and depression scale (HADS)<br><br>6. Items from Structured Interview for Symptoms and Concerns (SISC) including dignity, desire for death, suffering, hopelessness, depression, suicidal ideation and sense of burden to others.<br><br>7. Two item quality of life scale<br><br>Secondary outcome:<br>8. Detailed survey of experience of study	between the three groups in any outcome measure.<br><br>8. Dignity therapy group more likely to have found the study helpful

For peer review only - http://bmjopen.bmj.com/site/about/guidelines.xhtml

Study Number	Author & Year/ Country	Aim Design Theoretical model	Sample	Intervention(s)	Outcomes/measures and follow-up period	Results
				(i.e. experts in pain and symptom management), social workers, chaplains, and psychologists and/or psychiatrists. No participating site provided a formal approach to addressing generativity issues; as such, a program comparable to Dignity Therapy was not available to patients who were not randomized to the Dignity Therapy arm of this trial.		(p<0.001), that it improved their quality of life (p<0.001), sense of dignity (p=0.002), spiritual wellbeing (p=0.006), lessened sadness or depression (p=0.009) and felt satisfied with the study arm assignment (p<0.001). The Dignity Therapy group were likely to report that being in the study changed how their family appreciate and see them (p<0.001) and that it will help their family p<0.001).
41	Goelz et al (2011) (73)<br><br>Germany	To demonstrate that COM-ON-p concise and individualized communication skills training (CST) improves oncologists communication skills in consultations focussing on the transition to palliative care	N=41 physicians in charge of patients with cancer and practising at a University Medical Centre in Germany n=22 physicians in intervention group	Intervention: Participants undertook the COM-ON-p training programme including pre-assessment with an actor patient (1 hour), a 1.5 day workshop and an individual coaching workshop (30 mins) 2 weeks after the workshop and post assessment with an actor patient (1 hour).	COM-ON-Checklist: Participants were ranked on 5 point scale for relevant behavioural domains.<br>Primary outcome:<br><br>1. Section A average score for 6 items specific to the transition to palliative care	 <br><br><br><br><br>1. IG had significantly higher scores than CG after intervention (Effect size 0.78, p=0.0026)

For peer review only - http://bmjopen.bmj.com/site/about/guidelines.xhtml

Study Number	Author & Year/ Country	Aim Design Theoretical model	Sample	Intervention(s)	Outcomes/measures and follow-up period	Results
		RCT	n=19 physicians in control group	Facilitators were experienced in oncology and CST and helped physicians focus on individual learning goals which they had developed with video analysis.<br><br>Control: No additional training.<br>All physicians undertook 2 video recorded consultations with actor patients at baseline and 5 weeks later.	2.Section B average score for 9 general communication items<br><br>Secondary outcome:<br>3. Involving significant others: Section C average score of 4 items on the involvement of significant others and global item 2.	2. IG had significantly higher scores than CG after intervention (Effect size 0.78, p=0.0078.<br><br>3. IG had significantly higher scores than CG after intervention (Effect size 0.65, p=0.0070).
42	Murphy et al (2010) (74)<br><br>USA	To examine whether tailored activity pacing intervention was more effective than general activity pacing intervention for managing pain and fatigue in adults with osteoarthritis.<br><br>RCT	n=13 intervention group with OA, mean age 63.9 (SD=7.8)<br>n=11 control group with OA, mean age 59.5 (SD= 6,6)	Intervention: Education module on activity pacing tailored to the individual delivered by an occupational therapist. Participants undertook 5 days of home monitoring of activity levels with an accelerometer and a log of symptoms and activity. A personalised report detailing the relationship between activity and symptoms was the basis for pacing recommendations.	Primary outcomes:<br>1. Pain: WOMAC<br><br>2. Fatigue: Brief Fatigue Inventory	1. WOMAC pain score decreased from baseline to week 10 in the control group (9.4 to 7.6) and the intervention group (7.9 to 6.7). The difference between groups was not statistically significant (p=0.35) with small effect size d=0.38.<br><br>2. BFI Fatigue Severity reduced in the control group (4.3 to 4.8) and

For peer review only

Study Number	Author & Year/ Country	Aim Design Theoretical model	Sample	Intervention(s)	Outcomes/measures and follow-up period	Results
				Second session focussing on individual progress.<br><br>Control: Education module on generalised activity pacing delivered by an occupational therapist with advice to implement the strategies. Second session focussing on individual progress.	Data collected at baseline and 10 week follow up	the intervention group (4.1 to 3.3). The difference between groups was not statistically significant (p=0.09) with a moderate to large effect size (d=0.79) BFI Fatigue Interference increased in the control group (3.6 to 4.2) and decreased in the intervention group (3.1 to 1.6). The difference between groups was statistically significant (p=0.02) with a large effect size (d=1.10)
43	Wolff et al (2010) (75)<br><br>USA	Determine whether guided care (GC) improves patients' primary caregivers' depressive symptoms, strain, productivity and perceptions of quality of care for care recipients.<br><br>Clustered RCT	N=308 primary caregivers/patient dyads<br>n= 156 intervention caregivers (mean age 60.9 years)/patient (mean age 78.0 years) dyads randomised to	Guided Care (GC)provided by nurses: included training and supporting patient's family caregivers. Designed to address deficiencies in the quality of chronic care delivery by facilitating coordinated, comprehensive, evidence-based heath care for multimorbid adults.	Primary outcomes:<br><br>1. Caregiver depressive symptoms: Centre for Epidemiological Studies (CES-D)	At 18 months follow-up:<br><br>1. CES-D changed from 6.4 to 6.8 in the GC compared with 7.1 to 5.8 in the UC. The results were not statistically significant between groups<br><br>2. CSI increased from 6.5 to 6.7 in the GC

For peer review only - http://bmjopen.bmj.com/site/about/guidelines.xhtml

Study Number	Author & Year/ Country	Aim Design Theoretical model	Sample	Intervention(s)	Outcomes/measures and follow-up period	Results
			Guided Care (GC) n=152 usual care caregiver (mean age 61.6)/patient (mean age 77.9) dyads (UC) n=22 usual care, mean age 31.91 (SD=6.52), male gender n=21 (95.5%). Participants recruited within 14 primary care physician teams (PCP)	GC nurses collaborated with patients PCP to provide clinical processes: assessing the patient at home, creating an evidence-based care plan, promoting patient self-management, proactively monitoring patient condition, coaching the patient to practice healthy behaviours, coordinating patients transition between sites and providers of care, facilitating access to community resources, and educating and supporting patients family caregivers.<br><br>Comparison group received usual care (details not provided).	2. Caregiver strain: Modified Caregiver Strain Index (CSI)<br><br>3. Quality of Chronic Illness Care: modified version of the Patient Assessment of Chronic Illness Care (PACIC)<br><br>4.Caregiver Productivity Loss: Work Productivity and Activity Impairment questionnaire (WPAI:CG)<br><br>Baseline and 18-month follow-ups.	group and 6.6 to 7.7 in the UC group. These results were not statistically significant between the two groups.<br><br>3.Aggregate QoL was higher in the GC group compared with the usual care group (0.40; p<0.001)<br><br>4. Work productivity loss was more substantial in the GC group compared with the UC group (14.6% to 8.4% vs 18.2% to 16.1%). Presentism declined from 16.7% to 11.9% in the UC group compared with 12.9% to 5.3% in the GC group.
44	Dobscha et al (2009) (76)<br><br>USA	To assess whether a collaborative intervention can improve chronic pain-related outcomes in a Department of Veteran	N=401 patients at 5 primary care clinics with moderate or severe chronic pain	Intervention: clinicians in intervention practices undertook two 90 minute workshops including abbreviated training in shared decision making skills and chronic pain	Primary Outcome: 1. Self-reported pain disability: Roland Morris Disability Questionnaire for pain (RMDQ) score Additional main outcomes:	1. Greater improvement from baseline to 12 months in intervention group than control (-1.4 vs -0.2, p=0.004).

For peer review only

For peer review only - http://bmjopen.bmj.com/site/about/guidelines.xhtml

Study Number	Author & Year/ Country	Aim Design Theoretical model	Sample	Intervention(s)	Outcomes/measures and follow-up period	Results
		Affairs (VA) primary care setting.<br><br>Cluster RCT	n=187 intervention group, mean age 62.1 (SD 11.2) n= 214 control group, mean age 61.3 (SD 12.3)	education. Patients received an assessment with a care manager to develop individualised functional goals and a treatment plan was communicated to the clinician. Patients were invited to a four session workshop based on the brief activating approach. Care managers contacted patients every 2 months for 12 months to provide support and reassess goals and activities.<br><br>Control: treatment as usual including referral to speciality pain clinic, ancillary services such as physiotherapy and occupational therapy.	2. Depression severity: PHQ-9<br><br>3. Pain intensity: CPG Pain Intensity subscale<br><br>Secondary outcomes:<br>4. CPG Pain interference subscale<br><br>5. Patient rated global impression of change<br><br>6. Global VA health care satisfaction<br><br>7. Health related quality of life: EQ-5D	2. Greater improvement from baseline to 12 months in IG than CG (-3.7 vs -1.2, p=0.003).<br><br>3. Greater improvement from baseline to 12 months in IG than CG (-4.7 vs -0.6, p=0.01).<br><br>Secondary outcomes:<br>4. Improvement from baseline to 12 months in IG and worsening in CG (-5.7 vs 2.3, p=0.03)<br><br>5. Greater improvement in IG than CG at 12 months (3.7 vs 4.4, p<0.01)<br><br>6. No difference in change from baseline to 12 months in IG and CG (-0.27 vs -0.36, p=0.44)<br><br>7. No difference between IG and CG in change from baseline to 12

For peer review only - http://bmjopen.bmj.com/site/about/guidelines.xhtml

Study Number	Author & Year/ Country	Aim Design Theoretical model	Sample	Intervention(s)	Outcomes/measures and follow-up period	Results
					8. Effectiveness of VA chronic pain treatment Outcomes collected at baseline, 3, 6 and 12 months.	months (-0.02 vs -0.04, p=0.17)<br><br>8. No difference in change from baseline to 12 months in IG and CG (0.33 vs 0.2, p=0.64)
45	Machado et al, (2007) (77)<br><br>Brazil	To compare effectiveness of psychotherapy based on client-centred therapy and exercise for patients with chronic nonspecific low back pain<br><br>RCT.	N=33 participants with nonspecific low back pain (LBP)<br>n=16 intervention, mean age 44.6 (SD=12.1) years.<br>n=17 control, mean age 42.4 (SD=13.2) years.	Psychotherapy based on the principles of nondirective counselling. Patients in groups attended 80 minute treatment sessions twice a week for 9 weeks. Therapists provided support as patients discussed life stressors, including chronic pain.<br><br>Control group received Physiotherapists-led exercise therapy. General exercise consisting of 20 minute walking, general stretching, and strengthening of the bridge (lying supine with knees flexed, raising hips and hold for 5 seconds, repeating the procedure for 15 minutes).	1. Disability: Brazil Roland-Morris Questionnaire (BRM)<br><br>2. Pain: Visual Analogue Scale (VAS)	1. Exercise group showed lower disability at 9 weeks compared with the psychotherapy group (-4.9 points difference; p=0.02), at 6 months (4 points difference; p=0.13)<br><br>2. Pain scores were not significantly lower in the exercise group compared with psychotherapy group at nine weeks (-1.8; p=0.27)<br>At six months the exercise group again scored lower compared with the psychotherapy group (-1.3; p=0.38).

For peer review only - http://bmjopen.bmj.com/site/about/guidelines.xhtml

Study Number	Author & Year/ Country	Aim Design Theoretical model	Sample	Intervention(s)	Outcomes/measures and follow-up period	Results
				Patients attended the 40 minute sessions in groups, twice a week for 9 weeks.	3. Depressive symptoms: Beck Depression Inventory (BDI)<br><br>Assessments conducted at baseline, 9 weeks and 6 months (depression was not assessed at 6 months).	3. Exercise group showed less depressive symptoms compared with the psychotherapy group at nine week (-6.3 points difference; p=0.29).
46	Glasgow et al (2005) (78)<br><br>USA	To determine if an interactive computer technology intervention designed to improve patient centred communication improves diabetes care.<br><br>Cluster RCT	N=886 adults with Type 2 Diabetes under the care of 52 primary care physicians<br>n=469 intervention group, mean age 62 (SD 1.4)<br>n=417 control group, mean age 64 (SD 1.3)	Intervention: Before two appointments, 6 months apart, patients completed computerized touch screen assessments including recall of clinical interventions and developing a self-management action plan. Received detailed personalised printout of results. Patients met a Care manager trained in patient centred self-management approaches to review care needs and self-care goals followed by a follow-up call after each visit.	Primary outcome:<br>1. Patient reports of receiving American Diabetes Association recommended laboratory screenings and recommended patient centred care activities<br>Secondary outcomes.<br><br>2. Diabetes quality of life (The revised Problem Area in Diabetes 2 Scale, PAID-2)<br><br>3. HbA1c	Primary outcome:<br>1. intervention group had greater improvement in laboratory screenings completed than controls (F=11.6, p<0.001) and patient centred activities (F=39.5, p<0.001).<br><br>2. No significant difference between intervention and control groups at 12 months (27.4 VS 27.5, p=0.964).<br><br>3. No difference in HbA1c between

For peer review only - http://bmjopen.bmj.com/site/about/guidelines.xhtml

Study Number	Author & Year/<br><br>Country	Aim<br>Design<br>Theoretical model	Sample	Intervention(s)	Outcomes/measures and follow-up period	Results
				Control: Completed the same touch screen computer assessment but received a print- out of general health risks. No meetings or calls from care manager but same number of physician appointments.	4. Total cholesterol to HDL cholesterol ratio.<br><br>5. Depression (Patient Health Questionnaire, PHQ-9, % with 10 or higher).<br><br>Outcomes measured at baseline and 12 months.	intervention and control groups (7.11 vs 7.17%, p=0.571.<br><br>4. No difference between intervention and control groups (4.11 vs 4.15, p=0.733).<br><br>5. No difference between intervention and control groups (12.3 vs 13.9%).
47	Mills et al (2003) (79)<br><br>Australia	Geographically controlled study	N=509 people with Type 2 Diabetes in rural Australia n=398 intervention n=111 control	Intervention: Care planning using a patient centred care planning model. Emotions, thoughts and behaviours translated into patient specific problem statements then goals. Care plans created and reviewed annually. Relevant health services were scheduled in line with best practice. Patients were followed for two years at minimum 6 month intervals.	1.Problem and goal scores recorded on linear analogue scale recorded by patients and service co-ordinators<br><br>2.Work and social adjustment: Work and Social Adjustment Scale (WASAS) at each visit.	1.Up to 60% of IG felt their main problem improved by the end of the trial. 40-60% of patients made some progress toward achieving their first goal.<br><br>2. The WASAS scores between the two groups were statistically significant (P < 0.01) over time, with

For peer review only - http://bmjopen.bmj.com/site/about/guidelines.xhtml

Study Number	Author & Year/ Country	Aim Design Theoretical model	Sample	Intervention(s)	Outcomes/measures and follow-up period	Results
				Control: usual care in rural Southern Australia	3.Medical Outcomes Study 36-Short Form (SF36).<br><br>4.Emergency and elective admission rates	mean scores improving 10%.<br><br>3.Statistically significant difference (p<0.01) between IG and CG in SF 36.<br><br>4. IG group hospital admission rate fell 18.2% compared to CG.
48	Kennedy, et al (2003) (80)<br><br>UK	To evaluate the effects of a PC intervention on clinical outcomes and health service use among patients with inflammatory bowel disease (IBD).<br><br>Multicentre cluster RCT.	N=19 hospitals, outpatient (n=9 treatment, n=10 control).<br>n=635 patients with inflammatory bowel disease (IBD)<br>n=270 intervention (mean age 44.4, sd=14.9)<br>n=365 control (mean age 46.3, sd 15.1)	Clinicians at the intervention sites received a 2-hr training session led by an expert in postgraduate medical education using role play and video feedback titled 'patient-centred consultation in gastroenterology'. Training focused in PC medicine principles and applied to self-management in IBD. Patients at the intervention sites participated in PC consultations conducted by clinicians. A self-management plan was negotiated and written into	1. Hospital appointments<br><br>2. Quality of life: Inflammatory bowel	1. The number of kept appointments reduced by app. one third in the intervention group compared with the control group (difference -1.4; p<0.001). The mean number of clinic non-attendances per person during the trial was also lower for the intervention group (difference -0.08; p=0.034).<br><br>2. IBDQ did not differ significantly between the

For peer review only - http://bmjopen.bmj.com/site/about/guidelines.xhtml

Study Number	Author & Year/<br><br>Country	Aim<br>Design<br>Theoretical model	Sample	Intervention(s)	Outcomes/measures and follow-up period	Results
				the guidebook. Patients were instructed to call a specified number if they needed to schedule an appointment according to circumstances listed in the guidebook.<br><br>Patients at the control sites received management processes deemed appropriate by hospital specialists.	disease questionnaire (IBDQ)<br><br>3. Anxiety and depression: Hospital Anxiety and Depression Scale (HADS)<br><br>4. Patient enablement: patient enablement instrument (PEI)<br><br>5. Satisfaction : Consultation satisfaction questionnaire (CSQ).	two groups (difference 1.94; p=0.45)<br><br>3. HADS did not differ significantly between two groups (difference -0.35; p=0.40)<br><br>4. the intervention group showed a higher enablement score (difference 0.90; p=0.026)<br><br>5. satisfaction did not differ significantly between the two groups (3.47; p=0.09).
49	Martin et al, (2004) (81)<br><br>New Zealand	To test whether individualised care plan for patients experiencing acute exacerbations of COPD result in reduced health care utilisation and improved quality of life<br><br>RCT.	N=93 COPD patients<br>n=44 intervention group, mean age 71.1 years.<br>n=49 control group, mean age 61.9 years.	Individualised care plan based on an interview between patient and respiratory nurse, review of hospital records by respiratory specialist and by patient's own GP. Each patient was given instructions about how to use the plan by the respiratory nurse. Copies of the plan were held by	Primary outcome:<br>1.Utilisation of primary care services and hospital admissions	1. Intervention group called out the ambulance service more frequent (2.8 vs 1.1) calls per 12 months (p=0.03).<br>Intervention group had more GP visits compared with control group (15.6 vs 11.6) in 12 months; p=0.08<br>The intervention group has more hospital

For peer review only - http://bmjopen.bmj.com/site/about/guidelines.xhtml

Study Number	Author & Year/ Country	Aim Design Theoretical model	Sample	Intervention(s)	Outcomes/measures and follow-up period	Results
				patient, GP, ambulance service, emergency department and after hour's surgery.<br><br>Control group received usual care. They did not have an individualised care plan.<br>All participants remained under the care of their own GP.	2. Quality of Life: St George's Respiratory Questionnaire (SGRQ)<br>Outcomes assessed at baseline, three, six and 12 months.	admissions compared with the control group (1.1 vs 0.7); p=0.17.<br><br>2. SGRQ did not differ significantly between groups (57.3,sd=13.5 for intervention and 55.1, sd=14.6) for control.
50	Alamo, et al, (2002) (82)<br><br>Spain	To assess whether patient-centred consultations are more effective than usual care style of consultations among patient with chronic musculoskeletal pain and fibromyalgia<br><br>Clustered RCT	N=20 GP's in 13 health centres.<br>N=110 patients<br>n=10 GP's intervention, n=10 GP's control.<br>N=63 (mean age 39.2; sd=7.6 years) patients intervention<br>N=47 (mean age 42.3; sd=10) patients control	GP's in the intervention received training on communication skills necessary to undertake PC approach. These focused on active listening, asking patients' to express their fears and concerns, offering reassurance, coming up with a management plan together with the patient.<br><br>Control group GP's provided usual care	1. Pain intensity: VAS and pain scale of the Nottingham health profile (NHP) questionnaire<br><br>2. Number of tender points and subjective	Pain reduced in the intervention group (mean pain at baseline 3.4 (sd=1.2), at 6 months 3.3 (sd=1.0) and at 12 months 3.1 (sd=1.0). Mean pain in the control group was 4.1 (sd=0.8), at 6 months 3.9 (sd=0.8) and at 12 months 3.9 (sd=0.8). The difference between the two groups was not statistically significant (p=0.73)<br><br>2.Number of tender points reduced significantly in the

For peer review only

Study Number	Author & Year/<br><br>Country	Aim<br>Design<br>Theoretical model	Sample	Intervention(s)	Outcomes/measures and follow-up period	Results
					health status: NHP questionnaire<br><br>3. Psychological disturbance: Goldberg Scale of anxiety and depression (GHQ)<br><br>Participants were followed-up at 6 and 12 months.	intervention group compared with the control group (p=0.05)<br><br>3.GHQ anxiety significantly reduced in the intervention compared with the control group (p=0.04) GHQ depression was not statistically significant (p=0.33)
51	Sommers et al. (2000) (83)<br><br>USA	To examine the impact of an interdisciplinary, collaborative practice intervention involving a primary care physician, a nurse, and a social worker for community-dwelling seniors with chronic illnesses<br><br>Concurrent, controlled cohort study	N=543 patients aged 65 or older under treatment for at least 2 chronic conditions. Recruited from 18 private primary care physician offices n=280 intervention group, mean age 78 (SD 6.8)	Intervention: home assessment from a nurse or social worker including listening to health concerns, home safety check and functional assessment. Creation of risk reduction plans and treatment plans based on chronic disease self-management strategies. Follow up sessions at least every 6 weeks including telephone, home visit, small group sessions or office or hospital visit.	Utilisation of medical services at baseline, 1 and 2 years<br>1. Change in number of hospital admissions per patient per year<br><br>2. Change in percentage of patients with 1 or more hospital readmissions within 60 days<br><br>3. Change mean number of visits to all physicians	1. Statistically significant reduction in admissions in IG vs CG (-0.02 vs 0.18, p=0.03)<br><br>2. Statistically significant reduction in readmissions in IG vs CG (-2.0 vs 5.4, p=0.03)<br><br>3. Statistically significant reduction in visits in IG

For peer review only

Study Number	Author & Year/ Country	Aim Design Theoretical model	Sample	Intervention(s)	Outcomes/measures and follow-up period	Results
			n=263 control group, mean age 77 (SD 6.6)	Control: usual care from the primary physician	4. Change in percentage of patients with 1 or more visits to the emergency department<br><br>5. Change in proportion of patients with 1 or more home care visits<br><br>6. Change in number of patients with 1 or more nursing home placements<br>Patient reported health status at baseline, 1 and 2 years.<br><br>7. Change in Health Activities Questionnaire<br><br>8. Geriatric Depression Scale<br><br>9. Medications count	vs CG (-1.5 vs 0.5, p=0.003)<br><br>4. No difference in change between IG and CG (1.2 vs -0.66, p=0.77)<br><br>5. No difference in change between IG and CG (1.8 vs -2.6, p=0.81)<br><br>6. No difference in change between IG and CG (5.0 vs -5.4, p=0.59)<br><br>7. No difference in change between IG and CG (0.03 vs 0.08, p=0.14<br><br>8. No difference in change between IG and CG (0.3 vs 0.5, p=0.52)<br><br>9. No difference in change between IG and CG (0.3 vs 0, p=0.26)

For peer review only

Study Number	Author & Year/ Country	Aim Design Theoretical model	Sample	Intervention(s)	Outcomes/measures and follow-up period	Results
					10. Social activities count<br><br>11. Symptom scale<br><br>12. SF-36 self-rated health<br><br>13. Nutrition checklist	10. Significant increase in IG vs reduction in CG (0.2 vs -0.3, p=0.04)<br><br>11. No significant change in IG vs CG (-0.5 vs 1.0, p=0.08)<br><br>12. No significant change in IG vs CG (0 vs 0.1, p=0.08)<br><br>13. No significant change in IG or CG (0.3 vs 0, p=0.12)
52	Gustafson et al (1994) (84)<br><br>USA	Test the impact of an interactive, computerised, personal health support system on adults with HIV<br><br>RCT	N=107 in intervention group, mean age 34.8 years n=97 in control group, mean age 34.5 years	Intervention: Participants were given a PC based Comprehensive Health Enhancement Support System (CHESS) in their homes for 6 or 3 months. This enables access to health information, asking experts questions anonymously and reading personal accounts of others with similar problems.	1.Quality of life scores: Medical Outcomes Survey (MOS) at baseline, 2 and 5 months	1.At 2 months the intervention group reported significantly improved cognitive functioning (p=0.053), more active lives (p=0.013), decreased negative emotion (p=0.013) and better social support (p=0.074) than controls. Depression, physical

For peer review only - http://bmjopen.bmj.com/site/about/guidelines.xhtml

Study Number	Author & Year/ Country	Aim Design Theoretical model	Sample	Intervention(s)	Outcomes/measures and follow-up period	Results
				Control: no details provided		function, energy and participation in healthcare did not show significant differences between groups. At 5 months the intervention group reported more active life (p=0.034), improved social support (p=0.017) and more active participation in their healthcare (p=0.020). There was no difference between groups in cognitive function, negative emotions, depression, physical function, or energy.
					2.Use of ambulatory care services in 2 months before and after intervention implementation	2. No difference in frequency of visits to ambulatory care services between groups. Intervention group reported shorter visits than controls during the intervention (p=0.043) and were more likely to telephone providers both during (p=0.013) and

For peer review only

Study Number	Author & Year/ Country	Aim Design Theoretical model	Sample	Intervention(s)	Outcomes/measures and follow-up period	Results
					3.Hospitalisation before, during and after intervention implementation	after (p=0.094) the intervention.<br><br>3.Hospitalisations were lower for the intervention group than controls during the intervention (p=0.020) and shorter (p=0.009). These differences were not maintained after the intervention.

For peer review only

Study Number	Author & Year/ Country	Aim Design Theoretical model	Sample	Intervention(s)	Outcomes/measures and follow-up period	Results
53	Kinmonth et al (1998) (85) , UK	To assess the effect of additional training of practice nurses and general practitioners in patient centred care on lifestyle, psychological and physiological status of patients with type 2 diabetes. Pragmatic parallel group design, randomisation between practice teams to routine care. RCT.	N=41 practices n=21 intervention practices and 142 patients n=20 usual care practices and 108 patients. 250/360 patients (30-70 years) Mean age 41.54(SD=9.83) years.	1.5 days group training for the nurses and 0.5 days for doctors:<br><br>Reviewed evidence-based person-centred consulting and practised the skills they learnt with an experienced facilitator. Skills included active listening and negotiation of behavioural change. They produced materials including a booklet for patients, 'Diabetes in your hands' which encouraged patients to ask questions.<br><br>Comparison group nurses were offered similar support sessions focusing on use of guidelines and materials.	1. Quality of life: Audit of diabetes dependent quality of life (ADDQoL)<br><br>2. Communication and satisfaction with treatment<br><br>3.Wellbeing: The wellbeing questionnaire<br><br>4. Blood pressure<br><br>5. Body mass index (kg/m^2)<br><br>6. Haemoglobin A1c %	1. QoL mean in the intervention -1.09 and -1.23 in the control group (p=0.27).<br><br>2. Intervention showed better communication with doctors (odds 2.8 p<0.001), satisfaction with treatment (1.6 p=0.05)<br><br>3. Wellbeing: mean difference 2.8 (p=0.03)<br><br>4. Mean systolic BP 144.3 in the intervention and 142.8 in the control groups p=0.18 Diastolic BP 89.0 in the intervention and 87.2 in the control p=0.10<br><br>5. Mean BMI 31.3 in the intervention and 29.5 in the control p=0.03.<br><br>6. Mean HbA1c 7.07 in the IF and 7.17 in the control group (p=0.31).

For peer review only - http://bmjopen.bmj.com/site/about/guidelines.xhtml

Study Number	Author & Year/ Country	Aim Design Theoretical model	Sample	Intervention(s)	Outcomes/measures and follow-up period	Results
54	Landefeld (1995) (86), USA	To compare outcomes of people admitted to a unit especially designed to improve the functional outcomes of acutely ill older patients with standard care<br><br>RCT	n=651 people aged 70 or older admitted for general medical care at a teaching hospital<br>n=327 intervention group, mean age 80.2 (SD)<br>n=324 control group, mean age 80.1 (SD 6.6)	Intervention: Admission to a unit practising the Acute Care for Elders programme including a specially prepared environment, patient-centred care emphasizing independence, discharge planning aiming to discharge patients home and intensive review of medical care to minimise adverse effects of interventions and procedures.<br><br>Usual care: admission to acute care medical unit.<br>In both groups patients were assigned a primary nurse, two resident physicians and an attending physician. Staffing ratios and access to hospital support services including social work, physiotherapy, and nutrition.	Primary outcome:<br>1. Change from admission to discharge in the number of basic activities of daily living (ADLs) that the patient could perform independently<br><br>Secondary outcomes<br>2. Patients admitted from own home being discharged to a long-term care institution<br><br>3. Overall health status at discharge<br><br>4. Mean length of hospital stay<br><br>5. Mean total hospital charges	1. IG had greater improvement compared to CG (p=0.009)<br>The mean ADLs performed independently at discharge were 3.6 for IG and 3.3 for CG (p=0.05)<br><br>2. Fewer IG patients discharged to institution than CG (14% vs 22%, p=0.01)<br><br>3.Better health status in IG than CG (p<0.001)<br><br>4. Not significant<br><br>5. Not significant

Characteristics of the included studies

The n=54 studies included were conducted in 17 countries, the majority were high-income (n=13/17). Studies were conducted predominantly in Sweden n=16 (22, 25, 28-34, 36, 40-46, 50-58, 60, 61, 67, 68, 87), USA n=11 (26, 65, 70, 72, 74-76, 78, 83, 84, 86, 88), UK=4 (63, 80, 85), Germany n=4 (38, 73, 89, 90), Hong Kong=3 (47, 59, 64), Australia n=3 (37, 71, 72, 79), Spain n=2 (82, 91) and, Canada n=2 (39, 72, 92),. One study was conducted in each of the following countries: Iran (35), Kenya (69), Denmark (66) , Norway (23), Singapore (93) , Thailand (49), New Zealand (81), Brasil (77), and Netherlands (62). A further study was multi-country, conducted in Canada, Australia, and USA (72).

Study designs

Of the included studies, n=44 were randomised controlled trials (RCT) (23, 88, 90, 92, 94), pre-and post-test experimental/controlled before and after design (25, 52, 54, 55, 83, 95), quasi-experimental study designs (26, 31, 57, 58), a comparative study (70) and a geographically controlled study (79). Of the n=44 RCT's, n=10 were clustered trials (38, 62, 75, 76, 78, 80, 82, 85, 92, 96).

Diagnostic groups

The interventions addressed the following diagnostic groups: n=12 heart failure (32, 34, 41-45, 52, 60, 61, 66, 97), n=8 T2D (49, 56, 59, 63, 78, 79, 85, 92), n=7 COPD (34, 36, 47, 62, 65, 81, 91), n=5 cancer (31, 39, 46, 71, 73), n=5 multimorbidity (23, 37, 38, 83, 88), n=3 fibromyalgia (50, 51, 82), n=3 rheumatoid arthritis (22, 40, 67), n=2 HIV (69, 84), n=1 back pain (77) n=1 inflammatory bowel disease (80), n=1 osteoarthritis (74), n=1 Stroke (28), n=1 chronic pain (76), n=1 dementia (89), Parkinson disease (90) and n=1 beta-thalassaemia major (35).

For peer review only - http://bmjopen.bmj.com/site/about/guidelines.xhtml

Intervention target & delivery

The interventions were nurse-led (22, 65, 69-71, 75, 81, 89, 93), nurse and physiotherapist-led (34), nurse, physician and social worker-led (23, 60, 83).

The targets of the interventions were patient and caregiver dyads (49, 64, 75, 91) or delivered to both patients and health professionals (56, 62, 76, 80, 82, 92) in T2D (56, 92), chronic pain/fibromyalgia (76, 82), COPD (62) and inflammatory bowel disease populations (80). The interventions were technology-based involving a tablet computer or mobile phone (47, 59, 71, 78, 91-93), or delivered to professionals such as doctors, nurses, social workers (41, 42, 73, 85, 92) working with patients with heart failure (41, 42), T2D (85, 92) and cancer (73).

Intervention components and delivery

Interventions delivered to health professionals (nurses, doctors, physiotherapists) consisted of training, mentorship and support through lecturers, seminars and/or workshops in the philosophy and delivery of person-centred care (22, 25, 26, 28, 32-34, 36, 41, 56, 62, 63, 69, 70, 72, 73, 76, 80, 81, 85, 89, 92, 97) for example clinical consultations using person-centred approach, person-centred communication and patient centred self-management approach (31, 36, 56, 73, 78, 80, 81, 85, 88, 91). Health professionals then implemented what they learnt as they provided care to the patients and/or families.

Interventions delivered to patients and/or caregivers consisted of information provision, education, and training (31, 35, 47, 49, 57-59, 63-67, 74-77, 91). The interventions were either individualised and delivered face-to-face (47, 56) or delivered in groups (56, 65, 77). Educational materials, information leaflets, booklets, brochures were provided to participants (25, 31, 59, 63, 70, 85). Some interventions delivered to patients focused on developing or creating a health plan. Participants identified or set aims or goals with targets to achieve and patients identified resources and tools to achieve the targets. Health professionals worked with the patients to achieve the targets and care was provided in line with patient needs and

For peer review only - http://bmjopen.bmj.com/site/about/guidelines.xhtml

wants and what matters to them (23, 26, 34, 36-38, 40, 41, 46, 50, 52, 54, 55, 57, 58, 60, 76, 79-81, 83, 88, 92, 93, 95, 97). The health plan was reviewed and revised when necessary. Interventions were delivered either in nursing homes (25) primary care/outpatient care (22, 23, 37, 38, 40, 46, 47, 69, 76, 91, 92), surgical departments (31, 39, 57, 58, 71), inpatient facilities (28, 32, 33, 52, 54, 55, 86, 95) or in home and/or community settings (26, 28, 49, 50, 60, 61, 65, 83, 84, 88, 90, 91, 93).

Some interventions involved using mobile technology (36, 46, 47, 49, 71, 80, 83, 93), mobile app (39) to contact patients at home. In one study patients in the intervention arm used either mobile-based or web-based eHealth tool preinstalled or downloaded it to use on their own mobile (45) or a tablet computer to self-monitor blood glucose and blood pressure (59), or to complete self-assessments using a computer touch screen and to develop a self-management action plan (78).

Methodological quality of the studies

The majority of the studies (n=39) stated the method of randomisation, although this was not clearly stated in n=13 studies (28, 39, 60, 63, 66, 75, 76, 78-82, 91). Twenty-five studies achieved allocation concealment, however n=19 did not clearly state allocation concealment (28, 35, 41, 60, 63, 64, 66, 70, 74, 75, 79-83, 86, 88, 91, 92). Blinding of participants was reported in only three studies (37, 49, 76). Blinding of outcome assessors was reported in n=17 studies (34, 35, 37, 47, 49, 64-66, 72, 74-77, 82, 88, 93), while n=20 studies did not clearly state if outcome assessors were blinded. With respect to follow-up data collection, n=32 studies retained at least 80% participants to the final point of data collection. In n=19 studies details were lacking regarding what constitutes usual care (25, 34, 35, 38, 40, 52, 61, 64, 66, 71, 75, 79, 81-84, 89, 91, 93). The following studies included all participants including those who withdraw from the study in data analysis (34, 35, 41, 47, 49, 73, 80, 82, 87, 98).

For peer review only

Outcomes assessed

For patient outcomes quality of life was reported in n=21 studies (32, 34, 38, 47, 49, 60-62, 65, 69, 76, 78-81, 83, 85, 90-92). These studies were conducted in COPD (34, 47, 62, 65, 81, 91), T2D (49, 78, 79, 85, 92), heart failure (60, 61), chronically ill elderly (38, 83), HIV (69), acute respiratory syndrome (32), chronic pain (76), Parkinson's disease (90) and Inflammatory bowel disease (80) populations.

General symptom burden was reported in n=3 studies in heart failure, chronically ill elderly, and cancer (61, 72, 83). Fatigue symptom was reported in n=4 studies among patients with rheumatoid arthritis, COPD, stroke, chronic illnesses (elderly populations) (27, 34, 40, 88). Dyspnoea symptom was reported in n=3 COPD studies (34, 47, 65), while only one study reported data on sleep disturbance (88).

Pain outcomes (severity/intensity, interference and disability) were reported in nine studies (67-70, 74, 76, 77, 82, 88), among patients with chronic inflammatory arthritis (67, 68, 74), chronic pain, low back pain, infected chronic ulcers (76, 77), HIV (69), multiple chronic diseases (88), and fibromyalgia (82). Another nine studies reported data on communication and satisfaction with treatment (28, 34, 38, 39, 67, 68, 76, 80, 85).

Self-management and related outcomes were reported in the following studies: T2D self-management (49), COPD self-management and co-morbidity (65), enablement (80), Patient confidence in managing coronary heart disease and obtaining rheumatology care (41, 68, 70), self-efficacy (34, 37, 41, 49, 88), change from admission to discharge in the number of basic activities of daily living (ADLs) that the patient could perform independently (86), performance in activities (34, 68), patient reported health status and change in health activities (83), and health education impact (37).

The main psychosocial outcomes and concerns reported were psychiatric morbidity (69), psychological disturbance (68, 82), concerns and wellbeing (85), anxiety and

For peer review only - http://bmjopen.bmj.com/site/about/guidelines.xhtml

depression/mood (34, 65, 72, 76-78, 80, 88), Motor function (90), primary emotions (80). Distress (71, 88, 92), and decisional conflict (92).

Caregiver outcomes assessed were depressive symptoms, caregiver strain, caregiver productivity loss (75), caregiver quality of life (26, 64), caregiver burden was assessed in n=2 studies (28, 64). Other caregiver outcomes were informal care that is percentage reported providing assistance with personal ADLs (28), participation in everyday occupations and Life satisfaction (28).

Health care professional outcomes included job strain (89), transition to palliative care, general communication, involvement of significant others (73), GP's knowledge about medication taken by the patient (38)), and intention to engage in Interprofessional Shared Decision Making (92).

Data on costs and utilisation of services

Six studies reported data on costs of health care services (32, 52, 60, 67, 86, 91), and four on number of hospital appointments (80, 81, 83, 99). Two studies reported data on hospital admissions (81, 83), and two studies reported length of hospital stay (86, 99). Seven studies reported data on unplanned readmissions, emergency room attendance (71, 79, 83, 91, 93, 98, 99), and four studies reported health care utilization (37, 38, 83, 98), and medications count (change in number of medications taken by the patient) (38).

Data on clinical outcomes

Clinical outcomes assessed were systolic and diastolic blood pressure (56, 59, 85), fasting blood sugar, HbA1c (49, 56, 59, 63, 78), Body mass index, Haemoglobin (56, 85), Lung function FEV_1/FVC ratio, exercise capacity, (47), total cholesterol to HDL cholesterol ratio (78), serum ferritin, iron level, total iron binding capacity (35), mortality (47, 65, 93, 98).

For peer review only - http://bmjopen.bmj.com/site/about/guidelines.xhtml
For peer review only

Synthesis of the findings

We synthesise the findings using methods of narrative synthesis in systematic reviews (100). A narrative synthesis is presented based on the model which informed the intervention, interventions elements/components, study population and intervention, study design (RCT or non-RCT) and outcomes.

Theoretical model/framework used by the study

The majority of the studies (n=34) did not report which theory or model informed the design or delivery of the interventions (23, 26, 35, 37-39, 47, 59, 62-64, 66, 70-86, 89-93). One study was informed by the Theory of Hernandez (56), three studies were developed and designed based on Bandura's self-efficacy theory (34, 49, 88), and another study used the person-centred palliative care model, Six S: self-image, self-determination, social relationships, symptom control, synthesis and surrender (60, 61). Person-centred care according to the framework by the Gothenburg Centre for Person-Centred Care (GPCC) informed most of the studies conducted in Sweden (22, 28-33, 36, 38, 40-43, 46, 50-55, 57, 58, 97).

Mechanism of action of the interventions.

For the GPCC model which involved three main parameters (initiation of partnership between the patient/caregiver and health professional, implementing the partnership and documenting/safeguarding the partnership). This model was applied across different settings and populations. It also involved both patients and health professionals in developing and designing the intervention and implementation.

Person-centred care requires ongoing systematic engagement between the patient and health professionals. Furthermore, requires to be adapted to each patient population (cancer, HIV, COPD, T2D etc) and context (primary care, outpatient, residential homes, emergency care, hospital, rehabilitation etc). Care plans, goals of care discussed and

For peer review only

For peer review only - http://bmjopen.bmj.com/site/about/guidelines.xhtml

revised as necessary continuously. Communication is also an important component in the GPCC model. Communication offered by the GPCC model gives patients (for example inpatient setting) information and confidence about care processes and self-management of their own problems and concerns. This leads to understanding of the discharge processes and readiness and eagerness to return home which promotes self-efficacy. For the theory of Hernandez, self-efficacy and all other studies which did not state the theoretical framework, their mechanism of action were similar with the GPCC because they either had a self-management component or self-efficacy and were aimed at empowering the patient or caregiver or improving communication between the patient and the health professional. We summarise narrative synthesis below based on self-management component of the interventions.

Interventions comprising of a self-management component

Thirteen RCTs consisted of a self-management intervention or component. These were conducted in COPD (34, 47, 65), T2D (49, 56, 63, 91), elderly with chronic conditions (37, 83, 88), cancer (46), IBD(80), multimorbidity (75) populations. All the self-management interventions were educational and consisted of training of patients and/or caregivers (34, 37, 46, 47, 49, 75, 83, 88, 91) or both health care professionals and patients/caregivers (56, 63, 75, 80). Educational sessions were either group-based (49, 56, 63, 75, 80, 83) or individualised/face-to-face (47, 65). Four of the thirteen studies examined effects of the intervention on hospital admissions (47, 65, 83, 91). Three studies showed positive benefits of self-management interventions in reducing hospital admissions. One of these four studies assessed mortality (65), another one length of stay in the hospital (47) while one study assessed unplanned visits to the hospital (91). All studies reported positive benefits of the intervention in reducing mortality, length of hospital stay and unplanned visits. Six of the thirteen studies assessed quality of life outcomes (46, 47, 49, 65, 75, 80). In three studies QoL was assessed using the St George's Respiratory Questionnaire (47, 65, 75) and the results were significant. One study used the HRQoL measure and the results were non-

For peer review only

significant, but significant on specific problems such as swallowing, social eating and feeding (46). Two studies reported non-significant results and assessed quality of life using the IBD questionnaire (80) and the Thai Version short-form Health Survey (49). HADS was used in three studies (34, 65, 80) but only one reported significant findings (65) and two reported non-significant findings (34, 80). Self-efficacy was assessed in four studies (34, 37, 49, 88) with only one study reporting significant results (49). Knowledge on self-management was reported in two studies, T2D (49) and COPD (65) populations, with both studies reporting significant differences between the intervention and control groups (49, 65).

Technology based interventions

Twelve studies used technology. These were conducted among patients with T2D (49, 59, 78, 92), cancer (39, 46, 71), COPD (36, 47, 91), chronic disease among elderly (83), and IBD (80). Two of these studies were informed by the GPCC model (36, 46) and one was informed by Bandura's model (49). The rest were not informed by a theoretical model. Most of these technology-based intervention studies used a telephone-based intervention (36, 46, 47, 49, 71, 83). Only one study used a mobile app (39), four used tablet or computer technology (59, 78, 91, 92), and two used a video (80, 92). The mechanism of action was similar across all these technological based interventions. Patients were communicating using the phone or mobile app or tablet to ask for help if they have problems and concerns and health professionals acted accordingly. This meant patient were involved in taking care of themselves and making decisions.

The outcomes however varied across these studies. Self-efficacy was examined in two studies (36, 49), with different population (COPD (36) and T2D (49)) and they used different measures to assess self-efficacy, both studies reported significant improvement in self-efficacy. Quality of life was examined in five studies (46, 78, 80, 91, 92) and they all used different measures. Only one study reported significant benefits of the intervention (46). Hospitalisations/rehospitalisations, length of stay, unplanned visits were reported in four

For peer review only

For peer review only - http://bmjopen.bmj.com/site/about/guidelines.xhtml

studies (36, 47, 83, 91). All studies reported positive benefits of technology in reducing hospitalisations, length of stay and unplanned visits. Three of these studies were in COPD population (36, 47, 91), one study in the elderly population (83) and another in T2D population (92). Two studies reported data on knowledge of management of T2D (49, 59). One study recruited participants with T2D and hypertension (59). However only one study reported significant differences between the two groups on knowledge of T2D management (49).

Synthesis based on study design

Of the n=55 studies included studies, n=6 studies (n=9 papers) were non-RCT (23, 25, 26, 31, 52, 54, 55, 57, 58, 95). Participants in these studies were elderly people with multi-morbidity (23), total hip replacement (57, 58), cancer patients (31), chronic heart failure (52, 54, 55, 95), patients approaching death and their family caregivers (26), health professionals in nursing homes (25). Length of stay was assessed in heart failure, cancer, and hip replacement studies and was significant all studies (31, 57, 58, 95). Quality of life was assessed in three studies (26, 31, 54), and was significant in two studies (26, 31), among cancer patients (31) and family caregivers of patients approaching death (26).

For RCT design, n=12 studies did not clearly state the methods of randomisation. These were conducted in various populations: IBD (80), T2D (63, 79), breast reconstruction (39), stroke patients and their families (27-30), multi-morbidity patients and their families (75) heart failure/COPD (60, 61, 81), chronic pain/musculoskeletal pain/fibromyalgia (76, 82). Quality of life was assessed in seven studies and was significant in three studies (60, 61, 75), but remain unchanged in four studies (27, 76, 80, 81). Pain disability, intensity, and interference was assessed in the chronic pain study and showed positive benefits in all outcomes (76), while the MSP/Fibromyalgia assessed pain intensity and number of tender points. Only number of tender points significantly reduced (82). Health care utilisation was assessed in three studies (39, 79, 81). Emergency and elective admission rates significantly

For peer review only - http://bmjopen.bmj.com/site/about/guidelines.xhtml

For peer review only

decreased in T2D study (79), follow-up hospital visits significantly decreased in breast reconstruction study (39) while hospital admissions were not statistically significant in COPD population (81). Caregiver outcomes: burden, mood/anxiety (27), depression and strain (75) were not significant in both studies.

Thirty-six RCTs clearly stated randomisation methods and these recruited participants from patient, family caregivers and health care professionals. The main patient population were COPD (n=5) (34, 36, 47, 62, 65) T2D (n=6) (49, 56, 59, 78, 85, 92), multiple chronic conditions and /or elderly population n=6 (37, 38, 64, 83, 88, 93), arthritis n=4 (22, 40, 67, 74), cancer n=3 (46, 71, 73), acute coronary syndrome n=2 (32, 33, 41, 44, 45, 97), HIV n=2, and Parkinson's disease n=1 (69, 84, 90).

Quality of life, self-efficacy, health utilisation and costs of care were the main outcomes reported. Quality of life was assessed in n=13 studies, with six studies reporting statistically significant results. Quality of life was significant in a study among patients with chronic multiple conditions (37), COPD (47, 62, 65), and HIV (69, 84), but was not significant in T2D population (49, 78, 85), cancer (46), elderly with chronic conditions (38), acute coronary syndrome (32, 33) and patients at end of life (72).

Self-efficacy was assessed in seven studies (33, 34, 36, 37, 41, 49, 70) with only two reporting positive benefits of the intervention (41, 49). Health utilisation was reported in ten studies (36-38, 47, 65, 71, 83, 84, 86, 93). Rehospitalisations significantly improved in COPD population and chronic multiple conditions (36, 47, 83, 84, 93), mortality also reduced in COPD and chronic multiple conditions (36, 48, 65).

Health care use significantly reduced among the elderly with chronic conditions (38), length of hospital stay significantly reduced in COPD population (47), but was non-significant among older people (86). Hospital admission/visit to emergency was not significant in one COPD and cancer population (65, 71). Health care use was not significant in chronic multiple conditions (37).

For peer review only - http://bmjopen.bmj.com/site/about/guidelines.xhtml

Caregiver outcomes

Quality of life among caregivers and caregiver perceived burden significantly improved among family caregivers of older people in a geriatric practice (64). In a guided care intervention quality of chronic Illness care, work productivity loss and absenteeism improved significantly for caregivers (75). However depressive symptoms, and caregiver strain were not significantly changed (75). In a cluster randomized controlled trial of a client-centred, activities of daily living intervention for caregivers of people with stroke, caregiver burden, life satisfaction, perceived burden, mood, did not differ significantly (28).

Health professional outcomes

A training programme among oncologists resulted in significant changes in the following behavioural domains: transition to palliative care, general communication, and involving significant others (73). A patient-centred communication intervention reported that GP's knowledge about medication taken by the patient was not significant (38). Job strain did not differ significantly between groups even though the intervention reported greater job satisfaction. Similarly modified task and job analysis did not differ significantly, however time pressure did decrease significantly (89). Intention to engage in interprofessional shared decision making did not differ significantly in a Canadian trial (92).

Costs of care and utilisation of health services

A person-centred integrated intervention and a technology-based intervention for heart failure patients reduced the costs of care in the Swedish and Spanish trials, a nurse-led rheumatology clinic vs rheumatologists-led clinic, and in acute coronary syndrome (32, 52, 60, 67, 91), however costs of services were not different among elderly admitted to a unit with acute illness (86).

Hospital appointments decreased in the PC intervention compared to control in a multicenter cluster intervention for IBD patients (80) likewise in an interdisciplinary collaborative practice intervention hospital visits to see the physician reduced significantly (83). Patients in the

For peer review only - http://bmjopen.bmj.com/site/about/guidelines.xhtml

individualised care plan intervention called out the ambulance more frequent than those who received usual care (81), even though the intervention group had more GP visits compared with control group (15.6 vs 11.6) in 12 months and the intervention group had more hospital admissions compared with the control group the differences were not statistically significant (81), health utilization was not significantly different between a clinician-led self-management trial and usual care (37). A quasi-experimental design also showed no significant differences on health utilization, hospitalization, emergency department attendance (26).

In an integrated practice unit and modified virtual ward model in Singapore, unplanned readmissions at 30, 90 and 180 days were significantly lower in the intervention group than the control group (93), emergency department attendance were significantly lower at 30,90 and 180 days in the intervention (93). Likewise an interdisciplinary, collaborative practice intervention involving a primary care physician, a nurse, and a social worker for community-dwelling seniors with chronic illnesses, showed significant changes in number of hospital admissions per patient per year, percentage of patients with 1 or more hospital readmissions within 60 days, and mean number of visits to all physicians (83), fewer attendances at physical, occupational or speech therapy units (38) compared to control group. However, change in percentage of patients with 1 or more visits to the emergency department, change in proportion of patients with 1 or more home care visits, and change in number of patients with 1 or more nursing home placements and emergency visits were not significant (83). Similarly, in a centralized, nurse-delivered telephone-based service to improve care coordination and patient reported outcomes after surgery for colorectal cancer unplanned readmission changes in emergency visits were non-significant (71).

Mortality was significantly reduced in the community-based integrated care for frail COPD patients (65). Mortality was significantly lower in an integrated practice unit and modified virtual ward model (93). A comprehensive care programme with multidisciplinary input for patients with COPD reported reduction in mortality rates compared to usual care (47).

For peer review only - http://bmjopen.bmj.com/site/about/guidelines.xhtml

However, a team intervention for the multi-morbid elderly reported that mortality risk at 3- and 6-months follow-up were all nonsignificant (98).

A technology-based intervention of a home monitoring via mobile app on the number of in-person visits following ambulatory surgery showed that follow-up visits were significantly lower after surgery in the intervention compared to the control group (39), number of phone calls and emails made to the health care in 30 days after surgery were not significant (39). A person-centred communication intervention did not lead to change in number of medications taken by patient (38).

In a Norwegian patient-centred team intervention number of emergency admissions, sum of emergency inpatient bed days, count of emergency re-admissions within 30 days of discharge, count of planned out-patient visits, count of emergency outpatient visits, mortality risk at three- and six-months follow-up were all nonsignificant (98).

Clinical outcomes

Significant improvements were seen among T2D and hypertensive patients in systolic and diastolic blood pressure (59), likewise a patient-centred education programme among newly diagnosed patients with T2D, HbA1c was significant (63). Fasting blood sugar, HbA1c was not statistically different between the two groups (56, 59). In a self-management trial in Sweden among T2D patients HbA1c was significant (56), but not significant in a Thai trial (49), and computer-based USA trial (78). Furthermore, cholesterol levels were not different in a computer-based trial (78). Blood pressure (both systolic and diastolic) in a T2D trial was not significant (56, 85), haemoglobin was not significant (85). In a T2D UK trial body mass index was significant (85), but was not significant in a self-management trial for T2D patients (56). An Iranian trial to test the effect of a holistic care programme (HCP) on the reduction of iron overload in patients with beta-thalassaemia major change in serum ferritin at three months (mg/L), change in iron level at three months (micrograms/dL) were significant, but change in serum ferritin 1 year and 2 years post intervention, Total iron binding capacity at three months, haemoglobin (Hb) at three months was not significant (35).

For peer review only - http://bmjopen.bmj.com/site/about/guidelines.xhtml

Discussion

Person-centred care (PCC) is a crucial mechanism to achieve UHC and WHO policies. Our review found a need for data on operationalising PCC in the delivery of care for patients with serious illness. Furthermore, PCC can be provided across all settings (hospitals: in-patient, outpatient, primary care, community settings and residential homes), but majorly in primary care. Furthermore, PCC can be achieved by involving patients, their families and health professionals. PCC can also be provided using various approaches such as self-management interventions and technology-based interventions.

Most of the studies included in the review were conducted in high income countries predominantly in Sweden and USA, and most of the studies using technology were conducted in high income countries. Most participants in these studies had heart failure, T2D, COPD, cancer, and arthritis. The core component of the intervention included workshop training of health professionals on communication skills, training patients and families on self-assessment, identifying their problems and concerns, creating action plans based on the problems, identifying resources to self-management the problems, and evaluating the care. These components are in line with a systematic review of effective elements in a patient-centred and multi-morbidity care (101).
The main outcomes reported across most studies were quality of life, health utilisation, self- and self-efficacy.

Some studies found effectiveness of PCC interventions in improving quality of life, self-efficacy, health utilisation and reducing costs of care. However, some studies reported no significant differences between PCC interventions and usual care on those outcomes.

Most studies which used person-centred self-management approaches and technology demonstrated positive benefits of the interventions in reducing hospital admissions, length of stay and unplanned visits. This finding concurs with a review of self-management

For peer review only - http://bmjopen.bmj.com/site/about/guidelines.xhtml
For peer review only

interventions in respiratory and cardiovascular illness which reported that self-management support interventions reduces health service utilisation without compromising patient health outcomes (102). However self-efficacy outcomes were mostly significant in technology-based interventions, but not significant across most studies which utilised self-management approaches. Studies reported conflicting results on quality-of-life outcomes. Three of the six studies which used self-management approaches reported statistically significant results while only one of the six technology-based interventions reported statistically significant findings.

In terms of synthesis based on study design most non RCT reported significantly improved quality of life and reduced length of hospital stay. For RCT, of the twenty studies that reported data on quality-of-life outcomes, nine of them reported significant results, however some of these studies did not clearly state the method of randomisation. Our data is at odds with a previous review of palliative care interventions for patients with incurable illness which concluded that quality of life outcomes favoured palliative care interventions (103).
Most of the RCTs demonstrated positive effects on the interventions in reducing re/hospitalisation, and improving health utilisation, however self-efficacy was non-significant across most RCT's.
This is in line with many other reviews of effectiveness of person-centred interventions for non-serious illness (104). The conflicting results are probably because of variation in interpretation of what person-centred is. For instance a review by Constand et al (2014) identified several different patient-cantered care frameworks and models in their studies they included in their review (105).

Very few studies recruited health professionals (n=4) and caregivers (n=3) as study participants. Quality of life improved and perceived burden significantly reduced in two caregiver studies. Our findings concur with a review of caregiving intervention in cancer population (106, 107).

For peer review only

However psychosocial outcomes remained unchanged in our review. This is contrary to a review of multi-component and psycho-educational interventions designed to support caregivers in their role such as training, education and skill which found positive benefits in reducing depression and burden of caregiving (108). Our data is also at odds with findings among family caregivers in oncology populations which showed improved emotional support (106).

Studies among health professionals showed positive benefits on time pressure and communication skills, but no differences were reported on knowledge and job strain outcomes. No study reported data on implementation science outcomes among health professionals. The methodological quality of these studies was poor due small sample sizes, unclear randomisation methods and allocation concealment, therefore studies that reported data on caregivers and health professional outcomes are inconclusive.

Only two studies from this review demonstrated that person-centred interventions were effective in reducing pain outcomes, with five studies showing that interventions had no effect on pain and physical symptoms such as fatigue, shortness of breath in COPD and heart disease populations. However, a previous review on self-initiated interventions among cancer patients with peripheral neuropathy showed that strategies were beneficial in reducing symptoms and concerns (109).

Patient communication and satisfaction with PCC interventions was significant in three of the six studies that reported data on this outcome. Our findings agree with a systematic review on effectiveness of communication-related quality improvement interventions for patients with advanced and serious illness which reported significant improvements on patients' satisfaction with care (103, 110).

This review has shown that PCC interventions reduced costs of care in heart failure, COPD, acute coronary syndrome, and rheumatology populations. This is in line with a meta-analysis

For peer review only - http://bmjopen.bmj.com/site/about/guidelines.xhtml

on the economics of palliative care for adults with serious illness admitted to a facility that reported lower costs of palliative care consultations than usual care (111).

Meta-analysis was not possible in this review due to heterogenicity of studies. Studies were from different patient populations, different trial designs (parallel trials or clustered trials), different sample sizes, different interventions and dimensions, different outcomes and measures used, different follow-up periods and intervals, and interventions delivered in different settings. Some interventions targeted health care professionals and outcomes assessed among patients and health care professionals. Some interventions targeted patients and family dyads and captured data from both patients and their families, while some interventions targeted patients only, and family caregivers only.

Furthermore, interventions were delivered or led by different groups of professionals such as nurses, physiotherapists, physicians, social workers.

Due to nature of the interventions, it was difficult to blind study participants and those delivering the intervention, however three studies blinded study participants and two studies blinded those who delivered the intervention. It is challenging to design double-blinded or triple-blinded complex person-centred interventions. However, it is possible to blind outcome assessors. In this review only n=17 studies blinded outcomes assessors.

It is also interesting to note that the majority of the studies n=30 studies achieved relative complete follow-up, that is at least 80% of the participants were followed-up at trial end points. This is encouraging considering that is it challenging to follow-up participants with serious illnesses.

Conclusions, implications for policy, practice, and research

There is some evidence that PCC interventions using self-management have some effects in reducing health utilisation, costs of care, and improving quality of life. Technology based

For peer review only

interventions also reduces health utilisation and improves self-efficacy but have no effect on quality of life. However very few studies used self-management and technology approaches. Further work is needed to identify how self-management and technology PCC approaches can be used in serious illness across different disease conditions.

PCC can be designed and evaluated using robust study designs, and can be delivered in primary, secondary and tertiary care including home settings and residential homes. Institutions should therefore consider implementing person-centred care interventions using locally available resources.

PCC interventions can target patients, their families or health professionals. Person-centred care research has mainly focused in HIC, more research needs to be done in LMIC. Further work to consider designing and evaluating PCC interventions at community level targeting community health workers, and family members. Health service researchers should consider incorporating costs of person-centred care as an outcome when designing and evaluating complex interventions.

For peer review only

For peer review only - http://bmjopen.bmj.com/site/about/guidelines.xhtml

Contributors

KN planned, conducted searches, and submitted the manuscript. KN and AC extracted data. KN and AC assessed quality of the included studies and compared assessments. RH reviewed data extraction and quality appraisal. AG, RP, IP, LF, LG, and SV contributed to design and interpretation. All authors approved the manuscript.

Funding

This work is funded by the National Institute of Health Research (NIHR) Global Health Research Unit on Health System Strengthening in sub-Saharan Africa, King's College London (GHRU 16/136/54) using UK aid from the UK Government to support global health research.

For peer review only

Data availability statement

Data for this review are available in a public, open access repository. This review involved analysis of anonymised participant data available in published research studies. All studies included are referenced in the manuscript and are available online through research databases.

Ethics statement

Patient consent for publication: Not required.

Ethics approval

No ethical approval was sought for this work. This study is a systematic review that analysed anonymised data from published studies, which already obtained informed consent/ethical approval.

Conflict of interest

No conflict of interest to declare

References

1. WHO. Person-Centred Health Care: A policy framework Geneva: WHO; 2007 [
2. Gillian Le RM, Janine Bestall, Tim Ensor, Imogen Featherstone, Thomas Veale. The Impact of Universal Health Coverage, People Centred Care and Integrated Service Delivery on Key Health System Outcomes 2014 Leeds University of Leeds 2014 [
3. WHO. Universal Health Coverage: Moving Towards Better Health

Action Framework for the Western Pacific Region. WHO; 2016.
4. Kitson A, Marshall A, Bassett K, Zeitz K. What are the core elements of patient-centred care? A narrative review and synthesis of the literature from health policy, medicine and nursing. J Adv Nurs. 2013;69(1):4-15.
5. Wilberforce M, Challis D, Davies L, Kelly MP, Roberts C, Loynes N. Person-centredness in the care of older adults: a systematic review of questionnaire-based scales and their measurement properties. BMC Geriatrics. 2016;16(1):63.
6. Giusti A, Nkhoma K, Petrus R, Petersen I, Gwyther L, Farrant L, et al. The empirical evidence underpinning the concept and practice of person-centred care for serious illness: a systematic review. BMJ Glob Health. 2020;5(12).
7. Kelley AS, Covinsky KE, Gorges RJ, McKendrick K, Bollens-Lund E, Morrison RS, et al. Identifying Older Adults with Serious Illness: A Critical Step toward Improving the Value of Health Care. Health Serv Res. 2016.
8. WHO. People-centred care in low and middle income countries2010.
9. WHO. WHO global strategy on people-centred and integrated health services2015.
10. Sleeman KE, de Brito M, Etkind S, Nkhoma K, Guo P, Higginson IJ, et al. The escalating global burden of serious health-related suffering: projections to 2060 by world regions, age groups, and health conditions. Lancet Glob Health. 2019;7(7):e883-e92.
11. Harding R, Leam C. Clinical notes for informal carers in palliative care: recommendations from a random patient file audit. Palliative medicine. 2005;19(8):639-42.
12. Moens K, Higginson IJ, Harding R, Brearley S, Caraceni A, Cohen J, et al. Are There Differences in the Prevalence of Palliative Care-Related Problems in People Living With Advanced Cancer and Eight Non-Cancer Conditions? A Systematic Review. Journal of pain and symptom management. 2014;48(4):660-77.
13. Streid J, Harding R, Agupio G, Dinat N, Downing J, Gwyther L, et al. Stressors and resources of caregivers of patients with incurable progressive illness in sub-Saharan Africa. Qualitative health research. 2014;24(3):317-28.
14. Kelley AS. Defining "serious illness". J Palliat Med. 2014;17(9):985.
15. Little P, Everitt H, Williamson I, Warner G, Moore M, Gould C, et al. Observational study of effect of patient centredness and positive approach on outcomes of general practice consultations. Bmj. 2001;323(7318):908-11.
16. Nkhoma K, Giusti A, Petrus R, Petersen I, Gwyther L, Harding R. A systematic review investigating the effectiveness of person-centred interventions in serious physical illness. https://www.crd.york.ac.uk/prospero/#recordDetails. 2018.
17. Moher D, Liberati A, Tetzlaff J, Altman DG. Preferred Reporting Items for Systematic Reviews and Meta-Analyses: The PRISMA Statement. Journal of Clinical Epidemiology. 2009;62(10):1006-12.
18. Higgins J, Green S. Cochrane handbook for systematic reviews of interventions 4.2. 5 [updated May 2005]. The cochrane library. 2005(3).
19. Centre for Review and Dissemination. Systematic Reviews: CRD's Guidance for Undertaking Reviews in Health Care2009. Available from: http://www.york.ac.uk/inst/crd/pdf/Systematic_Reviews.pdf.
20. Randomised JBICACf, https://jbi.global/critical-appraisal-tools CTAa, docs/critical-appraisal-tools/JBI_Critical_Appraisal-Checklist_for_, 2020 RCTpAJ. Critical Appraisal Checklist for Randomised

For peer review only - http://bmjopen.bmj.com/site/about/guidelines.xhtml

Controlled Trials. 2016.
21. Popay J, Roberts, H. M., Sowden, A., Petticrew, M., Arai, L., Rodgers, M., & Britten, N. (2006). Guidance on the conduct of narrative synthesis in sytematic reviews. Institute for Health Research. https://www.google.com/url?sa=t&rct=j&q=&esrc=s&source=web&cd=1&cad=rja&uact=8&ved=2ahUKEwiK6fuyk9fgAhVDtnEKHV9-AUgQFjAAegQIBxAC&url=http%3A%2F%2Fciteseerx.ist.psu.edu%2Fviewdoc%2Fdownload%3Fdoi%3D10.1.1.178.3100%26rep%3Drep1%26type%3Dpdf&usg=AOvVaw3xMoGRunApJPo0_YYk1hqo. Guidance on the conduct of narrative synthesis in sytematic reviews. 2006.
22. Bergsten U, Almehed K, Baigi A, Jacobsson LTH. A randomized study comparing regular care with a nurse-led clinic based on tight disease activity control and person-centred care in patients with rheumatoid arthritis with moderate/high disease activity: A 6-month evaluation. Musculoskeletal Care. 2019;17(3):215-25.
23. Berntsen GKR, Dalbakk M, Hurley JS, Bergmo T, Solbakken B, Spansvoll L, et al. Person-centred, integrated and pro-active care for multi-morbid elderly with advanced care needs: a propensity score-matched controlled trial. BMC health services research. 2019;19(1):682-.
24. Berendonk C, Kaspar R, Bär M, Hoben M. Improving Quality of Work life for Care Providers by Fostering the Emotional well-being of Persons with Dementia: A Cluster-randomized Trial of a Nursing Intervention in German long-term Care Settings. Dementia (London). 2019;18(4):1286-309.
25. Bökberg C, Behm L, Wallerstedt B, Ahlström G. Evaluation of person-centeredness in nursing homes after a palliative care intervention: pre- and post-test experimental design. BMC Palliative Care. 2019;18(1):44.
26. Britt HR, JaKa, M. M., Fernstrom, K. M., Bingham, P. E., Betzner, A. E., Taghon, J. R., Shippee, N. D., Shippee, T. P., Schellinger, S. E., & Anderson, E. W. . Quasi-Experimental Evaluation of LifeCourse on Utilization and Patient and Caregiver Quality of Life and Experience. . The American journal of hospice & palliative care 2019;36(5):408-16.
27. Hedman A, Eriksson G, von Koch L, Guidetti S. Five-year follow-up of a cluster-randomized controlled trial of a client-centred activities of daily living intervention for people with stroke. Clin Rehabil. 2019;33(2):262-76.
28. Bertilsson AS, Eriksson G, Ekstam L, Tham K, Andersson M, von Koch L, et al. A cluster randomized controlled trial of a client-centred, activities of daily living intervention for people with stroke: one year follow-up of caregivers. Clinical rehabilitation. 2016;30(8):765-75.
29. Guidetti S, Ranner M, Tham K, Andersson M, Ytterberg C, von Koch L. A "Client-Centred Activities of Daily Living" Intervention for Persons with Stroke: One-Year Follow-up of a Randomized Controlled Trial. Journal of Rehabilitation Medicine. 2015;47(7):605-11.
30. Bertilsson A-S, Ranner M, von Koch L, Eriksson G, Johansson U, Ytterberg C, et al. A client-centred ADL intervention: three-month follow-up of a randomized controlled trial. Scandinavian journal of occupational therapy. 2014;21(5):377-91.
31. Öhlén J, Sawatzky R, Pettersson M, Sarenmalm EK, Larsdotter C, Smith F, et al. Preparedness for colorectal cancer surgery and recovery through a person-centred information and communication intervention - A quasi-experimental longitudinal design. PLoS One. 2019;14(12):e0225816.
32. Pirhonen L, Bolin K, Olofsson EH, Fors A, Ekman I, Swedberg K, et al. Person-Centred Care in Patients with Acute Coronary Syndrome: Cost-Effectiveness Analysis Alongside a Randomised Controlled Trial. PharmacoEconomics - Open. 2019;3(4):495-504.
33. Pirhonen L, Olofsson EH, Fors A, Ekman I, Bolin K. Effects of person-centred care on health outcomes-A randomized controlled trial in patients with acute coronary syndrome. Health Policy. 2017;121(2):169-79.
34. Zakrisson AB, Arne M, Hasselgren M, Lisspers K, Ställberg B, Theander K. A complex intervention of self-management for patients with COPD or CHF in primary care improved performance and satisfaction with regard to own selected activities; A longitudinal follow-up. J Adv Nurs. 2019;75(1):175-86.

35. Arian M, Memarian R, Oghazian MB, Vakilian F, Badiee Z. The effect of a holistic care program on the reduction of iron over load in patients with beta-thalassemia major: A randomized clinical trial. Iranian Red Crescent Medical Journal. 2018;20 (4) (no pagination)(e60820).
36. Fors A, Blanck E, Ali L, Swedberg K, Ekman I. Person-centred telephone-support is effective in patients with chronic obstructive pulmonary disease and/or chronic heart failure-six-month follow-up of a randomized controlled trial. European Journal of Heart Failure. 2018;20 (Supplement 1):194.
37. Reed RL, Roeger L, Howard S, Oliver-Baxter JM, Battersby MW, Bond M, et al. A self-management support program for older Australians with multiple chronic conditions: A randomised controlled trial. Medical Journal of Australia. 2018;208(2):69-74.
38. Schafer I, Kaduszkiewicz H, Mellert C, Loffler C, Mortsiefer A, Ernst A, et al. Narrative medicine-based intervention in primary care to reduce polypharmacy: results from the cluster-randomised controlled trial MultiCare AGENDA. BMJ Open. 2018;8(1):e017653.
39. Armstrong KA, Coyte PC, Brown M, Beber B, Semple JL. Effect of home monitoring via mobile app on the number of in-person visits following ambulatory surgery a randomized clinical trial. JAMA Surgery. 2017;152(7):622-7.
40. Feldthusen C, Dean E, Forsblad-d'Elia H, Mannerkorpi K. Effects of Person-Centered Physical Therapy on Fatigue-Related Variables in Persons With Rheumatoid Arthritis: A Randomized Controlled Trial. Arch Phys Med Rehabil. 2016;97(1):26-36.
41. Fors A, Swedberg K, Ulin K, Wolf A, Ekman I. Effects of person-centred care after an event of acute coronary syndrome: Two-year follow-up of a randomised controlled trial. International Journal of Cardiology. 2017;249:42-7.
42. Fors A, Taft C, Ulin K, Ekman I. Person-centred care improves self-efficacy to control symptoms after acute coronary syndrome: A randomized controlled trial. European Journal of Cardiovascular Nursing. 2016;15(2):186-94.
43. Fors A, Gyllensten H, Swedberg K, Ekman I. Effectiveness of person-centred care after acute coronary syndrome in relation to educational level: Subgroup analysis of a two-armed randomised controlled trial. Int J Cardiol. 2016;221:957-62.
44. Fors A, Ekman I, Taft C, Björkelund C, Frid K, Larsson ME, et al. Person-centred care after acute coronary syndrome, from hospital to primary care - A randomised controlled trial. Int J Cardiol. 2015;187:693-9.
45. Wolf A, Fors A, Ulin K, Thorn J, Swedberg K, Ekman I. An eHealth Diary and Symptom-Tracking Tool Combined With Person-Centered Care for Improving Self-Efficacy After a Diagnosis of Acute Coronary Syndrome: A Substudy of a Randomized Controlled Trial. J Med Internet Res. 2016;18(2):e40.
46. Hansson E, Carlström E, Olsson LE, Nyman J, Koinberg I. Can a person-centred-care intervention improve health-related quality of life in patients with head and neck cancer? A randomized, controlled study. BMC Nurs. 2017;16:9.
47. Ko FWS, Cheung NK, Rainer TH, Lum C, Wong I, Hui DSC. Comprehensive care programme for patients with chronic obstructive pulmonary disease: A randomised controlled trial. Thorax. 2017;72(2):122-8.
48. Low LL, Tan SY, Ng MJM, Tay WY, Ng LB, Balasubramaniam K, et al. Applying the Integrated Practice Unit Concept to a Modified Virtual Ward Model of Care for Patients at Highest Risk of Readmission: A Randomized Controlled Trial. PloS one. 2017;12(1):e0168757-e.
49. Wichit N, Mnatzaganian G, Courtney M, Schulz P, Johnson M. Randomized controlled trial of a family-oriented self-management program to improve self-efficacy, glycemic control and quality of life among Thai individuals with Type 2 diabetes. Diabetes Research and Clinical Practice. 2017;123:37-48.
50. Larsson A, Palstam A, Löfgren M, Ernberg M, Bjersing J, Bileviciute-Ljungar I, et al. Resistance exercise improves muscle strength, health status and pain intensity in fibromyalgia--a randomized controlled trial. Arthritis Res Ther. 2015;17(1):161.

For peer review only - http://bmjopen.bmj.com/site/about/guidelines.xhtml

51. Ericsson A, Palstam A, Larsson A, Löfgren M, Bileviciute-Ljungar I, Bjersing J, et al. Resistance exercise improves physical fatigue in women with fibromyalgia: a randomized controlled trial. Arthritis Res Ther. 2016;18:176.
52. Hansson E, Ekman I, Swedberg K, Wolf A, Dudas K, Ehlers L, et al. Person-centred care for patients with chronic heart failure - a cost-utility analysis. Eur J Cardiovasc Nurs. 2016;15(4):276-84.
53. Ulin K, Olsson LE, Wolf A, Ekman I. Person-centred care - An approach that improves the discharge process. European Journal of Cardiovascular Nursing. 2016;15(3):e19-26.
54. Ekman I, Wolf A, Olsson LE, Taft C, Dudas K, Schaufelberger M, et al. Effects of person-centred care in patients with chronic heart failure: the PCC-HF study. European Heart Journal. 2012;33(9):1112-9.
55. Dudas K, Olsson LE, Wolf A, Swedberg K, Taft C, Schaufelberger M, et al. Uncertainty in illness among patients with chronic heart failure is less in person-centred care than in usual care. European Journal of Cardiovascular Nursing. 2013;12(6):521-8.
56. Jutterstrom L, Hornsten A, Sandstrom H, Stenlund H, Isaksson U. Nurse-led patient-centered self-management support improves HbA1c in patients with type 2 diabetes-A randomized study. Patient Education and Counseling. 2016;99(11):1821-9.
57. Olsson LE, Hansson E, Ekman I. Evaluation of person-centred care after hip replacement-a controlled before and after study on the effects of fear of movement and self-efficacy compared to standard care. BMC Nurs. 2016;15(1):53.
58. Olsson L-E, Karlsson J, Berg U, Kärrholm J, Hansson E. Person-centred care compared with standardized care for patients undergoing total hip arthroplasty—a quasi-experimental study. Journal of Orthopaedic Surgery and Research. 2014;9(1):95.
59. Or C, Tao D. A 3-Month Randomized Controlled Pilot Trial of a Patient-Centered, Computer-Based Self-Monitoring System for the Care of Type 2 Diabetes Mellitus and Hypertension. Journal of Medical Systems. 2016;40(4):81.
60. Sahlen K-G, Boman K, Brannstrom M. A cost-effectiveness study of person-centered integrated heart failure and palliative home care: Based on a randomized controlled trial. Palliative Medicine. 2016;30(3):296-302.
61. Brannstrom M, Boman K. Effects of person-centred and integrated chronic heart failure and palliative home care. PREFER: a randomized controlled study. European Journal of Heart Failure. 2014;16(10):1142-51.
62. Slok AH, Kotz D, van Breukelen G, Chavannes NH, Rutten-van Molken MP, Kerstjens HA, et al. Effectiveness of the Assessment of Burden of COPD (ABC) tool on health-related quality of life in patients with COPD: a cluster randomised controlled trial in primary and hospital care. BMJ Open. 2016;6(7):e011519.
63. Windrum P, Garcia-Goni M, Coad H. The Impact of Patient-Centered versus Didactic Education Programs in Chronic Patients by Severity: The Case of Type 2 Diabetes Mellitus. Value in Health. 2016;19(4):353-62.
64. Yu DSF. Effects of a Health and Social Collaborative Case Management Model on Health Outcomes of Family Caregivers of Frail Older Adults: Preliminary Data from a Pilot Randomized Controlled Trial. Journal of the American Geriatrics Society. 2016;64(10):2144-8.
65. Hernandez C, Alonso A, Garcia-Aymerich J, Serra I, Marti D, Rodriguez-Roisin R, et al. Effectiveness of community-based integrated care in frail COPD patients: A randomised controlled trial. npj Primary Care Respiratory Medicine. 2015;25 (no pagination)(15022).
66. Kikkenborg Berg S, Stoier L, Moons P, Zwisler AD, Winkel P, Ulrich Pedersen P. Emotions and health: findings from a randomized clinical trial on psychoeducational nursing to patients with implantable cardioverter defibrillator. The Journal of cardiovascular nursing. 2015;30(3):197-204.
67. Larsson I, Fridlund B, Arvidsson B, Teleman A, Svedberg P, Bergman S. A nurse-led rheumatology clinic versus rheumatologist-led clinic in monitoring of patients with chronic inflammatory arthritis undergoing biological therapy: A cost comparison study in a randomised controlled trial. BMC Musculoskeletal Disorders. 2015;16 (1) (no pagination)(817).

For peer review only - http://bmjopen.bmj.com/site/about/guidelines.xhtml

68. Larsson I, Fridlund B, Arvidsson B, Teleman A, Bergman S. Treatment outcomes from a nurse-led rheumatology clinic in monitoring of anti-TNF therapy-a randomised controlled trial. Arthritis and Rheumatism. 2012;10):S667.
69. Lowther K, Selman L, Simms V, Gikaara N, Ahmed A, Ali Z, et al. Nurse-led palliative care for HIV-positive patients taking antiretroviral therapy in Kenya: a randomised controlled trial. Lancet HIV. 2015;2(8):e328-34.
70. Kelechi TJ, Mueller M, Spencer C, Rinard B, Loftis G. The effect of a nurse-directed intervention to reduce pain and improve behavioral and physical outcomes in patients with critically colonized/infected chronic leg ulcers. Journal of Wound, Ostomy, & Continence Nursing. 2014;41(2):111-21.
71. Young JM, Butow PN, Walsh J, Durcinoska I, Dobbins TA, Rodwell L, et al. Multicenter randomized trial of centralized nurse-led telephone-based care coordination to improve outcomes after surgical resection for colorectal cancer: the CONNECT intervention. Journal of Clinical Oncology. 2013;31(28):3585-91.
72. Chochinov HM, Kristjanson LJ, Breitbart W, McClement S, Hack TF, Hassard T, et al. Effect of dignity therapy on distress and end-of-life experience in terminally ill patients: a randomised controlled trial. Lancet Oncology. 2011;12(8):753-62.
73. Goelz T, Wuensch A, Stubenrauch S, Ihorst G, de Figueiredo M, Bertz H, et al. Specific training program improves oncologists' palliative care communication skills in a randomized controlled trial. Journal of Clinical Oncology. 2011;29(25):3402-7.
74. Murphy SL, Lyden AK, Smith DM, Dong Q, Koliba JF. Effects of a tailored activity pacing intervention on pain and fatigue for adults with osteoarthritis. American Journal of Occupational Therapy. 2010;64(6):869-76.
75. Wolff JL, Giovannetti ER, Boyd CM, Reider L, Palmer S, Scharfstein D, et al. Effects of guided care on family caregivers. The Gerontologist. 2010;50(4):459-70.
76. Dobscha SK, Corson K, Perrin NA, Hanson GC, Leibowitz RQ, Doak MN, et al. Collaborative care for chronic pain in primary care: a cluster randomized trial. JAMA. 2009;301(12):1242-52.
77. Machado LA, Azevedo DC, Capanema MB, Neto TN, Cerceau DM. Client-Centered Therapy vs Exercise Therapy for Chronic Low Back Pain: A Pilot Randomized Controlled Trial in Brazil. Pain Medicine. 2007;8(3):251-8.
78. Glasgow RE, Nutting PA, King DK, Nelson CC, Cutter G, Gaglio B, et al. Randomized effectiveness trial of a computer-assisted intervention to improve diabetes care. Diabetes Care. 2005;28(1):33-9.
79. Mills PD, Harvey PW. Beyond community-based diabetes management and the COAG coordinated care trial. Australian Journal of Rural Health. 2003;11(3):131-7.
80. Kennedy A, Nelson E, Reeves D, Richardson G, Roberts C, Robinson A, et al. A randomised controlled trial to assess the impact of a package comprising a patient-orientated, evidence-based self-help guidebook and patient-centred consultations on disease management and satisfaction in inflammatory bowel disease. Health Technology Assessment. 2003;7(28).
81. Martin IR, McNamara D, Sutherland FR, Tilyard MW, Taylor DR. Care plans for acutely deteriorating COPD: a randomized controlled trial. Chronic respiratory disease. 2004;1(4):191-5.
82. Alamo MM, Moral RR, Perula de Torres LA. Evaluation of a patient-centred approach in generalized musculoskeletal chronic pain/fibromyalgia patients in primary care. Patient Education & Counseling. 2002;48(1):23-31.
83. Sommers LS, Marton KI, Barbaccia JC, Randolph J. Physician, nurse, and social worker collaboration in primary care for chronically ill seniors. Archives of Internal Medicine. 2000;160(12):1825-33.
84. Gustafson D, Hawkins R, Boberg E, Bricker E, Pingree S, Chan C-L. The use and impact of a computer-based support system for people living with AIDS and HIV infection

For peer review only

. Annual Symposium on Computer Application [sic] in Medical Care Symposium on Computer Applications in Medical Care. 1994;1(2):604-8.
85. Kinmonth AL, Woodcock A, Griffin S, Spiegal N, Campbell MJ. Randomised controlled trial of patient centred care of diabetes in general practice: Impact on current wellbeing and future disease risk. British Medical Journal. 1998;317(7167):1202-8.
86. Landefeld CS, Palmer RM, Kresevic DM, Fortinsky RH, Kowal J. A randomized trial of care in a hospital medical unit especially designed to improve the functional outcomes of acutely ill older patients. New England Journal of Medicine. 1995;332(20):1338-44.
87. Larsson I, Bergman S, Bremander A. Person-centred care (PCC) may improve health care consumer skills more than regular care-an RCT in patients with cia undergoing biological therapy. Annals of the Rheumatic Diseases. 2015;2):104.
88. Mielenz TJ, Tracy M, Jia H, Durbin LL, Allegrante JP, Arniella G, et al. Creation of the Person-Centered Wellness Home in Older Adults. Innovation in Aging. 2020;4(1).
89. Berendonk C, Kaspar R, Bär M, Hoben M. Improving Quality of Work life for Care Providers by Fostering the Emotional well-being of Persons with Dementia: A Cluster-randomized Trial of a Nursing Intervention in German long-term Care Settings Dementia 2019;18(4):1286-309.
90. Eggers C, Dano R, Schill J, Fink GR, Hellmich M, Timmermann L. Patient-centered integrated healthcare improves quality of life in Parkinson's disease patients: a randomized controlled trial. J Neurol. 2018;265(4):764-73.
91. de Batlle J, Massip M, Vargiu E, Nadal N, Fuentes A, Ortega Bravo M, et al. Implementing Mobile Health-Enabled Integrated Care for Complex Chronic Patients: Intervention Effectiveness and Cost-Effectiveness Study. JMIR Mhealth Uhealth. 2021;9(1):e22135.
92. Yu C, Choi D, Bruno BA, Thorpe KE, Straus SE, Cantarutti P, et al. Impact of MyDiabetesPlan, a Web-Based Patient Decision Aid on Decisional Conflict, Diabetes Distress, Quality of Life, and Chronic Illness Care in Patients With Diabetes: Cluster Randomized Controlled Trial. J Med Internet Res. 2020;22(9):e16984.
93. Low LL, Tan SY, Ng MJM, Tay WY, Ng LB, Balasubramaniam K, et al. Applying the integrated practice unit concept to a modified virtual ward model of care for patients at highest risk of readmission: A randomized controlled trial. PLoS ONE. 2017;12 (1) (no pagination)(e0168757).
94. Larsson I, Fridlund B, Arvidsson B, Teleman A, Bergman S. Biological therapy can be monitored more cost effectively by a nurse-led rheumatology clinic. Annals of the Rheumatic Diseases Conference: Annual European Congress of Rheumatology of the European League Against Rheumatism, EULAR. 2014;73(SUPPL. 2).
95. Ulin K, Olsson LE, Wolf A, Ekman I. Person-centred care - An approach that improves the discharge process. European journal of cardiovascular nursing : journal of the Working Group on Cardiovascular Nursing of the European Society of Cardiology. 2016;15(3):e19-e26.
96. Dambha-Miller H, Cooper AJM, Simmons RK, Kinmonth AL, Griffin SJ. Patient-centred care, health behaviours and cardiovascular risk factor levels in people with recently diagnosed type 2 diabetes: 5-year follow-up of the ADDITION-Plus trial cohort. BMJ Open. 2016;6 (1) (no pagination)(e008931).
97. Fors A, Ekman I, Ulin K, Wolf A, Swedberg K. Person-centred care is effective after an event of acute coronary syndrome; particularly in patients with low educational level-two-year follow-up of a randomised controlled trial. European Heart Journal. 2017;38 (Supplement 1):116.
98. Berntsen G, Hoyem A, Lettrem I, Ruland C, Rumpsfeld M, Gammon D. A person-centered integrated care quality framework, based on a qualitative study of patients' evaluation of care in light of chronic care ideals. BMC Health Serv Res. 2018;18(1):479.
99. Bergmo TS, Berntsen GK, Dalbakk M, Rumpsfeld M. The effectiveness and cost effectiveness of the PAtient-Centred Team (PACT) model: study protocol of a prospective matched control before-and-after study. BMC Geriatrics. 2015;15:133.
100. Popay J, Roberts, H. M., Sowden, A., Petticrew, M., Arai, L., Rodgers, M., & Britten, N. (2006). Guidance on the conduct of narrative synthesis in sytematic reviews. Institute for Health Research.

For peer review only - http://bmjopen.bmj.com/site/about/guidelines.xhtml

https://www.google.com/url?sa=t&rct=j&q=&esrc=s&source=web&cd=1&cad=rja&uact=8&ved=2ahUKEwiK6fuyk9fgAhVDtnEKHV9-AUgQFjAAegQIBxAC&url=http%3A%2F%2Fciteseerx.ist.psu.edu%2Fviewdoc%2Fdownload%3Fdoi%3D10.1.1.178.3100%26rep%3Drep1%26type%3Dpdf&usg=AOvVaw3xMoGRunApJPo0_YYk1hqo.
101. Poitras M-E, Maltais M-E, Bestard-Denommé L, Stewart M, Fortin M. What are the effective elements in patient-centered and multimorbidity care? A scoping review. BMC Health Services Research. 2018;18(1):446.
102. Panagioti M, Richardson G, Small N, Murray E, Rogers A, Kennedy A, et al. Self-management support interventions to reduce health care utilisation without compromising outcomes: a systematic review and meta-analysis. BMC Health Services Research. 2014;14(1):356.
103. El-Jawahri AR, Greer JA, Temel JS. Does palliative care improve outcomes for patients with incurable illness? A review of the evidence. The journal of supportive oncology. 2011;9(3):87-94.
104. Ratti V, Hassiotis A, Crabtree J, Deb S, Gallagher P, Unwin G. The effectiveness of person-centred planning for people with intellectual disabilities: A systematic review. Research in Developmental Disabilities. 2016;57:63-84.
105. Constand MK, MacDermid JC, Dal Bello-Haas V, Law M. Scoping review of patient-centered care approaches in healthcare. BMC Health Services Research. 2014;14(1):271.
106. Ferrell B, Wittenberg E. A review of family caregiving intervention trials in oncology. . CA Cancer J Clin. 2017;67(4): 318-25.
107. Northouse LL, Katapodi MC, Song L, Zhang, L., , Mood DWC. Interventions with family caregivers of cancer patients: meta-analysis of randomized trials. a cancer journal for clinicians. 2010;60(5):317-39.
108. Parker D, Mills S, J. A. Effectiveness of interventions that assist caregivers to support people with dementia living in the community: a systematic review. Int J Evid Based Healthc. 2008;6(2).
109. Ogle T, Alexander K, Miaskowski C, Yates P. Systematic review of the effectiveness of self-initiated interventions to decrease pain and sensory disturbances associated with peripheral neuropathy. Journal of Cancer Survivorship. 2020;14(4):444-63.
110. Fawole OA, Dy SM, Wilson RF, Lau BD, Martinez KA, Apostol CC, et al. A systematic review of communication quality improvement interventions for patients with advanced and serious illness. J Gen Intern Med. 2013;28(4):570-7.
111. May P, Normand C, Cassel JB, Del Fabbro E, Fine RL, Menz R, et al. Economics of Palliative Care for Hospitalized Adults With Serious Illness: A Meta-analysis. JAMA Intern Med. 2018;178(6):820-9.

For peer review only

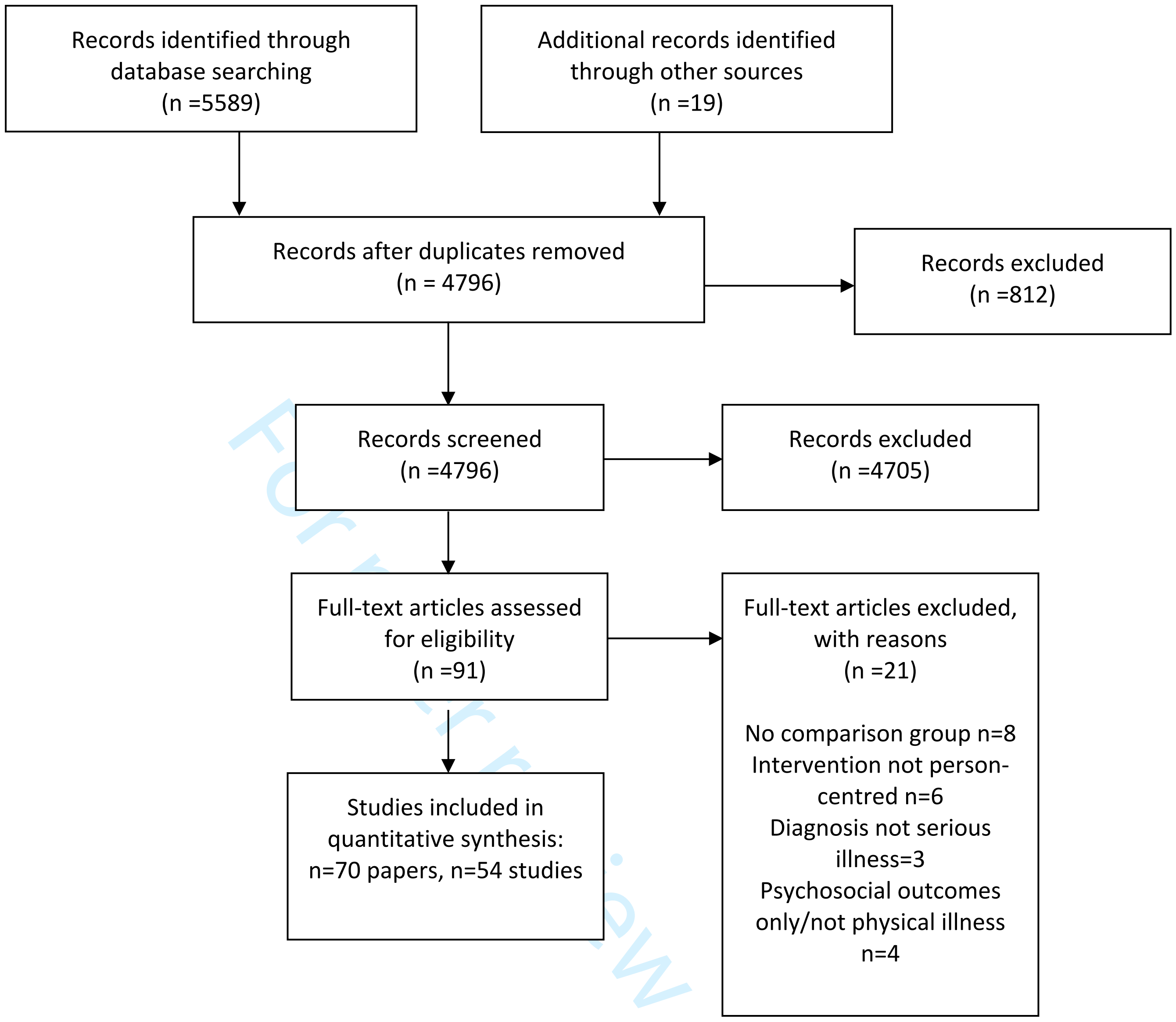

Figure 1: PRISMA Flow Diagram

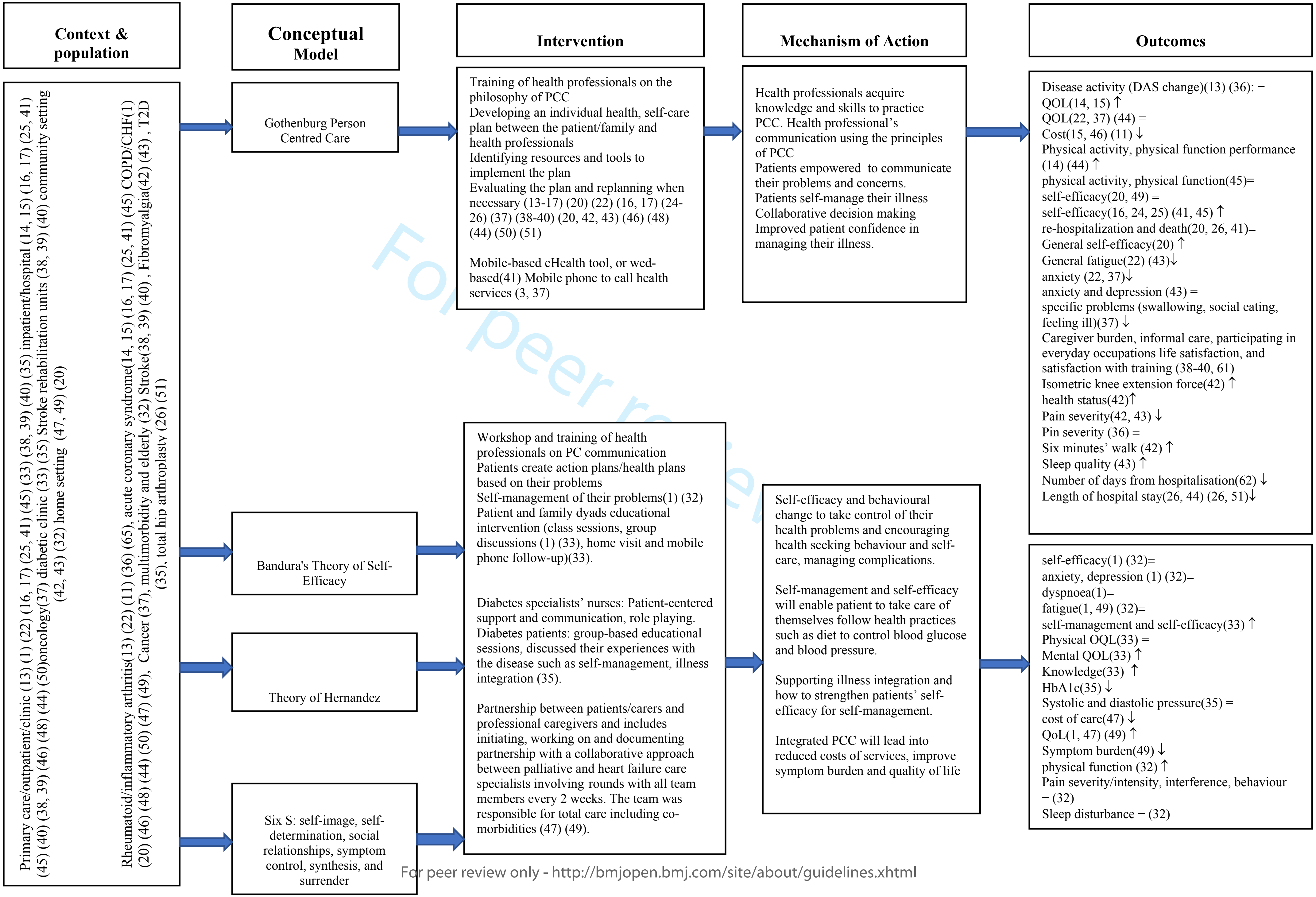

For peer review only - http://bmjopen.bmj.com/site/about/guidelines.xhtml

Figure 2: Logic Model for interventions with a theoretical model

Context & population

Primary care (2) (3) (5) (7) (8) (10, 11) (18) (21) (21) (19) (27) (28), hospital (4) (29) (30) (27) geriatric/care home(2) (31) (34), long-term care facilities (52), home sett(56) (59) (6) (4) (12) (19, 55)55] (19), community (29) wound centers (53) surgery (60) (59) (12), Oncology(54)

multimorbid elderly/frail (2) (34) (55) (19) , dementia/Parkinson's disease (52) (63), nursing home staff(31) , multiple conditions (cardiovascular, cancer, dementia, comorbidity)(56) (67) (3) (4) , cancer (60) (59) (12) (29) (54), Beta-thalassaemia major(64) , COPD/CHF (23) (57) (6) (68) (18) (27), T2D(33) (28) (66) (58) (10) (9), infected leg ulcer (53) , HIV (5) (70) , muscular skeletal/chronic pain (7) (8), inflammatory bowel disease (30)

Conceptual Model

None Stated

Intervention description

Multidisciplinary teamwork (2) (31) (23) (27)
Training, seminars and implementation of the training (31, 52) (12) (34) (6) (53) (29) (54) (55) (7) (30) (21) (28)

Formulation of goals(2, 56) (3) (57) (58) (7) (28)
patient-centred communication (21) (60) (54)

Patient, family, community involvement in intervention implementation (52) (56) (64) (34) (6) (65) (55)

Group-based training of patients (64)
Face-to-face discussion with GP, nurse (3) (6)

Education materials: Booklet/brochure(31, 60) (58) (53) (65)
Individualised care plans (4) (55) (10) (9) (18) (21) (19) (28) (63)
Self-management materials and/or approaches (66) (6) (55) (10) (30) (27)

Technology-based (23) (59) (12) (4) (54) (66) (10) (30) (19) (27) (28)remote consultation follow-up using mobile app/phone(23) (59) (12) (4) (30) (19) (63)

Mechanism of action

Enables proper assessment and prompt management of patient's problems thereby reducing unnecessary hospitalisation and saves costs.

Educational intervention will improve knowledge and care will be provided in a PC environment.
Improving communication skills during consultation
Self-management promotes self-efficacy and improves quality of life.

Outcomes

Emergency care/visit(2) (4) (6) (6) (9)↓
Emergency visit (12) (19)=
Mortality/mortality risk(2) (23) (4) (6) ↓
Job satisfaction(31, 52) ↑
Patient satisfaction (30) =
Patient enablement/decision making (28) (30) ↑
Time pressure(52) ↓
PCC assessment and environment(31) =
Patient/caregiver depression (56) (6) (7)↓
Patient/caregiver anxiety (56) (21)↓
patient/caregiver anxiety (6) (29) (30)=
caregiver strain (55) =
patient/caregiver depression (29, 31) (55) (8) (10) (30) (21) (63)=
Length of stay (60) (3) (23)↓
QOL (60) (23) (57) (34) (6) (5) (55) (63)↑
QOL: general health, emotional, physical, cognitive functions (60) (3) (7) (10) (30) (18) (27) (28) (63)=
Motor and non-motor functioning (63)
Patient satisfaction (3) (59) =
Number of physical contacts/follow-up visits (27) (59) ↓
Post-operative complications (59) =
Hospital admissions/unplanned (6) (12) =
Readmission rate/hospital appointments (23) (4) (69) (30) (19)↓
Dyspnoea (23) ↓
Dyspnoea (6) =
Distress (12) (28)=
6MWT (23) =
Systolic and diastolic blood pressure ↓
HbA1c ↓
COPD knowledge and self-management (6) ↑
Pain disability/intensity/severity (53) (7)↓
Pain disability (8) =
Pain severity (5) (65) (8) (21)=
Number of pain sites (21) =
Pain interference (7) ↓
Fatigue (65) =
Communication skills (54) ↑
Self-efficacy/confidence (53)=
Cost of care (27) ↓

Figure 3: Logic model for Interventions without a theoretic model

For peer review only

1. Zakrisson AB, Arne M, Hasselgren M, Lisspers K, Ställberg B, Theander K. A complex intervention of self-management for patients with COPD or CHF in primary care improved performance and satisfaction with regard to own selected activities; A longitudinal follow-up. J Adv Nurs. 2019;75(1):175-86.
2. Berntsen GKR, Dalbakk M, Hurley JS, Bergmo T, Solbakken B, Spansvoll L, et al. Person-centred, integrated and pro-active care for multi-morbid elderly with advanced care needs: a propensity score-matched controlled trial. BMC health services research. 2019;19(1):682-.
3. Schafer I, Kaduszkiewicz H, Mellert C, Loffler C, Mortsiefer A, Ernst A, et al. Narrative medicine-based intervention in primary care to reduce polypharmacy: results from the cluster-randomised controlled trial MultiCare AGENDA. BMJ Open. 2018;8(1):e017653.
4. Low LL, Tan SY, Ng MJM, Tay WY, Ng LB, Balasubramaniam K, et al. Applying the integrated practice unit concept to a modified virtual ward model of care for patients at highest risk of readmission: A randomized controlled trial. PLoS ONE. 2017;12 (1) (no pagination)(e0168757).
5. Lowther K, Selman L, Simms V, Gikaara N, Ahmed A, Ali Z, et al. Nurse-led palliative care for HIV-positive patients taking antiretroviral therapy in Kenya: a randomised controlled trial. Lancet HIV. 2015;2(8):e328-34.
6. Hernandez C, Alonso A, Garcia-Aymerich J, Serra I, Marti D, Rodriguez-Roisin R, et al. Effectiveness of community-based integrated care in frail COPD patients: A randomised controlled trial. npj Primary Care Respiratory Medicine. 2015;25 (no pagination)(15022).
7. Dobscha SK, Corson K, Perrin NA, Hanson GC, Leibowitz RQ, Doak MN, et al. Collaborative care for chronic pain in primary care: a cluster randomized trial. JAMA. 2009;301(12):1242-52.
8. Machado LA, Azevedo DC, Capanema MB, Neto TN, Cerceau DM. Client-Centered Therapy vs Exercise Therapy for Chronic Low Back Pain: A Pilot Randomized Controlled Trial in Brazil. Pain Medicine. 2007;8(3):251-8.
9. Mills PD, Harvey PW. Beyond community-based diabetes management and the COAG coordinated care trial. Australian Journal of Rural Health. 2003;11(3):131-7.
10. Glasgow RE, Nutting PA, King DK, Nelson CC, Cutter G, Gaglio B, et al. Randomized effectiveness trial of a computer-assisted intervention to improve diabetes care. Diabetes Care. 2005;28(1):33-9.
11. Larsson I, Fridlund B, Arvidsson B, Teleman A, Svedberg P, Bergman S. A nurse-led rheumatology clinic versus rheumatologist-led clinic in monitoring of patients with chronic inflammatory arthritis undergoing biological therapy: A cost comparison study in a randomised controlled trial. BMC Musculoskeletal Disorders. 2015;16 (1) (no pagination)(817).
12. Young JM, Butow PN, Walsh J, Durcinoska I, Dobbins TA, Rodwell L, et al. Multicenter randomized trial of centralized nurse-led telephone-based care coordination to improve outcomes after surgical resection for colorectal cancer: the CONNECT intervention. Journal of Clinical Oncology. 2013;31(28):3585-91.
13. Bergsten U, Almehed K, Baigi A, Jacobsson LTH. A randomized study comparing regular care with a nurse-led clinic based on tight disease activity control and person-centred care in patients with rheumatoid arthritis with moderate/high disease activity: A 6-month evaluation. Musculoskeletal Care. 2019;17(3):215-25.

For peer review only - http://bmjopen.bmj.com/site/about/guidelines.xhtml

14. Pirhonen L, Olofsson EH, Fors A, Ekman I, Bolin K. Effects of person-centred care on health outcomes-A randomized controlled trial in patients with acute coronary syndrome. Health Policy. 2017;121(2):169-79.
15. Pirhonen L, Bolin K, Olofsson EH, Fors A, Ekman I, Swedberg K, et al. Person-Centred Care in Patients with Acute Coronary Syndrome: Cost-Effectiveness Analysis Alongside a Randomised Controlled Trial. PharmacoEconomics - Open. 2019;3(4):495-504.
16. Fors A, Swedberg K, Ulin K, Wolf A, Ekman I. Effects of person-centred care after an event of acute coronary syndrome: Two-year follow-up of a randomised controlled trial. International Journal of Cardiology. 2017;249:42-7.
17. Fors A, Ekman I, Ulin K, Wolf A, Swedberg K. Person-centred care is effective after an event of acute coronary syndrome; particularly in patients with low educational level-two-year follow-up of a randomised controlled trial. European Heart Journal. 2017;38 (Supplement 1):116.
18. Martin IR, McNamara D, Sutherland FR, Tilyard MW, Taylor DR. Care plans for acutely deteriorating COPD: a randomized controlled trial. Chronic respiratory disease. 2004;1(4):191-5.
19. Sommers LS, Marton KI, Barbaccia JC, Randolph J. Physician, nurse, and social worker collaboration in primary care for chronically ill seniors. Archives of Internal Medicine. 2000;160(12):1825-33.
20. Fors A, Blanck E, Ali L, Swedberg K, Ekman I. Person-centred telephone-support is effective in patients with chronic obstructive pulmonary disease and/or chronic heart failure-six-month follow-up of a randomized controlled trial. European Journal of Heart Failure. 2018;20 (Supplement 1):194.
21. Alamo MM, Moral RR, Perula de Torres LA. Evaluation of a patient-centred approach in generalized musculoskeletal chronic pain/fibromyalgia patients in primary care. Patient Education & Counseling. 2002;48(1):23-31.
22. Feldthusen C, Dean E, Forsblad-d'Elia H, Mannerkorpi K. Effects of Person-Centered Physical Therapy on Fatigue-Related Variables in Persons With Rheumatoid Arthritis: A Randomized Controlled Trial. Arch Phys Med Rehabil. 2016;97(1):26-36.
23. Ko FWS, Cheung NK, Rainer TH, Lum C, Wong I, Hui DSC. Comprehensive care programme for patients with chronic obstructive pulmonary disease: A randomised controlled trial. Thorax. 2017;72(2):122-8.
24. Fors A, Taft C, Ulin K, Ekman I. Person-centred care improves self-efficacy to control symptoms after acute coronary syndrome: A randomized controlled trial. European Journal of Cardiovascular Nursing. 2016;15(2):186-94.
25. Fors A, Gyllensten H, Swedberg K, Ekman I. Effectiveness of person-centred care after acute coronary syndrome in relation to educational level: Subgroup analysis of a two-armed randomised controlled trial. Int J Cardiol. 2016;221:957-62.
26. Olsson L-E, Karlsson J, Berg U, Kärrholm J, Hansson E. Person-centred care compared with standardized care for patients undergoing total hip arthroplasty—a quasi-experimental study. Journal of Orthopaedic Surgery and Research. 2014;9(1):95.
27. de Batlle J, Massip M, Vargiu E, Nadal N, Fuentes A, Ortega Bravo M, et al. Implementing Mobile Health-Enabled Integrated Care for Complex Chronic Patients: Intervention Effectiveness and Cost-Effectiveness Study. JMIR Mhealth Uhealth. 2021;9(1):e22135.
28. Yu C, Choi D, Bruno BA, Thorpe KE, Straus SE, Cantarutti P, et al. Impact of MyDiabetesPlan, a Web-Based Patient Decision Aid on Decisional Conflict, Diabetes Distress, Quality of Life, and Chronic Illness Care in Patients With Diabetes: Cluster Randomized Controlled Trial. J Med Internet Res. 2020;22(9):e16984.

For peer review only

29. Chochinov HM, Kristjanson LJ, Breitbart W, McClement S, Hack TF, Hassard T, et al. Effect of dignity therapy on distress and end-of-life experience in terminally ill patients: a randomised controlled trial. Lancet Oncology. 2011;12(8):753-62.
30. Kennedy A, Nelson E, Reeves D, Richardson G, Roberts C, Robinson A, et al. A randomised controlled trial to assess the impact of a package comprising a patient-orientated, evidence-based self-help guidebook and patient-centred consultations on disease management and satisfaction in inflammatory bowel disease. Health Technology Assessment. 2003;7(28).
31. Bökberg C, Behm L, Wallerstedt B, Ahlström G. Evaluation of person-centeredness in nursing homes after a palliative care intervention: pre- and post-test experimental design. BMC Palliative Care. 2019;18(1):44.
32. Mielenz TJ, Tracy M, Jia H, Durbin LL, Allegrante JP, Arniella G, et al. Creation of the Person-Centered Wellness Home in Older Adults. Innovation in Aging. 2020;4(1).
33. Wichit N, Mnatzaganian G, Courtney M, Schulz P, Johnson M. Randomized controlled trial of a family-oriented self-management program to improve self-efficacy, glycemic control and quality of life among Thai individuals with Type 2 diabetes. Diabetes Research and Clinical Practice. 2017;123:37-48.
34. Yu DSF. Effects of a Health and Social Collaborative Case Management Model on Health Outcomes of Family Caregivers of Frail Older Adults: Preliminary Data from a Pilot Randomized Controlled Trial. Journal of the American Geriatrics Society. 2016;64(10):2144-8.
35. Jutterstrom L, Hornsten A, Sandstrom H, Stenlund H, Isaksson U. Nurse-led patient-centered self-management support improves HbA1c in patients with type 2 diabetes-A randomized study. Patient Education and Counseling. 2016;99(11):1821-9.
36. Larsson I, Fridlund B, Arvidsson B, Teleman A, Bergman S. Treatment outcomes from a nurse-led rheumatology clinic in monitoring of anti-TNF therapy-a randomised controlled trial. Arthritis and Rheumatism. 2012;10):S667.
37. Hansson E, Carlström E, Olsson LE, Nyman J, Koinberg I. Can a person-centred-care intervention improve health-related quality of life in patients with head and neck cancer? A randomized, controlled study. BMC Nurs. 2017;16:9.
38. Bertilsson AS, Eriksson G, Ekstam L, Tham K, Andersson M, von Koch L, et al. A cluster randomized controlled trial of a client-centred, activities of daily living intervention for people with stroke: one year follow-up of caregivers. Clinical rehabilitation. 2016;30(8):765-75.
39. Bertilsson A-S, Ranner M, von Koch L, Eriksson G, Johansson U, Ytterberg C, et al. A client-centred ADL intervention: three-month follow-up of a randomized controlled trial. Scandinavian journal of occupational therapy. 2014;21(5):377-91.
40. Guidetti S, Ranner M, Tham K, Andersson M, Ytterberg C, von Koch L. A "Client-Centred Activities of Daily Living" Intervention for Persons with Stroke: One-Year Follow-up of a Randomized Controlled Trial. Journal of Rehabilitation Medicine. 2015;47(7):605-11.
41. Wolf A, Fors A, Ulin K, Thorn J, Swedberg K, Ekman I. An eHealth Diary and Symptom-Tracking Tool Combined With Person-Centered Care for Improving Self-Efficacy After a Diagnosis of Acute Coronary Syndrome: A Substudy of a Randomized Controlled Trial. J Med Internet Res. 2016;18(2):e40.
42. Larsson A, Palstam A, Löfgren M, Ernberg M, Bjersing J, Bileviciute-Ljungar I, et al. Resistance exercise improves muscle strength, health status and pain intensity in fibromyalgia--a randomized controlled trial. Arthritis Res Ther. 2015;17(1):161.

For peer review only

43. Ericsson A, Palstam A, Larsson A, Löfgren M, Bileviciute-Ljungar I, Bjersing J, et al. Resistance exercise improves physical fatigue in women with fibromyalgia: a randomized controlled trial. Arthritis Res Ther. 2016;18:176.
44. Ekman I, Wolf A, Olsson LE, Taft C, Dudas K, Schaufelberger M, et al. Effects of person-centred care in patients with chronic heart failure: the PCC-HF study. European Heart Journal. 2012;33(9):1112-9.
45. Fors A, Ekman I, Taft C, Björkelund C, Frid K, Larsson ME, et al. Person-centred care after acute coronary syndrome, from hospital to primary care - A randomised controlled trial. Int J Cardiol. 2015;187:693-9.
46. Hansson E, Ekman I, Swedberg K, Wolf A, Dudas K, Ehlers L, et al. Person-centred care for patients with chronic heart failure - a cost-utility analysis. Eur J Cardiovasc Nurs. 2016;15(4):276-84.
47. Sahlen K-G, Boman K, Brannstrom M. A cost-effectiveness study of person-centered integrated heart failure and palliative home care: Based on a randomized controlled trial. Palliative Medicine. 2016;30(3):296-302.
48. Ulin K, Olsson LE, Wolf A, Ekman I. Person-centred care - An approach that improves the discharge process. European journal of cardiovascular nursing : journal of the Working Group on Cardiovascular Nursing of the European Society of Cardiology. 2016;15(3):e19-e26.
49. Brannstrom M, Boman K. Effects of person-centred and integrated chronic heart failure and palliative home care. PREFER: a randomized controlled study. European Journal of Heart Failure. 2014;16(10):1142-51.
50. Dudas K, Olsson LE, Wolf A, Swedberg K, Taft C, Schaufelberger M, et al. Uncertainty in illness among patients with chronic heart failure is less in person-centred care than in usual care. European Journal of Cardiovascular Nursing. 2013;12(6):521-8.
51. Olsson LE, Hansson E, Ekman I. Evaluation of person-centred care after hip replacement-a controlled before and after study on the effects of fear of movement and self-efficacy compared to standard care. BMC Nurs. 2016;15(1):53.
52. Berendonk C, Kaspar R, Bär M, Hoben M. Improving Quality of Work life for Care Providers by Fostering the Emotional well-being of Persons with Dementia: A Cluster-randomized Trial of a Nursing Intervention in German long-term Care Settings Dementia 2019;18(4):1286-309.
53. Kelechi TJ, Mueller M, Spencer C, Rinard B, Loftis G. The effect of a nurse-directed intervention to reduce pain and improve behavioral and physical outcomes in patients with critically colonized/infected chronic leg ulcers. Journal of Wound, Ostomy, & Continence Nursing. 2014;41(2):111-21.
54. Goelz T, Wuensch A, Stubenrauch S, Ihorst G, de Figueiredo M, Bertz H, et al. Specific training program improves oncologists' palliative care communication skills in a randomized controlled trial. Journal of Clinical Oncology. 2011;29(25):3402-7.
55. Wolff JL, Giovannetti ER, Boyd CM, Reider L, Palmer S, Scharfstein D, et al. Effects of guided care on family caregivers. The Gerontologist. 2010;50(4):459-70.
56. Britt HR, JaKa, M. M., Fernstrom, K. M., Bingham, P. E., Betzner, A. E., Taghon, J. R., Shippee, N. D., Shippee, T. P., Schellinger, S. E., & Anderson, E. W. . Quasi-Experimental Evaluation of LifeCourse on Utilization and Patient and Caregiver Quality of Life and Experience. . The American journal of hospice & palliative care 2019;36(5):408-16.

For peer review only

57. Slok AH, Kotz D, van Breukelen G, Chavannes NH, Rutten-van Molken MP, Kerstjens HA, et al. Effectiveness of the Assessment of Burden of COPD (ABC) tool on health-related quality of life in patients with COPD: a cluster randomised controlled trial in primary and hospital care. BMJ Open. 2016;6(7):e011519.
58. Windrum P, Garcia-Goni M, Coad H. The Impact of Patient-Centered versus Didactic Education Programs in Chronic Patients by Severity: The Case of Type 2 Diabetes Mellitus. Value in Health. 2016;19(4):353-62.
59. Armstrong KA, Coyte PC, Brown M, Beber B, Semple JL. Effect of home monitoring via mobile app on the number of in-person visits following ambulatory surgery a randomized clinical trial. JAMA Surgery. 2017;152(7):622-7.
60. Öhlén J, Sawatzky R, Pettersson M, Sarenmalm EK, Larsdotter C, Smith F, et al. Preparedness for colorectal cancer surgery and recovery through a person-centred information and communication intervention - A quasi-experimental longitudinal design. PLoS One. 2019;14(12):e0225816.
61. WHO. Quality of care: a process for making strategic choices in health systems.: WHO; 2006 [
62. Ulin K, Olsson LE, Wolf A, Ekman I. Person-centred care - An approach that improves the discharge process. European Journal of Cardiovascular Nursing. 2016;15(3):e19-26.
63. Eggers C, Dano R, Schill J, Fink GR, Hellmich M, Timmermann L. Patient-centered integrated healthcare improves quality of life in Parkinson's disease patients: a randomized controlled trial. J Neurol. 2018;265(4):764-73.
64. Arian M, Memarian R, Oghazian MB, Vakilian F, Badiee Z. The effect of a holistic care program on the reduction of iron over load in patients with beta-thalassemia major: A randomized clinical trial. Iranian Red Crescent Medical Journal. 2018;20 (4) (no pagination)(e60820).
65. Murphy SL, Lyden AK, Smith DM, Dong Q, Koliba JF. Effects of a tailored activity pacing intervention on pain and fatigue for adults with osteoarthritis. American Journal of Occupational Therapy. 2010;64(6):869-76.
66. Or C, Tao D. A 3-Month Randomized Controlled Pilot Trial of a Patient-Centered, Computer-Based Self-Monitoring System for the Care of Type 2 Diabetes Mellitus and Hypertension. Journal of Medical Systems. 2016;40(4):81.
67. Reed RL, Roeger L, Howard S, Oliver-Baxter JM, Battersby MW, Bond M, et al. A self-management support program for older Australians with multiple chronic conditions: A randomised controlled trial. Medical Journal of Australia. 2018;208(2):69-74.
68. Kikkenborg Berg S, Stoier L, Moons P, Zwisler AD, Winkel P, Ulrich Pedersen P. Emotions and health: findings from a randomized clinical trial on psychoeducational nursing to patients with implantable cardioverter defibrillator. The Journal of cardiovascular nursing. 2015;30(3):197-204.
69. Low LL, Tan SY, Ng MJM, Tay WY, Ng LB, Balasubramaniam K, et al. Applying the Integrated Practice Unit Concept to a Modified Virtual Ward Model of Care for Patients at Highest Risk of Readmission: A Randomized Controlled Trial. PloS one. 2017;12(1):e0168757-e.
70. Gustafson D, Hawkins R, Boberg E, Bricker E, Pingree S, Chan C-L. The use and impact of a computer-based support system for people living with AIDS and HIV infectionAnnual Symposium on Computer Application [sic] in Medical Care Symposium on Computer Applications in Medical Care. 1994;1(2):604-8.

For peer review only

PRISMA 2020 Checklist

Section and Topic	Item #	Checklist item	Location where item is reported
**TITLE**			
Title	1	Identify the report as a systematic review.	1
**ABSTRACT**			
Abstract	2	See the PRISMA 2020 for Abstracts checklist.	2
**INTRODUCTION**			
Rationale	3	Describe the rationale for the review in the context of existing knowledge.	8
Objectives	4	Provide an explicit statement of the objective(s) or question(s) the review addresses.	8
**METHODS**			
Eligibility criteria	5	Specify the inclusion and exclusion criteria for the review and how studies were grouped for the syntheses.	10
Information sources	6	Specify all databases, registers, websites, organisations, reference lists and other sources searched or consulted to identify studies. Specify the date when each source was last searched or consulted.	8,9
Search strategy	7	Present the full search strategies for all databases, registers and websites, including any filters and limits used.	9
Selection process	8	Specify the methods used to decide whether a study met the inclusion criteria of the review, including how many reviewers screened each record and each report retrieved, whether they worked independently, and if applicable, details of automation tools used in the process.	11
Data collection process	9	Specify the methods used to collect data from reports, including how many reviewers collected data from each report, whether they worked independently, any processes for obtaining or confirming data from study investigators, and if applicable, details of automation tools used in the process.	12
Data items	10a	List and define all outcomes for which data were sought. Specify whether all results that were compatible with each outcome domain in each study were sought (e.g. for all measures, time points, analyses), and if not, the methods used to decide which results to collect.	12
	10b	List and define all other variables for which data were sought (e.g. participant and intervention characteristics, funding sources). Describe any assumptions made about any missing or unclear information.	12
Study risk of bias assessment	11	Specify the methods used to assess risk of bias in the included studies, including details of the tool(s) used, how many reviewers assessed each study and whether they worked independently, and if applicable, details of automation tools used in the process.	12
Effect measures	12	Specify for each outcome the effect measure(s) (e.g. risk ratio, mean difference) used in the synthesis or presentation of results.	N/A
Synthesis methods	13a	Describe the processes used to decide which studies were eligible for each synthesis (e.g. tabulating the study intervention characteristics and comparing against the planned groups for each synthesis (item #5)).	13
	13b	Describe any methods required to prepare the data for presentation or synthesis, such as handling of missing summary statistics, or data conversions.	N/A
	13c	Describe any methods used to tabulate or visually display results of individual studies and syntheses.	13
	13d	Describe any methods used to synthesize results and provide a rationale for the choice(s). If meta-analysis was performed, describe the model(s), method(s) to identify the presence and extent of statistical heterogeneity, and software package(s) used.	N/A
	13e	Describe any methods used to explore possible causes of heterogeneity among study results (e.g. subgroup analysis, meta-regression).	N/A
	13f	Describe any sensitivity analyses conducted to assess robustness of the synthesized results.	N/A
Reporting bias assessment	14	Describe any methods used to assess risk of bias due to missing results in a synthesis (arising from reporting biases).	N/A
Certainty assessment	15	Describe any methods used to assess certainty (or confidence) in the body of evidence for an outcome.	N/A

For peer review only - http://bmjopen.bmj.com/site/about/guidelines.xhtml

PRISMA 2020 Checklist

Section and Topic	Item #	Checklist item	Location where item is reported
**RESULTS**			
Study selection	16a	Describe the results of the search and selection process, from the number of records identified in the search to the number of studies included in the review, ideally using a flow diagram.	22
	16b	Cite studies that might appear to meet the inclusion criteria, but which were excluded, and explain why they were excluded.	22, Figure 1
Study characteristics	17	Cite each included study and present its characteristics.	23
Risk of bias in studies	18	Present assessments of risk of bias for each included study.	14-21
Results of individual studies	19	For all outcomes, present, for each study: (a) summary statistics for each group (where appropriate) and (b) an effect estimate and its precision (e.g. confidence/credible interval), ideally using structured tables or plots.	23-122
Results of syntheses	20a	For each synthesis, briefly summarise the characteristics and risk of bias among contributing studies.	125
	20b	Present results of all statistical syntheses conducted. If meta-analysis was done, present for each the summary estimate and its precision (e.g. confidence/credible interval) and measures of statistical heterogeneity. If comparing groups, describe the direction of the effect.	N/A
	20c	Present results of all investigations of possible causes of heterogeneity among study results.	126-127
	20d	Present results of all sensitivity analyses conducted to assess the robustness of the synthesized results.	N/A
Reporting biases	21	Present assessments of risk of bias due to missing results (arising from reporting biases) for each synthesis assessed.	N/A
Certainty of evidence	22	Present assessments of certainty (or confidence) in the body of evidence for each outcome assessed.	128-135
**DISCUSSION**			
Discussion	23a	Provide a general interpretation of the results in the context of other evidence.	136
	23b	Discuss any limitations of the evidence included in the review.	138
	23c	Discuss any limitations of the review processes used.	139
	23d	Discuss implications of the results for practice, policy, and future research.	139-140
**OTHER INFORMATION**			
Registration and protocol	24a	Provide registration information for the review, including register name and registration number, or state that the review was not registered.	3,7
	24b	Indicate where the review protocol can be accessed, or state that a protocol was not prepared.	N/A
	24c	Describe and explain any amendments to information provided at registration or in the protocol.	N/A
Support	25	Describe sources of financial or non-financial support for the review, and the role of the funders or sponsors in the review.	141
Competing interests	26	Declare any competing interests of review authors.	141
Availability of data, code and other materials	27	Report which of the following are publicly available and where they can be found: template data collection forms; data extracted from included studies; data used for all analyses; analytic code; any other materials used in the review.	141

From: Page MJ, McKenzie JE, Bossuyt PM, Boutron I, Hoffmann TC, Mulrow CD, et al. The PRISMA 2020 statement: an updated guideline for reporting systematic reviews. BMJ 2021;372:n71. doi: 10.1136/bmj.n71

For peer review only - http://bmjopen.bmj.com/site/about/guidelines.xhtml

For more information, visit: http://www.prisma-statement.org/

For peer review only

BMJ Open

A systematic review of impact of person-centred interventions for serious physical illness in terms of outcomes and costs.

Journal:	*BMJ Open*
Manuscript ID	bmjopen-2021-054386.R1
Article Type:	Original research
Date Submitted by the Author:	11-May-2022
Complete List of Authors:	Bashan Nkhoma, Kennedy; King's College London, Florence Nightingale Faculty of Nursing Midwifery and Palliative Care<br>Cook, Amelia; King's College London, Cicely Saunders Institute for Palliative Care, Policy and Rehabilitation<br>Giusti, Alessandra; King's College London, Cicely Saunders Institute<br>Farrant , Lindsay ; University of Cape Town Faculty of Health Sciences, School of Public Health and Family Medicine<br>Petrus , Ruwayda; University of KwaZulu-Natal College of Humanities, School of Applied Human Sciences<br>Petersen, I.; Univ KwaZulu Natal, Centre for Rural Health<br>Gwyther, Liz; University of Cape Town Faculty of Health Sciences, School of Public Health and Family Medicine<br>Venkatapuram, Sridhar; King's College London,<br>Harding, Richard; King's College London, of Palliative Care, Policy and Rehabilitation ;
**Primary Subject Heading**:	Health services research
Secondary Subject Heading:	Palliative care, Global health
Keywords:	Health policy < HEALTH SERVICES ADMINISTRATION & MANAGEMENT, Quality in health care < HEALTH SERVICES ADMINISTRATION & MANAGEMENT, Clinical trials < THERAPEUTICS, Health economics < HEALTH SERVICES ADMINISTRATION & MANAGEMENT

SCHOLARONE™
Manuscripts

I, the Submitting Author has the right to grant and does grant on behalf of all authors of the Work (as defined in the below author licence), an exclusive licence and/or a non-exclusive licence for contributions from authors who are: i) UK Crown employees; ii) where BMJ has agreed a CC-BY licence shall apply, and/or iii) in accordance with the terms applicable for US Federal Government officers or employees acting as part of their official duties; on a worldwide, perpetual, irrevocable, royalty-free basis to BMJ Publishing Group Ltd ("BMJ") its licensees and where the relevant Journal is co-owned by BMJ to the co-owners of the Journal, to publish the Work in this journal and any other BMJ products and to exploit all rights, as set out in our licence.

The Submitting Author accepts and understands that any supply made under these terms is made by BMJ to the Submitting Author unless you are acting as an employee on behalf of your employer or a postgraduate student of an affiliated institution which is paying any applicable article publishing charge ("APC") for Open Access articles. Where the Submitting Author wishes to make the Work available on an Open Access basis (and intends to pay the relevant APC), the terms of reuse of such Open Access shall be governed by a Creative Commons licence – details of these licences and which Creative Commons licence will apply to this Work are set out in our licence referred to above.

Other than as permitted in any relevant BMJ Author's Self Archiving Policies, I confirm this Work has not been accepted for publication elsewhere, is not being considered for publication elsewhere and does not duplicate material already published. I confirm all authors consent to publication of this Work and authorise the granting of this licence.

For peer review only

A systematic review of impact of person-centred interventions for serious physical illness in terms of outcomes and costs.

Kennedy Bashan Nkhoma[1], Amelia Cook[1], Alessandra Giusti[1], Lindsay Farrant[2], Ruwayda Petrus[3], Inge Petersen[4], Liz Gwyther[2], Sridhar Venkatapuram[5], Richard Harding[1]

Affiliations

1. King's College London, Cicely Saunders Institute of Palliative Care, Policy and Rehabilitation, London, SE5 9PJ, United Kingdom

2. University of Cape Town, School of Public Health and Family Medicine (Palliative Medicine) Falmouth Building, Cape Town, Western Cape, South Africa

3. University of KwaZulu-Natal, College of Humanities, School of Applied Human Sciences Durban, ZA 4001, South Africa

4. University of KwaZulu Natal, Centre for Rural Health, Howard College, Durban, ZA 4001, South Africa

5. King's College London, King's Global Health Institute, Franklin Wilkins Building, 150 Stamford Street, London, UK SE1 9NH

Correspondence: Dr Kennedy B. Nkhoma

Email: kennedy.nkhoma@kcl.ac.uk Telephone: +44 (0)207 848 5566

A systematic review of impact of person-centred interventions for serious physical illness in terms of outcomes and costs.

Background

Person-centred care (PCC) is being internationally recognised as a critical attribute of high-quality healthcare. The International Alliance of Patients Organisations defines PCC as care that is focused and organised around people, rather than disease. Focusing on delivery, we aimed to review and evaluate the evidence from interventions that aimed to deliver PCC for people with serious physical illness and identify models of PCC interventions.

Methods

Systematic review of literature using PRISMA guidelines. We searched Amed, CINAHL, Cochrane Library, Embase, Medline, PsycInfo, using the following key concepts: Patient/person-centred care, family-centred care, family-based care, individualised care, holistic care, serious illness, chronic illness, long term conditions from inception to April 2022. Due to heterogeneity of interventions and populations studied, narrative synthesis was conducted. Study quality was appraised using the Joanna Briggs checklist.

Results

We screened n=6156 papers. Seventy-two papers (reporting n=55 different studies) were retained in the review. Most of these studies n=47 studies were RCT's. Our search yielded two main types of interventions: 1) studies with self-management components and 2) technology-based interventions. We synthesised findings across these two models:

Self-management component. The interventions consisted of training of patients and/or caregivers or staff. Some studies reported that interventions had effect in reduction hospital admissions, improving quality of life and reducing costs of care.

Technology-based interventions: consisted of mobile phone, mobile app, tablet/computer, and video. Although some interventions showed improvements for self-efficacy,

For peer review only - http://bmjopen.bmj.com/site/about/guidelines.xhtml

hospitalisations and length of stay, quality of life did not improve across most studies.

Discussion

PCC interventions using self-management have some effects in reducing costs of care and improving quality of life. Technology-based interventions improves self-efficacy but has no effect on quality of life. However very few studies used self-management and technology approaches. Further work is needed to identify how self-management and technology approaches can be used to manage serious illness.

Funding: National Institute of Health and Care Research (NIHR) Global Health Research Unit on Health System Strengthening in sub-Saharan Africa, King's College London (GHRU 16/136/54) using UK aid from the UK Government to support global health research.

Review Registration number: PROSPERO CRD42018108302

Key words: Person-centred care, Serious physical illness, Systematic Reviews, Self-management interventions, technology-based interventions

For peer review only

Strengths and limitations of this study

- A study provides a systematic review of the evidence on the impact of person-centred interventions for serious physical illness in terms of outcomes and costs.
- We used robust procedures for systematic reviewing and quality assessment of the studies included, in line with Preferred Reporting Items for Systematic Reviews and Meta-Analyses (PRISMA) reporting guidelines.
- Most of the studies identified and included were conducted in high income countries (HIC).
- We conducted a narrative synthesis due to heterogenicity of the studies included (different disease population, different outcome measures and different trial end points).
- Most of the studies included did not state the theoretical framework underpinning the person-centred interventions. However, many studies that reported the theoretical framework used the Goldenberg theory of person-centred care and were conducted in Sweden across various clinical settings.

For peer review only

For peer review only - http://bmjopen.bmj.com/site/about/guidelines.xhtml

Introduction

World Health Organization (WHO) guidance emphasise person-centredness as a core component of health professionals' skills and quality health-care (1). Integrated, person-centred care (PCC) is essential to achieving Universal Health Coverage (UHC) (2, 3). The core elements of person-centred care (PCC) in health policy, medicine and nursing have been described as: patient participation and involvement, patient relationship with the healthcare professionals and context where care is delivered (4). The International Alliance of Patients' Organisations defines person-centred (or patient-centred) care (PCC) as "focused and organised around people, rather than disease"(5). PCC views individuals, families and communities as participants in health systems responsive to their needs(6) Person-centred care aims to give meaningful assessment and equal weight to a patient's subjective understanding of their illness, including their needs, concerns, and expectations. This occurs, alongside a biomedical diagnosis; PCC also promote their equal participation in treatment decision-making and empowers them to take greater control of their own health and management of their condition (7).

Our first systematic review identified and appraised the empirical evidence underpinning conceptualisations of 'person centredness' for serious illness (8). Serious illness, as defined in that review, includes conditions that carry a high degree of clinical uncertainty, may require high care dependency because of decreased function, but may not be advanced (9). The review concluded that PCC (through valuing the social needs of patients, promoting quality of life, and reform of health structures) will improve patients' experience of interaction with healthcare systems (8). The review also concluded that primary data are needed that investigate the meaning and practice of PCC in a diverse diagnostic groups and settings (8). Re-engineering health systems to deliver PCC has particular relevance to low- and middle-income countries (LMIC) (6, 10). Serious health-related suffering places a huge burden on health systems, with the greatest burden in LMIC. Projections from WHO mortality data estimate that LMIC face the largest proportional increase, largely due to ageing (155% increase in people with serious health related suffering in the last year of life by 2060 to 5.14

For peer review only - http://bmjopen.bmj.com/site/about/guidelines.xhtml

million people) (11). In such contexts, serious illness also places huge psychological, social, economic, physical, and spiritual burdens on patients and (largely female) family caregivers. (12-14). It carries a high risk of mortality, negatively impacts quality of life and daily function, and is burdensome in symptoms, treatments and or caregiver stress (15).

PCC has great potential for patients, families, staff and the health care system in terms of engagement, enablement, management of symptoms and reduction in re-referrals, reducing readmission, frequent visits to primary care and/or emergency visits (16). Identification, refinement, adaptation, and implementation of effective PCC interventions may thus help to achieve the WHO and Universal Health Coverage goals. However, no review to date has aimed to identify and synthesise the evidence for the outcomes and cost of PCC across serious physical illness. We aimed to review the evidence (in terms of outcomes and costs) for interventions that aim to deliver person-centred care to, or enhance person-centredness of care for, adults with serious physical illness.

Methods

Design

Systematic review of peer-reviewed literature drawing on PRISMA guidelines, with quality appraisal using the Joanna Briggs Institute critical appraisal checklist, and narrative synthesis of findings. A full protocol is registered with PROSPERO, CRD42018108302 (17).

Objectives

The objectives of this review were to i) identify models of person-centred care interventions for adults with serious illness and how these were delivered.

ii) determine which outcomes have been measured as endpoints; iii) appraise intervention effectiveness in terms of outcomes and costs, using narrative synthesis; iv) evaluate the quality of the evidence.

For peer review only

Search strategy

The following databases were searched in January 2020: Amed, Assian, Cumulative Index to Nursing and Allied Health Literature (CINAHL), Cochrane Library, Embase, Medline, PsycInfo, Scopus and Web of Science. Key journals and reference lists from included studies and relevant review articles were hand searched. We conducted a search rerun limiting it from 2020 to April 2022 (supplementary file 1 has the details).

The search strategy (Table 1) was developed in consultation with an information specialist. We used the following key concepts, drawing on our prior review of the concepts and primary data underpinning person-centred care (8): person/patient-centred care, family-centred care, family-based care, individualised care, holistic care. Data bases were searched from inception.

Reference lists of identified papers and previous systematic reviews on person-centred care were hand searched.

Table 1: Search strategy

Search strategy number	Key concepts	Key words
1	Patient centred<br>Family centred<br>Person centred<br>Individualised<br>Holistic	Patient-centered care or patient-centred care or client-centred care or client-centered care or client-focused care or person-centred care or person-centered care or person-focused care or family-centred care or family-focused care or family-centered care or individuali?ed or holistic care or holistic health
2	Serious illness<br>Chronic illness<br>Long term illness	chronic diseases or serious illness or chronic illness or long term conditions or long term illness

For peer review only - http://bmjopen.bmj.com/site/about/guidelines.xhtml

Subject headings and word truncations were entered according to requirements of each database to map all potential keywords. Group 1 concepts were combined using the 'OR' function. Likewise group 2 concepts were combined using OR function. Finally search strategies 1 and 2 were intersected using the 'AND' function

Eligibility criteria

The inclusion and exclusion criteria are summarised in table 2 below:

For peer review only

Table 2: Inclusion and exclusion criteria

	**Inclusion**	**Exclusion**
Participants	All serious physical illness as defined by Kelly et al 2014; 2016: *Serious illness is a health condition that carries a high risk of mortality AND either negatively impacts a person's daily function or quality of life, OR excessively strains their caregivers*.<br><br>Caregivers of patients with serious physical illness defined above.<br><br>Health care professionals (doctors, nurses, social workers etc) caring for patients with serious physical illness.	Patients with conditions considered risk factors to develop serious illness such as hypertension.
Interventions	Any interventions delivered using a person-centred, or client-centred, or patient-centred, or family centred approach such as involving patients in decision-making about their care, setting goals and plans, patient being involved managing their own disease, interventions focused on the whole	Any interventions delivered without patient involvement or decision making about their care or helping them take actions to support themselves.

For peer review only - http://bmjopen.bmj.com/site/about/guidelines.xhtml

	person, holistic approach. Interventions delivered in any format that is focused on the needs of the patients.	
Studies and comparator	Published intervention studies<br>Written in English language only<br>Evaluations using a comparator.<br>The comparison group should either be usual care/standard care, or a comparator intervention.	Unpublished studies, studies not written in English language, conference proceedings, conference abstracts,<br>Non-randomised trials<br>No comparison group.
Outcomes	Patient and family caregiver self-report outcomes, e.g.:<br>-pain and symptom prevalence and intensity/severity, interference with daily activities, knowledge and practice of self-management, quality of life;<br>-psychosocial outcomes such as stress, anxiety, depression, burnout, distress.<br>-social, practical, and spiritual; knowledge of pain and/or symptom management, quality of life, psychological outcomes (anxiety, stress, depression, distress) and caregiver motivation to provide care.<br><br>Formal and informal health service use<br>Costs of services.	Outcomes not related to person-centred care (outcomes not focusing on physical, psychological social and spiritual aspects of care).

Selection of studies, data collection and management

We report the search strategy process using the PRISMA flow chart (18). All references identified by the search strategy were exported to Endnote software and deduplicated. One reviewer (KN) independently appraised all titles and abstracts against the inclusion and exclusion criteria. If the title and abstract was obviously irrelevant, the reference was excluded at this stage. Full text retained references were obtained and appraised against

For peer review only - http://bmjopen.bmj.com/site/about/guidelines.xhtml

inclusion and exclusion criteria, and if the decision was unclear this was discussed with a second reviewer (AC) and if necessary adjudicated by a third (RH).

Data extraction

KN and AC extracted study data using methods described in the Cochrane handbook for systematic reviews of interventional studies (19). A standardised data extraction form was used to ensure consistency in the review (20). KN extracted n=46 papers and AC extracted n=26 papers, then both authors peer reviewed data extraction. Any queries were resolved through discussion. RH reviewed the final data extraction.

The following variables were extracted: authors, year of publication, aims and objectives, setting and country, study design, selection of participants, sample characteristics, randomisation procedures, blinding of participants and outcome assessors, assessment of outcomes and measures used, description of the intervention and comparison group, intervention delivery, sample size, data analysis, loss to follow-up, findings for outcomes and costs, and study conclusions (supplementary file 2).

Assessment of methodological quality of the studies

We applied the Joanna Briggs Institute Critical Appraisal checklist for Randomised and non-Randomised trials to assess methodological quality of the studies (21). These are summarised in supplementary file 3. This was conducted at individual study level. AC and KN assessed each study independently, and thereafter discussed critical appraisal. Discrepancies in the assessment of quality between AC and KN were resolved by discussion, and RH checked the critical appraisals of the papers.

Synthesis of the evidence

Due to heterogeneity of the studies, interventions, participants, and outcomes a meta-synthesis was not conducted. We performed narrative synthesis to synthesize the findings of

For peer review only

the different studies using the Guidance on the Conduct of Narrative Synthesis in Systematic Reviews, which consists of four elements: 1) the role of theory in evidence synthesis, 2) developing a preliminary synthesis, 3) exploring relationships within and between studies and 4) assessing the robustness of the synthesis (22).

We developed two logic models (Figures 1 and 2) to summarise the context, study population, to describe the intervention components, mechanism of action, and outcomes. Figure 1 contains studies which reported a theory or conceptual framework which informed the development of the intervention. Figure 2 reports studies which did not state a theory or conceptual framework of the intervention.

A preliminary synthesis was undertaken in form of a thematic analysis involving listing and presenting results in tabular form. The results of the included studies were summarised in a narrative synthesis within a framework (participants, study aims, intervention description, usual care description, outcomes and measures used as presented in supplementary file 2. For each study the effects of the intervention on the outcomes tested in provided.

We explored relationships in the data, for example similar study design use (RCT vs non-RCT), similar methods of randomisation, similar intervention components and mode of delivery and similar outcomes. We then made conclusions based on the robustness of the synthesis and the quality of evidence.

Patient and public involvement

Patient and public involvement was not conducted as part of this review.

For peer review only

Results

The PRISMA flow diagram (figure 3) presents the results of the search strategy. After deduplication, we screened n=5302 papers (title, abstract) and n=95 papers were retained for full text screening. Of these, n=23 were excluded (reasons are reported in the flow chart) and n=72 papers (reporting 55 different studies) were retained in the review.

Characteristics of the included studies

The n=56 studies included were conducted in 17 countries, the majority were high-income countries (n=13/17). Studies were conducted predominantly in Sweden n=16, USA n=12, Canada n=4, Germany n=4, Australia n=3, Hong Kong=3, UK=3 and Spain n=2. One study was conducted in each of the following countries: Brasil, Denmark, Iran, Kenya, Netherlands New Zealand, Norway, Singapore, and Thailand. A further study was multi-country, conducted in Canada, Australia, and USA. Table 3 summaries number of studies conducted in each country.

Table 3: Studies and countries

Country	Number of studies	References
Sweden	16 with 31references/papers	(23-53)
USA	12 with 13 references/papers	(54-66),
Canada	4	(57, 67-69)
Germany	4	(70-73)
Australia	3 with 4 references/papers	(57, 74-76)
Hong Kong	3	(77-79)
UK	3	(80-82)
Spain	2	(83, 84)
Brasil	1	(85)
Denmark	1	(86)
Iran	1	(87)

For peer review only - http://bmjopen.bmj.com/site/about/guidelines.xhtml

Kenya	1	(88)
Netherlands	1	(89)
New Zealand	1	(90)
Norway	1	(91)
Singapore	1	(92)
Thailand	1	(93)
Australia, Canada, and USA	1	(57)

For peer review only

Study designs

Of the n=55 included studies, n=47 were randomised controlled trials (RCT), pre-and post-test experimental/controlled before and after design (31, 46, 48, 49, 55, 94), quasi-experimental study designs (32, 50, 51, 62), a comparative study (54) and a geographically controlled study (74). Of the n=47 RCT's, n=11 were clustered trials (58, 60, 63, 68, 71, 81-83, 89, 95).

Diagnostic groups

The interventions addressed the following diagnostic groups: n=12 heart failure (23-25, 28, 34, 37-40, 46, 86, 96), n=9 T2D (26, 63, 68, 74, 77, 80, 82, 93), n=8 COPD (28, 35, 59, 65, 79, 84, 89, 90), n=5 cancer (32, 41, 67, 70, 76), n=6 multimorbidity (55, 64, 69, 71, 75, 91), n=3 fibromyalgia (44, 45, 83), n=3 rheumatoid arthritis (30, 36, 52), n=2 HIV (66, 88), n=1 back pain (85) n=1 inflammatory bowel disease (81), n=1 osteoarthritis (61), n=1 Stroke (27), n=1 chronic pain (58), n=1 dementia (72), Parkinson disease (73) and n=1 beta-thalassaemia major (87).

Intervention target & delivery

The interventions were nurse-led (30, 54, 59, 60, 72, 76, 88, 90, 92), nurse and physiotherapist-led (28), nurse, physician and social worker-led (23, 55, 69, 91).

The targets of the interventions were patient and caregiver dyads (60, 78, 84, 93) or delivered to both patients and health professionals (26, 58, 65, 68, 69, 81, 83, 89) in T2D (26, 68), chronic pain/fibromyalgia (58, 83), COPD (65, 89) inflammatory bowel disease (81) and multimorbidity (69) populations. The interventions were technology-based involving a tablet computer or mobile phone (63, 68, 76, 77, 79, 81, 84, 92) or delivered to professionals such as doctors, nurses, social workers (25, 37, 68, 70, 82) working with heart failure (25, 37), T2D (68, 82) and cancer (70) patients.

For peer review only

Intervention components and delivery

Interventions delivered to health professionals (nurses, doctors, physiotherapists) consisted of training, mentorship and support through lecturers, seminars and/or workshops in the philosophy and delivery of person-centred care (25-28, 30, 31, 33-35, 54, 57, 58, 62, 68-70, 72, 80-82, 88-90, 96) for example clinical consultations using person-centred approach, person-centred communication and patient centred self-management approach (26, 32, 35, 63, 64, 69, 70, 81, 82, 84, 90). Health professionals then implemented what they learnt as they provided care to the patients and/or families.

Interventions delivered to patients and/or caregivers consisted of information provision, education, and training (32, 50-52, 58-61, 65, 77-80, 84-87, 93). The interventions were either individualised and delivered face-to-face (26, 65, 79) or delivered in groups (26, 59, 85). Educational materials, information leaflets, booklets, brochures were provided to participants (31, 32, 54, 77, 80, 82). Some interventions delivered to patients focused on developing or creating a health plan. Participants identified or set aims or goals with targets to achieve and patients identified resources and tools to achieve the targets. Health professionals worked with patients to achieve the targets and care was provided in line with patient needs and wants and what matters to them (23, 25, 28, 35, 36, 41, 44, 46, 48-51, 55, 58, 62, 64, 65, 68, 71, 74, 75, 81, 90-92, 94, 96). The health plan was reviewed and revised when necessary.

Interventions were delivered either in nursing homes (31) primary care/outpatient care (30, 36, 41, 58, 65, 68, 69, 71, 75, 79, 84, 88, 91), surgical departments (32, 50, 51, 67, 76), inpatient facilities (27, 33, 34, 46, 48, 49, 56, 94) or in home and/or community settings (23, 24, 27, 44, 55, 59, 62, 64, 66, 73, 84, 92, 93).

Some interventions involved using mobile technology (35, 41, 55, 65, 76, 79, 81, 92, 93), mobile app (67) to contact patients at home. In some studies patients in the intervention arm used either mobile-based or web-based eHealth tool preinstalled or downloaded it to use on their own mobile (40) or a tablet computer to self-monitor blood glucose and blood pressure (77), or a web-based patient decision aid to populate their cardiometabolic and psychosocial

For peer review only - http://bmjopen.bmj.com/site/about/guidelines.xhtml

profiles and general care priorities (68) or to complete self-assessments using a computer touch screen and to develop a self-management action plan (63).

Risk of bias of the studies included in the review

The majority of the studies (n=42) stated the method of randomisation, although this was not clearly stated in n=13 studies (23, 27, 58, 60, 63, 67, 74, 80, 81, 83, 84, 86, 90). Twenty-eight studies achieved allocation concealment, however n=19 did not clearly state allocation concealment (23, 25, 27, 54-56, 60, 61, 64, 68, 74, 78, 80, 81, 83, 84, 86, 87, 90). Blinding of participants was reported in only three studies (58, 75, 93). Blinding of outcome assessors was reported in n=21 studies (28, 30, 31, 36, 45, 57-61, 64, 68, 75, 78, 79, 83, 85-87, 92, 93), two studies stated that patients self-completed outcomes by post or through a web-based survey (35, 84), while n=20 studies did not clearly state if outcome assessors were blinded. With respect to follow-up data collection, n=34 studies retained at least 80% participants to the final point of data collection. In n=19 studies details were lacking regarding what constitutes usual care (24, 28, 31, 36, 46, 55, 60, 66, 71, 72, 74, 76, 78, 83, 84, 86, 87, 90, 92). The following studies included all participants including those who withdraw from the study in data analysis (25, 28, 29, 65, 69, 70, 79, 81, 83, 87, 93, 97).

Outcomes assessed

For patient outcomes quality of life was reported in n=22 studies (23, 24, 28, 34, 55, 58, 59, 63, 65, 68, 69, 71, 73, 74, 79, 81, 82, 84, 88-90, 93). These studies were conducted in COPD (28, 59, 65, 79, 84, 89, 90), T2D (63, 68, 74, 82, 93), heart failure (23, 24), chronically ill elderly (55, 71), HIV (88), acute respiratory syndrome (34), chronic pain (58), Parkinson's disease (73) Inflammatory bowel disease (81) and multimorbidity (69) populations.
General symptom burden was reported in n=4 studies in heart failure, chronically ill elderly, COPD and cancer (24, 55, 57, 65). Fatigue symptom was reported in n=4 studies among patients with rheumatoid arthritis, COPD, stroke, chronic illnesses (elderly populations) (28,

For peer review only

For peer review only - http://bmjopen.bmj.com/site/about/guidelines.xhtml

36, 64, 98). Dyspnoea symptom was reported in n=3 COPD studies (28, 59, 79), while only one study reported data on sleep disturbance (64).

Pain outcomes (severity/intensity, interference and disability) were reported in nine studies (52-54, 58, 61, 64, 83, 85, 88), among patients with chronic inflammatory arthritis (52, 53, 61), chronic pain, low back pain, infected chronic ulcers (58, 85), HIV (88), multiple chronic diseases (64), and fibromyalgia (83). Nine studies reported data on communication and satisfaction with treatment (27, 28, 52, 53, 58, 67, 71, 81, 82).

Self-management and related outcomes were reported in the following studies: T2D self-management (93), COPD self-management and co-morbidity (59), enablement (81), Patient confidence in managing coronary heart disease and obtaining rheumatology care (25, 53, 54), self-efficacy (25, 28, 64, 65, 69, 75, 93), change from admission to discharge in the number of basic activities of daily living (ADLs) that the patient could perform independently (56), performance in activities (28, 53), patient reported health status and change in health activities (55, 69), and health education impact (75).

The main psychosocial outcomes and concerns reported were psychiatric morbidity (88), psychological disturbance (53, 83), concerns and wellbeing (82), anxiety and depression/mood (28, 57-59, 63, 64, 81, 85), Motor function (73), primary emotions (81). Distress (64, 68, 69, 76, 99), and decisional conflict (68).

Caregiver outcomes assessed were depressive symptoms, caregiver strain, caregiver productivity loss (60), caregiver quality of life (62, 78), and caregiver burden (27, 78). Other caregiver outcomes were informal care that is percentage reported providing assistance with personal ADLs (27), participation in everyday occupations and Life satisfaction (27).

Health care professional outcomes included job strain (72), transition to palliative care, general communication, involvement of significant others (70), GP's knowledge about

For peer review only - http://bmjopen.bmj.com/site/about/guidelines.xhtml

medication taken by the patient (71)), and intention to engage in Interprofessional Shared Decision Making (68).

Data on costs and healthcare utilisation

Six studies reported data on costs of healthcare utilisation (23, 34, 46, 52, 56, 84), and four on number of hospital appointments (55, 81, 90, 100). Two studies reported data on hospital admissions (55, 90), and three studies reported length of hospital stay (56, 65, 100). Seven studies reported data on unplanned readmissions, emergency room attendance (55, 74, 76, 84, 92, 97, 100), and four studies reported healthcare utilisation (55, 71, 75, 97), and medications count (change in number of medications taken by the patient) (71).

Data on clinical outcomes

Clinical outcomes assessed were systolic and diastolic blood pressure (26, 77, 82), fasting blood sugar, HbA1c (26, 63, 77, 80, 93), Body mass index, Haemoglobin (26, 82), Lung function FEV_1/FVC ratio, exercise capacity, (79), total cholesterol to HDL cholesterol ratio (63), serum ferritin, iron level, total iron binding capacity (87), mortality (59, 79, 92, 97).

Synthesis of the findings

We synthesised the findings using methods of narrative synthesis in systematic reviews (101). A narrative synthesis is presented based on the model which informed the intervention, interventions elements/components, mechanism of action, study population, study design (RCT or non-RCT) and outcomes.

Theoretical model/framework used by the study

The majority of the studies (n=34) did not report which theory or model informed the design or delivery of the interventions (54-58, 60-63, 66-68, 70-87, 89-92). One study was informed by the Theory of Hernandez (26), three studies were developed and designed based on

For peer review only

Bandura’s self-efficacy theory (28, 64, 93), and another study used the person-centred palliative care model, Six S: self-image, self-determination, social relationships, symptom control, synthesis and surrender (23, 24). One study reported the Chronic Care Model and person-centred clinical method (69). Person-centred care according to the University of Gothenburg Centre for Person-centred Care” (GPCC) informed most of the studies conducted in Sweden (25, 27, 30, 32-38, 41-51, 71, 96).

Mechanism of action of the interventions.

For the GPCC model which involved three main parameters (initiation of partnership between the patient/caregiver and health professional, implementing the partnership and documenting/safeguarding the partnership). This model was applied across different settings and populations. It also involved both patients and health professionals in developing and designing the intervention and implementation.

Person-centred care requires ongoing systematic engagement between the patient and health professionals. Furthermore, requires to be adapted to each patient population (cancer, HIV, COPD, T2D etc) and context (primary care, outpatient, residential homes, emergency care, hospital, rehabilitation etc). Care plans, goals of care discussed and revised as necessary continuously. Communication is also an important component in the GPCC model. Communication offered by the GPCC model gives patients (for example inpatient setting) information and confidence about care processes and self-management of their own problems and concerns. This leads to understanding of the discharge processes and readiness and eagerness to return home which promotes self-efficacy. For the theory of Hernandez, self-efficacy and all other studies which did not state the theoretical framework, their mechanism of action were similar with the GPCC because they either had a self-management component or self-efficacy and were aimed at empowering the patient or caregiver or improving communication between the patient and the health professional.

For peer review only

Interventions comprising of a self-management component

Fifteen RCTs consisted of a self-management intervention or component. These were conducted in COPD (28, 59, 79), T2D (26, 65, 80, 84, 93), elderly with chronic conditions (55, 64, 75), cancer (41), IBD(81), multimorbidity (60, 69) populations. All the self-management interventions were educational and consisted of training of patients and/or caregivers (28, 41, 55, 60, 64, 75, 79, 84, 93) or both health care professionals and patients/caregivers (26, 60, 80, 81). Educational sessions were either group-based (26, 55, 60, 80, 81, 93) or individualised/face-to-face (59, 79). Four of the thirteen studies examined effects of the intervention on hospital admissions (55, 59, 79, 84). Three studies showed positive benefits of self-management interventions in reducing hospital admissions. One of these four studies assessed mortality (59), another one length of stay in the hospital (79) while one study assessed unplanned visits to the hospital (84). All studies reported positive benefits of the intervention in reducing mortality, length of hospital stay and unplanned visits. Six of the thirteen studies assessed quality of life outcomes (41, 59, 60, 79, 81, 93). In three studies QoL was assessed using the St George's Respiratory Questionnaire (59, 60, 79) and the results were significant. One study used the HRQoL measure and the results were non-significant, but significant on specific problems such as swallowing, social eating and feeding (41). Three studies reported non-significant results and assessed quality of life using the IBD questionnaire (81), the Thai Version short-form Health Survey (93) and the Chronic Respiratory Disease Questionnaire (65) . HADS was used in three studies (28, 59, 81) but only one reported significant findings (59) and two reported non-significant findings (28, 81). Self-efficacy was assessed in six studies (28, 64, 65, 69, 75, 93) with only one study reporting significant results (93). Knowledge on self-management was reported in two studies, T2D (93) and COPD (59) populations, with both studies reporting significant differences between the intervention and control groups (59, 93).

For peer review only

Technology based interventions

Thirteen studies used technology. These were conducted among patients with T2D (63, 68, 77, 93), cancer (41, 67, 76), COPD (35, 79, 84), chronic disease among elderly (55), and IBD (81). Two of these studies were informed by the GPCC model (35, 41) and one was informed by Bandura's model (93). The rest were not informed by a theoretical model. Most of these technology-based intervention studies used a telephone-based intervention (35, 41, 55, 76, 79, 93). One study used a mobile app (67), web-based (68)., four used tablet or computer technology (63, 68, 77, 84), and three used a video (68, 81). The mechanism of action was similar across all these technological based interventions. Patients were communicating using the phone or mobile app or tablet to ask for help if they have problems and concerns and health professionals acted accordingly. This meant patient were involved in taking care of themselves and making decisions.

The outcomes however varied across these studies. Self-efficacy was examined in two studies (35, 93), with different population (COPD (35) and T2D (93)) and they used different measures to assess self-efficacy, both studies reported significant improvement in self-efficacy. Quality of life was examined in five studies (41, 63, 68, 81, 84) and they all used different measures. Only one study reported significant benefits of the intervention (41). Hospitalisations/rehospitalisations, length of stay, unplanned visits were reported in four studies (35, 55, 79, 84). All studies reported positive benefits of technology in reducing hospitalisations, length of stay and unplanned visits. Three of these studies were in COPD population (35, 79, 84), one in T2D population (68). and another one study in the elderly population (55). Two studies reported data on knowledge of management of T2D (77, 93). One study recruited participants with T2D and hypertension (77).

However only one study found that knowledge of T2D management was statistically significant between the intervention and control group. (93)

One study reported data on patient assessment of chronic illness and found statistically significant differences between web-based decision aid intervention and usual care (68).

For peer review only - http://bmjopen.bmj.com/site/about/guidelines.xhtml

Synthesis based on study design

Of the n=55 included studies, n=6 studies (n=10 papers) were non-RCT (31, 32, 46, 48-51, 62, 91, 94). Participants in these studies were elderly people with multi-morbidity (91), total hip replacement (50, 51), cancer patients (32), chronic heart failure (46, 48, 49, 94), patients approaching death and their family caregivers (62), health professionals in nursing homes (31). Length of stay was assessed in heart failure, cancer, and hip replacement studies and was significant all studies (32, 50, 51, 94). Quality of life was assessed in three studies (32, 48, 62), and two studies reported statistically significant differences between two groups (32, 62), among cancer patients (32) and family caregivers of patients approaching death (62).

For RCT design, n=12 studies did not clearly state the methods of randomisation. These were conducted in various populations: IBD (81), T2D (74, 80), breast reconstruction (67), stroke patients and their families (27, 42, 43, 98), multi-morbidity patients and their families (60) heart failure/COPD (23, 24, 90), chronic pain/musculoskeletal pain/fibromyalgia (58, 83).

Quality of life was assessed in seven studies and was statistically significant in three studies (23, 24, 60), but was statistically non-significant in four studies (58, 81, 90, 98). Pain disability, intensity, and interference was assessed in the chronic pain study and showed positive benefits in all outcomes (58), while the MSP/Fibromyalgia assessed pain intensity and number of tender points. Only number of tender points significantly reduced in the intervention compared with the control group (83). Healthcare utilisation was assessed in three studies (67, 74, 90). Emergency and elective admission rates significantly decreased in the intervention compared with the control group in T2D study (74), follow-up hospital visits significantly decreased in breast reconstruction study (67) while hospital admissions were not statistically significant between two groups in COPD population (90). Caregiver outcomes: burden, mood/anxiety (98), depression and strain (60) were not significantly different in both studies.

For peer review only - http://bmjopen.bmj.com/site/about/guidelines.xhtml

Thirty-nine RCTs clearly stated randomisation methods and these recruited participants from patient, family caregivers and health care professionals. The main patient population were COPD (n=6) (28, 35, 59, 65, 79, 89) T2D (n=6) (26, 63, 68, 77, 82, 93), multiple chronic conditions and /or elderly population n=7 (55, 64, 69, 71, 75, 78, 92), arthritis n=4 (30, 36, 52, 61), cancer n=3 (41, 70, 76), acute coronary syndrome n=6 (25, 33, 34, 39, 40, 96), HIV n=2, and Parkinson's disease n=1 (66, 73, 88).

Quality of life, self-efficacy, health utilisation and costs of care were the main outcomes reported. Quality of life was assessed in n=16 studies, with six studies reporting statistically significant results. Quality of life was significant in a study among patients with chronic multiple conditions (75), COPD (59, 79, 89), and HIV (66, 88), but was not significant in T2D population (63, 68, 82, 93), cancer (41), elderly with chronic conditions (71), acute coronary syndrome (33, 34), COPD (65), multimorbidity (69) and patients at end of life (57).

Self-efficacy was assessed in nine studies (25, 28, 33, 35, 54, 65, 69, 75, 93) with only two reporting positive benefits of the intervention (25, 93). Health utilisation was reported in ten studies (35, 55, 56, 59, 66, 71, 75, 76, 79, 92). Rehospitalisations significantly improved in COPD population and chronic multiple conditions (35, 55, 66, 79, 92), mortality also reduced in COPD and chronic multiple conditions (35, 59, 102).

Healthcare use significantly reduced among the elderly with chronic conditions (71), length of hospital stay significantly reduced in one COPD study (79), but was non-significant in another COPD study (65), and among older people (56). Hospital admission/visit to emergency was not significant in COPD and cancer population (59, 76). Health care use was not significant in chronic multiple conditions (75).

Caregiver outcomes

Quality of life among caregivers and caregiver perceived burden significantly improved among family caregivers of older people in a geriatric practice (78). In a guided care intervention quality of chronic Illness care, work productivity loss and absenteeism improved significantly for caregivers (60). However depressive symptoms, and caregiver strain were

For peer review only

not significantly changed (60). In a cluster randomized controlled trial of a client-centred, activities of daily living intervention for caregivers of people with stroke, caregiver burden, life satisfaction, perceived burden, mood, did not differ significantly (27).

Health professional outcomes

A training programme among oncologists resulted in significant changes in the following behavioural domains: transition to palliative care, general communication, and involving significant others (70). A patient-centred communication intervention reported that GP's knowledge about medication taken by the patient was not significant (71). Job strain did not differ significantly between groups even though the intervention reported greater job satisfaction. Similarly modified task and job analysis did not differ significantly, however time pressure did decrease significantly (72). Intention to engage in interprofessional shared decision making did not differ significantly in a Canadian trial (68).

Costs of care and healthcare utilisation

A person-centred integrated intervention and a technology-based intervention for heart failure patients reduced the costs of care in the Swedish and Spanish trials, a nurse-led rheumatology clinic vs rheumatologists-led clinic, and in acute coronary syndrome (23, 34, 46, 52, 84), however costs of services were not different among elderly admitted to a unit with acute illness (56).

Hospital appointments decreased in the PC intervention compared to control in a multicenter cluster intervention for IBD patients (81) likewise in an interdisciplinary collaborative practice intervention hospital visits to see the physician reduced significantly (55). Patients in the individualised care plan intervention called out the ambulance more frequent than those who received usual care (90), even though the intervention group had more GP visits compared with control group (15.6 vs 11.6) in 12 months and the intervention group had more hospital admissions compared with the control group the differences were not statistically significant (90), healthcare utilisation was not significantly different between a clinician-led self-

For peer review only - http://bmjopen.bmj.com/site/about/guidelines.xhtml

management trial and usual care (75). A quasi-experimental design also showed no significant differences on healthcare utilisation, hospitalization, emergency department attendance (62).

In an integrated practice unit and modified virtual ward model in Singapore, unplanned readmissions at 30, 90 and 180 days were significantly lower in the intervention group than the control group (92), emergency department attendance were significantly lower at 30,90 and 180 days in the intervention (92). Likewise an interdisciplinary, collaborative practice intervention involving a primary care physician, a nurse, and a social worker for community-dwelling seniors with chronic illnesses, showed significant changes in number of hospital admissions per patient per year, percentage of patients with 1 or more hospital readmissions within 60 days, and mean number of visits to all physicians (55), fewer attendances at physical, occupational or speech therapy units (71) compared to control group. However, change in percentage of patients with 1 or more visits to the emergency department, change in proportion of patients with 1 or more home care visits, and change in number of patients with 1 or more nursing home placements and emergency visits were not significant (55). Similarly, in a centralised, nurse-delivered telephone-based service to improve care coordination and patient reported outcomes after surgery for colorectal cancer unplanned readmission changes in emergency visits were non-significant (76).

Mortality was significantly reduced in the community-based integrated care for frail COPD patients (59). Mortality was significantly lower in an integrated practice unit and modified virtual ward model (92). A comprehensive care programme with multidisciplinary input for patients with COPD reported reduction in mortality rates compared to usual care (79). However, a team intervention for the multi-morbid elderly reported that mortality risk at 3- and 6-months follow-up were all nonsignificant (97).

A technology-based intervention of a home monitoring via mobile app on the number of in-person visits following ambulatory surgery showed that follow-up visits were significantly

For peer review only - http://bmjopen.bmj.com/site/about/guidelines.xhtml

lower after surgery in the intervention compared to the control group (67), number of phone calls and emails made to the health care in 30 days after surgery were not significant (67). A person-centred communication intervention did not lead to change in number of medications taken by patient (71).

In a Norwegian patient-centred team intervention number of emergency admissions, sum of emergency inpatient bed days, count of emergency re-admissions within 30 days of discharge, count of planned out-patient visits, count of emergency outpatient visits, mortality risk at three- and six-months follow-up were all nonsignificant (97).

Clinical outcomes

Significant improvements were seen among T2D and hypertensive patients in systolic and diastolic blood pressure (77), likewise a patient-centred education programme among newly diagnosed patients with T2D, HbA1c was significant (80). Fasting blood sugar, HbA1c was not statistically different between the two groups (26, 77). In a self-management trial in Sweden among T2D patients, HbA1c was significant (26), but not significant in a Thai trial (93), and computer-based USA trial (63). Furthermore, cholesterol levels were not different in a computer-based trial (63). Blood pressure (both systolic and diastolic) in a T2D trial (26, 82), and haemoglobin were not significant (82). In a T2D UK trial body mass index was significant (82), but was not significant in a Swedish self-management trial for T2D patients (26). An Iranian trial to test the effect of a holistic care programme (HCP) on the reduction of iron overload in patients with beta-thalassaemia major change in serum ferritin at three months (mg/L), change in iron level at three months (micrograms/dL) were significant, but change in serum ferritin 1 year and 2 years post intervention, total iron binding capacity at three months, haemoglobin (Hb) at three months were not significant (87).

Discussion

Our review found a need for data on operationalising PCC in the delivery of care for patients with serious illness. Furthermore, findings show that PCC can be provided across all settings

For peer review only - http://bmjopen.bmj.com/site/about/guidelines.xhtml

(hospitals: in-patient, outpatient, primary care, community settings and residential homes), but majorly in primary care. PCC can be achieved by involving patients, their families and health professionals. PCC can also be provided using various approaches such as self-management interventions and technology-based interventions.

Most of the studies included in the review were conducted in high income countries predominantly in Sweden and USA, and most of the studies using technology were conducted in high income countries. Most participants in these studies had heart failure, T2D, COPD, cancer, and arthritis. The core component of the intervention included workshop training of health professionals on communication skills, training patients and families on self-assessment, identifying their problems and concerns, creating action plans based on the problems, identifying resources to self-management the problems, and evaluating the care. These components are in line with a systematic review of effective elements in a patient-centred and multi-morbidity care (103). The main outcomes reported across most studies were quality of life, healthcare utilisation, and self-efficacy.
Some studies found effectiveness of PCC interventions in improving quality of life, self-efficacy, health utilisation and reducing costs of care. However, some studies reported no significant differences between PCC interventions and usual care on those outcomes.

Most studies which used person-centred self-management approaches and technology demonstrated positive benefits of the interventions in reducing hospital admissions, length of stay and unplanned visits. This finding concurs with a review of self-management interventions in respiratory and cardiovascular illness which reported that self-management support interventions reduces healthcare utilisation without compromising patient health outcomes (104). However self-efficacy outcomes were mostly significant in technology-based interventions, but not significant across most studies which utilised self-management approaches. Studies reported conflicting results on quality-of-life outcomes. Three of the six studies which used self-management approaches reported statistically significant results

For peer review only - http://bmjopen.bmj.com/site/about/guidelines.xhtml

while only one of the six technology-based interventions reported statistically significant findings. It seems that involving a person in decision making enables them to manage their own disease through technology which leads to reduced hospital visits and length of hospital admission. Our results concur with a previous scoping review that reported positive benefits of information and communication technology PCC interventions on five main chronic diseases (diabetes, cardiovascular, chronic respiratory, stroke and cancer) (105).

In terms of synthesis based on study design most non RCT reported significantly improved quality of life and reduced length of hospital stay. For RCT, of the twenty studies that reported data on quality-of-life outcomes, nine of them reported significant results, however some of these studies did not clearly state the method of randomisation. Our findings are in line with a previous review of palliative care interventions for patients with incurable illness which concluded that quality of life outcomes favoured palliative care interventions (106). Most of the RCTs demonstrated positive effects on the interventions in reducing re/hospitalisation, and improving health utilisation, however self-efficacy was non-significant across most RCT's.

Very few studies delivered the intervention to health professionals (n=4) and caregivers (n=3). Quality of life improved and perceived burden significantly reduced in two caregiver studies. Our findings concur with a review of caregiving intervention in cancer population (107, 108).

However psychosocial outcomes remained unchanged in our review. This is contrary to a review of multi-component and psycho-educational interventions designed to support caregivers in their role such as training, education and skill which found positive benefits in reducing depression and burden of caregiving (109). Our data is also at odds with findings among family caregivers in oncology populations which showed improved emotional support (107).

Studies among health professionals showed positive benefits on time pressure and communication skills, but no differences were reported on knowledge and job strain

For peer review only

outcomes. No study reported data on implementation science outcomes among health professionals. The methodological quality of these studies was poor due small sample sizes, unclear randomisation methods and allocation concealment, therefore studies that reported data on caregivers and health professional outcomes are inconclusive.

Only two studies from this review demonstrated that person-centred interventions were effective in reducing pain outcomes, with five studies showing that interventions had no effect on pain and physical symptoms such as fatigue, shortness of breath in COPD and heart disease populations. However, a previous review on self-initiated interventions among cancer patients with peripheral neuropathy showed that strategies were beneficial in reducing symptoms and concerns (110).

Patient communication and satisfaction with PCC interventions was significant in three of the six studies that reported data on this outcome. Our findings agree with a systematic review on effectiveness of communication-related quality improvement interventions for patients with advanced and serious illness which reported significant improvements on patients' satisfaction with care (106, 111).

This review has shown that PCC interventions reduced costs of care in heart failure, COPD, acute coronary syndrome, and rheumatology populations. This is in line with a meta-analysis on the economics of palliative care for adults with serious illness admitted to a facility that reported lower costs of palliative care consultations than usual care (112). Previous studies have reported that integrated palliative care (breathless support service) reduces costs in cancer patients and their families (113). However the same intervention resulted in extra mean costs of £799 in non-malignant conditions and their families (114), therefore we can attribute the differences due to diagnosis or type of serious illness.

In our review, of the six studies that reported data on costs, five reported that PCC resulted in reduction of costs of care (23, 34, 46, 52, 84). All these studies were conducted in primary care or home setting and two of these recruited both patients and family members as study

For peer review only - http://bmjopen.bmj.com/site/about/guidelines.xhtml

participants (23, 84). The disease conditions were CHF(23, 46, 84), acute coronary syndrome (34) and rheumatoid arthritis (52). The majority of these studies were conducted in Sweden informed by the GPCC model of care (23, 34, 46, 52), while one was conducted in Spain (84).

The intervention comprised of routines for establishment of a partnership between patients, and/or families and healthcare professionals (who received training on how to provide person-centred care, developing a health plan with the patients and/or families. The health plan also contained agreed goals (23, 34, 46, 52, 84), These interventions were integrated in primary care. In person-centred care interventions informed by GPCC, healthcare professionals acquire knowledge and skills to practice PCC. Presumably this reduces hospital attendance thereby saving time and costs travelling to the health facility. However, these are not clearly stated in the studies so we can only speculate. The only study which reported nonsignificant differences between the intervention and control on costs of care was among elderly people admitted to a hospital unit with acute illness(56). This study differs from the other studies in terms of setting, and it has a heterogenous group of patients with CHF, cancer, dementia, chronic lung disease, cardiovascular disease and it is not clear which model informed the intervention.

Some studies included in this review showed significant improvements in both clinical, and psychosocial outcomes, while some showed no improvements in either of them. For example among beta-thalassaemia major patients, significant results were reported on clinical outcomes such as serum ferritin (mg/L) and iron levels (micrograms/dL) including change in physical activity: six-minute walk test (6MWT) (87), a technology-based trial of a person-centred tablet computer-based self-monitoring system for chronic disease (T2D and/or hypertension)(77) reported significant improvement on systolic and diastolic blood pressure but did not show significant differences on fasting blood sugar levels and patient's knowledge of T2D and hypertension. In HIV population a Kenyan trial showed no differences between groups on the primary outcome of pain, but showed significant differences between

For peer review only

groups on psychiatric morbidity and quality of life (88) and another study showed no significant differences on both clinical and psychosocial outcomes in T2D population (63).

Strengthens and limitations

It is interesting to note that the majority of the studies n=31 studies achieved relative complete follow-up, that is at least 80% of the participants were followed-up at trial end points. This is encouraging considering that is it challenging to follow-up participants with serious illnesses. We used robust procedures for systematic reviewing and quality assessment of the studies included, in line with Preferred Reporting Items for Systematic Reviews and Meta-Analyses (PRISMA) reporting guidelines, however we did not use a checklist for health economic outcome studies. We only used the critical appraisal checklist for randomised controlled studies. Furthermore, we did not GRADE (Grading of Recommendations, Assessment, Development and Evaluations) the quality of evidence for each outcome (115). Most of the studies included did not state the theoretical framework underpinning the person-centred interventions. However, many studies that reported the theoretical framework used the GPCC and were conducted in Sweden across various clinical settings. Most of the studies identified and included were conducted in HIC.

Meta-analysis was not possible in this review due to heterogenicity of studies. Studies were from different patient populations, different trial designs (parallel trials or clustered trials), different sample sizes, different interventions and dimensions, different outcomes and measures used, different follow-up periods and intervals, and interventions delivered in different settings. Some interventions targeted health care professionals and outcomes assessed among patients and health care professionals. Some interventions targeted patients and family dyads and captured data from both patients and their families, while some interventions targeted patients only, and family caregivers only.

Furthermore, interventions were delivered or led by different groups of professionals such as nurses, physiotherapists, physicians, social workers.

For peer review only - http://bmjopen.bmj.com/site/about/guidelines.xhtml

Due to nature of the interventions, it was difficult to blind study participants and those delivering the intervention, however three studies blinded study participants and two studies blinded those who delivered the intervention. It is challenging to design double-blinded or triple-blinded complex person-centred interventions. However, it is possible to blind outcome assessors. In this review n=21 studies blinded outcomes assessors and two studies used postal questionnaires or web-based survey.

Some studies clearly stated the PCC model which informed the intervention while some studies did not state the PCC model. We still included studies that did not state the PCC model after critically reading through the text to understand important concepts and elements of PCC such as holistic care, coordinated physical health and supportive services, person-focused care, multidisciplinary team approach, involvement of patient and family and emphasise on person and family outcomes, respectful care and responsive to individual patient preferences, needs, and values to guide all clinical decisions (116, 117). It is possible that through this process, we might have missed some papers.

Conclusions, implications for policy, practice, and research

There is some evidence that PCC interventions using self-management have some effects in reducing health utilisation, costs of care, and improving quality of life.

Technology based interventions also reduces healthcare utilisation and improves self-efficacy but appears to have less effect on quality of life. However very few studies used self-management and technology approaches. Further work is needed to identify how self-management and technology PCC approaches can be used in serious illness across different disease conditions and settings. The majority of studies clearly defined what constituted usual care or the comparator. This shows that it is possible to design and deliver a person-centred care intervention in different care settings where this is currently not being practiced.

For peer review only - http://bmjopen.bmj.com/site/about/guidelines.xhtml

PCC can be designed and evaluated using robust study designs, and can be delivered in primary, secondary and tertiary care including home settings and residential homes. Institutions should therefore consider implementing person-centred care interventions using locally available resources.

PCC interventions can target patients, their families or health professionals. PCC research has mainly focused in HIC, more research needs to be done in LMIC. Further work to consider designing and evaluating PCC interventions at community level targeting community health workers, and family members. Few studies (6/55) examined costs of person-centred care interventions. Health service researchers should consider incorporating costs of PCC or health economic outcomes when designing and evaluating complex PCC interventions.

For peer review only

Contributors

KN planned, conducted searches, and submitted the manuscript. KN and AC extracted data. KN and AC assessed quality of the included studies and compared assessments. RH reviewed data extraction and quality appraisal. AG, RP, IP, LF, LG, and SV contributed to design and interpretation. All authors approved the manuscript.

Funding

This work is funded by the National Institute of Health Research (NIHR) Global Health Research Unit on Health System Strengthening in sub-Saharan Africa, King's College London (GHRU 16/136/54) using UK aid from the UK Government to support global health research.

Data availability statement

Data available upon reasonable request.

Ethics statement

Patient consent for publication: Not required.

Ethics approval

No ethical approval was sought for this work. This study is a systematic review that analysed anonymised data from published studies, which already obtained informed consent/ethical approval.

Conflict of interest

No conflict of interest to declare

Figure 1: Logic Model for interventions with a theoretical model

Figure 2: Logic model for Interventions without a theoretic model

Figure 3: PRISMA Flow Diagram

For peer review only

References

1. WHO. Person-Centred Health Care: A policy framework Geneva: WHO; 2007 [
2. Gillian Le RM, Janine Bestall, Tim Ensor, Imogen Featherstone, Thomas Veale. The Impact of Universal Health Coverage, People Centred Care and Integrated Service Delivery on Key Health System Outcomes 2014 Leeds University of Leeds 2014 [
3. WHO. Universal Health Coverage: Moving Towards Better Health

Action Framework for the Western Pacific Region. WHO; 2016.
4. Kitson A, Marshall A, Bassett K, Zeitz K. What are the core elements of patient-centred care? A narrative review and synthesis of the literature from health policy, medicine and nursing. J Adv Nurs. 2013;69(1):4-15.
5. 2007 IhiousdffIP-CHRnepP.
6. WHO. WHO global strategy on people-centred and integrated health services2015.
7. Wilberforce M, Challis D, Davies L, Kelly MP, Roberts C, Loynes N. Person-centredness in the care of older adults: a systematic review of questionnaire-based scales and their measurement properties. BMC Geriatrics. 2016;16(1):63.
8. Giusti A, Nkhoma K, Petrus R, Petersen I, Gwyther L, Farrant L, et al. The empirical evidence underpinning the concept and practice of person-centred care for serious illness: a systematic review. BMJ Glob Health. 2020;5(12).
9. Kelley AS, Covinsky KE, Gorges RJ, McKendrick K, Bollens-Lund E, Morrison RS, et al. Identifying Older Adults with Serious Illness: A Critical Step toward Improving the Value of Health Care. Health Serv Res. 2016.
10. WHO. People-centred care in low and middle income countries2010.
11. Sleeman KE, de Brito M, Etkind S, Nkhoma K, Guo P, Higginson IJ, et al. The escalating global burden of serious health-related suffering: projections to 2060 by world regions, age groups, and health conditions. Lancet Glob Health. 2019;7(7):e883-e92.
12. Harding R, Leam C. Clinical notes for informal carers in palliative care: recommendations from a random patient file audit. Palliative medicine. 2005;19(8):639-42.
13. Moens K, Higginson IJ, Harding R, Brearley S, Caraceni A, Cohen J, et al. Are There Differences in the Prevalence of Palliative Care-Related Problems in People Living With Advanced Cancer and Eight Non-Cancer Conditions? A Systematic Review. Journal of pain and symptom management. 2014;48(4):660-77.
14. Streid J, Harding R, Agupio G, Dinat N, Downing J, Gwyther L, et al. Stressors and resources of caregivers of patients with incurable progressive illness in sub-Saharan Africa. Qualitative health research. 2014;24(3):317-28.
15. Kelley AS. Defining "serious illness". J Palliat Med. 2014;17(9):985.
16. Little P, Everitt H, Williamson I, Warner G, Moore M, Gould C, et al. Observational study of effect of patient centredness and positive approach on outcomes of general practice consultations. Bmj. 2001;323(7318):908-11.
17. Nkhoma K, Giusti A, Petrus R, Petersen I, Gwyther L, Harding R. A systematic review investigating the effectiveness of person-centred interventions in serious physical illness. https://www.crd.york.ac.uk/prospero/#recordDetails. 2018.
18. Moher D, Liberati A, Tetzlaff J, Altman DG. Preferred Reporting Items for Systematic Reviews and Meta-Analyses: The PRISMA Statement. Journal of Clinical Epidemiology. 2009;62(10):1006-12.
19. Higgins J, Green S. Cochrane handbook for systematic reviews of interventions 4.2. 5 [updated May 2005]. The cochrane library. 2005(3).
20. Centre for Review and Dissemination. Systematic Reviews: CRD's Guidance for Undertaking Reviews in Health Care2009. Available from: http://www.york.ac.uk/inst/crd/pdf/Systematic_Reviews.pdf.
21. Randomised JBICACf, https://jbi.global/critical-appraisal-tools CTAa, docs/critical-appraisal-tools/JBI_Critical_Appraisal-Checklist_for_, 2020 RCTpAJ. Critical Appraisal Checklist for Randomised

For peer review only - http://bmjopen.bmj.com/site/about/guidelines.xhtml

Controlled Trials. 2016.
22. Popay J, Roberts, H. M., Sowden, A., Petticrew, M., Arai, L., Rodgers, M., & Britten, N. (2006). Guidance on the conduct of narrative synthesis in sytematic reviews. Institute for Health Research. https://www.google.com/url?sa=t&rct=j&q=&esrc=s&source=web&cd=1&cad=rja&uact=8&ved=2ahUKEwiK6fuyk9fgAhVDtnEKHV9-AUgQFjAAegQIBxAC&url=http%3A%2F%2Fciteseerx.ist.psu.edu%2Fviewdoc%2Fdownload%3Fdoi%3D10.1.1.178.3100%26rep%3Drep1%26type%3Dpdf&usg=AOvVaw3xMoGRunApJPo0_YYk1hqo. Guidance on the conduct of narrative synthesis in sytematic reviews. 2006.
23. Sahlen K-G, Boman K, Brannstrom M. A cost-effectiveness study of person-centered integrated heart failure and palliative home care: Based on a randomized controlled trial. Palliative Medicine. 2016;30(3):296-302.
24. Brannstrom M, Boman K. Effects of person-centred and integrated chronic heart failure and palliative home care. PREFER: a randomized controlled study. European Journal of Heart Failure. 2014;16(10):1142-51.
25. Fors A, Swedberg K, Ulin K, Wolf A, Ekman I. Effects of person-centred care after an event of acute coronary syndrome: Two-year follow-up of a randomised controlled trial. International Journal of Cardiology. 2017;249:42-7.
26. Jutterstrom L, Hornsten A, Sandstrom H, Stenlund H, Isaksson U. Nurse-led patient-centered self-management support improves HbA1c in patients with type 2 diabetes-A randomized study. Patient Education and Counseling. 2016;99(11):1821-9.
27. Bertilsson AS, Eriksson G, Ekstam L, Tham K, Andersson M, von Koch L, et al. A cluster randomized controlled trial of a client-centred, activities of daily living intervention for people with stroke: one year follow-up of caregivers. Clinical rehabilitation. 2016;30(8):765-75.
28. Zakrisson AB, Arne M, Hasselgren M, Lisspers K, Ställberg B, Theander K. A complex intervention of self-management for patients with COPD or CHF in primary care improved performance and satisfaction with regard to own selected activities; A longitudinal follow-up. J Adv Nurs. 2019;75(1):175-86.
29. Larsson I, Bergman S, Bremander A. Person-centred care (PCC) may improve health care consumer skills more than regular care-an RCT in patients with cia undergoing biological therapy. Annals of the Rheumatic Diseases. 2015;2):104.
30. Bergsten U, Almehed K, Baigi A, Jacobsson LTH. A randomized study comparing regular care with a nurse-led clinic based on tight disease activity control and person-centred care in patients with rheumatoid arthritis with moderate/high disease activity: A 6-month evaluation. Musculoskeletal Care. 2019;17(3):215-25.
31. Bökberg C, Behm L, Wallerstedt B, Ahlström G. Evaluation of person-centeredness in nursing homes after a palliative care intervention: pre- and post-test experimental design. BMC Palliative Care. 2019;18(1):44.
32. Öhlén J, Sawatzky R, Pettersson M, Sarenmalm EK, Larsdotter C, Smith F, et al. Preparedness for colorectal cancer surgery and recovery through a person-centred information and communication intervention - A quasi-experimental longitudinal design. PLoS One. 2019;14(12):e0225816.
33. Pirhonen L, Olofsson EH, Fors A, Ekman I, Bolin K. Effects of person-centred care on health outcomes-A randomized controlled trial in patients with acute coronary syndrome. Health Policy. 2017;121(2):169-79.
34. Pirhonen L, Bolin K, Olofsson EH, Fors A, Ekman I, Swedberg K, et al. Person-Centred Care in Patients with Acute Coronary Syndrome: Cost-Effectiveness Analysis Alongside a Randomised Controlled Trial. PharmacoEconomics - Open. 2019;3(4):495-504.
35. Fors A, Blanck E, Ali L, Swedberg K, Ekman I. Person-centred telephone-support is effective in patients with chronic obstructive pulmonary disease and/or chronic heart failure-six-month follow-up of a randomized controlled trial. European Journal of Heart Failure. 2018;20 (Supplement 1):194.

For peer review only

36. Feldthusen C, Dean E, Forsblad-d'Elia H, Mannerkorpi K. Effects of Person-Centered Physical Therapy on Fatigue-Related Variables in Persons With Rheumatoid Arthritis: A Randomized Controlled Trial. Arch Phys Med Rehabil. 2016;97(1):26-36.
37. Fors A, Taft C, Ulin K, Ekman I. Person-centred care improves self-efficacy to control symptoms after acute coronary syndrome: A randomized controlled trial. European Journal of Cardiovascular Nursing. 2016;15(2):186-94.
38. Fors A, Gyllensten H, Swedberg K, Ekman I. Effectiveness of person-centred care after acute coronary syndrome in relation to educational level: Subgroup analysis of a two-armed randomised controlled trial. Int J Cardiol. 2016;221:957-62.
39. Fors A, Ekman I, Taft C, Björkelund C, Frid K, Larsson ME, et al. Person-centred care after acute coronary syndrome, from hospital to primary care - A randomised controlled trial. Int J Cardiol. 2015;187:693-9.
40. Wolf A, Fors A, Ulin K, Thorn J, Swedberg K, Ekman I. An eHealth Diary and Symptom-Tracking Tool Combined With Person-Centered Care for Improving Self-Efficacy After a Diagnosis of Acute Coronary Syndrome: A Substudy of a Randomized Controlled Trial. J Med Internet Res. 2016;18(2):e40.
41. Hansson E, Carlström E, Olsson LE, Nyman J, Koinberg I. Can a person-centred-care intervention improve health-related quality of life in patients with head and neck cancer? A randomized, controlled study. BMC Nurs. 2017;16:9.
42. Bertilsson A-S, Ranner M, von Koch L, Eriksson G, Johansson U, Ytterberg C, et al. A client-centred ADL intervention: three-month follow-up of a randomized controlled trial. Scandinavian journal of occupational therapy. 2014;21(5):377-91.
43. Guidetti S, Ranner M, Tham K, Andersson M, Ytterberg C, von Koch L. A “Client-Centred Activities of Daily Living” Intervention for Persons with Stroke: One-Year Follow-up of a Randomized Controlled Trial. Journal of Rehabilitation Medicine. 2015;47(7):605-11.
44. Larsson A, Palstam A, Löfgren M, Ernberg M, Bjersing J, Bileviciute-Ljungar I, et al. Resistance exercise improves muscle strength, health status and pain intensity in fibromyalgia--a randomized controlled trial. Arthritis Res Ther. 2015;17(1):161.
45. Ericsson A, Palstam A, Larsson A, Löfgren M, Bileviciute-Ljungar I, Bjersing J, et al. Resistance exercise improves physical fatigue in women with fibromyalgia: a randomized controlled trial. Arthritis Res Ther. 2016;18:176.
46. Hansson E, Ekman I, Swedberg K, Wolf A, Dudas K, Ehlers L, et al. Person-centred care for patients with chronic heart failure - a cost-utility analysis. Eur J Cardiovasc Nurs. 2016;15(4):276-84.
47. Ulin K, Olsson LE, Wolf A, Ekman I. Person-centred care - An approach that improves the discharge process. European Journal of Cardiovascular Nursing. 2016;15(3):e19-26.
48. Ekman I, Wolf A, Olsson LE, Taft C, Dudas K, Schaufelberger M, et al. Effects of person-centred care in patients with chronic heart failure: the PCC-HF study. European Heart Journal. 2012;33(9):1112-9.
49. Dudas K, Olsson LE, Wolf A, Swedberg K, Taft C, Schaufelberger M, et al. Uncertainty in illness among patients with chronic heart failure is less in person-centred care than in usual care. European Journal of Cardiovascular Nursing. 2013;12(6):521-8.
50. Olsson L-E, Karlsson J, Berg U, Kärrholm J, Hansson E. Person-centred care compared with standardized care for patients undergoing total hip arthroplasty—a quasi-experimental study. Journal of Orthopaedic Surgery and Research. 2014;9(1):95.
51. Olsson LE, Hansson E, Ekman I. Evaluation of person-centred care after hip replacement-a controlled before and after study on the effects of fear of movement and self-efficacy compared to standard care. BMC Nurs. 2016;15(1):53.
52. Larsson I, Fridlund B, Arvidsson B, Teleman A, Svedberg P, Bergman S. A nurse-led rheumatology clinic versus rheumatologist-led clinic in monitoring of patients with chronic inflammatory arthritis undergoing biological therapy: A cost comparison study in a randomised controlled trial. BMC Musculoskeletal Disorders. 2015;16 (1) (no pagination)(817).

For peer review only

53. Larsson I, Fridlund B, Arvidsson B, Teleman A, Bergman S. Treatment outcomes from a nurse-led rheumatology clinic in monitoring of anti-TNF therapy-a randomised controlled trial. Arthritis and Rheumatism. 2012;10):S667.
54. Kelechi TJ, Mueller M, Spencer C, Rinard B, Loftis G. The effect of a nurse-directed intervention to reduce pain and improve behavioral and physical outcomes in patients with critically colonized/infected chronic leg ulcers. Journal of Wound, Ostomy, & Continence Nursing. 2014;41(2):111-21.
55. Sommers LS, Marton KI, Barbaccia JC, Randolph J. Physician, nurse, and social worker collaboration in primary care for chronically ill seniors. Archives of Internal Medicine. 2000;160(12):1825-33.
56. Landefeld CS, Palmer RM, Kresevic DM, Fortinsky RH, Kowal J. A randomized trial of care in a hospital medical unit especially designed to improve the functional outcomes of acutely ill older patients. New England Journal of Medicine. 1995;332(20):1338-44.
57. Chochinov HM, Kristjanson LJ, Breitbart W, McClement S, Hack TF, Hassard T, et al. Effect of dignity therapy on distress and end-of-life experience in terminally ill patients: a randomised controlled trial. Lancet Oncology. 2011;12(8):753-62.
58. Dobscha SK, Corson K, Perrin NA, Hanson GC, Leibowitz RQ, Doak MN, et al. Collaborative care for chronic pain in primary care: a cluster randomized trial. JAMA. 2009;301(12):1242-52.
59. Hernandez C, Alonso A, Garcia-Aymerich J, Serra I, Marti D, Rodriguez-Roisin R, et al. Effectiveness of community-based integrated care in frail COPD patients: A randomised controlled trial. npj Primary Care Respiratory Medicine. 2015;25 (no pagination)(15022).
60. Wolff JL, Giovannetti ER, Boyd CM, Reider L, Palmer S, Scharfstein D, et al. Effects of guided care on family caregivers. The Gerontologist. 2010;50(4):459-70.
61. Murphy SL, Lyden AK, Smith DM, Dong Q, Koliba JF. Effects of a tailored activity pacing intervention on pain and fatigue for adults with osteoarthritis. American Journal of Occupational Therapy. 2010;64(6):869-76.
62. Britt HR, JaKa, M. M., Fernstrom, K. M., Bingham, P. E., Betzner, A. E., Taghon, J. R., Shippee, N. D., Shippee, T. P., Schellinger, S. E., & Anderson, E. W. . Quasi-Experimental Evaluation of LifeCourse on Utilization and Patient and Caregiver Quality of Life and Experience. . The American journal of hospice & palliative care 2019;36(5):408-16.
63. Glasgow RE, Nutting PA, King DK, Nelson CC, Cutter G, Gaglio B, et al. Randomized effectiveness trial of a computer-assisted intervention to improve diabetes care. Diabetes Care. 2005;28(1):33-9.
64. Mielenz TJ, Tracy M, Jia H, Durbin LL, Allegrante JP, Arniella G, et al. Creation of the Person-Centered Wellness Home in Older Adults. Innovation in Aging. 2020;4(1).
65. Thom DH, Willard-Grace R, Tsao S, Hessler D, Huang B, DeVore D, et al. Randomized Controlled Trial of Health Coaching for Vulnerable Patients with Chronic Obstructive Pulmonary Disease. Annals of the American Thoracic Society. 2018;15(10):1159-68.
66. Gustafson DH, Hawkins RP, Boberg EW, Bricker E, Pingree S, Chan CL. The use and impact of a computer-based support system for people living with AIDS and HIV infection. Proc Annu Symp Comput Appl Med Care. 1994:604-8.
67. Armstrong KA, Coyte PC, Brown M, Beber B, Semple JL. Effect of home monitoring via mobile app on the number of in-person visits following ambulatory surgery a randomized clinical trial. JAMA Surgery. 2017;152(7):622-7.
68. Yu C, Choi D, Bruno BA, Thorpe KE, Straus SE, Cantarutti P, et al. Impact of MyDiabetesPlan, a Web-Based Patient Decision Aid on Decisional Conflict, Diabetes Distress, Quality of Life, and Chronic Illness Care in Patients With Diabetes: Cluster Randomized Controlled Trial. J Med Internet Res. 2020;22(9):e16984.
69. Fortin M, Stewart M, Ngangue P, Almirall J, Bélanger M, Brown JB, et al. Scaling Up Patient-Centered Interdisciplinary Care for Multimorbidity: A Pragmatic Mixed-Methods Randomized Controlled Trial. Annals of Family Medicine. 2021;19(2):126-34.

For peer review only

70. Goelz T, Wuensch A, Stubenrauch S, Ihorst G, de Figueiredo M, Bertz H, et al. Specific training program improves oncologists' palliative care communication skills in a randomized controlled trial. Journal of Clinical Oncology. 2011;29(25):3402-7.
71. Schafer I, Kaduszkiewicz H, Mellert C, Loffler C, Mortsiefer A, Ernst A, et al. Narrative medicine-based intervention in primary care to reduce polypharmacy: results from the cluster-randomised controlled trial MultiCare AGENDA. BMJ Open. 2018;8(1):e017653.
72. Berendonk C, Kaspar R, Bär M, Hoben M. Improving Quality of Work life for Care Providers by Fostering the Emotional well-being of Persons with Dementia: A Cluster-randomized Trial of a Nursing Intervention in German long-term Care Settings Dementia 2019;18(4):1286-309.
73. Eggers C, Dano R, Schill J, Fink GR, Hellmich M, Timmermann L. Patient-centered integrated healthcare improves quality of life in Parkinson's disease patients: a randomized controlled trial. J Neurol. 2018;265(4):764-73.
74. Mills PD, Harvey PW. Beyond community-based diabetes management and the COAG coordinated care trial. Australian Journal of Rural Health. 2003;11(3):131-7.
75. Reed RL, Roeger L, Howard S, Oliver-Baxter JM, Battersby MW, Bond M, et al. A self-management support program for older Australians with multiple chronic conditions: A randomised controlled trial. Medical Journal of Australia. 2018;208(2):69-74.
76. Young JM, Butow PN, Walsh J, Durcinoska I, Dobbins TA, Rodwell L, et al. Multicenter randomized trial of centralized nurse-led telephone-based care coordination to improve outcomes after surgical resection for colorectal cancer: the CONNECT intervention. Journal of Clinical Oncology. 2013;31(28):3585-91.
77. Or C, Tao D. A 3-Month Randomized Controlled Pilot Trial of a Patient-Centered, Computer-Based Self-Monitoring System for the Care of Type 2 Diabetes Mellitus and Hypertension. Journal of Medical Systems. 2016;40(4):81.
78. Yu DSF. Effects of a Health and Social Collaborative Case Management Model on Health Outcomes of Family Caregivers of Frail Older Adults: Preliminary Data from a Pilot Randomized Controlled Trial. Journal of the American Geriatrics Society. 2016;64(10):2144-8.
79. Ko FWS, Cheung NK, Rainer TH, Lum C, Wong I, Hui DSC. Comprehensive care programme for patients with chronic obstructive pulmonary disease: A randomised controlled trial. Thorax. 2017;72(2):122-8.
80. Windrum P, Garcia-Goni M, Coad H. The Impact of Patient-Centered versus Didactic Education Programs in Chronic Patients by Severity: The Case of Type 2 Diabetes Mellitus. Value in Health. 2016;19(4):353-62.
81. Kennedy A, Nelson E, Reeves D, Richardson G, Roberts C, Robinson A, et al. A randomised controlled trial to assess the impact of a package comprising a patient-orientated, evidence-based self-help guidebook and patient-centred consultations on disease management and satisfaction in inflammatory bowel disease. Health Technology Assessment. 2003;7(28).
82. Kinmonth AL, Woodcock A, Griffin S, Spiegal N, Campbell MJ. Randomised controlled trial of patient centred care of diabetes in general practice: Impact on current wellbeing and future disease risk. British Medical Journal. 1998;317(7167):1202-8.
83. Alamo MM, Moral RR, Perula de Torres LA. Evaluation of a patient-centred approach in generalized musculoskeletal chronic pain/fibromyalgia patients in primary care. Patient Education & Counseling. 2002;48(1):23-31.
84. de Batlle J, Massip M, Vargiu E, Nadal N, Fuentes A, Ortega Bravo M, et al. Implementing Mobile Health-Enabled Integrated Care for Complex Chronic Patients: Intervention Effectiveness and Cost-Effectiveness Study. JMIR Mhealth Uhealth. 2021;9(1):e22135.
85. Machado LA, Azevedo DC, Capanema MB, Neto TN, Cerceau DM. Client-Centered Therapy vs Exercise Therapy for Chronic Low Back Pain: A Pilot Randomized Controlled Trial in Brazil. Pain Medicine. 2007;8(3):251-8.

For peer review only - http://bmjopen.bmj.com/site/about/guidelines.xhtml

86. Kikkenborg Berg S, Stoier L, Moons P, Zwisler AD, Winkel P, Ulrich Pedersen P. Emotions and health: findings from a randomized clinical trial on psychoeducational nursing to patients with implantable cardioverter defibrillator. The Journal of cardiovascular nursing. 2015;30(3):197-204.
87. Arian M, Memarian R, Oghazian MB, Vakilian F, Badiee Z. The effect of a holistic care program on the reduction of iron over load in patients with beta-thalassemia major: A randomized clinical trial. Iranian Red Crescent Medical Journal. 2018;20 (4) (no pagination)(e60820).
88. Lowther K, Selman L, Simms V, Gikaara N, Ahmed A, Ali Z, et al. Nurse-led palliative care for HIV-positive patients taking antiretroviral therapy in Kenya: a randomised controlled trial. Lancet HIV. 2015;2(8):e328-34.
89. Slok AH, Kotz D, van Breukelen G, Chavannes NH, Rutten-van Molken MP, Kerstjens HA, et al. Effectiveness of the Assessment of Burden of COPD (ABC) tool on health-related quality of life in patients with COPD: a cluster randomised controlled trial in primary and hospital care. BMJ Open. 2016;6(7):e011519.
90. Martin IR, McNamara D, Sutherland FR, Tilyard MW, Taylor DR. Care plans for acutely deteriorating COPD: a randomized controlled trial. Chronic respiratory disease. 2004;1(4):191-5.
91. Berntsen GKR, Dalbakk M, Hurley JS, Bergmo T, Solbakken B, Spansvoll L, et al. Person-centred, integrated and pro-active care for multi-morbid elderly with advanced care needs: a propensity score-matched controlled trial. BMC health services research. 2019;19(1):682-.
92. Low LL, Tan SY, Ng MJM, Tay WY, Ng LB, Balasubramaniam K, et al. Applying the integrated practice unit concept to a modified virtual ward model of care for patients at highest risk of readmission: A randomized controlled trial. PLoS ONE. 2017;12 (1) (no pagination)(e0168757).
93. Wichit N, Mnatzaganian G, Courtney M, Schulz P, Johnson M. Randomized controlled trial of a family-oriented self-management program to improve self-efficacy, glycemic control and quality of life among Thai individuals with Type 2 diabetes. Diabetes Research and Clinical Practice. 2017;123:37-48.
94. Ulin K, Olsson LE, Wolf A, Ekman I. Person-centred care - An approach that improves the discharge process. European journal of cardiovascular nursing : journal of the Working Group on Cardiovascular Nursing of the European Society of Cardiology. 2016;15(3):e19-e26.
95. Dambha-Miller H, Cooper AJM, Simmons RK, Kinmonth AL, Griffin SJ. Patient-centred care, health behaviours and cardiovascular risk factor levels in people with recently diagnosed type 2 diabetes: 5-year follow-up of the ADDITION-Plus trial cohort. BMJ Open. 2016;6 (1) (no pagination)(e008931).
96. Fors A, Ekman I, Ulin K, Wolf A, Swedberg K. Person-centred care is effective after an event of acute coronary syndrome; particularly in patients with low educational level-two-year follow-up of a randomised controlled trial. European Heart Journal. 2017;38 (Supplement 1):116.
97. Berntsen G, Hoyem A, Lettrem I, Ruland C, Rumpsfeld M, Gammon D. A person-centered integrated care quality framework, based on a qualitative study of patients' evaluation of care in light of chronic care ideals. BMC Health Serv Res. 2018;18(1):479.
98. Hedman A, Eriksson G, von Koch L, Guidetti S. Five-year follow-up of a cluster-randomized controlled trial of a client-centred activities of daily living intervention for people with stroke. Clin Rehabil. 2019;33(2):262-76.
99. Yu C, Choi D, Bruno BA, Thorpe KE, Straus SE, Cantarutti P, et al. Impact of MyDiabetesPlan, a Web-Based Patient Decision Aid on Decisional Conflict, Diabetes Distress, Quality of Life, and Chronic Illness Care in Patients With Diabetes: Cluster Randomized Controlled Trial. Journal of Medical Internet Research. 2020;22(9):N.PAG-N.PAG.
100. Bergmo TS, Berntsen GK, Dalbakk M, Rumpsfeld M. The effectiveness and cost effectiveness of the PAtient-Centred Team (PACT) model: study protocol of a prospective matched control before-and-after study. BMC Geriatrics. 2015;15:133.
101. Popay J, Roberts, H. M., Sowden, A., Petticrew, M., Arai, L., Rodgers, M., & Britten, N. (2006). Guidance on the conduct of narrative synthesis in sytematic reviews. Institute for Health Research. https://www.google.com/url?sa=t&rct=j&q=&esrc=s&source=web&cd=1&cad=rja&uact=8&ved=2ah

For peer review only - http://bmjopen.bmj.com/site/about/guidelines.xhtml

UKEwiK6fuyk9fgAhVDtnEKHV9-AUgQFjAAegQIBxAC&url=http%3A%2F%2Fciteseerx.ist.psu.edu%2Fviewdoc%2Fdownload%3Fdoi%3D10.1.1.178.3100%26rep%3Drep1%26type%3Dpdf&usg=AOvVaw3xMoGRunApJPo0_YYk1hqo.
102. Low LL, Tan SY, Ng MJM, Tay WY, Ng LB, Balasubramaniam K, et al. Applying the Integrated Practice Unit Concept to a Modified Virtual Ward Model of Care for Patients at Highest Risk of Readmission: A Randomized Controlled Trial. PloS one. 2017;12(1):e0168757-e.
103. Poitras M-E, Maltais M-E, Bestard-Denommé L, Stewart M, Fortin M. What are the effective elements in patient-centered and multimorbidity care? A scoping review. BMC Health Services Research. 2018;18(1):446.
104. Panagioti M, Richardson G, Small N, Murray E, Rogers A, Kennedy A, et al. Self-management support interventions to reduce health care utilisation without compromising outcomes: a systematic review and meta-analysis. BMC Health Services Research. 2014;14(1):356.
105. Wildevuur SE, Simonse LW. Information and communication technology-enabled person-centered care for the "big five" chronic conditions: scoping review. J Med Internet Res. 2015;17(3):e77.
106. El-Jawahri AR, Greer JA, Temel JS. Does palliative care improve outcomes for patients with incurable illness? A review of the evidence. The journal of supportive oncology. 2011;9(3):87-94.
107. Ferrell B, Wittenberg E. A review of family caregiving intervention trials in oncology. . CA Cancer J Clin. 2017;67(4): 318-25.
108. Northouse LL, Katapodi MC, Song L, Zhang, L., , Mood DWC. Interventions with family caregivers of cancer patients: meta-analysis of randomized trials. a cancer journal for clinicians. 2010;60(5):317-39.
109. Parker D, Mills S, J. A. Effectiveness of interventions that assist caregivers to support people with dementia living in the community: a systematic review. Int J Evid Based Healthc. 2008;6(2).
110. Ogle T, Alexander K, Miaskowski C, Yates P. Systematic review of the effectiveness of self-initiated interventions to decrease pain and sensory disturbances associated with peripheral neuropathy. Journal of Cancer Survivorship. 2020;14(4):444-63.
111. Fawole OA, Dy SM, Wilson RF, Lau BD, Martinez KA, Apostol CC, et al. A systematic review of communication quality improvement interventions for patients with advanced and serious illness. J Gen Intern Med. 2013;28(4):570-7.
112. May P, Normand C, Cassel JB, Del Fabbro E, Fine RL, Menz R, et al. Economics of Palliative Care for Hospitalized Adults With Serious Illness: A Meta-analysis. JAMA Intern Med. 2018;178(6):820-9.
113. Farquhar MC, Prevost AT, McCrone P, Brafman-Price B, Bentley A, Higginson IJ, et al. Is a specialist breathlessness service more effective and cost-effective for patients with advanced cancer and their carers than standard care? Findings of a mixed-method randomised controlled trial. BMC Medicine. 2014;12(1):194.
114. Farquhar MC, Prevost AT, McCrone P, Brafman-Price B, Bentley A, Higginson IJ, et al. The clinical and cost effectiveness of a Breathlessness Intervention Service for patients with advanced non-malignant disease and their informal carers: mixed findings of a mixed method randomised controlled trial. Trials. 2016;17(1):185.
115. Guyatt GH, Oxman AD, Vist GE, Kunz R, Falck-Ytter Y, Alonso-Coello P, et al. GRADE: an emerging consensus on rating quality of evidence and strength of recommendations. Bmj. 2008;336(7650):924-6.
116. Kogan AC, Wilber K, Mosqueda L. Person-Centered Care for Older Adults with Chronic Conditions and Functional Impairment: A Systematic Literature Review. J Am Geriatr Soc. 2016;64(1):e1-7.
117. American Geriatrics Society Expert Panel on Person-Centered C. Person-Centered Care: A Definition and Essential Elements. Journal of the American Geriatrics Society. 2016;64(1):15-8.

For peer review only

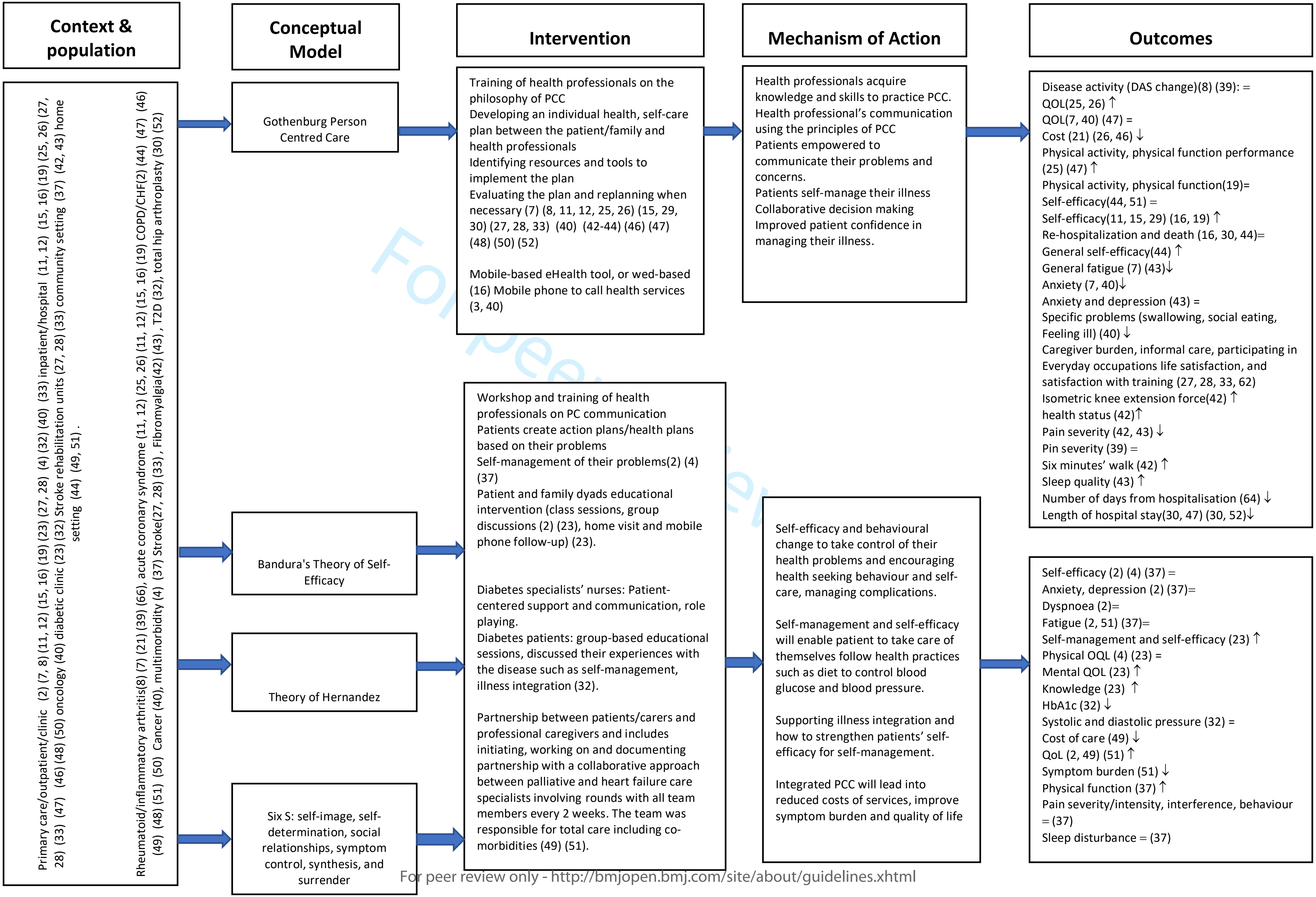

Figure 1: Logic Model for interventions with a theoretical model

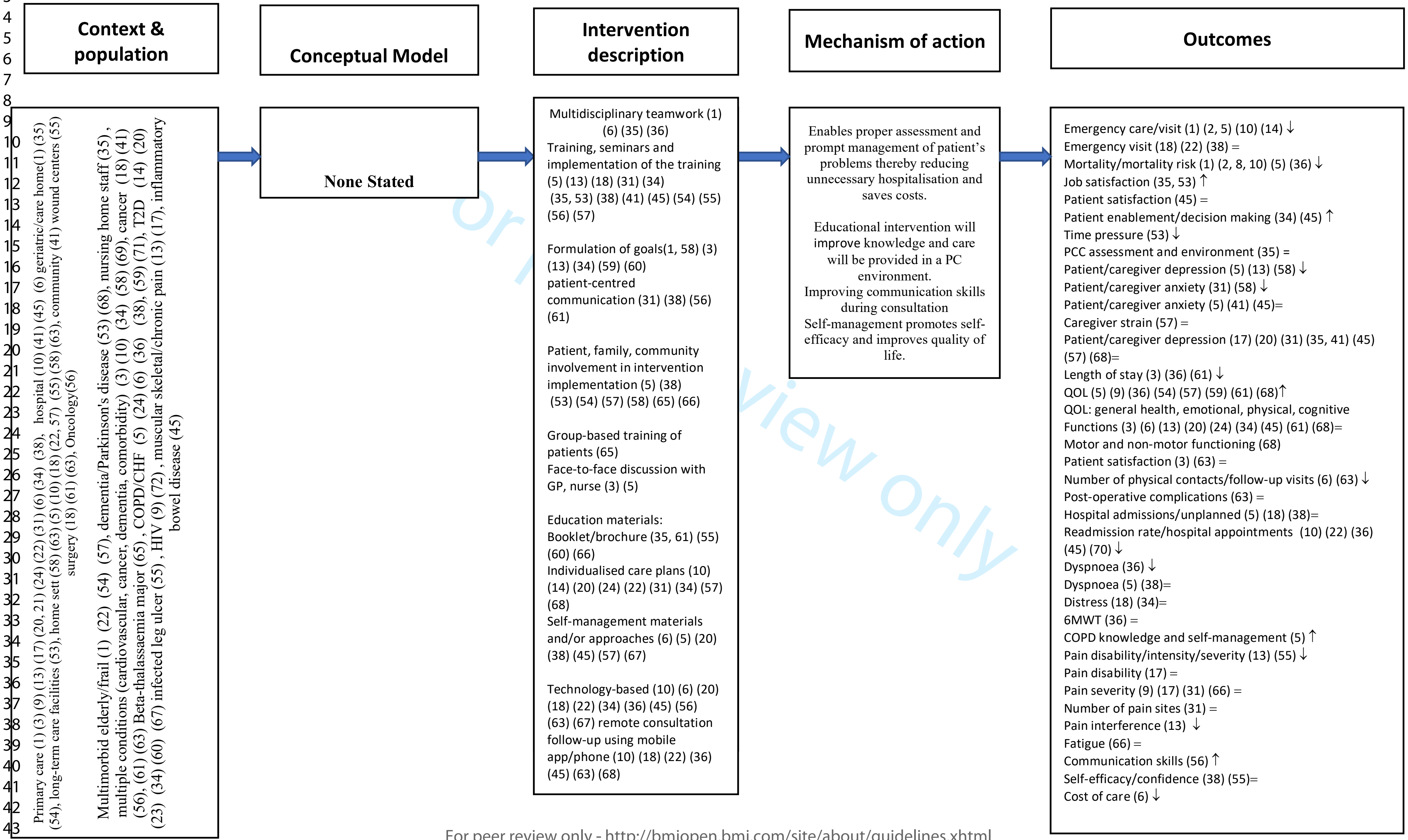

For peer review only - http://bmjopen.bmj.com/site/about/guidelines.xhtml

Figure 2: Logic model for Interventions without a theoretic model

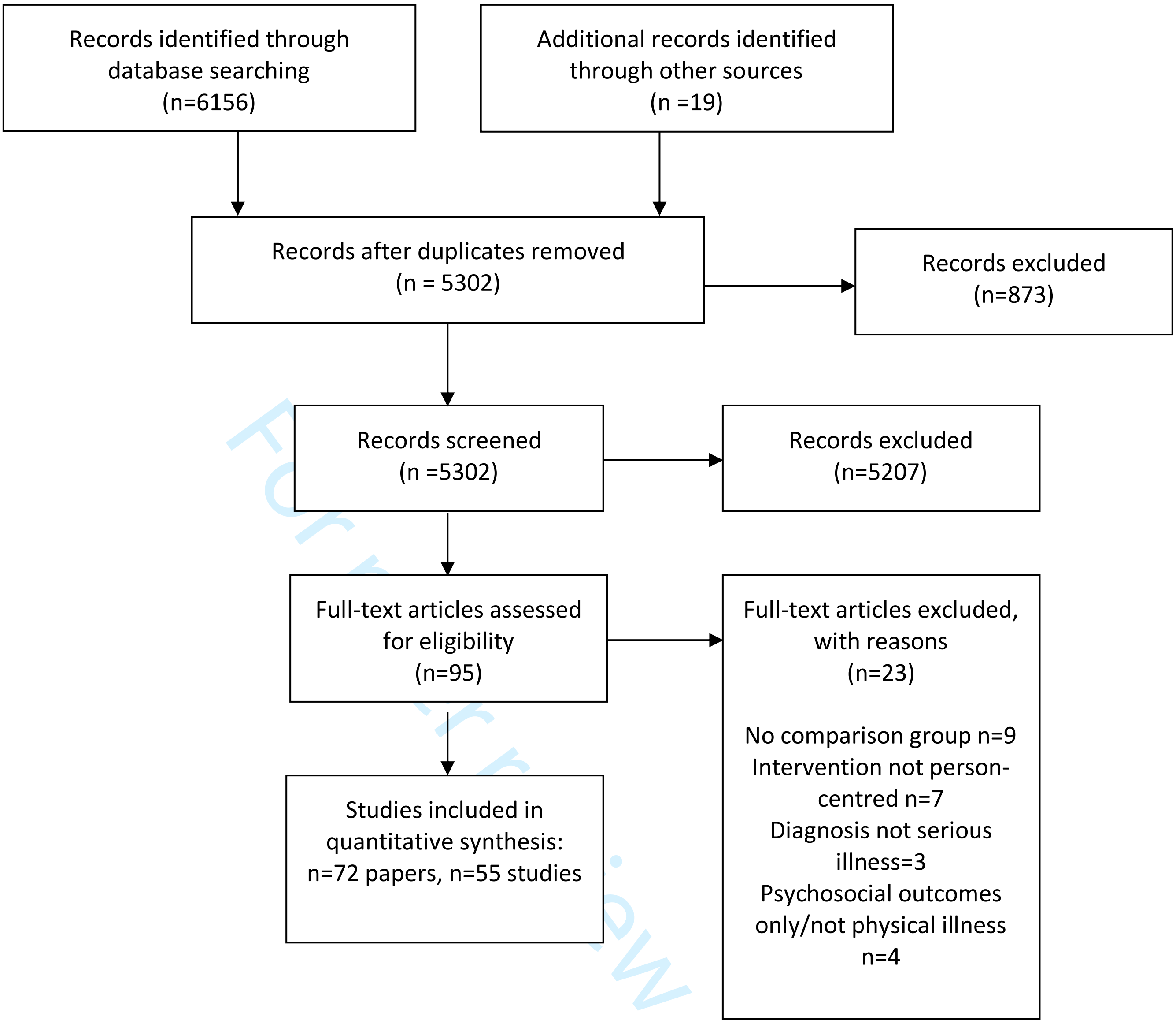

Figure 3: PRISMA Flow Diagram

For peer review only - http://bmjopen.bmj.com/site/about/guidelines.xhtml

Supplementary file 1: Searches conducted in electronic databases

Embase Classic+Embase <1947 to 2022 April 08>

1 (patient centred care or patient focused care or person centred care or person focused care or family centred care or family focused care or individualised care or individualized care or holistic care).mp. [mp=title, abstract, heading word, drug trade name, original title, device manufacturer, drug manufacturer, device trade name, keyword heading word, floating subheading word, candidate term word] 15839
2 limit 1 to yr="2020 -Current" 3865
3 (serious illnesse$ or serious illness or chronic disease or chronic illnesse$ or chronic illness or long term conditions or life limiting illnesse$ or life threatening illnesse$).mp. [mp=title, abstract, heading word, drug trade name, original title, device manufacturer, drug manufacturer, device trade name, keyword heading word, floating subheading word, candidate term word] 267608
4 limit 3 to yr="2020 -Current" 28132
5 2 and 4= **208**

https://access.ovid.com/custom/redirector/wayfless.html?idp=https://kclidpdev.kcl.ac.uk/idp/shibboleth&url=http://ovidsp.ovid.com/ovidweb.cgi?T=JS&NEWS=N&PAGE=main&SHAREDSEARCHID=28YQbqmYyLtIarRwfTMEDazD3SF1RTirGR7GVD1pZwebUiJijdYVVfOGwyYvC4Q9F

Ovid MEDLINE(R) ALL <1946 to April 08, 2022>

1 (patient centred care or patient focused care or person centred care or person focused care or family centred care or family focused care or individualised care or individualized care or holistic care).mp. [mp=title, abstract, original title, name of substance word, subject heading word, floating sub-heading word, keyword heading word, organism supplementary concept word, protocol supplementary concept word, rare disease supplementary concept word, unique identifier, synonyms] 9843
2 limit 1 to yr="2022 -Current" 463
3 (serious illnesse$ or serious illness or chronic disease or chronic illnesse$ or chronic illness or long term conditions or life limiting illnesse$ or life threatening illnesse$).mp. [mp=title, abstract, original title, name of substance word, subject heading word, floating sub-heading word, keyword heading word, organism supplementary concept word, protocol supplementary concept word, rare disease supplementary concept word, unique identifier, synonyms] 323614
4 limit 3 to yr="2020 -Current" 22416
5 2 and 4= **21**

https://access.ovid.com/custom/redirector/wayfless.html?idp=https://kclidpdev.kcl.ac.uk/idp/shibboleth&url=http://ovidsp.ovid.com/ovidweb.cgi?T=JS&NEWS=N&PAGE=main&SHAREDSEARCHID=3k81aYpUq8d9oWXfG0tpLyxhYNYL4I8uKFDxDdUBPLBxl0VFpcFdxjgQG9nGHLTIL

AMED (Allied and Complementary Medicine) <1985 to April 2022>

1	patient centred care.mp.	76
2	limit 1 to yr="2020 -Current"	7
3	patient focused care.mp.	15
4	limit 3 to yr="2020 -Current"	0
5	person centred care.mp.	37
6	limit 5 to yr="2020 -Current"	4
7	person focused care.mp.	1
8	limit 7 to yr="2020 -Current"	0
9	family centred care.mp.	35
10	limit 9 to yr="2020 -Current"	3
11	family focused care.mp.	2
12	limit 11 to yr="2020 -Current"	0
13	family focused care.mp.	2
14	individualised care.mp.	10
15	limit 14 to yr="2020 -Current"	0
16	individualized care.mp.	39
17	limit 16 to yr="2020 -Current"	6
18	holistic care.mp.	198
19	limit 18 to yr="2020 -Current"	10
20	2 or 4 or 6 or 8 or 10 or 13 or 15 or 17 or 19	30
21	serious illnesse$.mp.	18
22	limit 21 to yr="2020 -Current"	1
23	chronic illnesse$.mp.	184
24	limit 23 to yr="2020 -Current"	8
25	chronic disease$.mp.	8168
26	limit 25 to yr="2020 -Current"	449
27	life limiting condition$.mp.	67
28	limit 27 to yr="2020 -Current"	2
29	long term condition$.mp.	103
30	limit 29 to yr="2020 -Current"	4
31	22 or 24 or 26 or 28 or 30	461
32	20 and 31	3

https://access.ovid.com/custom/redirector/wayfless.html?idp=https://kclidpdev.kcl.ac.uk/idp/shibboleth&url=http://ovidsp.ovid.com/ovidweb.cgi?T=JS&NEWS=N&PAGE=main&SHAREDSEARCHID=5GZpoqjqkW0mUgtn9svLWQBU36eWXerU3Fd7QGdHRVZTQBRzrvXtT9tbtNMrEBzMp

APA PsycInfo <1806 to April Week 1 2022>

1 (patient centred care or patient focused care or person centred care or person focused care or family centred care or family focused care or individualised care or individualized care or holistic care).mp. [mp=title, abstract, heading word, table of contents, key concepts, original title, tests & measures, mesh word] 3321
2 limit 1 to yr="2020 -Current" 699
3 (serious illnesse$ or serious illness or chronic disease or chronic illnesse$ or chronic illness or long term conditions or life limiting illnesse$ or life threatening illnesse$).mp. [mp=title, abstract, heading word, table of contents, key concepts, original title, tests & measures, mesh word] 45885
4 limit 3 to yr="2020 -Current" 2633
5 2 and 4= **32**

https://access.ovid.com/custom/redirector/wayfless.html?idp=https://kclidpdev.kcl.ac.uk/idp/shibboleth&url=http://ovidsp.ovid.com/ovidweb.cgi?T=JS&NEWS=N&PAGE=main&SHAREDSEARCHID=HpRfZ61FWxXJBuPHZcLglO4z1gqDLHbl79dpT2rMPZwi5gtxtwDxWaR3c9OQEHek

MY EBSCOhost

Saturday, April 30, 2022 4:12:13 PM

#	Query	Limiters/Expanders	Last Run Via	Results
S22	S13 AND S21	Expanders - Apply equivalent subjects Search modes - Boolean/Phrase	Interface - EBSCOhost Research Databases Search Screen - Advanced Search Database - CINAHL	303
S21	S14 OR S15 OR S16 OR S17 OR S18 OR S19 OR S20	Expanders - Apply equivalent subjects Search modes - Boolean/Phrase	Interface - EBSCOhost Research Databases Search Screen - Advanced Search Database - CINAHL	87,551
S20	"long term conditions"	Expanders - Apply equivalent subjects Search modes - Boolean/Phrase	Interface - EBSCOhost Research Databases Search Screen - Advanced Search Database - CINAHL	1,903
S19	"long term condition$"	Expanders - Apply equivalent subjects Search modes - Boolean/Phrase	Interface - EBSCOhost Research Databases Search Screen - Advanced Search Database - CINAHL	467
S18	"serious illness"	Expanders - Apply equivalent subjects Search modes - Boolean/Phrase	Interface - EBSCOhost Research Databases Search Screen - Advanced Search Database - CINAHL	1,987
S17	"serious illnesse$"	Expanders - Apply equivalent subjects Search modes - Boolean/Phrase	Interface - EBSCOhost Research Databases Search Screen - Advanced Search Database - CINAHL	0
S16	"chronic illness"	Expanders - Apply equivalent subjects Search modes - Boolean/Phrase	Interface - EBSCOhost Research Databases Search Screen - Advanced Search Database - CINAHL	29,877
S15	"chronic illnesse$"	Expanders - Apply equivalent subjects	Interface - EBSCOhost Research Databases	2

		Search modes - Boolean/Phrase	Search Screen - Advanced Search<br>Database - CINAHL	
S14	"chronic disease$"	Expanders - Apply equivalent subjects<br>Search modes - Boolean/Phrase	Interface - EBSCOhost Research Databases<br>Search Screen - Advanced Search<br>Database - CINAHL	79,620
S13	S1 OR S2 OR S3 OR S4 OR S5 OR S6 OR S7 OR S8 OR S9 OR S10 OR S11 OR S12	Expanders - Apply equivalent subjects<br>Search modes - Boolean/Phrase	Interface - EBSCOhost Research Databases<br>Search Screen - Advanced Search<br>Database - CINAHL	7,546
S12	"holistic care"	Limiters - Published Date: 20200101-20220431<br>Expanders - Apply equivalent subjects<br>Search modes - Boolean/Phrase	Interface - EBSCOhost Research Databases<br>Search Screen - Advanced Search<br>Database - CINAHL	983
S11	(MH "Holistic Care")	Limiters - Published Date: 20200101-20220431<br>Expanders - Apply equivalent subjects<br>Search modes - Boolean/Phrase	Interface - EBSCOhost Research Databases<br>Search Screen - Advanced Search<br>Database - CINAHL	690
S10	"individualized care"	Limiters - Published Date: 20200101-20220431<br>Expanders - Apply equivalent subjects<br>Search modes - Boolean/Phrase	Interface - EBSCOhost Research Databases<br>Search Screen - Advanced Search<br>Database - CINAHL	199
S9	"individualised care"	Limiters - Published Date: 20200101-20220431<br>Expanders - Apply equivalent subjects<br>Search modes - Boolean/Phrase	Interface - EBSCOhost Research Databases<br>Search Screen - Advanced Search<br>Database - CINAHL	67
S8	"family focused care"	Limiters - Published Date: 20200101-	Interface - EBSCOhost Research Databases	9

		20220431 Expanders - Apply equivalent subjects Search modes - Boolean/Phrase	Search Screen - Advanced Search Database - CINAHL	
S7	"family centered care"	Limiters - Published Date: 20200101-20220431 Expanders - Apply equivalent subjects Search modes - Boolean/Phrase	Interface - EBSCOhost Research Databases Search Screen - Advanced Search Database - CINAHL	2,200
S6	(MH "Family Centered Care")	Limiters - Published Date: 20200101-20220431 Expanders - Apply equivalent subjects Search modes - Boolean/Phrase	Interface - EBSCOhost Research Databases Search Screen - Advanced Search Database - CINAHL	898
S5	(MH "Patient Centered Care")	Limiters - Published Date: 20200101-20220431 Expanders - Apply equivalent subjects Search modes - Boolean/Phrase	Interface - EBSCOhost Research Databases Search Screen - Advanced Search Database - CINAHL	4,081
S4	"patient focused care"	Limiters - Published Date: 20200101-20220431 Expanders - Apply equivalent subjects Search modes - Boolean/Phrase	Interface - EBSCOhost Research Databases Search Screen - Advanced Search Database - CINAHL	1,756
S3	"patient centred care"	Limiters - Published Date: 20200101-20220431 Expanders - Apply equivalent subjects Search modes - Boolean/Phrase	Interface - EBSCOhost Research Databases Search Screen - Advanced Search Database - CINAHL	2,006
S2	"person focused care"	Limiters - Published Date: 20200101-20220431 Expanders - Apply	Interface - EBSCOhost Research Databases Search Screen - Advanced	4

For peer review only - http://bmjopen.bmj.com/site/about/guidelines.xhtml

		equivalent subjects<br>Search modes - Boolean/Phrase	Search<br>Database - CINAHL	
S1	"person centred care"	Limiters - Published Date: 20200101-20220431<br>Expanders - Apply equivalent subjects<br>Search modes - Boolean/Phrase	Interface - EBSCOhost Research Databases<br>Search Screen - Advanced Search<br>Database - CINAHL	453

Supplementary file 2: Characteristics of studies included in the review N=55

Study Number	Author & Year/ Country	Aim Design Theoretical model	Sample	Intervention(s)	Outcomes/measures and follow-up period	Results
1	Fortin et al 2021 (1)<br><br>Canada	To measure the effectiveness of a 4-month interdisciplinary multifaceted intervention based on a change in care delivery for patients with multimorbidity in primary care practices.<br><br>RCT<br><br>Chronic Care Model and Patient-Centred Clinical Method	N=284 patients with multimorbidity (n=144 mean age (SD) 60.8 (10.6) intervention and n=140 mean age (SD) 61.1 (10.3) control)	Consisted of: (1) training the professionals on patient-centered care for persons with multimorbidity, self-management support, interprofessional collaboration, and motivational approach. (2) suggested clinical pathways for patients, with individual visits to health care professionals were developed for each patient. Pathways started with a contact nurse who performed a clinical assessment, elicited patients' goals, and created an individualized care plan. Patients were then referred to the most appropriate professional(s) matching patient goals, including referrals to the nurses themselves. A final visit was with the contact nurse to summarize and plan for sustainability. and (3)	Primary outcomes:<br>1. Health education impact: Health Education Impact Questionnaire<br><br>2. Self-Efficacy: Self-Efficacy for Managing Chronic Diseases<br><br>Secondary outcomes:<br><br>3. Health status: Veterans RAND<br><br>4. Quality of Life: EuroQoL<br><br>5.Psychosocial distress: Kessler Psychological Distress Scale Questionnaire<br><br>6.Health behaviours: Behavioural Risk Surveillance System Outcomes collected at baseline and month 4	1-5 No statistically significant differences<br><br>6. Significant differences on physical activity and healthy eating, but not significant on high-risk alcohol

Study Number	Author & Year/<br><br>Country	Aim<br>Design<br>Theoretical model	Sample	Intervention(s)	Outcomes/measures and follow-up period	Results
				creating a community of practice within each family medicine group (FMG).<br><br>Patients assigned to the control group were placed on a waiting list to receive the intervention after 4 months.<br>In the meantime, they had access to their usual care including elective appointments with their family doctors or urgent appointments with their heath care professionals for acute reasons (trauma, infection, etc).		consumption and smoking habit.
2	de Batlle, 2020 (2)<br><br>Spain	To assess the effectiveness and cost-effectiveness of the implementation of a mobile health (mHealth)-enabled integrated care model for complex chronic patients.<br><br>a prospective, pragmatic, two-arm,	Elderly patients with COPD, heart failure and caregivers<br><br>N=52 integrated care model, mean age (SD): 82(7)<br><br>n=35 usual care, mean age (SD): 82(8).	The combined benefits of the CONNECARE (Personalised Connected Care for Complex Chronic Patients) organizational integrated care model and the eHealth platform supporting it, consisting of a (i) self-management app, with status and performance reports, a	1. Quality of life (changes in health status): 12-Item Short-Form Survey (SF-12), Barthel index for Activities of Daily Living and, Hospital Anxiety and Depression scale<br><br>2. Use of health care resources and estimated	1. No significant differences between the two groups (mean change (SD) 5.0 (5.2) p= .10<br><br>2.Unplanned visits were significantly

For peer review

Study Number	Author & Year/<br><br>Country	Aim<br>Design<br>Theoretical model	Sample	Intervention(s)	Outcomes/measures and follow-up period	Results
		parallel implementation trial		virtual coach with customizable automated feedback, and full communication with the care team; (ii) a Fitbit Flex 2 digital activity tracker and any additional sensor deemed necessary by the care team including a digital pulse-oximeter, digital scale, and digital blood pressure monitor, that were fully integrated into the self-management app; (iii) a patient profile in the SACM (Smart Adaptive Case Management) web-based platform, accessible to all members of the care team (family physicians, hospital specialists, and social workers), that was used for coordination and communication among professionals in the different settings, and to contact the patient when needed; and (iv) assignment of a case manager in charge of supervising the whole process and	associated costs based on Catalan Health Department official data: Unplanned visits and admission<br><br>3. cost-effectiveness, based on the improvement in QoL relative to costs, assessed by means of the incremental cost-effectiveness ratio (ICER);<br><br>Data collected at baseline and a 6-month follow up,	lower in the intervention group (2.3 (3.1) vs 1.0 (1.1) P=0.004).<br><br>3. The integrated care program generated savings from US $584 to $1434 per patient, depending on the scenarios. The integrated care program was cost-effective according to the ICER, performing better in terms of QoL while reducing overall expenses

For peer review only

Study Number	Author & Year/ Country	Aim Design Theoretical model	Sample	Intervention(s)	Outcomes/measures and follow-up period	Results
				serving as the main patient contact point.<br><br>Control group received usual care (details not provided).		
3	Mielenz et al 2020 (3)<br><br>USA	To evaluate the Self-management Resource Center Small Group Programs (SMRCSGP), plus wellness coaching, as a booster intervention in older adults with chronic diseases.<br><br>To evaluate the role of personal health records (PHR) prototype as the linkage between the clinic and community.<br><br>RCT<br><br>Self-efficacy theory	Elderly people >55 years old.<br>N=125<br>Intervention n=62, mean age (SD) 72 (0.94)<br><br>Control n=63, mean age (SD) 73.1 (0.95)	The intervention: The wellness self-coaching program asked participants to create a "Wellness Vision," wherein the participants set monthly and weekly behavioural goals that were agreed upon by participant and coach. Class lesson titles were as follows: taming frenzy, self-compassion, focus, mindfulness, strengths (two-part), motivation, legacy, creativity (two-part), body intelligence (two-part), relationships (two-part), positivity (two-part), meaning (two-part), curiosity (two-part), standard setter (two-part), self-leadership, and your plan to thrive.	Primary outcomes<br>1. Physical activity: The Community Health Activities Model Program for Seniors (CHAMPS) was used to collect information on physical activity.<br>-Frequency per week of all exercise-related activities<br>-Hours per week of all exercise-related activities<br><br>2.Behavioral Risk Factor Surveillance System physical activity measures<br>-Met aerobic physical activity guidelines,<br>-Met aerobic and muscle strengthening guidelines,	Across the 6 months of our study the intervention and control groups did not vary significantly on any primary physical activity outcomes of interest (CHAMPS and BRFSS measures) in models.<br><br>The intervention and control groups did vary significantly (p = .03) over time on one secondary outcome: the PROMIS physical function variable. Although both groups reported

For peer review only

Study Number	Author & Year/ Country	Aim Design Theoretical model	Sample	Intervention(s)	Outcomes/measures and follow-up period	Results
				Control: Both groups received usual care consisting of self-management Resource Center Small Group Programs (SMRCSGP) (including programs on general chronic disease and specific conditions: arthritis, diabetes, HIV, chronic pain, and cancer) are structured wellness interventions that encourage self-management in older adults living with chronic conditions and are implemented by lay leaders	Secondary outcomes:<br>3. Patient-Reported Outcomes Measurement Information System (PROMIS) v1.0 short form (SF) measures:<br><br>Depressin: Emotional Distress-Depression—SF<br><br>Fatigue: Fatigue—SF 4a,<br><br>Pain behaviour: Pain Behavior—SF 7a,<br><br>Pain intensity: Pain Intensity—SF 3a,<br><br>Pain interference: Pain Interference—SF 4a,<br><br>Physical function: Physical Function—SF20a),<br><br>Sleep: Sleep Disturbance—SF 4a.	improvements on this measure over time (higher scores indicating that participants can do more and feel better), overall improvement was greater for the wellness coaching intervention group (2.6) than for the control (0.6).

For peer review only

Study Number	Author & Year/<br><br>Country	Aim<br>Design<br>Theoretical model	Sample	Intervention(s)	Outcomes/measures and follow-up period	Results
					4. Medical care questions:<br>- Times visiting a physician<br>- Times visiting a hospital emergency department<br>- Times hospitalized for one night or longer<br>- Total nights spent in the hospital<br><br>-Self-efficacy for exercise was assessed on the Resnick Self- Efficacy for Exercise (SEE)<br>-Falls in the past month<br><br>CHAMPS data were collected at baseline, 3 months, and 6 months.	
4	Yu et al 2020 (4)<br><br>Canada	To assess the impact of 'MyDiabetesPlan' on decisional conflict, diabetes distress, health-related	N=102 patients<br>n=29 clinicians<br><br>N=111 patients<br>n=24 clinicians	A web-based PtDA in which patients populate their cardiometabolic and psychosocial profiles and general care	Primary outcome:<br>1. Decisional conflict: the Decisional Conflict Scale (DCS),<br><br>Secondary outcomes:	1. No significant differences between the two groups; mean 0.5; p=0.08

For peer review only

Study Number	Author & Year/ Country	Aim<br>Design<br>Theoretical model	Sample	Intervention(s)	Outcomes/measures and follow-up period	Results
		quality of life, and patient assessment of chronic illness care at the individual patient level.<br><br>Cluster RCT		priorities: MyDiabetesPlan then generates individualized diabetes-specific goals and strategies based on these inputs that the patients then select, resulting in an action plan. Clinicians at intervention sites underwent a one-on-one 60-min tutorial in their clinic room by the research coordinator, with access to a one-page how-to guide and 2-min video. During subsequent clinical encounters, a member of the interprofessional team (nurse or dietitian) logged into MyDiabetesPlan and completed it with the patient; the physician subsequently reviewed the resultant action plan with the patient. At 6 months, patients at intervention sites were provided with a patient-directed how-to guide and video and	2. Diabetes distress: Diabetes Distress Scale (DSS)<br><br>3., Health-related quality of life: SF-12<br><br>4. Chronic illness care: PACIC (Patient Assessment of Chronic Illness Care) Scale<br><br>5. intention to engage in IPSDM (Interprofessional Shared Decision-Making): CPD (Continuing Professional Development.) Reaction Questionnaire<br><br>Outcomes were assessed at the individual participant level, at baseline, and at 6 months and 12 months (after an appointment) through a web-based survey or by mail.	2. mean change 0.2 p=0.12<br><br>3. mean change 1.2 p=0.57<br><br>4. Mean change 0.15 p<0.001<br><br>5. No significant differences between two groups.

Study Number	Author & Year/ Country	Aim Design Theoretical model	Sample	Intervention(s)	Outcomes/measures and follow-up period	Results
				directed to update MyDiabetesPlan according to their progress before the appointment.<br><br>Clinicians in the control sites received paper copies of the executive summary of the Diabetes Canada clinical practice guidelines, and a postcard outlining web-based clinical information resources. After 6 months, patients in the control sites received a Diabetes Canada patient education pamphlet regarding diabetes self-management and a postcard outlining web-based additional patient resources.		
5	Bergsten et al 2019 (5)<br><br>Sweden	To evaluate the effect of a nurse-led clinic with frequent visits, treat-to-target and person-centred care of patients with rheumatoid arthritis and moderate-to-high	N=70 patients with moderate to severe symptoms.<br><br>n=36 intervention group, mean age 60.3 (SD 15.9),	4 nurses attended 2 days' training on principles, philosophy, and delivery of person-centred care.<br>An individual health plan agreed by patient and nurse, including aims for disease activity and	(1) Primary outcome was the difference in the DAS28 change: DAS28 is an index based on the number of tender and swollen joints, patients' global health assessment and the	In the PP analyses, the primary outcome (i.e., the difference in delta-DAS28 between the IG and CG) was not statistically

For peer review only

Study Number	Author & Year/<br><br>Country	Aim<br>Design<br>Theoretical model	Sample	Intervention(s)	Outcomes/measures and follow-up period	Results
		disease activity compared with patients receiving regular care.<br><br>RCT<br><br>Gothenburg PCC	n=34 control group, mean age 62.4 (SD 12.2).	participation, tools to achieve these goals.<br><br>Patients in the control group were offered a telephone appointment with their regular physician, in order to discuss their disease activity and whether a physical appointment, and potentially a change in therapy, should be made. All patients were then followed by their treating physician according to regular care, with follow-up visits decided either at this telephone appointment or according to previous plans. In regular care, the patients usually visited the clinic every 6–12 months. As part of regular care, patients also had the possibility of making appointments with the physician in the event of flares.	erythrocyte sedimentation rate.<br><br>Secondary outcomes:<br><br>(2) the proportions with minimal clinical important improvement in DAS28 (>0.6)<br><br>(3) the proportions achieving low disease activity (DAS28 <3.2);<br><br>(4) the proportions achieving a EULAR moderate or good response<br><br>(5) the Health Assessment Questionnaire score, measuring daily function<br><br>(6) the RA impact of disease (RAID) score, measuring the impact of RA from the patient's perspective;	significant (0.43; 95% CI −0.27, 1.13)<br><br>Nonsignificant difference in ITT primary PCC in DAS 26 (mean (95% CI)): 1.39 (0.97 to 1.82) v control 1.04 (0.54 to 1.53).<br><br>In PP PCC 1.50 (1.00 to 2.00) v control 1.07 (0.56 to 1.57). Trial inclusion terminated because more patients in the interventions dropped out

For peer review only

Study Number	Author & Year/ Country	Aim Design Theoretical model	Sample	Intervention(s)	Outcomes/measures and follow-up period	Results
					(7) Patient Acceptable Symptom State (PASS) score (8) the Beliefs about Medicines Questionnaire (BMQ) responses, measuring patients' attitude to medication split in two domains (BMQ-necessity, BMQ-concerns)<br><br>(9) the EuroQol-5D (EQ-5D) score).	
6	Berntsen et al (2019)(6)<br><br>Norway	To determine if the Patient-Centred Team Intervention (PACT) causes reduced use of high-level emergency care and increased use of low-level planned care with unchanged mortality risk for the multi-morbid elderly<br><br>Parallel arm study	N=1218 patients >60 years, with multi-morbidity, complex long-term needs and high short-term risk for emergency hospital admission n=439 intervention group, referred to the PACT team. Mean age 80.02 (SD8.72)	Intervention: Patient is assigned to a mini-team of nurse co-ordinator, physician, physiotherapist, occupational therapist and pharmacist. They work with the patient to explore goals using a person-centred approach including a comprehensive geriatric assessment methodology. The team address immediate clinical needs and co-ordinate Average intervention time 30 days.	1. Number of emergency admissions<br><br>2. Sum of emergency inpatient bed days<br><br>3. Count of emergency re-admissions within 30 days of discharge<br><br>4. Count of planned out-patient visits<br><br>5. Count of emergency outpatient visits	1. Adjusted RR 0.90 (95%CI: 0.82-0.99)<br><br>2. Adjusted RR 0.68 (95%CI 0.52-0.79)<br><br>3. Adjusted RR 0.72 (95%CI 0.41-1.24)<br><br>4. Adjusted RR 2.27 (95%CI 2.02-2.55)<br><br>5. Adjusted RR 0.90 (95%CI 0.68-1.2)

For peer review only

Study Number	Author & Year/ Country	Aim Design Theoretical model	Sample	Intervention(s)	Outcomes/measures and follow-up period	Results
			n=779 control group, mean age 78.8 years (SD 8.68). Patients had an emergency admission but not received PACT intervention. A matched local and distant control was sought for each intervention participant.	Control group: usual care defined as evidence-based care for the cause of the emergency admission to hospital, referral for other diagnoses to GP or specialist care and standard electronic communication.	6. Mortality risk at 3 and 6 months follow-up Follow up began at first referral to PACT (IG) or time of emergency admission (CG) and ended after 6 months or death.	6. Adjusted RR 0.39 (95%CI 0.22-0.7) at 3 months and 0.57 (95%CI 0.34-0.94) at 6 months.
7	Berendonk (2019) (7)<br><br>Germany.	To test the feasibility of a nursing intervention (DEMIAN) in routine care and its effects on care providers' job satisfaction, motivation and work strain.<br><br>Pragmatic two-group cluster RCT	N=20 German long-term care facilities n= 84 care providers (mean age 41.8, SD 10.2) and 42 residents with dementia in intervention group n= 96 care providers (mean age 38.5, SD 11.9) and 42 residents with	Intervention: Registered nurses completed two days of training within a two week period on the DEMIAN intervention. Its objectives are to gather information on meaningful situations for each individual and to use this knowledge to plan and provide care. There was a 6 week implementation phase after training to carry out mini-interventions. Nurses encourages all team members, relatives and	1. Screening instrument for job strain in human service work (BHD)<br><br>2. Modified Task and Job Analysis Tool- residential LTC version (TAA-A) Baseline assessment and at post intervention follow up	1. Greater job satisfaction in IG than CG post intervention (p=0.053)<br><br>2. Most TAA-A outcomes did not differ significantly between IG and CG after intervention. Time pressure did decrease in IG compared to CG (p=0.026)

Study Number	Author & Year/<br><br>Country	Aim<br>Design<br>Theoretical model	Sample	Intervention(s)	Outcomes/measures and follow-up period	Results
			dementia in control group	volunteers to be involved in the interventions.<br><br>Control: usual care (details not provided).		
8	Bökberg et al (2019) (8)<br><br>Sweden	To evaluate whether an educational intervention had any effect in the staff's perception of providing person-centred palliative care for older persons in nursing homes.<br><br>Pre- and post-test experimental design.	N=365 nursing home staff (nurses, assistant nurses, physiotherapists, occupational therapists, social workers and unit managers) recruited from 20 urban and rural, small (<25 residents) and large (>100 residents) nursing homes in two Swedish counties<br>n=167 intervention group, median age 47<br>n=198 control group, median age 49 years	Intervention: A knowledge-based palliative care intervention consisting of five 2h educational seminars for nursing home staff based on Swedish national documents on the key principles of palliative care intending to improve quality of life for individuals and their families. Participants were provided with a study booklet. The intervention was implemented over 6 months.<br><br>Control: usual training. None of the participating homes had had workplace education or training in palliative care before the intervention.	1. Person-centred Care Assessment Tool (P-CAT)<br><br>2. Person-Centred Climate Questionnaire (PCQ-S)<br><br>Data collected at baseline and post-intervention	1. No significant change in total P-CAT score pre- and post intervention in IG (p=0.715)or CG (p=0.601)<br>No statistically significant changes in pre and psot intervention scores on any subscale for either group.<br><br>2. No significant change in total PCQ-S scores pre and post intervention in IG (p=0.685) or CG (p+0.451)<br>No statistically significant changes in pre and post intervention scores on any subscale for either group.

For peer review only

Study Number	Author & Year/ Country	Aim Design Theoretical model	Sample	Intervention(s)	Outcomes/measures and follow-up period	Results
9	Britt et al (2019) (9)<br><br>USA	To assess the effect of the LifeCourse (LC) programme on healthcare utilisations<br><br>Quasi-experimental trial	N=903 patients estimated to be within 3 years of end of life with 1+ serious illness n=450 intervention, mean age 78.1 (SD 12.0) n= 453 control, mean age 74.3 (SD 12.5) recruited from area hospitals or care centres	Intervention: Hour long, monthly home visits for patients and caregivers if the patient desired. Structured visits included setting intentions, discussing goals and guided assessments with the aim of enabling patients to articulate what mattered to them and their goals for living. Visit delivered by a community health worker who had undertaken a 2 week training programme.<br><br>Control: Usual care – standard medical care including palliative, care management, home care, and/or hospice care services	1. Patient healthcare utilisation<br><br>2. Patient Quality of Life: FACIT-Pal<br><br>3. Patient care experience	1. Higher proportion of IG completed an advanced directive than CG (173 vs 66, p<0.001). No significant difference in hospice use between dying patients in IG and CG. IG patients spent longer in hospice than CG (88 days vs 44 days, p<0.18). No significant differences between groups in days spent in the ED, hospital or ICU.<br><br>2. No difference between groups (p=0.649)<br><br>3. IG reported greater improvement in the communication domain than CG (p=0.16). No other statistically

Study Number	Author & Year/ Country	Aim Design Theoretical model	Sample	Intervention(s)	Outcomes/measures and follow-up period	Results
						significant treatment by time effects.
					4. Caregiver experience	4. No effect
					5. Caregiver quality of life: PROMIS-29 Measures collected at baseline then every 3 months until death or 30 months	5. CG carers had greater increase in anxiety and depression domains compared to IG (B=-0.98, p=0.038 and B=-0.098, p=0.014). No other statistically significant treatment by time effects.

For peer review only

Study Number	Author & Year/ Country	Aim Design Theoretical model	Sample	Intervention(s)	Outcomes/measures and follow-up period	Results
10a	Hedman, et al 2019 (10)<br><br>Sweden	To compare five-year outcomes and changes over time of a client-centred activities of daily living (ADL) intervention versus usual ADL interventions for people with stroke and their significant others.<br><br>RCT<br><br>Gothenburg PCC	People with stroke and significant others.<br><br>N=145 people with stroke (intervention group: n = 71): mean age (SD): 71(9)<br><br>control group: n = 74): mean age (SD): 68 (9)<br><br>N=75 significant others (intervention group: n = 36): mean age (SD) 65 (17)<br><br>(control group: n = 39): mean age (SD) 69 (10).	Intervention: Participants with stroke received an occupational therapist delivered client centred ADL intervention aiming to increase agency in daily activities and participation in everyday life guided by their expressed desires. Occupational therapists had participated in a 5 day workshop on client centredness.<br><br>Control: Rehabilitation in a unit providing usual ADL interventions	Primary outcome<br>1. Perceived participation: Stroke Impact scale<br><br>Secondary outcome:<br>2. Perceived participation: Occupational gaps questionnaire<br><br>3. Frequency of participation in social and complex everyday activities: Frenchay Activities Index<br><br>4. Self-reported use of assistance (yes/no) in six personal and four instrumental ADL: The Katz Extended Scale<br><br>5. Perceived self-efficacy in performing everyday activities: a Self-Efficacy Scale<br><br>6. Overall satisfaction with life: Life Satisfaction Scale	For patients:<br>1. Mean difference –6.5 (–13.3 to 0.3), p= 0.062<br><br>2. Mean difference 0.7 (–0.6 to 2.0), p=0.293<br><br>3. Mean difference –0.2 (–3.2 to 2.7), p=0.885<br><br>4. Odds ratio 0.4 (0.2 to 0.8) p=0.012<br><br>5. Mean difference 2.7 (–8.2 to 13.6), p=0.621<br><br>6. Odds ratio 0.6 (0.2 to 1.3), p= 0.219

For peer review only

Study Number	Author & Year/ Country	Aim Design Theoretical model	Sample	Intervention(s)	Outcomes/measures and follow-up period	Results
					7. Globally assess perceived quality of life: Reintegration into normal living index<br><br>8. Mood: Hospital anxiety and depression scale<br><br>9. Fatigue severity: fatigue severity scale<br><br>For significant others:<br><br>10. Burden of care: caregiver burden scale<br><br>11. Informal care was assessed by the use of the question 'To what extent do you assist your significant other?'	7. Mean difference –0.6 (–3.0 to 1.8), p=0.617<br><br>8. Anxiety: mean difference –0.3 (–1.6 to 1.0) p=0.611 Depression: mean difference –0.4 (–1.6 to 0.7), p=0.474<br><br>9: Mean difference –2.6 (–6.9 to 1.8), p=0.245<br><br>:<br>10: Mean difference −4.7 (−12.0 to 2.5), p=0.196<br><br>11: Mean difference −6.0 (−20.1 to 8.1), p=0.402

For peer review only

Study Number	Author & Year/<br><br>Country	Aim<br>Design<br>Theoretical model	Sample	Intervention(s)	Outcomes/measures and follow-up period	Results
					12. Mood: HADS as above<br><br>13. The overall satisfaction with life: The 'My life as a whole' item in LiSat-11 was used to assess<br><br>14. Restrictions (gaps) in participation in everyday occupations: The 30-item version of the Occupational Gaps Questionnaire.	12. Significant differences between two groups −1.7 (−3.0 to −0.5); p=0.005<br><br>13: Odds 1.1 (0.4 to 2.8) p=0.922<br><br>14: Mean difference −0.6 (−2.0 to 0.7), p=0.329
10 b, c, d	Bertilsson et al (2016) (11)<br><br>Guidetti et al (2015) (12)<br><br>Bertilsson et al (2014) (13)<br><br>(Four papers one study)	a) To determine if a client centred activity of daily living (ADL) group after stroke has an effect on caregiver burden, provision of informal care, perceived participation in everyday occupations and life satisfaction.	N= 183 caregivers of people with stroke attending inpatient or home rehabilitation<br>n=88 intervention group, mean age 60 (SD 14.6)<br>n=95 control group, mean age 64 (SD 13.1)	As above	1. Caregiver burden: Caregiver Burden Scale.<br><br>2. Informal care: percentage reporting providing assistance with personal ADLs, instrumental ADLs or other activities.	1. No difference between intervention and control groups at 12 months (42.7 vs 41.8, p=0.75).<br><br>2. No difference between intervention and control groups in for personal ADLs (42 vs 50%, p=0.51), Instrumental ADLs (67 vs 68%, p=0.88)

For peer review only

Study Number	Author & Year/<br><br>Country	Aim<br>Design<br>Theoretical model	Sample	Intervention(s)	Outcomes/measures and follow-up period	Results
	Sweden	b) To compare changes regarding perceived participation, independence in activities of daily living (ADL) and life satisfaction between 3, 6 and 12 months after inclusion in a study of a client-centred ADL intervention and usual ADL intervention after stroke.<br><br>c) To study a client-centred activities of daily living (ADL) intervention (CADL) compared with the usual ADL intervention (UADL) in people with stroke regarding: independence in ADL, perceived participation, life satisfaction, use of home-help service, and satisfaction with training.<br><br>Cluster RCT	N=280 people with stroke<br><br>Intervention n=129, mean age (SD) 74 (10)<br><br>Control n=151, mean age (SD) 71 (10.8)		3. Participation in everyday occupations: Occupational Gaps Questionnaire (OGQ).<br><br>4. Life satisfaction: Life satisfaction scale (LiSat-11)<br>Outcomes measured at 3 and 12 months	or other support (65 vs 76%, p=0.09) at 12 months.<br><br>3. No difference between intervention and control groups (3.5 vs 4.0, p=0.52) at 12 months.<br><br>4. No difference between intervention and control groups (47 vs 47%, p=0.87) at 12 months<br>No differences between intervention and control groups in changes in outcomes between 3 and 12 months. Except the intervention group had lower General strain at 12 months than 3 months (OR 1.74, p=0.014).

Study Number	Author & Year/ Country	Aim Design Theoretical model	Sample	Intervention(s)	Outcomes/measures and follow-up period	Results
					5. Independence on ADL: Katz Extended scale (KE)<br><br>6. Perceived participation: Stroke Impact Scale (SIS)<br><br>7. Participation in everyday occupations: Occupational Gaps Questionnaire (OGQ).<br><br>8. Life satisfaction: The Life Satisfaction Scale<br><br>9. Home-help service and satisfaction with training: Self-reported (yes/no) by people with stroke.<br><br>Measures at three, six and twelve months.	5. Intervention n=38; 29.4% vs control n=52; 34.4% p=0.83<br><br>6. No significant different between groups in all 9 items.<br><br>7. Mean OGQ 9.1 intervention, 107 control; p=0.10<br><br>8. N=47 (36.4%) intervention vs n=56 (37.1%) control; p=0.79<br><br>9. Home help service n=57 (44.2%) intervention vs n=60 (39.7%) control; p=0.54 Satisfaction with training n=94 (72.9%) vs n=105 (69.5%); p=0.33

For peer review only

Study Number	Author & Year/ Country	Aim Design Theoretical model	Sample	Intervention(s)	Outcomes/measures and follow-up period	Results
11	Ohlen et al (2019) (14)<br><br>Sweden	To evaluate whether an intervention with a person-centred approach to information and communication for patients diagnosed with colorectal cancer undergoing surgery can improve the patients' preparedness for surgery, discharge and recovery during six months following diagnosis and initial treatment<br><br>Quasi-experimental longitudinal study.	People undergoing elective surgery for cancer in the colon or rectum n=238 intervention and n=250 control.	Intervention has two components:<br>1) Written interactive patient education materials tool pertaining to phases of care process (examination, diagnosis, surgery, and recovery).<br>2) Person-centred communication in dialogue format using patient education materials. This was the tool used to communicate between the patient and health professionals.<br><br>Control group: Patients received several written patients education materials related to specific parts or procedures related to surgery and recovery. Communication occurred according to standard care.	1. The Longitudinal Preparedness for Colorectal Cancer Surgery Questionnaire (PCSQ) in Swedish measures preparedness for surgery and recovery	1. Relative to the control group, patients in the intervention group reported less decline in the domain "searching for and making use of information" (slopes for control and intervention groups were -18.8 and -14.8, respectively, p = 0.01). Relative to the intervention group, the control group participants reported lower scores for the domain "making sense of the recovery process" at time point 1 pre-surgery (intercepts were 80.9 and 84.4 in the control and intervention groups, p = 0.04) but no difference was

Study Number	Author & Year/<br><br>Country	Aim<br>Design<br>Theoretical model	Sample	Intervention(s)	Outcomes/measures and follow-up period	Results
					Length of stay	detected in the slope of the trajectory. There were no statistically significant differences in intercepts or slopes between the two groups for “understanding and involvement in the care process” and “support and access to medical care.<br><br>The length of stay patients who were hospitalized in relation to surgery was 8.8 days (median = 8.0) for the control group compared with 8.0 days (median = 7.0) in the intervention group (N = 488, p = 0.033, based on the logarithm of length of stay).

For peer review only

**Study Number**	**Author & Year/ Country**	**Aim Design Theoretical model**	**Sample**	**Intervention(s)**	**Outcomes/measures and follow-up period**	**Results**
					2. EORTC QLQ-C30 version 3.0 (30 items) is a widely used measure of HRQOL for patients diagnosed with cancer and the Swedish version was used<br><br>3. The National Comprehensive Cancer Network (NCCS) Distress Thermometer (DT; Version 1.2013) was used to detect clinically significant distress in patients	2. Patients also reported a decline in their role function; however, there was a statistically significant difference in the slopes between the two groups (-17.5 versus -7.9 in the control and intervention groups, p = 0.01).<br><br>General health, emotional function, physical function, and cognitive functions were not significant.<br><br>3. No statistically significant differences detected between the two groups

For peer review only

Study Number	Author & Year/ Country	Aim Design Theoretical model	Sample	Intervention(s)	Outcomes/measures and follow-up period	Results
					Outcomes collected at six weeks, three and six months.	
12a	Pirhonen et al 2019 (15)<br><br>Sweden	To calculate the cost-effectiveness of a person-centred care intervention compared with usual care in patients with acute coronary syndrome (ACS)<br><br>RCT<br><br>Person-centred care according to the framework by the Gothenburg Centre for Person-Centred Care (GPCC)	N=252<br>n=124 intervention,<br><br>n=128 control<br><br>(1) age < 75 years, and (2) were hospitalised for myocardial infarction or unstable angina pectoris.	The intervention group received person-centred care according to the framework developed by the Gothenburg Centre for Person-Centred Care (GPCC), which comprises routines for establishment of a partnership between patients and healthcare professionals.<br>The intervention was provided by designated healthcare professionals (physicians and registered nurses), at each care level, who had received training through lectures, seminars, and workshops on how to apply the intervention.	1. Quality of life: EQ-5D-3L questionnaire<br><br>2. Direct Costs and Productivity Losses: in and outpatient care visits, diagnosis related costs, pharmaceutical costs productivity losses (indirect costs) associated with temporary and permanent illness, valued according to the human capital method, that is, time units of lost production were valued at their market value.	The base-case calculations showed that person-centred care was more effective and less costly compared with usual care for patients under 65 years of age, while usual care was more effective and less costly in the older age group.<br><br>The cost-effectiveness of the intervention was found to differ between the two age groups (< 65 years with 117 patients

Study Number	Author & Year/ Country	Aim Design Theoretical model	Sample	Intervention(s)	Outcomes/measures and follow-up period	Results
				Professionals listened carefully to the patient's narrative in order to include his or her needs and intrinsic personal resources relevant for the treatment and care process. Based on this narrative, a health plan was co-created, which reflects both the perspective of the patient and the expertise of the healthcare professionals. The health plan also contained agreed goals for the recovery period, which were followed-up and revised by the patient together with the designated healthcare professionals at each care level when necessary.<br><br>Control: Both the intervention group and the control group received usual care according to national guidelines for cardiac care	Data collected at baseline, months 1, 2 and 6 (clinical endpoint) and 1 year after the initial hospital discharge. Information on total healthcare utilisation, sickness absenteeism and drug prescriptions were collected for the 1-year period	and ≥ 65 years with 75 patients). In the younger age group, the intervention induced lower total costs and higher quality of life, while the opposite was true in the older age group. Thus, the person-centred care intervention was the cost effective alternative when compared with usual care for those under the age of 65 years, while usual care was the cost-effective alternative in the older age group.

For peer review only

Study Number	Author & Year/<br><br>Country	Aim<br>Design<br>Theoretical model	Sample	Intervention(s)	Outcomes/measures and follow-up period	Results
12b	Pirhonen et al 2017 (16)<br><br><br>Sweden<br><br>(One study reporting two papers).	To study the effects of person-centred care provided to patients with acute coronary syndrome, using four different health-related outcome measures and to examine the performance of these outcomes when measuring person-centred care.<br><br>RCT<br><br>Person-centred care according to the framework by the Gothenburg Centre for Person-Centred Care (GPCC)	The intervention n= 94 and control n=105 patients.<br><br>All other details as above	1) Patients and clinician Hansson s identify and discuss problems caused by or related to the patient's condition(s), giving due consideration to both clinical tests and treatments and the practical, social, and emotional effects of their condition(s) and treatment(s) on their daily lives.<br>2) They then engage in a shared decision-making process involving goal setting and action planning, focused on determining priorities, agreeing about realistic objectives, solving specific problems, and identifying relevant sources of support.<br>3) The agreed plan is documented and followed up.<br><br>Both groups received six-months of standard care comprised of a sequence of inpatient care, hospital-	1. General self-efficacy<br><br><br><br><br><br><br><br><br>2. Quality of life: EQ-5D<br><br>3. Physical activity: Grimby scale<br><br>4.Return to work	1. Patients in the intervention group reported significantly higher general self-efficacy than those in the control group six months after intervention start-up.<br><br>2-4. No significant differences between the two groups.

For peer review only - http://bmjopen.bmj.com/site/about/guidelines.xhtml

Study Number	Author & Year/ Country	Aim Design Theoretical model	Sample	Intervention(s)	Outcomes/measures and follow-up period	Results
				based outpatient care and primary care.		
12c	Fors et al (2017) (17)<br><br>Sweden	To assess the long-term effect of PCC in patients with acute coronary syndrome (ACS).<br><br>RCT.<br><br>Gothenburg PCC framework	N=199 with diagnosis of ACS and aged <75 years<br><br>n=94 intervention, Mean age (SD) 60.5 (9.3)<br>n=105 control, Mean age (SD) 61.3 (8.9)	PCC according to the Gothenburg PCC framework containing three routines for guiding PCC process to initiate, integrate and safeguard PCC in clinical practice. The PCC teams were trained through lecturers, workshops, and seminars on how to apply the intervention.<br><br>Comparison group received usual care comprising procedures in line with national guidelines.	Primary outcome:<br>1. Self-efficacy: general self-efficacy scale (GSE)<br><br>Measures completed at one month, two months, six months, and 24 months.	1.The composite score improved in the PCC group compared with the control group at two-year follow-up (18.1% vs 10.5% p=0.127).<br>In the per-protocol analysis, the number of patients improving was significant in favour of the PCC (21.8% vs 10.5%, P=0.039).
12d	Fors (2016)(18)<br><br>Sweden	Evaluating the effects of PCC intervention on self-efficacy after hospitalisations for acute coronary syndrome (ACS).<br><br>RCT.	N=177 patients <75 years hospitalised for ACS<br>n=84 intervention. Mean age 61.0 (SD 9.2)<br>n=93 control.	Provided by a group of health care professionals at the designated hospitals, outpatient clinics, and five primary care centres. Professionals were instructed through lecturers, workshops, seminars on application of PCC through	Patient confidence in managing coronary heart disease: Swedish Cardiac Self-Efficacy Scale (S-CSES). Assessments were conducted at baseline, one month and six months.	PCC improved significantly on the dimension of control symptoms (mean 0.81 vs -0.20; p=0.049) at 1 month. No significant differences were

For peer review only

Study Number	Author & Year/ Country	Aim Design Theoretical model	Sample	Intervention(s)	Outcomes/measures and follow-up period	Results
		Person-centred care after acute coronary syndrome, from hospital to primary care - A randomised controlled trial" Gothenburg PCC framework	Mean age 61.8 (SD 8.8) years.	teams (patient, physician, and registered nurse). Patients were engaged as partners in their care. Patients and professionals created a collaborative PCC plan within 48 hours of recruitment, then reviewed and revised at 48 hour intervals during admission. After discharge follow-up appointments were held at 4 and 8 weeks with further visits scheduled if required. Comparison received usual care following guidelines previously developed including follow up visits with a nurse at 2-3 weeks and a cardiologist at 6 weeks, then afterwards with their primary care physician at 8-10 weeks.		seen at six months (p=0.366). No significant difference between IG and CG in global cardiac self-efficacy at one month (p=0.299) or six months (p=0.577)
12e	Fors et al 2016 (19) Sweden	The aim of this study was to evaluate the effects of person-centred care (PCC) after acute coronary syndrome (ACS) in	As above (Sub study RCT)	As above	The primary endpoint was a composite of changes combining self-reported general self-efficacy with return to work or previous activity level and clinical	In the group of patients without postsecondary education (n=90) the composite score showed a significant improvement in

Study Number	Author & Year/<br><br>Country	Aim<br>Design<br>Theoretical model	Sample	Intervention(s)	Outcomes/measures and follow-up period	Results
		relation to educational level of participants.<br><br>RCT<br><br>Gothenburg PCC framework			outcomes such as re-hospitalisation or death.<br><br>The General Self-Efficacy Scale (GSES) is a 10-item assessed the strength in personal beliefs to cope with and adapt to a variety of daily challenges.<br><br>The Saltin-Grimby Physical Activity Level Scale was used to determine return to previous activity level among those not working. The scale is a self-reported measure of physical activity.<br><br>At 6 months after discharge, each patient was assessed as improved, unchanged, or deteriorated.<br><br>To be classified as improved required	favour of the PCC intervention (n=40) vs. usual care (n=50) at six months (35.0%, n= 14 vs. 16.0%, n = 8; odds ratio (OR) = 2.8, 95% confidence interval (CI): 1.0–7.7, P = 0.041). In patients with postsecondary education (n= 109), a non-significant difference in favour of the PCC intervention (n= 54) vs. usual care (n = 55) was observed in the composite score (13.0%, n = 7 vs 3.6%, n = 2; OR = 3.9, 95% CI: 0.8–19.9, P = 0.097).<br><br>A higher proportion of patients receiving the PCC intervention improved according to the composite

For peer review only

Study Number	Author & Year/ Country	Aim Design Theoretical model	Sample	Intervention(s)	Outcomes/measures and follow-up period	Results
					improvement in the GSES with ≥5 units, return to work or previous activity level (improved from step 1 or at least unchanged from step 2) and no re-hospitalisation or death. A decrease in the GSES with ≥5 units or re-admission for unexpected cardiovascular reasons or death represented a deteriorated condition. Patients were dichotomised into two categories: improved vs. unchanged/deteriorated.	score: 21 of 94 (22%) in the intervention group vs. 10 of 105 (10%) in the controls, p = 0.013. The same outcome applied for the GSES criteria (≥5-point improvement in the GSES): 23 of 94 (24%) vs. 14 of 105 (13%), p = 0.043. A higher proportion of individuals in the intervention group that fulfilled the criteria for GSES also fulfilled the other two criteria included in the composite score: 21 of 23 (91%) vs. 10 of 14 (71%), although the difference was not statistically significant (p = 0.11). This applied to 100% of the patients with low educational

For peer review only

Study Number	Author & Year/ Country	Aim Design Theoretical model	Sample	Intervention(s)	Outcomes/measures and follow-up period	Results
						level that received the PCC intervention which can be compared with the corresponding figures for patients with high education that received the intervention (7 of 9, 78%) (p = 0.06) or to the controls with a low educational level (8 of 11, 73%) (p= 0.04).
12f	Fors et al 2015 (20)<br><br>Sweden	To evaluate if person-centred care can improve self-efficacy and facilitate return to work or prior activity level in patients after an event of acute coronary syndrome<br><br>RCT<br><br>Gothenburg PCC framework	N=199 patients with acute coronary syndrome <75 years.<br><br>n=94 intervention mean age 60.5 (SD 9.3)<br><br>n=105 control 61.3 (SD 8.9)	In the intervention group a person-centred care process was added to treatment as usual, emphasising the patient as a partner in care. Care was co-created in collaboration between patients, physicians, registered nurses and other health care professionals and documented in a health plan. A team-based partnership across three health care levels included transparent knowledge	1. Main outcome measure was a composite score of changes in general self-efficacy ≥ 5 units, return to work or prior activity level and re-hospitalisation or death.<br><br>*Self-efficacy: General Self-Efficacy Scale* (GSE scale) a 10-item self-assessment questionnaire designed to measure a broad and stable sense of personal	1. The composite score showed that more patients (22.3%, n = 21) improved in the intervention group at 6 months compared to the control group (9.5%, n = 10) (odds ratio, 2.7; 95% confidence interval: 1.2–6.2; P = 0.015). The effect was driven by improved self-efficacy ≥ 5 units in the

For peer review only

Study Number	Author & Year/ Country	Aim Design Theoretical model	Sample	Intervention(s)	Outcomes/measures and follow-up period	Results
				about the disease and medical state to achieve agreed goals during recovery<br><br>All gPCC professionals had received training in the theory and practice of gPCC through lectures, seminars and workshops and were given practice in how to formulate and execute gPCC plans. Training emphasised the importance of seeing the patient as a person with needs as well as resources and of a person-centred dialogue as a basis for engaging patients as actively involved partners in their own care.	competence to deal effectively with a variety of stressful situations<br><br>2. Physical activity: Saltin Grimby Physical Activity Level Scale (SGPALS) is a validated measure of self-reported physical activity.<br><br>Questionnaires were completed by patients at baseline in hospital and at four, eight and 24 weeks per post.	intervention group. Overall general self-efficacy improved significantly more in the intervention group compared with the control group (P = 0.026).<br><br>2. There was no difference between groups on re-hospitalisation or death, return to work or prior activity level.
12g	Wolf et al 2016 (21)<br><br>Sweden	To investigate the effect of an eHealth diary and symptom-tracking tool in combination with PCC for patients with acute coronary syndrome (ACS).	This was a sub-study of a RCT investigating the effects of PCC in patients hospitalized with ACS.	Patients in the intervention arm could choose to use a Web-based or mobile-based eHealth tool, or both, for at least 2 months after hospital discharge.	The primary end point was a composite score of changes in general self-efficacy: General Self-Efficacy Scale (GSES) using the Swedish version.	In the intervention arm, n=37 (39%) used the eHealth tool at least once after the index hospitalization. Most of these (24/37, 65%) used the

For peer review only

Study Number	Author & Year/ Country	Aim Design Theoretical model	Sample	Intervention(s)	Outcomes/measures and follow-up period	Results
			N=199 patients with ACS aged <75 years were randomly assigned to a PCC intervention (n=94) or standard treatment (control group, n=105)<br><br>Group 1: Person-centred care plus eHealth (n=37)<br><br>Group 2: Person-centred care only (n=57)<br><br>Group 3: Control (n=105)	A registered nurse at the hospital asked all of the patients in the eHealth group if they were interested in using the eHealth tool. Patients had the opportunity to borrow a mobile phone with the eHealth app preinstalled or to download it for use on their own mobile phone. An introductory demonstration, which required the patient to test the eHealth tools, was provided by a registered nurse who was familiar with the study so that patients could start using the tools freely during their hospital stay. Patients also had access to a video demonstration online for further information. The patients themselves decided on the frequency and patterns of use of the eHealth tools. Access to the webpage had no time restriction.		mobile app and not the Web-based app as the primary source of daily self-rating input. Patients used the eHealth tool a mean of 38 times during the first 8 weeks (range 1–118, SD 33) and 64 times over a 6-month period. Patients who used the eHealth tool in combination with the PCC intervention had a 4-fold improvement in the primary end point compared with the control group (odds ratio 4.0, 95% CI 1.5–10.5; P=.005). This improvement was driven by a significant increase in general self-efficacy compared with the control group (P=.011).

For peer review only

Study Number	Author & Year/ Country	Aim Design Theoretical model	Sample	Intervention(s)	Outcomes/measures and follow-up period	Results
				Patients in the control group were managed according to standard rehabilitation, which followed guideline-directed care that was compliant with Swedish standards.	Return to work or prior activity level, and rehospitalization or death 6 months after discharge.<br><br>Patients filled out the GSES instrument at baseline at the hospital, and at 4 weeks, 8 weeks, and 6 months.	Patients in the PCC group who did not use the eHealth tool (n=57) showed a nonsignificant composite score improvement compared with those in the control group (n=105) (odds ratio 2.0, 95% CI 0.8–5.2; P=.14).<br><br>There were 6 events in the PCC + eHealth group (1 death, 5 readmissions), 12 events in the PCC group without eHealth (3 deaths, 9 readmissions), and 16 events in the control group (2 deaths, 14 readmissions). The proportion of patients who returned to work was similar between

For peer review only

Study Number	Author & Year/ Country	Aim Design Theoretical model	Sample	Intervention(s)	Outcomes/measures and follow-up period	Results
						groups at 6 months (PCC + eHealth 30/34, 88%; PCC no eHealth 47/53, 89%; control 89/98, 91%).
13	Zakrisson (2019) (22)<br><br>Sweden	To test a self-management intervention in primary health care (PHC) for patients with COPD or chronic heart failure (CHF) on self-efficacy, symptoms, functioning and health<br><br>Multi-centre RCT<br><br>Based on Bandura's theory of self-efficacy	N=150 patients with COPD or CHF from 9 PHC n=73 intervention group, mean age 74.0 (SD 7.4) n=77 control group, mean age 71.4 (SD 8.9)	Intervention: Delivered by a physiotherapist and a nurse who had undertaken a 2-day training programme. Groups of 3 COPD and 3 CHF patients and their relatives attended six 90-minute meetings every other week for a total of 6 meetings. Patients created individual action plans based on personal problems and goal setting discussions. Patients were supported to practice skills and gain knowledge for better self-management and behavioural changes. Further meetings at 6 and 9 months to study long term effects.<br><br>Control: details not provided	1. Self-efficacy: perceived self-efficacy for fatigue self-management scale (PSEFSM)<br><br>2. Anxiety and depression: Hospital Anxiety and Depression Scale (HADS)<br><br>3. Dyspnoea: modified Medical Research Council dyspnoea scale (mMRC) and New York Heart Association scale (NYHA)<br><br>4. Fatigue Impact Scale (FIS)<br><br>5. Canadian Occupational	1. No significant change of score at 3 or 12 months for either group.<br><br>2. No significant change of score at 3 or 12 months for either group.<br><br>3. No significant change of score at 3 or 12 months for either group.<br><br>4. No significant change of score at 3 or 12 months for either group.<br><br>5. Significant improvement in IG

For peer review only

Study Number	Author & Year/ Country	Aim Design Theoretical model	Sample	Intervention(s)	Outcomes/measures and follow-up period	Results
					Performance Measure (COPM)<br><br>6. Six-minute walking distance test (6MWD)<br><br>7. 36 Item Short Form Survey (SF-36)<br>COPM assessed at baseline and 3 months. All other measures collected at baseline, 3 months and 1 year.	group from baseline to 3 months (performance scores 4.7 and 5.3, p=0.04, satisfaction scores 4.5 and 5.1, p=0.03)<br><br>6. No significant change of score at 3 or 12 months for either group<br><br>7. Statistically significant improvement on social function subscale for IG between baseline and 1 year for IG (-8.3 vs 2.6, p=0.005). All other subscales no significant change.

For peer review only

Study Number	Author & Year/ Country	Aim Design Theoretical model	Sample	Intervention(s)	Outcomes/measures and follow-up period	Results
14	Arian (2018) (23)<br><br>Iran	To investigate the effect of a holistic care programme (HCP) on the reduction of iron overload in patients with beta-thalassaemia major<br><br>RCT	N=90 patients with beta-thalassaemia major referred to a large thalassaemia centre in Iran<br>n=45 intervention, mean age 25.58 (SD 3.92)<br>n=45 control, mean age 23.91 (SD 5.03)	Intervention: Patients attended the HCP over 8 weeks. This comprised individual counselling for four 45-60 min sessions, group training for four 60-90 min sessions and rehabilitation for 20 sessions<br><br>Control: Routine care at the clinic for 8 weeks	Primary outcomes:<br>1. Change in serum ferritin at three months (mg/L)<br><br>2. Change in iron level at three months (micrograms/dL)<br><br>Secondary outcomes:<br>3. Change in serum ferritin 1 year and 2 years post intervention<br><br>4. Total iron binding capacity at three months<br><br>5. Six-minute walk test (6MWT) at three months (metres)	1. Significantly greater reduction in IG (mean difference between groups -1180.84mg/L, p=0.001)<br><br>2.Significantly greater reduction in IG (mean difference -65.555micrograms/dL, p=0.002)<br><br>3. No significant difference comparing IG and CG (p=0.07). Significant reduction within IG at 1 year (p=0.001) and 2 years (p=0.001).<br><br>4. Not significant (mean difference 8.33, p=0.724)<br><br>5. Significant improvement in IG compared to CG

For peer review only

Study Number	Author & Year/ Country	Aim Design Theoretical model	Sample	Intervention(s)	Outcomes/measures and follow-up period	Results
					6. Haemoglobin (Hb) at three months	(mean difference 99.95m, p=0.001)<br><br>6. No significant difference (mean difference -0.27, p=0.425)
15	Eggers et al 2018 (24)<br><br>Germany	To assess whether a community-based, open-label, integrated approach improves QoL in PD patients.<br><br>RCT	N=150 Intervention group (IG), mean age (SD) 69.8 (8.4)<br><br>and 150 Control group (CG), mean age (SD) 69.9 (7.8)	The interventional group (IG) received an individually tailored therapy plan and additional home visits.<br><br>Patients randomly assigned to a control group (CG), received standard German neurological treatment	Primary outcome<br>1. QoL: compared the differential change of Parkinson's Disease Questionnaire (PDQ-39) from baseline to 6-month follow-up between CG and IG.<br><br>2. Mood: Beck Depression Inventory (BDI-2)<br><br>3. Motor: (United Parkinson's Disease Rating scale, Part III, UPDRS-III)	1. PDQ-39 significantly improved in the IG compared to the CG over the 6-month period The mean group difference as a change from baseline over 6 months was 2.20 points (95% CI − 4.4 to − 0.1), p = 0.044.<br><br>2. No significant differences<br><br>3. For motor symptoms, there was a significant reduction in

For peer review only

**Study Number**	**Author & Year/ Country**	**Aim Design Theoretical model**	**Sample**	**Intervention(s)**	**Outcomes/measures and follow-up period**	**Results**
					4. Non-motor functioning: Nonmotor Symptom Score, NMS-Score<br><br>5. Cognition: Parkinson	UPDRS part III over the first 3 months in the IG (p < 0.001), and a significant between-group difference (p = 0.003). Over the 6-month period, UPDRS-III significantly improved in the IG compared to the CG (p ≤ 0.001). The mean group difference as a change from baseline over 6 months was 3.3 points (95% CI − 4.9 to − 1.7; p<0.001).<br><br>4. The scores of the PD-NMS improved after 6 months in favour of the IG (mean change 11.3, 95% CI − 17.1 to − 5.5; p<0.001).

For peer review only

Study Number	Author & Year/<br><br>Country	Aim<br>Design<br>Theoretical model	Sample	Intervention(s)	Outcomes/measures and follow-up period	Results
					Neuropsychometric Dementia Assessment, (PANDA)<br><br>Data collected at baseline, three and six months.	5. No significant differences
16	Fors et al (2018) (25)<br><br>Sweden	To evaluate the effects of person-centred support via telephone in two chronically ill patient<br>groups, chronic obstructive pulmonary disease (COPD) and/or chronic heart failure (CHF).<br><br>RCT<br><br>Person-centred care according to the framework by the Gothenburg Centre for Person-Centred<br>Care (GPCC)	N=221 patients ≥50 years with COPD and/or CHF<br><br>n=103 intervention Mean age (SD) 78.3 (9.5)<br><br>n=118 control Mean age (SD) 76.9 (8.3)	Patients in the intervention group were telephoned one to four weeks after discharge by a registered nurse initially to co-create a person-centred health plan with the patient and subsequently to discuss and evaluate the<br>plan.<br><br>Nurse's initially received extensive training<br>in person-centred communication and a two day dedicated education about CHF and COPD.<br><br><br>Patients in the control care group received usual care and were managed using existing guidelines for the	1. compost score in general self-efficacy: General Self-Efficacy (GSE)	1. No significant differences between the two groups (57.6%, n = 68 vs. 46.6%, n = 48; OR = 1.6, 95% CI: 0.9±2.7; P = 0.102).<br><br>Significantly more patients in the control group had deteriorated in self-efficacy<br>(GSE scores ≥5 units) than in the intervention group at three months (23.7%, n = 28 vs. 11.7%, n = 12; OR = 2.4, 95% CI: 1.1±4.9; P = 0.022) and at six months follow-up (22.9%, n

For peer review only

Study Number	Author & Year/ Country	Aim Design Theoretical model	Sample	Intervention(s)	Outcomes/measures and follow-up period	Results
				diagnosis and treatment of acute and chronic heart failure.	2. Re-hospitalization and death<br><br>Each patient classified as deteriorated, improved or unchanged: Deteriorated: if GSE had decreased by ≥5 units	= 27 vs. 9.7%, n = 10; OR = 2.8, 95% CI: 1.3±6.0; P = 0.011).<br><br>Improvement in GSE was significantly greater in favour of the intervention group at both three months (0.7 (mean) ± 5.8 (SD); n = 79 vs. -2.2 (mean) ± 6.1 (SD); n = 89; P = 0.010) and six months (0.9 (mean) ± 6.4 (SD); n = 69 vs. -2.0 (mean) ± 6.8 (SD); n = 85; P = 0.006<br><br>2. There were 49 clinical events (14 deaths, 35 re-admissions) in the control group and 41 in the intervention group (9 deaths, 32 re-admissions).

For peer review only

Study Number	Author & Year/<br><br>Country	Aim<br>Design<br>Theoretical model	Sample	Intervention(s)	Outcomes/measures and follow-up period	Results
					OR re-admitted to hospital for unscheduled reasons related to COPD and/or CHF OR had died;<br><br>-Improved: if GSE had increased by≥5 units AND the patient had not been hospitalized for unscheduled reasons related to COPD and/or CHF AND not died.<br>-Unchanged: neither deteriorated nor improved according to the above criteria.<br><br>GSE completed at baseline, three and at six months.	Per-protocol analysis (n = 202) of the composite score showed that more patients deteriorated in the control group than in the intervention group (57.6%, n = 68 vs. 42.9%, n = 36; OR = 1.8, 95% CI 1.0±3.2; P = 0.039).
17	Reed et al (2018) (26)<br><br>Australia	To determine whether a clinician-led chronic disease self-management support (CDSMS) program improves the overall self-rated health level of older Australians	N=254 patients over 60 years with at least 2 chronic conditions from 5 general practices<br>n=127 intervention, of which 48% 60-75	Intervention: CDSMS program which uses a set of tools and structured process that enables clinicians and patients to collaboratively assess self-management behaviour, identify problems, set goals	Primary outcome measure:<br>1. Self-rated health measured with 5-point likert scale	1.IG more likely to report better health than CG (OR 2.5, p=0.023) at 6 months. Most participants in both IG and CG reported no change to self-reported health from

Study Number	Author & Year/ Country	Aim Design Theoretical model	Sample	Intervention(s)	Outcomes/measures and follow-up period	Results
		with multiple chronic health conditions<br><br>RCT	years, 36% 76-85 years and 16% >85<br>n=127 control, of which 46% 60-75 years, 40% 76-85 years and 14% >85 years	and develop individual care plans.<br><br>Control: Semi-structured positive attention program. Participants receive information relevant to their condition and scheduled contact with their clinician who was instructed to provide positive attention. All participants received 3 home visits and four follow up phone calls over 6 months from a clinician.	Secondary outcome measures:<br>2. Health status<br><br>3. Health behaviours<br><br>4. Self-efficacy<br><br>5. Health Education Impact Questionnaire (heiQ)<br><br>6. Health care utilisation Assessed at baseline and 6 months.	baseline to 6 months (57% IG and 69% CG). Improved health from baseline to 6 months reported in 34% of IG and 19% CG.<br><br>Secondary outcomes: 2-6 No statistically significant between group differences for any outcome

For peer review only

Study Number	Author & Year/ Country	Aim Design Theoretical model	Sample	Intervention(s)	Outcomes/measures and follow-up period	Results
18	Schäfer et al. (2018) (27)<br><br>Germany	To determine if patient-centred communication leads to a reduction of the number of medications taken without reducing health-related quality of life<br>Two-arm cluster-randomised controlled trial	N=604 patients aged 65-84 with at least three chronic conditions recruited from 55 primary care practices<br>n=299 Intervention group, mean age 73.3 (SD 4.8)<br>n=305 control group, mean age 73.5 (SD 5.0)	Intervention: Three 30-minute PC talks with a GP over 12 months to identify treatment targets and priorities of the patient, review of all medications and discuss goal attainment and future treatment targets<br><br>Control: care as usual (details not provided)	Primary outcomes:<br>1. Change in number of medications taken by the patient<br><br>2. Health related quality of life: EQ-5D<br><br>Secondary outcomes:<br><br>3. Patient satisfaction<br><br>4. Patient empowerment<br><br>5. GP's knowledge about medication taken by the patient<br><br>6. Healthcare use	1. No statistically significant difference between IG and CG for change in number of medications (p=0.43)<br><br>2. No significant difference between groups (p=0.34)<br><br>3. No effect<br><br>4. No effect<br><br>5. No effect (p=0.772)<br><br>6. IG had greater contact with GPs than CG (p=0.010) but fewer days in hospital (p=0.006) and fewer attendances at

Study Number	Author & Year/ Country	Aim Design Theoretical model	Sample	Intervention(s)	Outcomes/measures and follow-up period	Results
						physical, occupational or speech therapy units (p=0.044)
19	Thom et al 2018 (28)<br><br>USA	To determine the benefit of health coaching for patients with moderate to severe COPD relative to usual care.<br><br>RCT	N=192 COPD patients: n=100 intervention, mean age (SD) 60.7 (8.0).and n= 92 control mean age (SD) 61.9 (7.2).	Patients randomized to the health coaching arm received health coaching for 9 months. Each health coach worked with a total of 50 patients with a maximum caseload of 30 patients at any given time. Health coaches were expected to complete an initial visit within 2–3 weeks of enrollment; to meet in person with the patient at least three additional times over the course of the study; and to have a phone check-in call at least every 3 weeks, including within 2 weeks after each medical visit (minimum of 13 phone check-ins over 9 mo). In-person visits could be at the	Primary outcomes:<br><br>1. COPD quality of life: Chronic Respiratory Disease Questionnaire (CRQ-SF)<br><br>2.dyspnoea: CRQ-SF dyspnoea subscale score<br><br>3. Number of COPD exacerbations: a standardized 6-minute walk test<br><br>4. Self-efficacy for COPD management	1-9 There were no significant differences between study arms for any of the primary outcomes or for the secondary outcomes

For peer review only

Study Number	Author & Year/ Country	Aim Design Theoretical model	Sample	Intervention(s)	Outcomes/measures and follow-up period	Results
				clinic, at the patient's home, or at a public location that afforded sufficient privacy. Additional contacts were guided by patient needs and preferences. Coaches were also expected to conduct at least one in-depth consultation with the study pulmonary nurse practitioner specialist and to attend medical visits between the patient and their PCP when possible. Health coaching focused on helping patients identify and achieve self-care goals for their COPD using techniques from motivational interviewing and adult learning models. Specific content included COPD education, action planning for exacerbations, teaching proper inhaler use, and facilitating consultation with a pulmonary nurse practitioner specialist.<br><br>Patients randomised to usual care continued to	5. COPD symptoms and functional capacity: COPD Assessment Test<br><br>6. Lung function: spirometry as the percent predicted FEV_1,<br><br>7. Current smoking status: defined as any self-reported cigarette use in the past 30 days,<br><br>8. Number of bed days owing to respiratory problems in the past 4 weeks.<br><br>9. Knowledge of COPD: the percentage of correct responses to four questions developed for the present study.<br><br>10. Patient-reported quality of care: Patient Assessment of Chronic Illness Care	10: Statistically significant differences between coaching and usual care (0.07 to 0.68 p=0.02).

For peer review only

Study Number	Author & Year/ Country	Aim Design Theoretical model	Sample	Intervention(s)	Outcomes/measures and follow-up period	Results
				have visits with their PCP over the course of the 9-month period. They received any resources their provider and their clinic offered as part of standard care, including access to COPD educators, respiratory therapists, COPD education classes, pulmonary rehabilitation, smoking cessation classes, and pulmonary specialist referrals by the primary care clinician.	Outcomes at baseline, 3, 6, and 9 months.	
20	Armstrong et al (2017) (29)<br><br>Canada	To determine whether follow-up care delivered via a mobile app can be used to avert in-person follow-up care visits compared with conventional, in-person follow-up care in the first 30 days following ambulatory surgery<br><br>RCT	N=65 women undergoing elective breast reconstruction surgery<br>n=32 intervention, mean age 50.3 (SD12.3)<br>n=33 control, mean age 45.1 (SD 14.1)	Intervention: Planned clinic follow up replaced with daily use of QoC Health Inc mobile app. Allows users to submit photographs and responses to validated quality of recovery questionnaire and visual analogue scale for first 30 days post operatively. Surgeons follow patient reports on a web portal.	Primary outcome:<br>1. Total number of follow-up visits associated with the surgery at 30 days post-op.<br><br>Secondary outcomes:<br><br>2. Total number of telephone calls and emails to the healthcare team associated with the surgery at 30 days post-op.	1. IG had fewer follow up visits than CG (mean 0.66 vs 1.64) IG 0.4 times less likely to attend in person (p<0.001)<br><br>2. No significant difference between IG and CG in telephone calls (mean 0.31 vs 0.3, IRR 1.03, p=0.95).

For peer review only

Study Number	Author & Year/ Country	Aim Design Theoretical model	Sample	Intervention(s)	Outcomes/measures and follow-up period	Results
				Control: planned clinic follow up at 1 and 4 weeks post operatively		IG sent more emails than CG (mean 0.65 vs 0.15, IRR 4.13, p=0.05)
					3.Patient reported satisfaction and convenience scores: 5 point Likert scale	3. No significant difference between groups in satisfaction scores (IRR 0.95, p=0.7). IG had higher convenience scores than CG (IRR 1.39, p=0.08)
					4. Post-operative complications: adverse events attributed to the surgery requiring a medical or surgical intervention<br>All outcomes measured at 30 days.	4. No difference in rates of complications between groups (p=0.42).

For peer review only

Study Number	Author & Year/<br><br>Country	Aim<br>Design<br>Theoretical model	Sample	Intervention(s)	Outcomes/measures and follow-up period	Results
21	Feldthusen et al 2017 (30)	To examine effects of person-centered physical therapy on fatigue and related variables in persons with rheumatoid arthritis (RA).<br><br>RCT<br><br>Gothenburg	Rheumatoid arthritis patients recruited at outpatient rheumatology clinic<br><br>(N=70):<br>intervention group (n=36) mean age 54.2 (SD 8.5) and control group (n=34) mean age 52.7 (SD 10.9).	Each participant in the intervention group participated in the 12-week intervention of person-centered physical therapy. The goal of the intervention was, in partnership between participant and physical therapist, to devise a mutually agreed self-care plan that guided the participant in managing his or her fatigue and to effectively do so over time. The same physical therapist, experienced and specialized in RA management and person-centered care, conducted the intervention.<br>The intervention was initiated with an individual person-centered meeting. A self-care plan was jointly developed and focused on tailoring health-enhancing physical activity and balancing life activities	1. Primary outcome was general fatigue (visual analog scale).<br><br>Secondary outcomes:<br>2. Multidimensional fatigue (Bristol Rheumatoid Arthritis Fatigue Multi-Dimensional Questionnaire)<br><br>3. Fatigue-related variables (ie, disease, health, function).<br><br>Data collected at baseline, three and six months.	1.General fatigue improved more in the intervention group than the reference group (P=.042). Improvement in median general fatigue reached minimal clinically important differences between and within groups at post test and follow-up.<br><br>2-3 Improvement was also observed for anxiety (P=.0099), and trends toward improvements were observed for most multidimensional aspects of fatigue (P=.023-.048), leg strength/endurance (P=.024), and physical activity (P=.023). Compared

Study Number	Author & Year/ Country	Aim Design Theoretical model	Sample	Intervention(s)	Outcomes/measures and follow-up period	Results
				The reference group continued with regular activities; both groups received usual health care		with the control group at follow-up, the intervention group improvement was observed for leg strength/endurance (P=001), and the trends toward improvements persisted for physical (P=041) and living related (P=031) aspects of fatigue, physical activity (P=019), anxiety (P=015), self-rated health (P=.010), and self-efficacy (P=046).
22a	Hansson et al 2017 (31)<br><br>Sweden	To compare a person-centred care intervention in terms of health-related quality of life, disease-specific symptoms or problems, with traditional care as a control group for patients with head and neck cancer.	N=96 patients with head and neck cancer (HNC) attending oncology care<br><br>n=54 intervention mean age 61 (SD 7.8)	Patients attended meetings with the intervention nurse, oncology specialist. The first meeting included a description of the study as well as information needed about the health-care plan. The plan was designed and developed according to a basic model from Gothenburg PCC	Health related Quality of Life (HRQoL): European Organization for Research and Treatment of Cancer (EORTC) QLQ-C30 and the EORTC QLQ-35 version 3.0.	HRQoL was nonsignificant in all instruments. gPCC-group tended, from the 10th week, to be better than those in the control group (CG) and were, from the 18th week, statistically significantly better in

For peer review only

Study Number	Author & Year/<br><br>Country	Aim<br>Design<br>Theoretical model	Sample	Intervention(s)	Outcomes/measures and follow-up period	Results
		RCT<br><br>Gothenburg PCC	n=42 control mean age 62 (SD 10.9)	(gPCC) and further adapted to suit patients with HNC and scheduled by the nurse and patient together. The health-care plan comprised self-management goals that were formed in partnership between the patient and the nurse. Each patient was encouraged to reflect on their self-management goals, how to reach them, and to anticipate barriers; and to refine the plan. The health plan includes both short- and long-term goals for the patient along with the actions needed to reach each goal.<br>The plan is a "living" document specific to each patient, in which the goals and actions are tracked and revised over time. The patient was also given a direct telephone number to reach the nurse specialist if they had any questions about anything relating to their treatment and	Data collected at baseline, weeks 4, 10, 18 and 52.	the gPCC-group in terms of HNC-specific problems (QLQ-35), swallowing (p = 0.014), social eating (p = 0.048) and feeling ill (p = 0.021).

For peer review only

Study Number	Author & Year/ Country	Aim Design Theoretical model	Sample	Intervention(s)	Outcomes/measures and follow-up period	Results
				wellbeing. The nurse documented the health-care plan in the medical record.<br><br>Patients randomized to the control group received usual care and return visits were scheduled according to the treatment procedure based on the Regional care program for patients with HNC which included post-treatment follow up visits to an oncologist at 6–8 weeks and from then on every third month for 2 years.		
22b	Gyllensten et al 2019	The aim was to examine the cost-effectiveness, including healthcare and productivity costs, of a person-centred care intervention versus standard medical care among patients with Head and Neck Care.	As above	As above	Health-related quality of life: EuroQol (Group's five-dimension health state questionnaire (EQ-5D™),	No significant differences<br><br>(The average total cost was Euro (EUR) 55,544 (95% confidence interval: EUR 48,474–62,614) in the intervention group

For peer review only

Study Number	Author & Year/ Country	Aim Design Theoretical model	Sample	Intervention(s)	Outcomes/measures and follow-up period	Results
		RCT<br><br>Gothenburg PCC			At baseline, 4 weeks, 10 weeks, 18 weeks, and 52 weeks.	and EUR 57,443 (EUR 48,607–66,279) among controls, with similar health-related quality of life)
23	Ko et al (2017) (32)<br>Hong Kong	To evaluate whether comprehensive care programme with multidisciplinary input will decrease hospital readmissions and length of hospital stay for patients with COPD<br><br>RCT.	N=180 COPD patients admitted with an acute exacerbation.<br>n=90 intervention. Mean age 74.9 (SD=7.9) years,<br><br>n=90 control. Mean age 74.6 (SD=8.6).	Individualised education sessions including anatomy and physiology, pathophysiology of COPD, smoking cessation, techniques of using medication, management of dyspnoea, self-management of exacerbations, coping, relaxation techniques, social and community support.<br>Patients were provided with telephone number to call and seek advice from respiratory nurse during office hours.	Primary Outcome:<br>1. Hospital readmission rate at one year.<br><br>Secondary outcomes:<br>2. Length of stay (LOS)<br><br>3. Dyspnoea: Modified Medical Research	1. At 12 months relative risk of readmission was 0.668, p=0.047 for the intervention group compared with the control group.<br><br>2. at 12 months IG had a shorter LOS 4.59 vs 8.86, p<0.001<br><br>3. IG had greater improvement on

For peer review only

Study Number	Author & Year/ Country	Aim Design Theoretical model	Sample	Intervention(s)	Outcomes/measures and follow-up period	Results
				Subsequently patients received three monthly telephone calls from respiratory nurse for one year to assess their condition and answer queries.<br><br>Comparison group received usual care, the attending physician determined the patient's medication and follow-up as normal practice.	Council Dyspnoea Scale (MMRC)<br><br>4. QoL: St George's Respiratory Questionnaire.<br><br>5. Lung function FEV_1/FVC ratio<br><br>6. Exercise capacity: 6 minute walk test<br><br>7. Mortality	MMRC -0.1 vs 0.2, p=0.003<br><br>4. SGRQ: Improvement for IG at 12 months, -6.9 vs -0.1, p=0.003<br><br>5. No significant difference between groups in change in lung function at 12 months (p=0.653)<br><br>6. No significant difference between groups in change in exercise capacity at 12 months (-10m vs -22.5m, p=0.233)<br><br>7. Ten patients in IG and 12 in CG had died at 12 months.
25	Low et al (2017) (33)<br><br>Singapore	Evaluate the effectiveness of an integrated practice unit and modified virtual ward model in reducing readmission rates in	N=840 patients with one or more unscheduled readmissions in last 90 days and at high risk of	Intervention: Hospital care transferred to Integrated Practice Unit MDT on randomisation. Intensive discharge planning including identifying and	Primary outcome:<br>1. Unplanned readmissions within 30 days of discharge	Primary outcome:<br>1. Readmission at 30 days was lower in the intervention group than the

For peer review only

Study Number	Author & Year/ Country	Aim Design Theoretical model	Sample	Intervention(s)	Outcomes/measures and follow-up period	Results
		patients at highest risk of readmission.<br><br>RCT	readmission (LACE score >/=10)<br>n=420 intervention group, mean age 70.5 (SD 13.5)<br>n=420 control group, mean age 70.3 (SD 13.7)	addressing risk factors for readmission. All patients provided with individualised care plan on discharge. Phone call from nurse case manager within 72 hours of discharge and home assessment within 1 week plus review at Virtual Ward MDT.<br><br>Control: Standard hospital care	Secondary outcomes:<br>2. Unplanned readmissions within 90 and 180 days of discharge (visits/patient/month)<br><br>3. Emergency department attendance rate within 30, 90 and 180 days of discharge (visits/patient/month).<br><br>4. Probability of death up to 180 days	control group (0.25 vs 0.38, p=0.001)<br><br>2. Readmissions at 90 (0.67 vs 0.90, p=0.001) and 180 (1.05 vs 1.46, p=<0.001) days were lower in the intervention group than the control group.<br><br>3. ED visits were lower in the intervention group than the control group at 30 (0.26 vs 0.43, p=<0.001), 90 (0.66 vs 0.92, p=0.001) and 180 (1.14 vs 1.60, p<0.001) days.<br><br>4. 28% reduction in mortality in intervention group compared to control (HR 0.72, p<0.001).

For peer review only

Study Number	Author & Year/<br><br>Country	Aim<br>Design<br>Theoretical model	Sample	Intervention(s)	Outcomes/measures and follow-up period	Results
25	Wichit et al (2017) (34)<br><br>Thailand	To evaluate a theoretically driven family-oriented intervention to improve self-efficacy, self-management, glycaemic control and quality of life in T2D<br><br>RCT.<br><br>Bandura's self-efficacy theory	N=140 T2D patients.<br><br>n=70 experimental group, mean age 61.3 (SD=11.6) years;<br>n=70 control group, mean age 55.5 (SD=10.5) years.	Family-oriented programme (patients/family dyads) consisting of education classes, group discussions, home visit, and telephone follow-up. Participants learned specialised skills such as meal planning, physical activities, managing complications. Education sessions were delivered at baseline, week 5 and week 9.<br><br>Control received usual care consisting of blood sugar testing, physical examinations and medication follow-up	Primary outcome<br>1. Type 2 Diabetes (T2D) self-management: Summary of Diabetes Self-Care Activities Scale (SDSCA)<br><br>Secondary outcomes:<br><br>2. T2D self-efficacy: Diabetes Management Self-Efficacy Scale (DMSES) and Perceived Therapeutic Self-Efficacy Scale (PTES)	1. At week 5 SDSCA increased from 80.9 to 96.5 in the intervention and decreased from 80.5 to 80.2 in the control, the results were significant between the two groups (p<0.001). At week 13 SDSCA was 1.2.8 in the intervention and 80.4 in the control (p<0.001).<br><br>2. At week 5 DMSES increased from 55.6 to 69.8 in the intervention, but decreased from 58.7 to 58.2 in the control (p<0.001)<br>At week 13 DMSES further increased to 76.0 in the intervention and slightly increased in

Study Number	Author & Year/<br><br>Country	Aim<br>Design<br>Theoretical model	Sample	Intervention(s)	Outcomes/measures and follow-up period	Results
					3. Quality of life: Thai Version short-form Health Survey (SF-12)	the control to 60.7 (p<0.001).<br>At week 5 PTES increased from 32.4 in the intervention to 37.9 but decreased from 34.8 to 33.7 in the control group (p<0.001). at week 13 PTES increased in both groups to 40.8 in the intervention and 35.3 in the control group (p<0.001).<br><br>3. At week 5, Physical aspect of QoL increased in both groups from 46.7 to 50.0 in the intervention and 48.2 to 49.2 in the control (p=0.2), similar pattern occurred at week 13.<br><br>Mental aspect of QoL increased from 54.1 to 56.0 in the intervention group.

For peer review only

Study Number	Author & Year/<br><br>Country	Aim<br>Design<br>Theoretical model	Sample	Intervention(s)	Outcomes/measures and follow-up period	Results
					4. Diabetes Knowledge: Diabetes Knowledge Questionnaire (DKQ)<br><br>5. HbA1c: extracted from patient's health records<br><br>Outcomes conducted at baseline and 3 weeks and 13 weeks (HbA1c	In the control group it remained at 54.3. (p=0.2). At week 13 QoL was 58.4 in the intervention and 54.7 in the control (p<0.001).<br><br>4. At week 5 DKQ was 17.1 from 10.7 in the intervention, while it was 11.7 from 10.6 in the control (p<0.001). At week 13 DKQ was 16.5 in the intervention group and 13.2 in the control group (p<0.001)<br><br>5. At baseline HbA1c was 7.0 in the intervention and 6.3 in the control. At week 13 it was 7.0 in the intervention and 7.3 in the control (p=0.2)

For peer review only

Study Number	Author & Year/ Country	Aim<br>Design<br>Theoretical model	Sample	Intervention(s)	Outcomes/measures and follow-up period	Results
					was assessed at baseline and week 13).	
26a	Larsson et al 2015 (35)<br><br>Sweden	To examine the effects of a progressive resistance exercise program on muscle strength, health status, and current pain intensity in women with Fibromyalgia (FM).<br><br>RCT<br><br>Gothenburg PCC	N=130 women with FM, n=67 resistance exercise, n=63 mean age 50.8 (SD 9.05) relaxation therapy mean age 52 (SD 9.08)	The intervention: The resistance exercise program was performed twice a week for 15 weeks and was supervised by experienced physiotherapists. It was conducted at physiotherapy premises and at a local gym at four different sites in groups comprising five to seven participants to promote interaction between participants and to facilitate physiotherapeutic guidance. The intervention was preceded by an individual introductory meeting.<br>The meeting was commenced with a dialogue between the participant and the physiotherapist about the participant's earlier experiences and thoughts of exercise.	1. The primary outcome was isometric knee-extension force (N) measured with a dynamometer (Steve Strong: Stig Starke HBI, Göteborg, Sweden) using a standard protocol.<br><br>Secondary outcomes were:<br>2. Fibromyalgia impact: the fibromyalgia impact questionnaire (FIQ) a disease-specific self-reported questionnaire that comprises ten subscales of disabilities and symptoms.<br><br>3. Current pain intensity: rated on a plastic 0-100 visual analogue scale	1. Significantly greater improvement (p = 0.010) was found for isometric knee-extension force in favor of the resistance exercise group as compared to the active control group<br><br>2. Significantly greater improvement was observed in health status (FIQ total score) (p = 0.038) in the resistance exercise group compared to the active control group<br><br>3. Significantly greater improvement was observed

Study Number	Author & Year/<br><br>Country	Aim<br>Design<br>Theoretical model	Sample	Intervention(s)	Outcomes/measures and follow-up period	Results
				The meeting also included exercise instructions, testing and adjustment of loads and modifications of specific exercises according to individual conditions and according to self-efficacy principles. The meeting resulted in a written protocol with descriptions of specific exercises and loads, which was used by each participant as an exercise program at each exercise session. The exercise was initiated at low loads, and possibilities for progressions of loads were evaluated every 3−4 weeks in dialogue between the physiotherapist and participant.<br><br>The control group was the relaxation therapy was performed twice a week for 15 weeks and was guided by experienced physiotherapists. It was conducted at physiotherapy premises at four different	with a moveable cursor along a line and anchors at the extremes.<br><br>4. The six-minute walk test (6MWT), a performance-based test that measures total walking distance (m) during a period of 6 minutes	in current pain intensity (VAS) (p = 0.033) in the resistance exercise group compared to the active control group<br><br>4. Significantly greater improvement was observed in the 6MWT (p = 0.003) in the resistance exercise group compared to the active control group

For peer review only

Study Number	Author & Year/ Country	Aim Design Theoretical model	Sample	Intervention(s)	Outcomes/measures and follow-up period	Results
				sites in groups comprising five to eight participants and was preceded by an individual introductory meeting at the premises, which included instructions and allowed for preparations and modifications of practical matter such as positioning and the use of mattresses and pillows to reach a good level of comfort. The relaxation therapy performed a series of mental exercises including relaxation and autosuggestion. The physiotherapist guided the participants through their bodies, during approximately 25 minutes, by focusing their minds on the bodily experience of relaxation and letting the body part in focus rest on the ground. This was repeated for each specific body-part, aiming at feeling as relaxed as possible in		

For peer review only

Study Number	Author & Year/<br><br>Country	Aim<br>Design<br>Theoretical model	Sample	Intervention(s)	Outcomes/measures and follow-up period	Results
				the whole of the body at the end of the session. Participants were invited to share experiences and ask each other and the physiotherapist questions and continued thereafter with the stretching exercises.		
26b	Ericsson et al 2016 (36)	This sub-study aimed to examine the effects of a person-centered progressive resistance exercise program on multiple dimensions of fatigue in women with fibromyalgia (FM), and to investigate predictors of the potential change in fatigue.	As above	as above	Outcomes were:<br><br>1. Five dimensions of fatigue measured with the Multidimensional Fatigue Inventory (MFI-20).	1.A higher improvement was found at the post-treatment examination for change in the resistance exercise group, as compared to change in the active control group in the MFI-20 subscale of physical fatigue (resistance group change –1.7, SD 4.3, controls change 0.0, SD 2.7, p = 0.013), with an effect size of 0.33.

For peer review only

Study Number	Author & Year/ Country	Aim Design Theoretical model	Sample	Intervention(s)	Outcomes/measures and follow-up period	Results
					2. FIQ fatigue (0–100) The VAS for fatigue included in the Fibromyalgia Impact Questionnaire (FIQ) was used as a one-dimensional measure of fatigue.<br><br>3. Pittsburgh Sleep Quality Index (PSQI) (0–21) The PSQI assesses sleep quality and disturbances over a 1-month period.<br><br>4. Pain catastrophizing scale (PCS) (0–52) The	2. The resistance exercise group improved in the FIQ for fatigue over time from baseline to post treatment (mean difference −8.6, SD 21.2, p = 0.002).<br><br>3. The resistance exercise group improved over time in the PSQI subscale for sleep quality (mean difference −0.2, SD 0.8, p = 0.047), while the active control group improved in the PSQI subscale for need of medications to sleep (mean difference 0.3 SD 1.0, p = 0.036)<br><br>4. The resistance exercise group

For peer review only

Study Number	Author & Year/ Country	Aim Design Theoretical model	Sample	Intervention(s)	Outcomes/measures and follow-up period	Results
					PCS assesses pain-related catastrophic thinking.<br><br>5. Hospital Anxiety and Depression Scale (HADS) (0–21)	improved significantly over time in all three PCS subscales and the PCS total score (mean difference in PCS total score −2.7 SD 7.6, p = 0.004). In the active control group there was a tendency towards improvement in two PCS subscales and the PCS total score (p = 0.051–0.056).<br><br>5. No significant changes during the study period were found within any of the groups for HADS anxiety or HADS depression.

For peer review only

Study Number	Author & Year/<br><br>Country	Aim<br>Design<br>Theoretical model	Sample	Intervention(s)	Outcomes/measures and follow-up period	Results
27a	Hansson et al 2016 (37)<br><br>Sweden	To estimate the cost–utility of PCC when compared with conventional care in patients hospitalized for worsening chronic heart failure.<br><br>A controlled before and after design<br><br>Gothenburg PCC framework	N=248 CHF patients<br>n=125 intervention, mean age 77 (SD 11)<br><br>n= 123 control, mean age 80 (SD 9)	Larsson Larsson Larsson	Costs of care:<br>An assessment of health-related quality of life used the EQ-5D 3L instrument at baseline and at three months after discharge to usual care.<br><br>The quality of life weight was then used to calculate QALYs. This measure combines years of life with quality of life so that the QALY, as a result of a treatment, can consist in increasing life expectancy and/or increased quality of life. QALY calculations were made on an individual level, reflecting the change from baseline to three months, assuming a linear increase in quality of life (QoL) between the two measurements.	We found that PCC resulted in lower costs (€863 per patient, p=0.026) and generated marginally more health benefits than conventional care.<br><br>The costs for those who actually received PCC, per protocol (PP) (63%) were significantly (p=0.026) lower than for those in the conventional care group, with an incremental cost-saving of €863. For the first three months, patients in the conventional care group showed decreasing health-related quality of life, with a corresponding improvement in the PCC(PP) group.

For peer review only

Study Number	Author & Year/<br><br>Country	Aim<br>Design<br>Theoretical model	Sample	Intervention(s)	Outcomes/measures and follow-up period	Results
27b	Ulin et al 2016 (38)<br><br>Sweden	To evaluate whether proactive care-planning based on the Gothenburg person-centred care (gPCC) model leads to improved efficiency in discharge procedures compared with usual care in patients hospitalized for worsening chronic heart failure.<br><br>A controlled before and after design<br><br>Gothenburg PCC framework	As above	The gPCC health plan starts with the patient narrative, which includes information regarding everyday life and symptoms prior to and during the worsening of the condition. In addition, the patient's resources are identified, including motivations and goals. The social situation and the possible need for additional support at home after discharge from hospital are also of importance. Finally, within 24–48 hours, all information and facts are summarized and written in the gPCC health plan, which also includes planned investigations, treatment goals and length of stay at hospital.<br><br>Thereafter, the first notification can be sent to the patient's municipal home care service and to the primary healthcare	The first endpoint was the number of days from admission to Step 1, the first notice to the municipality, including the municipal home care service and the primary healthcare service.<br><br>The second endpoint was the number of days from admission to the second notice to the municipal home care service and to the primary healthcare service confirming the discharge planning conference, or Step 2.	During hospitalization, first notifications (Step 1) to the patients' municipal home-care services and/or round-the-clock home nursing care services were more frequent in the per-protocol gPCC group (33.8%) compared with the usual care group (12.1%), but not significant.<br><br>During hospitalization, the number of days from admission to notices to the patients' municipal homecare services and/or round-the-clock home nursing care services for confirmed discharge planning conferences (the second notification

Study Number	Author & Year/ Country	Aim Design Theoretical model	Sample	Intervention(s)	Outcomes/measures and follow-up period	Results
				service, which is Step 1. The patient and healthcare professionals discuss the gPCC health plan and reach an agreement. The gPCC health plan is regularly evaluated (and if necessary, revised) in all aspects of care (such as symptoms, resources, management and treatment) by the patient and the healthcare professionals during the hospitalization. The gPCC health plan forms the basis for the second notice to the municipal home care service and to the primary healthcare service with an accurate and detailed description of the patient's anticipated status (including for example symptoms and resources) at discharge, as well as any anticipated discharge planning conference in the hospital, which is Step 2. The third notice is recorded when the patient is ready for	The third endpoint, Step 3, was the number of days from admission to the notice to the municipality that the patient was ready for discharge from hospital.	or Step 2) was significantly decreased (p=0.03) in the per-protocol gPCC group compared with the usual care group.<br><br>The length of stay in hospital and the time to the third notification (Step 3) to the patients' municipal home-care services and/or round-the-clock home nursing care services were significantly decreased: 6.77 days in the per-protocol gPCC group compared with 9.22 days in the usual care group (p<0.01), and 11 days in the per-protocol gPCC group

For peer review only

Study Number	Author & Year/ Country	Aim Design Theoretical model	Sample	Intervention(s)	Outcomes/measures and follow-up period	Results
				discharge, also in concordance with the gPCC health plan projected number of days of hospitalization, which is Step 3.		compared with 35 days in the usual care group (p=0.01), respectively
27c	Ekman et al (2012) (39)<br><br>Sweden	To evaluate outcomes of PCC in hospitalized patients with chronic heart failure (CHF) with respect to the length of hospital stay (LOS), activities of daily living (ADL), health-related quality of life (HRQL) and 6-month readmission rate<br><br>Controlled before and after design<br><br>Gothenburg PCC	As above	As above	Primary outcome:<br>1. Length of stay (LOS) computed as number of whole inpatient days from admission to discharge<br><br>Secondary outcomes:	1. The mean LOS in the Usual care group was 9.22 days (SD 7.4, median 7, IQR 5, range 2–44 days) compared with 8.22 days (SD 4.4, median 8, IQR 5, range 2–31 days) in the PCC group (P . 0.16). In the PP analysis, LOS was significantly shorter (2.5 days) in the PCC group (6.77 days, SD 3.2, median 6.5, IQR 3, range 2–25; P . 0.01),<br><br>2. Physical functional

For peer review only

Study Number	Author & Year/ Country	Aim Design Theoretical model	Sample	Intervention(s)	Outcomes/measures and follow-up period	Results
					2. Activities of daily living (ADL) using the Katz-ADL index<br><br>3. Quality of life (HRQL) assessed using the Swedish version of the Kansas City Cardiomyopathy Questionnaire (KCCQ)<br><br>Data collected at baseline, three months, and six months.	performance as assessed with the Katz–ADL index was similar at baseline between the two groups in the analysis of all patients as well as in the PP analysis. At discharge, ADL levels were better in the PCC group (all patients, P . 0.07; the PP group, P . 0.04).<br><br>3. There were no differences in the KCCQ Overall Summary Score or the Clinical Summary score after 3 months.

For peer review only

Study Number	Author & Year/ Country	Aim Design Theoretical model	Sample	Intervention(s)	Outcomes/measures and follow-up period	Results
27d	Dudas et al 2012 (40)	To evaluate whether PCC is associated with less self-reported uncertainty in illness compared with usual care in patients hospitalized for worsening chronic heart failure (CHF).<br><br>A controlled before and after design<br><br>Gothenburg PCC framework	As above	As above	The Swedish version of the Cardiovascular Population Scale (CPS) CPS consists of two dimensions:1) ambiguity (10 items), which covers the perception of patients concerning the severity of their illness; and 2) complexity (six items), which covers the perception of patients concerning their dignity, treatment and system of care.	The PCC group had better scores than the usual care group in the CPS domains complexity (M=15.2, SD=4.7 vs. M=16.8, SD=4.7; p=0.020) and ambiguity (M=27.8, SD=6.6 vs. M=29.8, SD=6.9; p=0.041).<br><br>The PCC group reported lower scores in the dimension of ambiguity, which measures patients' self-reported experiences about uncertainty in their illness, in both the ITT analysis and in the PP analysis (M = 28.2 (SD = 6.5) and 27.8 (SD = 6.6), respectively) than the usual care group (M

For peer review only

Study Number	Author & Year/<br><br>Country	Aim<br>Design<br>Theoretical model	Sample	Intervention(s)	Outcomes/measures and follow-up period	Results
						= 29.8 (SD = 6.9)). There was a significant difference in the dimension of ambiguity in the PP analysis between the groups for patients in the PCC group (p = 0.067).

For peer review only

28	Jutterström et al (2016) (41)<br><br>Sweden	To evaluate the effect of a nurse led patient-centered self-management support in T2D with regard to metabolic changes.<br><br>RCT<br><br>Theory of Hernandez	N=182 people aged 40-80 with T2DM<br>n=70 Group Intervention (GI)<br>n=35 Individual Intervention (II)<br>n=36 Internal control group<br><br>n=54 External Control	Ten Diabetes Specialists Nurses (DSNs) from nine health care centres participated in a preparatory workshop of approximately 20 hrs that emphasised the patients understanding of illness. DSNs received a theoretical and practical preparation and motivating patient-centred communication aimed at supporting illness integration and how to strengthen patient's self-efficacy for self-management.<br>In the patient intervention, participants in the GI and II groups were invited to six sessions of 45-90 minutes each over a period of up to six months.<br>In the GI groups, the patients reflected aspects of living with T2D together and DSNs acted as a moderator.<br>The intervention consisted of either discussions in groups or patients or individual conversations with the DSN, depending on the arm of allocation. During the six sessions, the	1. HbA1c<br><br>2. Body mass index<br><br>3. Systolic and diastolic blood pressure	1. HbA1c significantly decreased at 12 months follow-up by 5 mmol/mol in the GI (p<0.001) and 4 mmol/mol (p=0.004)in the individual intervention (II), in the internal control group there was no change (p=0.878), while in the external control group it increased with 2 mmol/mol (p=0.213). The results were significant between intervention groups (GI and II) and external control group.<br><br>2. Body mass index was not significant between groups<br><br>3. Both systolic and diastolic blood pressure were not significant between groups

For peer review only

				participants were free to discuss issues they considered important in relation to their experiences with the disease.<br><br>Control: IC and EC groups received standard care which normally included 1-2 visits per year as per national guidelines.		

For peer review only

Study Number	Author & Year/<br><br>Country	Aim<br>Design<br>Theoretical model	Sample	Intervention(s)	Outcomes/measures and follow-up period	Results
29a	Olsson et al 2016 (42)<br><br>Two papers one study	The study had two aims:<br>(1) to identify vulnerable patients using the general self-efficacy scale (GSES) and the Tampa scale for Kinesiophobia (TSK), and (2) to evaluate if person-centred care including the responses of the instruments made rehabilitation more effective in terms of shortening hospital length of stay.<br><br>A quasi-experimental design	Patients scheduled for total hip arthroplasty (THA), an intervention group (n = 128), mean age 68 and a control group (n = 138), mean age 66.	Intervention group received evidence-based information based on their own prerequisites. Evidence-based guidelines, clinical knowledge and patients' individual prerequisites were combined with forming a partnership with professionals.<br><br>The first step in establishing the partnership was for a RN specialized in surgical care to obtain a narrative from each patient, covering the patient's everyday life, resources, motivation, and goals; patients were also asked to fill out the General Self-efficacy (GSES) and Tampa scale of kinesiophobia (TSK) questionnaires.<br><br>The RN then made a tentative, detailed gPCC health plan based on the narrative, the medical examination, and the self-	The primary endpoint of the study was the number of days spent in the hospital relative to the self-rated GSES and TSK scores. The hospital Length of Stay was compared between the control group and the intervention group for patients scoring ≤ 29 on the GSES and/or ≥ 40 on the TSK. The relation between Length of Stay and American Society of Anesthesiologists" classification system (ASA) category was also studied.<br><br>1. Self-Efficacy: General self-efficacy scale (GSES)<br><br>2. Fear of Movement: Tampa Scale for Kinesiophobia (TSK)<br><br>3.Length of Stay	Significantly shorter stay in intervention group: 5.3 days (SD 2.2) vs control 7 days (SD 5.0); P<0.0005.<br><br>Patients with low GSES in the intervention group had shorter length of stay (LoS) by 1.6 days (95 % CI 0.16–3.15) p=0.03.<br><br>Patients with high TSK in the intervention group had shorter LoS by 2.43 days (95 % CI 0.76–4.12) p= 0.005. For patients who had both, the reduction of LoS was 2.15 days (95 % CI 0.24–4.04) p=0.028.

Study Number	Author & Year/ Country	Aim Design Theoretical model	Sample	Intervention(s)	Outcomes/measures and follow-up period	Results
				reported results of the GSES and TSK surveys. The gPCC health plan specified each patient's short-and long-term goals, resources, special needs, and plan for recovery after discharge. The tentative health care plan was included in the letter provided to the patient at the outpatient clinic appointment 2 weeks before surgery. The health plan was discussed with the patient and finalized when an agreement was reached between the professionals and the patient.<br><br>The patients were helped to familiarise themselves in the situation and to achieve their personal goal by emphasising their personal resources and capabilities documented in the health plan.	4. American Society of Anesthesiologists" classification system (ASA): Patients scheduled for planned surgery commonly belong to one of three categories: (1) healthy, (2) mild systemic disease, or (3) severe systemic disease. The patients in this study were classified by the anaesthesiologist responsible for anaesthetising patients during the surgical procedure.	

For peer review only

Study Number	Author & Year/ Country	Aim Design Theoretical model	Sample	Intervention(s)	Outcomes/measures and follow-up period	Results
				Control group received Standard care consisted of:<br><br>Completing questionnaires about their living circumstances, physical abilities and filled out surveys such as the GSES, TSK. Standardised information including peri-operative routines and postoperative training based on hip replacement patients in general. Patients also got a written booklet containing details from the oral information about pre and postoperative care.		
29b	Olsson et al 2014 (43)	To investigate if person-centred care intervention would improve patients' recovery as measured by Length of stay LoS following hip surgery	As above	As above	1. The primary outcome measure was Length of Stay LoS, calculated as the number of whole inpatient days from admission to discharge.<br><br>2. Secondary outcomes included physical function	1. The mean LoS in the control group was 7 days (SD 5.0) compared to 5.3 days in the gPCC group (SD 2.2) (p <0.0005)<br><br>2. Physical functional performance: At

For peer review only

Study Number	Author & Year/ Country	Aim Design Theoretical model	Sample	Intervention(s)	Outcomes/measures and follow-up period	Results
					at both discharge and 3 months later, measured with Activity of Daily Living (ADL) and Functional Recovery Scale (FRS). ADL was self-assessed by the patients at admission and measured by a nurse at discharge.<br><br>3. Readmission: Any hospital readmission within 3 months was obtained from the patient records.	discharge, 84% in the control group had regained ADL level A compared with 72% in the intervention group, the difference was not significant.<br><br>For FRS: Three months after surgery, 12% in the control group scored under 80% compared with 8.5% in the gPCC group and the difference was not significant.<br><br>3. Readmissions within 3 months were similar between the two groups; two patients in the control group and three in the gPCC group were readmitted and the

For peer review only

Study Number	Author & Year/ Country	Aim Design Theoretical model	Sample	Intervention(s)	Outcomes/measures and follow-up period	Results
						difference was not significant.
30	Or and Tao (2016) Hong (44)<br><br>Hong Kong	Evaluate the effects of a person-centred tablet computer-based self-monitoring system for chronic disease (T2D and/or hypertension).<br><br>RCT	N=63 patients with T2D and/or hypertension n=33 intervention, mean age 69.3 (SD 9.7) n=30 control, mean age 69.7 (SD 10.2)	Tablet computer-based disease self-monitoring system. The system was interactive with 10 inch tablet computer, blood glucose and blood pressure monitor (2 in 1). The system would indicate Vital signs values. Abnormal values were measured in red, normal values in green. The system also had video-based educational materials that allowed patients to learn how to self-manage their chronic conditions, e.g. how to measure glucose, BP, diet, and exercises.<br><br>Comparison group received a 2-in-1 blood glucose and blood pressure monitor for self-monitoring and a logbook for recording the vital signs measured and the dates and times of measurements.	1.Systolic and diastolic blood pressures	1. Significant improvements were seen in systolic blood pressure in the intervention group from baseline to 1 month (-16.7 mm Hg), 2 months (-10.3 mm Hg) and 3 months (-13.0 mm Hg). Non-significant differences were seen in the control group (-2.1 mm Hg) at month one, 6.2 at 2 months, and -5.4 mm Hg at 3 months. The differences were significant between the two groups after 1 month (p<0.001) and month 3 (p=0.043). Similarly significant differences were seen in diastolic pressure in the

For peer review only

Study Number	Author & Year/ Country	Aim Design Theoretical model	Sample	Intervention(s)	Outcomes/measures and follow-up period	Results
						intervention group (-8.0 mm Hg) at 1 month, -6.6 mm Hg at month 2, and -5.7 mm Hg at month 3. Non-significant decline were seen in the control group -0.3 mm Hg at 1 month, -1.9 mm Hg at 2 months, and -2.0 mm Hg at 3 months. The decline in diastolic pressure were significantly greater in the intervention group than control group after 1 (p<0.001) and 2 months (p=0.028).
					2.Fasting blood glucose	2. After 3 months non-significant decline in FBG was seen in the intervention group (-1.0 mmol/dL) and an increase in the control group (0.4 mmol/dL), the trend

For peer review only

Study Number	Author & Year/<br><br>Country	Aim<br>Design<br>Theoretical model	Sample	Intervention(s)	Outcomes/measures and follow-up period	Results
					3. HbA1c<br><br>4. Patient's knowledge of T2D and hypertension: Modified Michigan Diabetes knowledge Scale and the hypertension knowledge questionnaire.<br>Measured at baseline, months 1,2, and 3.	was not statistically different between groups (p=0.407).<br><br>3. HbA1c<br>Both decreased at 3 months -0.2 in the intervention and control groups. No between group differences.<br><br>4. No significant differences on knowledge of hypertension and T2D.
31a	Sahlen et al (2016) (45)<br><br>Sweden	To assess the cost-effectiveness of person-centred care integrated heart failure and palliative home care.<br><br>RCT	N=72 participants with NYHA class III-IV heart failure<br><br>n=36 intervention<br>n=36 control	Person-centred integrated intervention. Structured PCC (partnership between patients/carers and professional caregivers and includes initiating, working on and documenting partnership) with a collaborative approach	1.Quality adjusted life years (QALYS) EQ-5D	1.QALY was 0.569 in the intervention and 0.538 in the control group as baseline. Slight improvement was seen in the intervention (+0.006), but

For peer review only

Study Number	Author & Year/ Country	Aim Design Theoretical model	Sample	Intervention(s)	Outcomes/measures and follow-up period	Results
		Person-centred palliative care model. Six S: self-image, self-determination, social relationships, symptom control, synthesis and surrender.		between palliative and heart failure care specialists involving rounds with all team members every 2 weeks. Care delivered at home with easy access to care with frequency and duration of calls dependent on patient need. The team was responsible for total care including co-morbidities.<br><br>Comparison group received usual care consisting of nurse-led heart failure clinic at the hospital or primary health care centre.	2. Costs of health care: multiplying the allocated time for given services by the average salaries.<br><br>Data collected at baseline, and month six.	declined in the control group (-0.024), p=0.026.<br><br>2. Cost of intervention SEK (Swedish krona) 1.4 million (140,000 Euros). The control costed SEK 2 million (205,000 euros). The intervention reduced costs of SEK 600,000 over the 6 month intervention period.
31b	Brännstrom & Boman (2014) (46)<br><br>Sweden.	To evaluate the effect of a PCC and integrated palliative advanced home care and heart failure care.<br><br>RCT.<br><br>Person-centred palliative care model. Six S: self-image, self-determination, social	N=72 patients with CHF class III-IV.<br>n=36 intervention<br>n=36 control	Multi-disciplinary approach involving collaboration between specialists in palliative care and heart failure care (specialised nurses, palliative care nurses, cardiologists, palliative care physician, physiotherapists and occupational therapists. Patients also received structured PCC at home.	1. Symptom burden: Edmond Symptom Assessment Scale (ESAS)<br><br>2. Health related QoL-Euro QoL (EQ-5D)	1. ESAS was not significant between the groups (data not provided).<br><br>2. No significant differences in QoL between the two groups (47.7 to 60.4 in the intervention group and 48.2 to 52.3 in the control

For peer review only

Study Number	Author & Year/ Country	Aim Design Theoretical model	Sample	Intervention(s)	Outcomes/measures and follow-up period	Results
		relationships, symptom control, synthesis and surrender.		The model used the six S as Sahlen et al (2016) above<br><br>Control: usual care as described above (Sahlen et al; 2016).	3. Kansas City Cardiomyopathy Questionnaire (KCCQ)<br><br>Assessments were conducted at baseline, 3 and 6 months.	group), P=0.10. Age-adjusted analysis between groups showed delta value of HRQL from baseline to 6 months was significantly better in the intervention compared to control (p=0.02).<br><br>3. No significant differences were found between the two groups (data not provided).
32	Slok et al. (2016) (47)<br><br>The Netherlands	To assess the effectiveness of the Assessment of Burden of COPD (ABC) toll on disease specific quality of life in patients with COPD<br><br>A Cluster RCT.	N=39 primary care practices, 17 hospitals<br>N=357 COPD patients<br>n=175 intervention, mean age 64.8 (SD 8.7)	Applied the ABC tool consisting of a short validated questionnaire assessing the experienced burden of COPD, parameters of COPD lung function, and treatment algorithm including visual display and treatment advice.	Primary outcomes:<br>1. Improvement in disease-specific quality of life at 18 months; St George's Respiratory Questionnaire (SGRQ)	1. At 18-months 34% of the 146 patients from 27 health care providers in the intervention group had a clinically significant improvement in the SGRQ (at least 4 points) compared

For peer review only

Study Number	Author & Year/<br><br>Country	Aim<br>Design<br>Theoretical model	Sample	Intervention(s)	Outcomes/measures and follow-up period	Results
			n=182 control, mean age 65.8 (SD 8.8)	GPs, nurses, pulmonologists were instructed to use the ABC tool during their routine consultations. Patients visited health care professionals at least four times in 18 months. Patients were asked to fill out the ABC scale, report their dyspnoea using the MRC dyspnoea scale and self-report level of physical activity. Patients and providers could decide on treatment plan together. Patients formulated personal treatment goals.<br><br>Health care professionals in the control group provided usual care according to Dutch COPD guidelines.	Secondary outcomes:<br>2. Disease-specific quality of life; COPD Assessment Test (CAT)<br><br>3. Perceived QoL: Patient Assessment of Chronic Illness Care (PACIC)<br>Collected at four time points: baseline, 6 months, 12 months and 18 months.	with 22% of the 146 patients from the 29 healthcare providers in the control group (OR 1.85; p=0.02).<br><br>2. No significant differences in the CAT between the two groups (-0.26; p=0.68).<br><br>3. PACIC improved significantly in the intervention group compared with the control group at 18 months (0.32; p<0.01).
33	Windrum et al (2016) (48)<br><br>UK	To examine the relative impacts of alternative patient education programmes for people newly diagnosed with type 2 diabetes.	N=203 patients with Type 2 Diabetes from 6 General Practices in a city	Intervention: Patient centred education based on mediated learning. Delivered by health care professionals who attended a two-day course. Discussions were mediated	Fasting HbA1c at diagnosis and at 12 months after education programme in mmol/l.	1. HbA1c significantly lower in IG than CG after 12 months (6.838 vs 7.163, p<0.05)

For peer review only

Study Number	Author & Year/<br><br>Country	Aim<br>Design<br>Theoretical model	Sample	Intervention(s)	Outcomes/measures and follow-up period	Results
		RCT	n=94 intervention, mean age 65.8 (SD 9.69)<br>n=109 control, mean age 65.35 (SD 8.45)	between patients on key areas of health and self-management. Patients learnt to use and critically appraise information, translating it to their own individual circumstances. Patients received an 'education pack' with the same basic information as the control group and were encouraged to reflect on their own behaviour and health choices. Finally patients created a personal action plan with key goals for diet, exercise and lifestyle.<br><br>Control: Didactic course of diabetes education including causes of the condition, symptoms, diet and exercise and foot care. Patients also received NHS and Diabetes UK information leaflets.		
34	Yu (2016) (49)<br><br>Hong Kong	To develop an innovative geriatric practice, a health and	N=60 family caregivers co-residing with frail	Intervention: A comprehensive health and social assessment of	1. Caregiver perceived burden: Caregiver	1. IG had significantly greater reduction in

For peer review only

Study Number	Author & Year/ Country	Aim Design Theoretical model	Sample	Intervention(s)	Outcomes/measures and follow-up period	Results
		social collaborative case management (HSC-CM) for family caregivers of older adults and conduct a pilot RCT<br><br>Pilot RCT	older adults and providing 6 or more hours of care daily recruited from an elderly community centre run by the YWCA<br>n=30 carers in intervention group, mean age 61.5 (SD 15.5)<br>n=30 carers in control group, mean age 61.2 (SD 17.1)	caregiver and care recipient conducted in the first 4 weeks by two case managers, a registered nurse and a social worker. A case manager was assigned to provide integrated, coordinated continued care from week 5-16. Caregivers were invited to attend group workshops according to their needs to optimise informational, emotional and social support between peers.<br><br>Control: usual care.	burden inventory (CBI, Chinese version).<br><br>2. Caregiver and health-related quality of life: Medical Outcomes Study 36-item Short Form Health Survey (SF-36 Chinese version)	perceived burden (p=0.03) than CG<br><br>2. IG had significant improvement in vitality (p=0.049), social role functioning (p=0.47) and general well-being (p=0.49).
35	Hernandez et al, (2015) (50)<br><br>USA	Explore the effectiveness of a community-based integrated care (IC) service in preventing hospitalisations and emergency department visits in stable frail COPD patients<br><br>RCT	N=155 COPD patients.<br>n=71 intervention. Mean age 73 (SD=8) years.<br>n=84 control, mean age 75 (SD=9) years.	A 2-h educational programme administered by nurse covering disease knowledge, non-pharmacological treatments, techniques for pharmacological administration, and self-management of the disease and co-morbid conditions and strategies to adopt with future exacerbations. A joint	1. Hospital admission and visit to emergency department<br><br>2. Mortality	1. IC group showed decline in risk of emergency room visits; OR: 0.33 p=0.02. Hospital admissions did not differ significantly OR: 2.17; p=0.237<br><br>2. Mortality reduced in the IC group OR:0.36; p=0.034

For peer review only - http://bmjopen.bmj.com/site/about/guidelines.xhtml

Study Number	Author & Year/ Country	Aim Design Theoretical model	Sample	Intervention(s)	Outcomes/measures and follow-up period	Results
				visit of the specialist nurse and the primary care team (physician, nurse, social worker) at patient's home within 72 hours after study entry.<br>Community care team received 2 h face-to-face educational training and 1 day stay at the hospital ward, aiming at enhancing home-based management of frail COPD patients. Number of home visits individually tailored to patient needs.<br><br>Usual care: Comparison group received conventional treatment being managed by their physician without any support from specialised nurses. Visits were every 6 months in the out-patient clinic.	3. Dyspnoea: MRC dyspnoea scale<br><br>4. Anxiety and depression: HADS<br><br>5. QoL: St George's Respiratory Questionnaire<br><br>6. COPD knowledge and self-management	3. No difference between groups (p=0.96) at 12 months<br><br>4. No differences on anxiety between the groups (p=0.13), but depression significantly improved in the IC group (p<0.01) at 12 months<br><br>5. Symptoms score significantly reduced in the IC group compared with the control group 32 vs 42 p=0.02, activity and impacts scores did not change significantly 63 vs 69; p=0.20, 36 vs 40; p=0.28 respectively.<br><br>6. knowledge significantly increased in the IC

For peer review only

Study Number	Author & Year/<br><br>Country	Aim<br>Design<br>Theoretical model	Sample	Intervention(s)	Outcomes/measures and follow-up period	Results
					 <br><br><br>7. Percentage of current smokers	group compared with the control group 40 vs 25; p=0.02<br><br>7. Lower percentage of current smokers in the intervention group (3% vs 16%, p=0.002.
36	Kikkenborg et al (51)(2015)<br><br>Denmark	To examine the potential effects of a short psychoeducational nursing intervention on primary emotions and describe the trajectory of primary emotions over time in patients with implantable cardioverter defibrillators (ICD).<br>RCT<br><br>Theory of nursing, Rosemary Rizzo Parses Human Becoming Practice	N=196 adults with first time ICD implantation<br>n=99 intervention group, mean age 58<br>n=97 control group, mean age 58	Intervention: Three monthly, one hour nurse led psychosocial support and education sessions commencing on discharge.<br><br>Control: Usual care plus an invitation to attend a single 2 hour group session with information and sharing of experiences but no individual psycho-educational follow-up.	1. Primary Emotions using The Emotions and Health Scale<br>Measured at baseline and 3 months	1. No significant differences in primary emotions between intervention and control groups at 3 months.<br>Joy (11 vs 10.8, p=0.76),<br>Agreeableness (10.4 vs 10.2, p=0.64),<br>Surprise 77 vs 80, p=0.67, Fear 6.76 vs 6.94, p=0.42,<br>Sadness (8.15 vs 7.64, p=0.06)<br>Disgust (4.62 vs 4.96, p=0.83), Anger (5.68 vs 6.04, p=0.97, Anticipation

Study Number	Author & Year/ Country	Aim Design Theoretical model	Sample	Intervention(s)	Outcomes/measures and follow-up period	Results
						8.34 vs 8.83, p=0.35).
37a	Larsson et al (2015) (52)<br><br>Sweden	To compare the costs of rheumatology care between a nurse-led rheumatology clinic (NLC) based on person-centred care (PCC), versus a rheumatologist-led clinic (RLC) in monitoring patients with chronic inflammatory arthritis (CIA) undergoing biological therapy.<br><br>RCT<br><br>Gothenburg PCC	N=97 patients with CIA undergoing biological therapy and a disease activity score (DAS28 </=3.2) recruited from a rheumatology clinic in Southern Sweden<br>n=47 intervention group, mean age 55.0 (SD 12.3)<br>n=50 control group, mean age 55.8 (SD 13.2)	Intervention: Patients randomised to attend a NLC based on the principles of patient centred care. In addition to assessing disease activity and medication, visits focussed on patients needs and global health. Patients could contact their nurse when needed between appointments.<br>Control: attending a Rheumatologist led clinic. Visits to both clinics lasted about 30 minutes.	Total annual use of resources and direct costs of care monitoring biological therapy over 12 months<br>Secondary outcome measures:<br>Annual use of resources and direct costs for the components of the primary outcome (fixed monitoring, variable monitoring, rehabilitation, specialist consultations, radiography and pharmacological therapy).	Statistically significant lower costs in IG than CG (€14107.7 vs €16274.9 per patient, p=0.004)<br><br>Statistically significant cost reductions in total fixed monitoring (-€116.7, p=0.001), total (fixed and variable) monitoring (-€155.0, p=0.001) and pharmacological therapy (-€1444.5, p=0.029). No statistically significant reduction in monitoring visits, blood tests, additional phone consultations, inpatient and outpatient rehabilitation, physiotherapy,

**Study Number**	**Author & Year/<br><br>Country**	**Aim<br>Design<br>Theoretical model**	**Sample**	**Intervention(s)**	**Outcomes/measures and follow-up period**	**Results**
						occupational therapy, psychosocial treatment, specialist consultations or radiography.
37b	Larsson et al (2013) (53)<br><br>Sweden	To compare and evaluate the treatment outcomes of a nurse-led rheumatology clinic and a rheumatologist clinic in patients with low disease activity or undergoing remission who are undergoing biological therapy<br><br>RCT<br><br>Gothenburg PCC	n= 107 patients with chronic inflammatory arthritis undergoing biological therapy and a disease activity score (DAS28 </=3.2) recruited from a rheumatology clinic in Southern Sweden<br>n=53 intervention, mean age 55 (SD 12.3)<br>n=54 control, mean age 55.8 (SD 13.2)	Intervention: Patients randomised to attend a NLC based on the principles of patient centred care. In addition to assessing disease activity and medication, visits focussed on patients needs and global health. Patients could contact their nurse when needed between appointments.<br><br>Control: attending a Rheumatologist led clinic. Visits to both clinics lasted about 30 minutes	Primary outcome:<br><br>1. Disease activity: DAS28 and DAS28-CRP<br><br>Secondary outcomes:<br>2. Performing Activities of Daily Living (ADLs): Health Assessment Questionnaire (HAQ)<br><br>3. Pain assessed by Visual Analogue Scale<br><br>4. Satisfaction in obtaining rheumatology	Mean difference of change (IG-CG) between groups not statistically significant for any primary or secondary outcome<br>1. DAS28 (-0.06, p=0.66) or DAS28-CRP (0.05, p=0.70)<br><br>2. 0.02, p=0.79<br><br>3. Non-significant -0.24, p=0.95<br><br>4. Non-significant 0.25, p=0.43

For peer review only

Study Number	Author & Year/ Country	Aim Design Theoretical model	Sample	Intervention(s)	Outcomes/measures and follow-up period	Results
					care: Numerical Rating Scale<br><br>5. Confidence in obtaining rheumatology care: Numerical Rating Scale	5. Non-significant 0.2, p=0.42
38	Lowther et al (2015) (54)<br><br>Kenya	To evaluate the effectiveness of a nurse-led palliative care intervention among people with HIV<br><br>RCT	N=120 participants with HIV<br><br>n=60 intervention, mean age 38.3 (SD 8.2)<br>n=60 control, mean age 40.5 (SD9.2)	Patients in the intervention arm<br>received clinical care from a nurse who has received two weeks' training in palliative care and ongoing clinical<br>support and supervision from experienced palliative care providers.<br><br>Control group received care from nurse's who had no exposure to palliative care training.	Primary Outcome:<br>1.Pain severity: African Palliative Care Outcomes (APOS)<br><br>Secondary Outcomes:<br>2.Psychiatric morbidity: GHQ-12<br><br>3. Quality of Life (mental and physical: Medical Outcomes Study (MOS)-HIV	1.Mean change was +3.5 in the intervention and +4.0 in the control (p=0.83)<br>Total APOS mean change was +12 in the intervention and +7.5 in the control (p=0.04).<br><br>2. Significant difference was seen between intervention and control (-0.50; p=0.04).<br><br>3. Significant differences between groups on mental health subscale

For peer review only

Study Number	Author & Year/ Country	Aim Design Theoretical model	Sample	Intervention(s)	Outcomes/measures and follow-up period	Results
					Outcomes assessed at baseline, one, two, three and four months.	(0.61; p=0.01) but no significant differences between groups on physical aspects of QoL(0.44; p=0.06).
39	Kelechi et al. (2014) (55)<br><br>USA	To test the feasibility and efficacy of a motivational enhancement and conditioning activity for leg function (MECALF) in patients with critically colonized/infected chronic leg ulcers.<br><br>Comparative study<br>Motivational Enhancement	N=21 patients with critically colonised or infected leg or foot ulcers.<br>n=12 intervention<br>n= 9 control	Intervention: MECALF. Specialist nurses received 8 hours of training in motivational enhancement (ME). They used 10 minutes of each weekly wound visit to engage in ME over 6 weeks. Patients were given a brochure detailing an exercise programme (CALF) to promote walking and other physical activities developed by a physical therapist.<br><br>Control: CALF. Usual wound care as per protocols. Patients received the CALF exercise brochure but no ME.	Data collected at baseline and week 8 (2 weeks post intervention)<br>1. Pain : Leg Pain Questionnaire (LPQ)<br><br>2. Strength: dyanometer for ankle dorsiflexion and plantar flexion in lb/in^2<br><br>3. Ankle range of motion: goniometry for dorsiflexion, plantar flexion, inversion and eversion in degrees<br><br>4. Motivation: readiness ruler	1. Reduced pain at 8 weeks in CG compared to IG (p=0.046)<br><br>2. No statistically significant difference between groups.<br><br>3. No statistically significant difference between groups at 8 weeks (p=0.748)<br><br>4. No statistically significant difference between groups (p=0.641)

For peer review only

Study Number	Author & Year/<br><br>Country	Aim<br>Design<br>Theoretical model	Sample	Intervention(s)	Outcomes/measures and follow-up period	Results
					5. Self-efficacy/confidence: Questionnaire for Physical Activity and Exercise<br><br>6. Functional physical activity: Timed chair rise test, timed up and go, community healthy activities model for program for seniors (CHAMPS).	5. No statistically significant difference between groups (p=0.643)<br><br>6. No statistically significant difference between groups in any measure.
40	Young et al (2013) (56)<br><br>Australia	To investigate the effectiveness of a centralised, nurse-delivered telephone based service to improve care coordination and patient reported outcomes after surgery for colorectal cancer.<br><br>RCT	N= 756<br>n=387 intervention group, mean age 86.9 (SD 12.2)<br>n=369 control group, mean age 67 (SD 12.1)	Five scheduled, structured telephone calls from a nurse on days 3 and 10 then at 1,3 and 6 months after hospital discharge. Identified needs were addressed by the nurse using detailed standardized clinical protocols.<br><br>Control group received usual care.	Primary and secondary outcomes not specified.<br>1. Total care coordination score at 3 and 6 months<br><br>2. Global assessment of care coordination at 3 and 6 months	1. No significant differences between intervention and control groups at 3 (79.5 vs 78.7, p=0.3) or 6 months (80 vs 80.3, p=0.8).<br><br>2. No significant differences between intervention and control groups median scores at 3 (9 vs 9, p=1.0) or 6 months (10 vs 10, p=0.1).

Study Number	Author & Year/ Country	Aim Design Theoretical model	Sample	Intervention(s)	Outcomes/measures and follow-up period	Results
					3. Global assessment of quality of care at 3 and 6 months	3. No difference in intervention and control groups median scores at 3 (10 vs 10, p=1.0) or 6 months (10 vs 10, p=1.0)
					4. Supportive Care Needs Survey Short Form (SCNS-SF34) at 3 and 6 months	4. No difference in intervention and control group unmet needs median score at 3 (59.9 vs 56.8, p=0.6) or 6 months (50.0 vs 46.6, p=0.7)
					5. Unplanned readmissions at 1 and 6 months	5. No difference between intervention and control group in unplanned admissions at 1 (8.6 vs 10.5%, p=0.4) or 6 months (25.6 vs 27.9%, p=0.5)
					6. Emergency room presentations at 1 and 6 months	6. No difference between intervention and control group in emergency room presentations at 1 (10.8 vs 13.8%,

For peer review only

Study Number	Author & Year/ Country	Aim Design Theoretical model	Sample	Intervention(s)	Outcomes/measures and follow-up period	Results
						p=0.2) or 6 months (25.9 vs 25.4%, p=0.9)
					7. Proportion receiving postoperative chemotherapy	7. No significant difference between intervention and control groups in proportion receiving postoperative chemotherapy (73 vs 78%, p=0.5)
					8. Distress at baseline, 1, 3 and 6 months	8. No difference in intervention and control groups in mean distress scores at 1 (2.3 vs 2.4, p=0.1), 3 (2.0 vs 2.0, p=0.3) or 6 months (1.8 vs 1.8, p=0.2)
					9. Functional Assessment of Cancer Therapy- Colorectal (FACT-C) total score at baseline, 1, 3 and 6 months	9. No significant difference between intervention and control groups in FACT-C total score at 1 (100.61 vs 100.40, p=0.4, 3 (103.48 vs103.26,

For peer review only

Study Number	Author & Year/ Country	Aim Design Theoretical model	Sample	Intervention(s)	Outcomes/measures and follow-up period	Results
						p=0.4) or 6 months (105.10 vs 105.35, p=0.5)
41	Chochinov (2011) (57)<br><br>USA, Canada and Australia	To determine if dignity therapy could mitigate stress and/or bolster end-of-life experience for patients nearing death<br><br>Multi centre RCT	N=326 patients receiving hospital or community based palliative care<br>n=108 dignity therapy, mean age 64.2 (SD 14.6)<br>n=107 client centred care, mean age 64.3 (SD 14.3)<br>n=111 standard palliative care, mean age 66.7 (SD 14.2)	Dignity Therapy: novel brief (30 min) psychotherapy session providing an opportunity to speak about things that matter most to the patient often relating to meaning and purpose. Sessions were transcribed to produce a document that could be bequeathed to a recipient of patient's choice. Therapists undertook 3 day training.<br>Client Centred Care: Supportive psychotherapeutic approach focussing on 'here and now' issues such as symptoms and their illness. No permanent record of conversation given to patient.<br>Standard Palliative Care: access to MDT palliative care support services.	Primary outcomes:<br>1. Mean change in baseline and end of intervention<br><br>2. Palliative Performance Scale<br><br>3. FACIT spiritual well-being scale<br><br>4. Patient dignitary inventory (PDI)<br><br>5. Hospital anxiety and depression scale (HADS)<br><br>6. Items from Structured Interview for Symptoms and Concerns (SISC) including dignity, desire for death, suffering, hopelessness, depression, suicidal ideation and sense of burden to others.	Primary outcomes:<br>1-7. No significant differences found in change from baseline to end of intervention between the three groups in any outcome measure.

For peer review only - http://bmjopen.bmj.com/site/about/guidelines.xhtml

Study Number	Author & Year/<br><br>Country	Aim<br>Design<br>Theoretical model	Sample	Intervention(s)	Outcomes/measures and follow-up period	Results
				Control group: Participants assigned to the control group received Standard Palliative Care which included access to the full range of palliative care support services available to all study patients, including specialist palliative care physicians and nurses (i.e. experts in pain and symptom management), social workers, chaplains, and psychologists and/or psychiatrists. No participating site provided a formal approach to addressing generativity issues; as such, a program comparable to Dignity Therapy was not available to patients who were not randomized to the Dignity Therapy arm of this trial.	7. Two item quality of life scale<br><br>Secondary outcome:<br>8. Detailed survey of experience of study	8. Dignity therapy group more likely to have found the study helpful (p<0.001), that it improved their quality of life (p<0.001), sense of dignity (p=0.002), spiritual wellbeing (p=0.006), lessened sadness or depression (p=0.009) and felt satisfied with the study arm assignment (p<0.001). The Dignity Therapy group were likely to report that being in the study changed how their family appreciate and see them (p<0.001) and

For peer review only

Study Number	Author & Year/<br><br>Country	Aim<br>Design<br>Theoretical model	Sample	Intervention(s)	Outcomes/measures and follow-up period	Results
						that it will help their family p<0.001).
42	Goelz et al (2011) (58)<br><br>Germany	To demonstrate that COM-ON-p concise and individualized communication skills training (CST) improves oncologists communication skills in consultations focussing on the transition to palliative care<br><br>RCT	N=41 physicians in charge of patients with cancer and practising at a University Medical Centre in Germany<br>n=22 physicians in intervention group<br>n=19 physicians in control group	Intervention: Participants undertook the COM-ON-p training programme including pre-assessment with an actor patient (1 hour), a 1.5 day workshop and an individual coaching workshop (30 mins) 2 weeks after the workshop and post assessment with an actor patient (1 hour). Facilitators were experienced in oncology and CST and helped physicians focus on individual learning goals which they had developed with video analysis.<br><br>Control: No additional training.<br>All physicians undertook 2 video recorded consultations with actor patients at baseline and 5 weeks later.	COM-ON-Checklist: Participants were ranked on 5 point scale for relevant behavioural domains.<br>Primary outcome:<br><br>1. Section A average score for 6 items specific to the transition to palliative care<br><br>2.Section B average score for 9 general communication items<br><br>Secondary outcome:<br>3. Involving significant others: Section C average score of 4 items on the involvement of significant others and global item 2.	1. IG had significantly higher scores than CG after intervention (Effect size 0.78, p=0.0026)<br><br>2. IG had significantly higher scores than CG after intervention (Effect size 0.78, p=0.0078.<br><br>3. IG had significantly higher scores than CG after intervention (Effect size 0.65, p=0.0070).

For peer review only

Study Number	Author & Year/<br><br>Country	Aim<br>Design<br>Theoretical model	Sample	Intervention(s)	Outcomes/measures and follow-up period	Results
43	Murphy et al (2010) (59)<br><br>USA	To examine whether tailored activity pacing intervention was more effective than general activity pacing intervention for managing pain and fatigue in adults with osteoarthritis.<br><br>RCT	n=13 intervention group with OA, mean age 63.9 (SD=7.8)<br>n=11 control group with OA, mean age 59.5 (SD= 6,6)	Intervention: Education module on activity pacing tailored to the individual delivered by an occupational therapist. Participants undertook 5 days of home monitoring of activity levels with an accelerometer and a log of symptoms and activity. A personalised report detailing the relationship between activity and symptoms was the basis for pacing recommendations. Second session focussing on individual progress.<br><br>Control: Education module on generalised activity pacing delivered by an occupational therapist with advice to implement the strategies. Second session focussing on individual progress.	Primary outcomes:<br>1. Pain: WOMAC<br><br>2. Fatigue: Brief Fatigue Inventory<br>Data collected at baseline and 10 week follow up	1. WOMAC pain score decreased from baseline to week 10 in the control group (9.4 to 7.6) and the intervention group (7.9 to 6.7). The difference between groups was not statistically significant (p=0.35) with small effect size d=0.38.<br><br>2. BFI Fatigue Severity reduced in the control group (4.3 to 4.8) and the intervention group (4.1 to 3.3). The difference between groups was not statistically significant (p=0.09) with a moderate to large effect size (d=0.79) BFI Fatigue Interference increased in the control group (3.6 to

For peer review only

Study Number	Author & Year/ Country	Aim Design Theoretical model	Sample	Intervention(s)	Outcomes/measures and follow-up period	Results
						4.2) and decreased in the intervention group (3.1 to 1.6). The difference between groups was statistically significant (p=0.02) with a large effect size (d=1.10)
44	Wolff et al (2010) (60)<br><br>USA	Determine whether guided care (GC) improves patients' primary caregivers' depressive symptoms, strain, productivity and perceptions of quality of care for care recipients.<br><br>Clustered RCT	N=308 primary caregivers/patient dyads<br>n= 156 intervention caregivers (mean age 60.9 years)/patient (mean age 78.0 years) dyads randomised to Guided Care (GC)<br>n=152 usual care caregiver (mean age 61.6)/patient (mean age 77.9) dyads (UC)<br>n=22 usual care, mean age 31.91 (SD=6.52), male	Guided Care (GC)provided by nurses: included training and supporting patient's family caregivers. Designed to address deficiencies in the quality of chronic care delivery by facilitating coordinated, comprehensive, evidence-based heath care for multimorbid adults.<br><br>GC nurses collaborated with patients PCP to provide clinical processes: assessing the patient at home, creating an evidence-based care plan, promoting patient self-management, proactively monitoring patient condition,	Primary outcomes:<br><br>1. Caregiver depressive symptoms: Centre for Epidemiological Studies (CES-D)<br><br>2. Caregiver strain: Modified Caregiver Strain Index (CSI)	At 18 months follow-up:<br><br>1. CES-D changed from 6.4 to 6.8 in the GC compared with 7.1 to 5.8 in the UC. The results were not statistically significant between groups<br><br>2. CSI increased from 6.5 to 6.7 in the GC group and 6.6 to 7.7 in the UC group. These results were not statistically significant between the two groups.

For peer review only

Study Number	Author & Year/<br><br>Country	Aim<br>Design<br>Theoretical model	Sample	Intervention(s)	Outcomes/measures and follow-up period	Results
			gender n=21 (95.5%). Participants recruited within 14 primary care physician teams (PCP)	coaching the patient to practice healthy behaviours, coordinating patients transition between sites and providers of care, facilitating access to community resources, and educating and supporting patients family caregivers.<br><br>Comparison group received usual care (details not provided).	3. Quality of Chronic Illness Care: modified version of the Patient Assessment of Chronic Illness Care (PACIC)<br><br>4.Caregiver Productivity Loss: Work Productivity and Activity Impairment questionnaire (WPAI:CG)<br><br>Baseline and 18-month follow-ups.	3.Aggregate QoL was higher in the GC group compared with the usual care group (0.40; p<0.001)<br><br>4. Work productivity loss was more substantial in the GC group compared with the UC group (14.6% to 8.4% vs 18.2% to 16.1%). Presentism declined from 16.7% to 11.9% in the UC group compared with 12.9% to 5.3% in the GC group.
45	Dobscha et al (2009) (61)<br><br>USA	To assess whether a collaborative intervention can improve chronic pain-related outcomes in a Department of Veteran Affairs (VA) primary care setting.<br><br>Cluster RCT	N=401 patients at 5 primary care clinics with moderate or severe chronic pain<br>n=187 intervention group, mean age 62.1 (SD 11.2)	Intervention: clinicians in intervention practices undertook two 90 minute workshops including abbreviated training in shared decision making skills and chronic pain education. Patients received an assessment with a care manager to	Primary Outcome:<br>1. Self-reported pain disability: Roland Morris Disability Questionnaire for pain (RMDQ) score<br>Additional main outcomes:<br><br>2. Depression severity: PHQ-9	1. Greater improvement from baseline to 12 months in intervention group than control (-1.4 vs -0.2, p=0.004).<br><br>2. Greater improvement from

For peer review only

Study Number	Author & Year/ Country	Aim Design Theoretical model	Sample	Intervention(s)	Outcomes/measures and follow-up period	Results
			n= 214 control group, mean age 61.3 (SD 12.3)	develop individualised functional goals and a treatment plan was communicated to the clinician. Patients were invited to a four session workshop based on the brief activating approach. Care managers contacted patients every 2 months for 12 months to provide support and reassess goals and activities.<br><br>Control: treatment as usual including referral to speciality pain clinic, ancillary services such as physiotherapy and occupational therapy.	3. Pain intensity: CPG Pain Intensity subscale<br><br>Secondary outcomes:<br>4. CPG Pain interference subscale<br><br>5. Patient rated global impression of change<br><br>6. Global VA health care satisfaction	baseline to 12 months in IG than CG (-3.7 vs -1.2, p=0.003).<br><br>3. Greater improvement from baseline to 12 months in IG than CG (-4.7 vs -0.6, p=0.01).<br><br>Secondary outcomes:<br>4. Improvement from baseline to 12 months in IG and worsening in CG (-5.7 vs 2.3, p=0.03)<br><br>5. Greater improvement in IG than CG at 12 months (3.7 vs 4.4, p<0.01)<br><br>6. No difference in change from baseline to 12 months in IG and

For peer review only

Study Number	Author & Year/<br><br>Country	Aim<br>Design<br>Theoretical model	Sample	Intervention(s)	Outcomes/measures and follow-up period	Results
					7. Health related quality of life: EQ-5D<br><br>8. Effectiveness of VA chronic pain treatment Outcomes collected at baseline, 3, 6 and 12 months.	CG (-0.27 vs -0.36, p=0.44)<br><br>7. No difference between IG and CG in change from baseline to 12 months (-0.02 vs -0.04, p=0.17)<br><br>8. No difference in change from baseline to 12 months in IG and CG (0.33 vs 0.2, p=0.64)
46	Machado et al, (2007) (62)<br><br>Brazil	To compare effectiveness of psychotherapy based on client-centred therapy and exercise for patients with chronic nonspecific low back pain<br><br>RCT.	N=33 participants with nonspecific low back pain (LBP)<br>n=16 intervention, mean age 44.6 (SD=12.1) years.<br>n=17 control, mean age 42.4 (SD=13.2) years.	Psychotherapy based on the principles of nondirective counselling. Patients in groups attended 80 minute treatment sessions twice a week for 9 weeks. Therapists provided support as patients discussed life stressors, including chronic pain.<br><br>Control group received Physiotherapists-led exercise therapy. General	1. Disability: Brazil Roland-Morris Questionnaire (BRM)<br><br>2. Pain: Visual Analogue Scale (VAS)	1. Exercise group showed lower disability at 9 weeks compared with the psychotherapy group (-4.9 points difference; p=0.02), at 6 months (4 points difference; p=0.13)<br><br>2. Pain scores were not significantly lower in the exercise

For peer review only

Study Number	Author & Year/<br><br>Country	Aim<br>Design<br>Theoretical model	Sample	Intervention(s)	Outcomes/measures and follow-up period	Results
				exercise consisting of 20 minute walking, general stretching, and strengthening of the bridge (lying supine with knees flexed, raising hips and hold for 5 seconds, repeating the procedure for 15 minutes). Patients attended the 40 minute sessions in groups, twice a week for 9 weeks.	3. Depressive symptoms: Beck Depression Inventory (BDI)<br><br>Assessments conducted at baseline, 9 weeks and 6 months (depression was not assessed at 6 months).	group compared with psychotherapy group at nine weeks (-1.8; p=0.27)<br>At six months the exercise group again scored lower compared with the psychotherapy group (-1.3; p=0.38).<br><br>3. Exercise group showed less depressive symptoms compared with the psychotherapy group at nine week (-6.3 points difference; p=0.29).
47	Glasgow et al (2005) (63)<br><br>USA	To determine if an interactive computer technology intervention designed to improve patient centred communication	N=886 adults with Type 2 Diabetes under the care of 52 primary care physicians n=469 intervention	Intervention: Before two appointments, 6 months apart, patients completed computerized touch screen assessments including recall of clinical interventions and	Primary outcome:<br>1. Patient reports of receiving American Diabetes Association recommended laboratory screenings and	Primary outcome:<br>1. intervention group had greater improvement in laboratory screenings completed than

For peer review only

Study Number	Author & Year/ Country	Aim Design Theoretical model	Sample	Intervention(s)	Outcomes/measures and follow-up period	Results
		improves diabetes care.<br><br>Cluster RCT	group, mean age 62 (SD 1.4) n=417 control group, mean age 64 (SD 1.3)	developing a self-management action plan. Received detailed personalised printout of results. Patients met a Care manager trained in patient centred self-management approaches to review care needs and self-care goals followed by a follow-up call after each visit.<br><br>Control: Completed the same touch screen computer assessment but received a print- out of general health risks. No meetings or calls from care manager but same number of physician appointments.	recommended patient centred care activities Secondary outcomes.<br><br>2. Diabetes quality of life (The revised Problem Area in Diabetes 2 Scale, PAID-2)<br><br>3. HbA1c<br><br>4. Total cholesterol to HDL cholesterol ratio.<br><br>5. Depression (Patient Health Questionnaire, PHQ-9, % with 10 or higher).<br><br>Outcomes measured at baseline and 12 months.	controls (F=11.6, p<0.001) and patient centred activities (F=39.5, p<0.001).<br><br>2. No significant difference between intervention and control groups at 12 months (27.4 VS 27.5, p=0.964).<br><br>3. No difference in HbA1c between intervention and control groups (7.11 vs 7.17%, p=0.571.<br><br>4. No difference between intervention and control groups (4.11 vs 4.15, p=0.733).<br><br>5. No difference between intervention and control groups (12.3 vs 13.9%).

For peer review only

Study Number	Author & Year/ Country	Aim Design Theoretical model	Sample	Intervention(s)	Outcomes/measures and follow-up period	Results
48	Mills et al (2003) (64)<br><br>Australia	Geographically controlled study	N=509 people with Type 2 Diabetes in rural Australia n=398 intervention n=111 control	Intervention: Care planning using a patient centred care planning model. Emotions, thoughts and behaviours translated into patient specific problem statements then goals. Care plans created and reviewed annually. Relevant health services were scheduled in line with best practice. Patients were followed for two years at minimum 6 month intervals.<br><br>Control: usual care in rural Southern Australia	1.Problem and goal scores recorded on linear analogue scale recorded by patients and service co-ordinators<br><br>2.Work and social adjustment: Work and Social Adjustment Scale (WASAS) at each visit.<br><br>3.Medical Outcomes Study 36-Short Form (SF36).<br><br>4.Emergency and elective admission rates	1.Up to 60% of IG felt their main problem improved by the end of the trial. 40-60% of patients made some progress toward achieving their first goal.<br><br>2. The WASAS scores between the two groups were statistically significant (P < 0.01) over time, with mean scores improving 10%.<br><br>3.Statistically significant difference (p<0.01) between IG and CG in SF 36.<br><br>4. IG group hospital admission rate fell 18.2% compared to CG.

For peer review only

Study Number	Author & Year/<br><br>Country	Aim<br>Design<br>Theoretical model	Sample	Intervention(s)	Outcomes/measures and follow-up period	Results
49	Kennedy, et al (2003) (65)<br><br>UK	To evaluate the effects of a PC intervention on clinical outcomes and health service use among patients with inflammatory bowel disease (IBD).<br><br>Multicentre cluster RCT.	N=19 hospitals, outpatient (n=9 treatment, n=10 control).<br>n=635 patients with inflammatory bowel disease (IBD)<br>n=270 intervention (mean age 44.4, sd=14.9)<br>n=365 control (mean age 46.3, sd 15.1)	Clinicians at the intervention sites received a 2-hr training session led by an expert in postgraduate medical education using role play and video feedback titled 'patient-centred consultation in gastroenterology'. Training focused in PC medicine principles and applied to self-management in IBD. Patients at the intervention sites participated in PC consultations conducted by clinicians. A self-management plan was negotiated and written into the guidebook. Patients were instructed to call a specified number if they needed to schedule an appointment according to circumstances listed in the guidebook.<br><br>Patients at the control sites received management processes deemed appropriate by hospital specialists.	1. Hospital appointments<br><br>2. Quality of life: Inflammatory bowel disease questionnaire (IBDQ)<br><br>3. Anxiety and depression: Hospital Anxiety and Depression Scale (HADS)	1. The number of kept appointments reduced by app. one third in the intervention group compared with the control group (difference -1.4; p<0.001). The mean number of clinic non-attendances per person during the trial was also lower for the intervention group (difference -0.08; p=0.034).<br><br>2. IBDQ did not differ significantly between the two groups (difference 1.94; p=0.45)<br><br>3. HADS did not differ significantly between two groups (difference -0.35; p=0.40)

Study Number	Author & Year/ Country	Aim Design Theoretical model	Sample	Intervention(s)	Outcomes/measures and follow-up period	Results
					4. Patient enablement: patient enablement instrument (PEI)<br><br>5. Satisfaction : Consultation satisfaction questionnaire (CSQ).	4. the intervention group showed a higher enablement score (difference 0.90; p=0.026)<br><br>5. satisfaction did not differ significantly between the two groups (3.47; p=0.09).
50	Martin et al, (2004) (66)<br><br>New Zealand	To test whether individualised care plan for patients experiencing acute exacerbations of COPD result in reduced health care utilisation and improved quality of life<br><br>RCT.	N=93 COPD patients<br>n=44 intervention group, mean age 71.1 years.<br>n=49 control group, mean age 61.9 years.	Individualised care plan based on an interview between patient and respiratory nurse, review of hospital records by respiratory specialist and by patient's own GP. Each patient was given instructions about how to use the plan by the respiratory nurse. Copies of the plan were held by patient, GP, ambulance service, emergency department and after hour's surgery.<br><br>Control group received usual care. They did not	Primary outcome:<br>1.Utilisation of primary care services and hospital admissions	1. Intervention group called out the ambulance service more frequent (2.8 vs 1.1) calls per 12 months (p=0.03).<br>Intervention group had more GP visits compared with control group (15.6 vs 11.6) in 12 months; p=0.08<br>The intervention group has more hospital admissions compared with the control group (1.1 vs 0.7); p=0.17.

For peer review only

Study Number	Author & Year/ Country	Aim Design Theoretical model	Sample	Intervention(s)	Outcomes/measures and follow-up period	Results
				have an individualised care plan. All participants remained under the care of their own GP.	2. Quality of Life: St George's Respiratory Questionnaire (SGRQ) Outcomes assessed at baseline, three, six and 12 months.	2. SGRQ did not differ significantly between groups (57.3,sd=13.5 for intervention and 55.1, sd=14.6) for control.
51	Alamo, et al, (2002) (67) Spain	To assess whether patient-centred consultations are more effective than usual care style of consultations among patient with chronic musculoskeletal pain and fibromyalgia Clustered RCT	N=20 GP's in 13 health centres. N=110 patients n=10 GP's intervention, n=10 GP's control. N=63 (mean age 39.2; sd=7.6 years) patients intervention N=47 (mean age 42.3; sd=10) patients control	GP's in the intervention received training on communication skills necessary to undertake PC approach. These focused on active listening, asking patients' to express their fears and concerns, offering reassurance, coming up with a management plan together with the patient. Control group GP's provided usual care	1. Pain intensity: VAS and pain scale of the Nottingham health profile (NHP) questionnaire 2. Number of tender points and subjective health status: NHP questionnaire	Pain reduced in the intervention group (mean pain at baseline 3.4 (sd=1.2), at 6 months 3.3 (sd=1.0) and at 12 months 3.1 (sd=1.0). Mean pain in the control group was 4.1 (sd=0.8), at 6 months 3.9 (sd=0.8) and at 12 months 3.9 (sd=0.8). The difference between the two groups was not statistically significant (p=0.73) 2.Number of tender points reduced significantly in the intervention group

For peer review only

Study Number	Author & Year/<br><br>Country	Aim<br>Design<br>Theoretical model	Sample	Intervention(s)	Outcomes/measures and follow-up period	Results
					3. Psychological disturbance: Goldberg Scale of anxiety and depression (GHQ)<br><br>Participants were followed-up at 6 and 12 months.	compared with the control group (p=0.05)<br><br>3.GHQ anxiety significantly reduced in the intervention compared with the control group (p=0.04)<br>GHQ depression was not statistically significant (p=0.33)
52	Sommers et al. (2000) (68)<br><br>USA	To examine the impact of an interdisciplinary, collaborative practice intervention involving a primary care physician, a nurse, and a social worker for community-dwelling seniors with chronic illnesses<br><br>Concurrent, controlled cohort study	N=543 patients aged 65 or older under treatment for at least 2 chronic conditions. Recruited from 18 private primary care physician offices<br>n=280 intervention group, mean age 78 (SD 6.8)	Intervention: home assessment from a nurse or social worker including listening to health concerns, home safety check and functional assessment. Creation of risk reduction plans and treatment plans based on chronic disease self-management strategies. Follow up sessions at least every 6 weeks including telephone, home visit, small group sessions or office or hospital visit.	Utilisation of medical services at baseline, 1 and 2 years<br>1. Change in number of hospital admissions per patient per year<br><br>2. Change in percentage of patients with 1 or more hospital readmissions within 60 days<br><br>3. Change mean number of visits to all physicians	1. Statistically significant reduction in admissions in IG vs CG (-0.02 vs 0.18, p=0.03)<br><br>2. Statistically significant reduction in readmissions in IG vs CG (-2.0 vs 5.4, p=0.03)<br><br>3. Statistically significant reduction in visits in IG vs CG

For peer review only

Study Number	Author & Year/<br><br>Country	Aim<br>Design<br>Theoretical model	Sample	Intervention(s)	Outcomes/measures and follow-up period	Results
			n=263 control group, mean age 77 (SD 6.6)	Control: usual care from the primary physician	4. Change in percentage of patients with 1 or more visits to the emergency department<br><br>5. Change in proportion of patients with 1 or more home care visits<br><br>6. Change in number of patients with 1 or more nursing home placements<br>Patient reported health status at baseline, 1 and 2 years.<br><br>7. Change in Health Activities Questionnaire<br><br>8. Geriatric Depression Scale	(-1.5 vs 0.5, p=0.003)<br><br>4. No difference in change between IG and CG (1.2 vs -0.66, p=0.77)<br><br>5. No difference in change between IG and CG (1.8 vs -2.6, p=0.81)<br><br>6. No difference in change between IG and CG (5.0 vs -5.4, p=0.59)<br><br>7. No difference in change between IG and CG (0.03 vs 0.08, p=0.14<br><br>8. No difference in change between IG and CG (0.3 vs 0.5, p=0.52)

For peer review only

Study Number	Author & Year/<br><br>Country	Aim<br>Design<br>Theoretical model	Sample	Intervention(s)	Outcomes/measures and follow-up period	Results
					9. Medications count<br><br>10. Social activities count<br><br>11. Symptom scale<br><br>12. SF-36 self-rated health<br><br>13. Nutrition checklist	9. No difference in change between IG and CG (0.3 vs 0, p=0.26)<br><br>10. Significant increase in IG vs reduction in CG (0.2 vs -0.3, p=0.04)<br><br>11. No significant change in IG vs CG (-0.5 vs 1.0, p=0.08)<br><br>12. No significant change in IG vs CG (0 vs 0.1, p=0.08)<br><br>13. No significant change in IG or CG (0.3 vs 0, p=0.12)
53	Gustafson et al (1994) (69)<br><br>USA	Test the impact of an interactive, computerised, personal health support system on adults with HIV<br><br>RCT	N=107 in intervention group, mean age 34.8 years<br>n=97 in control group, mean age 34.5 years	Intervention: Participants were given a PC based Comprehensive Health Enhancement Support System (CHESS) in their homes for 6 or 3 months. This enables access to health information, asking	1.Quality of life scores: Medical Outcomes Survey (MOS) at baseline, 2 and 5 months	1.At 2 months the intervention group reported significantly improved cognitive functioning (p=0.053), more active lives (p=0.013),

For peer review only

Study Number	Author & Year/<br><br>Country	Aim<br>Design<br>Theoretical model	Sample	Intervention(s)	Outcomes/measures and follow-up period	Results
				experts questions anonymously and reading personal accounts of others with similar problems.<br><br>Control: no details provided	 <br><br><br><br><br><br><br><br><br><br><br><br><br><br><br><br><br><br><br><br><br><br><br><br><br><br><br><br><br>2.Use of ambulatory care services in 2 months	decreased negative emotion (p=0.013) and better social support (p=0.074) than controls. Depression, physical function, energy and participation in healthcare did not show significant differences between groups.<br>At 5 months the intervention group reported more active life (p=0.034), improved social support (p=0.017) and more active participation in their healthcare (p=0.020). There was no difference between groups in cognitive function, negative emotions, depression, physical function, or energy.<br><br>2. No difference in frequency of visits to

For peer review only

Study Number	Author & Year/<br><br>Country	Aim<br>Design<br>Theoretical model	Sample	Intervention(s)	Outcomes/measures and follow-up period	Results
					before and after intervention implementation<br><br><br><br><br><br><br><br><br><br><br><br><br>3.Hospitalisation before, during and after intervention implementation	ambulatory care services between groups. Intervention group reported shorter visits than controls during the intervention (p=0.043) and were more likely to telephone providers both during (p=0.013) and after (p=0.094) the intervention.<br><br>3.Hospitalisations were lower for the intervention group than controls during the intervention (p=0.020) and shorter (p=0.009). These differences were not maintained after the intervention.

For peer review only

Study Number	Author & Year/ Country	Aim Design Theoretical model	Sample	Intervention(s)	Outcomes/measures and follow-up period	Results
54	Kinmonth et al (1998) (70) , UK	To assess the effect of additional training of practice nurses and general practitioners in patient centred care on lifestyle, psychological and physiological status of patients with type 2 diabetes. Pragmatic parallel group design, randomisation between practice teams to routine care. RCT.	N=41 practices n=21 intervention practices and 142 patients n=20 usual care practices and 108 patients. 250/360 patients (30-70 years) Mean age 41.54(SD=9.83) years.	1.5 days group training for the nurses and 0.5 days for doctors:<br><br>Reviewed evidence-based person-centred consulting and practised the skills they learnt with an experienced facilitator. Skills included active listening and negotiation of behavioural change. They produced materials including a booklet for patients, 'Diabetes in your hands' which encouraged patients to ask questions.<br><br>Comparison group nurses were offered similar support sessions focusing on use of guidelines and materials.	1. Quality of life: Audit of diabetes dependent quality of life (ADDQoL)<br><br>2. Communication and satisfaction with treatment<br><br>3.Wellbeing: The wellbeing questionnaire<br><br>4. Blood pressure<br><br>5. Body mass index (kg/m^2)	1. QoL mean in the intervention -1.09 and -1.23 in the control group (p=0.27).<br><br>2. Intervention showed better communication with doctors (odds 2.8 p<0.001), satisfaction with treatment (1.6 p=0.05)<br><br>3. Wellbeing: mean difference 2.8 (p=0.03)<br><br>4. Mean systolic BP 144.3 in the intervention and 142.8 in the control groups p=0.18 Diastolic BP 89.0 in the intervention and 87.2 in the control p=0.10<br><br>5. Mean BMI 31.3 in the intervention and

Study Number	Author & Year/<br><br>Country	Aim<br>Design<br>Theoretical model	Sample	Intervention(s)	Outcomes/measures and follow-up period	Results
					6. Haemoglobin A1c %	29.5 in the control p=0.03.<br><br>6. Mean HbA1c 7.07 in the IF and 7.17 in the control group (p=0.31).

For peer review only

Study Number	Author & Year/<br><br>Country	Aim<br>Design<br>Theoretical model	Sample	Intervention(s)	Outcomes/measures and follow-up period	Results
55	Landefeld (1995) (71), USA	To compare outcomes of people admitted to a unit especially designed to improve the functional outcomes of acutely ill older patients with standard care<br><br>RCT	n=651 people aged 70 or older admitted for general medical care at a teaching hospital<br>n=327 intervention group, mean age 80.2 (SD)<br>n=324 control group, mean age 80.1 (SD 6.6)	Intervention: Admission to a unit practising the Acute Care for Elders programme including a specially prepared environment, patient-centred care emphasizing independence, discharge planning aiming to discharge patients home and intensive review of medical care to minimise adverse effects of interventions and procedures.<br><br>Usual care: admission to acute care medical unit.<br>In both groups patients were assigned a primary nurse, two resident physicians and an attending physician. Staffing ratios and access to hospital support services including social work, physiotherapy, and nutrition.	Primary outcome:<br>1. Change from admission to discharge in the number of basic activities of daily living (ADLs) that the patient could perform independently<br><br>Secondary outcomes<br>2. Patients admitted from own home being discharged to a long-term care institution<br><br>3. Overall health status at discharge<br><br>4. Mean length of hospital stay<br><br>5. Mean total hospital charges	1. IG had greater improvement compared to CG (p=0.009)<br>The mean ADLs performed independently at discharge were 3.6 for IG and 3.3 for CG (p=0.05)<br><br>2. Fewer IG patients discharged to institution than CG (14% vs 22%, p=0.01)<br><br>3.Better health status in IG than CG (p<0.001)<br><br>4. Not significant<br><br>5. Not significant

1. Fortin M, Stewart M, Ngangue P, Almirall J, Bélanger M, Brown JB, et al. Scaling Up Patient-Centered Interdisciplinary Care for Multimorbidity: A Pragmatic Mixed-Methods Randomized Controlled Trial. Annals of Family Medicine. 2021;19(2):126-34.
2. de Batlle J, Massip M, Vargiu E, Nadal N, Fuentes A, Ortega Bravo M, et al. Implementing Mobile Health-Enabled Integrated Care for Complex Chronic Patients: Intervention Effectiveness and Cost-Effectiveness Study. JMIR Mhealth Uhealth. 2021;9(1):e22135.
3. Mielenz TJ, Tracy M, Jia H, Durbin LL, Allegrante JP, Arniella G, et al. Creation of the Person-Centered Wellness Home in Older Adults. Innovation in Aging. 2020;4(1).
4. Yu C, Choi D, Bruno BA, Thorpe KE, Straus SE, Cantarutti P, et al. Impact of MyDiabetesPlan, a Web-Based Patient Decision Aid on Decisional Conflict, Diabetes Distress, Quality of Life, and Chronic Illness Care in Patients With Diabetes: Cluster Randomized Controlled Trial. Journal of Medical Internet Research. 2020;22(9):N.PAG-N.PAG.
5. Bergsten U, Almehed K, Baigi A, Jacobsson LTH. A randomized study comparing regular care with a nurse-led clinic based on tight disease activity control and person-centred care in patients with rheumatoid arthritis with moderate/high disease activity: A 6-month evaluation. Musculoskeletal Care. 2019;17(3):215-25.
6. Berntsen GKR, Dalbakk M, Hurley JS, Bergmo T, Solbakken B, Spansvoll L, et al. Person-centred, integrated and pro-active care for multi-morbid elderly with advanced care needs: a propensity score-matched controlled trial. BMC health services research. 2019;19(1):682-.
7. Berendonk C, Kaspar R, Bär M, Hoben M. Improving Quality of Work life for Care Providers by Fostering the Emotional well-being of Persons with Dementia: A Cluster-randomized Trial of a Nursing Intervention in German long-term Care Settings. Dementia (London). 2019;18(4):1286-309.
8. Bökberg C, Behm L, Wallerstedt B, Ahlström G. Evaluation of person-centeredness in nursing homes after a palliative care intervention: pre- and post-test experimental design. BMC Palliative Care. 2019;18(1):44.
9. Britt HR, JaKa, M. M., Fernstrom, K. M., Bingham, P. E., Betzner, A. E., Taghon, J. R., Shippee, N. D., Shippee, T. P., Schellinger, S. E., & Anderson, E. W. . Quasi-Experimental Evaluation of LifeCourse on Utilization and Patient and Caregiver Quality of Life and Experience. . The American journal of hospice & palliative care 2019;36(5):408-16.
10. Hedman A, Eriksson G, von Koch L, Guidetti S. Five-year follow-up of a cluster-randomized controlled trial of a client-centred activities of daily living intervention for people with stroke. Clin Rehabil. 2019;33(2):262-76.
11. Bertilsson AS, Eriksson G, Ekstam L, Tham K, Andersson M, von Koch L, et al. A cluster randomized controlled trial of a client-centred, activities of daily living intervention for people with stroke: one year follow-up of caregivers. Clinical rehabilitation. 2016;30(8):765-75.
12. Guidetti S, Ranner M, Tham K, Andersson M, Ytterberg C, von Koch L. A "Client-Centred Activities of Daily Living" Intervention for Persons with Stroke: One-Year Follow-up of a Randomized Controlled Trial. Journal of Rehabilitation Medicine. 2015;47(7):605-11.
13. Bertilsson A-S, Ranner M, von Koch L, Eriksson G, Johansson U, Ytterberg C, et al. A client-centred ADL intervention: three-month follow-up of a randomized controlled trial. Scandinavian journal of occupational therapy. 2014;21(5):377-91.
14. Öhlén J, Sawatzky R, Pettersson M, Sarenmalm EK, Larsdotter C, Smith F, et al. Preparedness for colorectal cancer surgery and recovery through a person-centred

For peer review only

information and communication intervention - A quasi-experimental longitudinal design. PLoS One. 2019;14(12):e0225816.
15. Pirhonen L, Bolin K, Olofsson EH, Fors A, Ekman I, Swedberg K, et al. Person-Centred Care in Patients with Acute Coronary Syndrome: Cost-Effectiveness Analysis Alongside a Randomised Controlled Trial. PharmacoEconomics - Open. 2019;3(4):495-504.
16. Pirhonen L, Olofsson EH, Fors A, Ekman I, Bolin K. Effects of person-centred care on health outcomes-A randomized controlled trial in patients with acute coronary syndrome. Health Policy. 2017;121(2):169-79.
17. Fors A, Swedberg K, Ulin K, Wolf A, Ekman I. Effects of person-centred care after an event of acute coronary syndrome: Two-year follow-up of a randomised controlled trial. International Journal of Cardiology. 2017;249:42-7.
18. Fors A, Taft C, Ulin K, Ekman I. Person-centred care improves self-efficacy to control symptoms after acute coronary syndrome: A randomized controlled trial. European Journal of Cardiovascular Nursing. 2016;15(2):186-94.
19. Fors A, Gyllensten H, Swedberg K, Ekman I. Effectiveness of person-centred care after acute coronary syndrome in relation to educational level: Subgroup analysis of a two-armed randomised controlled trial. Int J Cardiol. 2016;221:957-62.
20. Fors A, Ekman I, Taft C, Björkelund C, Frid K, Larsson ME, et al. Person-centred care after acute coronary syndrome, from hospital to primary care - A randomised controlled trial. Int J Cardiol. 2015;187:693-9.
21. Wolf A, Fors A, Ulin K, Thorn J, Swedberg K, Ekman I. An eHealth Diary and Symptom-Tracking Tool Combined With Person-Centered Care for Improving Self-Efficacy After a Diagnosis of Acute Coronary Syndrome: A Substudy of a Randomized Controlled Trial. J Med Internet Res. 2016;18(2):e40.
22. Zakrisson AB, Arne M, Hasselgren M, Lisspers K, Ställberg B, Theander K. A complex intervention of self-management for patients with COPD or CHF in primary care improved performance and satisfaction with regard to own selected activities; A longitudinal follow-up. J Adv Nurs. 2019;75(1):175-86.
23. Arian M, Memarian R, Oghazian MB, Vakilian F, Badiee Z. The effect of a holistic care program on the reduction of iron over load in patients with beta-thalassemia major: A randomized clinical trial. Iranian Red Crescent Medical Journal. 2018;20 (4) (no pagination)(e60820).
24. Eggers C, Dano R, Schill J, Fink GR, Hellmich M, Timmermann L. Patient-centered integrated healthcare improves quality of life in Parkinson's disease patients: a randomized controlled trial. J Neurol. 2018;265(4):764-73.
25. Fors A, Blanck E, Ali L, Swedberg K, Ekman I. Person-centred telephone-support is effective in patients with chronic obstructive pulmonary disease and/or chronic heart failure-six-month follow-up of a randomized controlled trial. European Journal of Heart Failure. 2018;20 (Supplement 1):194.
26. Reed RL, Roeger L, Howard S, Oliver-Baxter JM, Battersby MW, Bond M, et al. A self-management support program for older Australians with multiple chronic conditions: A randomised controlled trial. Medical Journal of Australia. 2018;208(2):69-74.
27. Schafer I, Kaduszkiewicz H, Mellert C, Loffler C, Mortsiefer A, Ernst A, et al. Narrative medicine-based intervention in primary care to reduce polypharmacy: results from the cluster-randomised controlled trial MultiCare AGENDA. BMJ Open. 2018;8(1):e017653.
28. Thom DH, Willard-Grace R, Tsao S, Hessler D, Huang B, DeVore D, et al. Randomized Controlled Trial of Health Coaching for Vulnerable Patients with Chronic Obstructive Pulmonary Disease. Annals of the American Thoracic Society. 2018;15(10):1159-68.

For peer review only

29. Armstrong KA, Coyte PC, Brown M, Beber B, Semple JL. Effect of home monitoring via mobile app on the number of in-person visits following ambulatory surgery a randomized clinical trial. JAMA Surgery. 2017;152(7):622-7.
30. Feldthusen C, Dean E, Forsblad-d'Elia H, Mannerkorpi K. Effects of Person-Centered Physical Therapy on Fatigue-Related Variables in Persons With Rheumatoid Arthritis: A Randomized Controlled Trial. Arch Phys Med Rehabil. 2016;97(1):26-36.
31. Hansson E, Carlström E, Olsson LE, Nyman J, Koinberg I. Can a person-centred-care intervention improve health-related quality of life in patients with head and neck cancer? A randomized, controlled study. BMC Nurs. 2017;16:9.
32. Ko FWS, Cheung NK, Rainer TH, Lum C, Wong I, Hui DSC. Comprehensive care programme for patients with chronic obstructive pulmonary disease: A randomised controlled trial. Thorax. 2017;72(2):122-8.
33. Low LL, Tan SY, Ng MJM, Tay WY, Ng LB, Balasubramaniam K, et al. Applying the Integrated Practice Unit Concept to a Modified Virtual Ward Model of Care for Patients at Highest Risk of Readmission: A Randomized Controlled Trial. PloS one. 2017;12(1):e0168757-e.
34. Wichit N, Mnatzaganian G, Courtney M, Schulz P, Johnson M. Randomized controlled trial of a family-oriented self-management program to improve self-efficacy, glycemic control and quality of life among Thai individuals with Type 2 diabetes. Diabetes Research and Clinical Practice. 2017;123:37-48.
35. Larsson A, Palstam A, Löfgren M, Ernberg M, Bjersing J, Bileviciute-Ljungar I, et al. Resistance exercise improves muscle strength, health status and pain intensity in fibromyalgia--a randomized controlled trial. Arthritis Res Ther. 2015;17(1):161.
36. Ericsson A, Palstam A, Larsson A, Löfgren M, Bileviciute-Ljungar I, Bjersing J, et al. Resistance exercise improves physical fatigue in women with fibromyalgia: a randomized controlled trial. Arthritis Res Ther. 2016;18:176.
37. Hansson E, Ekman I, Swedberg K, Wolf A, Dudas K, Ehlers L, et al. Person-centred care for patients with chronic heart failure - a cost-utility analysis. Eur J Cardiovasc Nurs. 2016;15(4):276-84.
38. Ulin K, Olsson LE, Wolf A, Ekman I. Person-centred care - An approach that improves the discharge process. European Journal of Cardiovascular Nursing. 2016;15(3):e19-26.
39. Ekman I, Wolf A, Olsson LE, Taft C, Dudas K, Schaufelberger M, et al. Effects of person-centred care in patients with chronic heart failure: the PCC-HF study. European Heart Journal. 2012;33(9):1112-9.
40. Dudas K, Olsson LE, Wolf A, Swedberg K, Taft C, Schaufelberger M, et al. Uncertainty in illness among patients with chronic heart failure is less in person-centred care than in usual care. European Journal of Cardiovascular Nursing. 2013;12(6):521-8.
41. Jutterstrom L, Hornsten A, Sandstrom H, Stenlund H, Isaksson U. Nurse-led patient-centered self-management support improves HbA1c in patients with type 2 diabetes-A randomized study. Patient Education and Counseling. 2016;99(11):1821-9.
42. Olsson LE, Hansson E, Ekman I. Evaluation of person-centred care after hip replacement-a controlled before and after study on the effects of fear of movement and self-efficacy compared to standard care. BMC Nurs. 2016;15(1):53.
43. Olsson L-E, Karlsson J, Berg U, Kärrholm J, Hansson E. Person-centred care compared with standardized care for patients undergoing total hip arthroplasty—a quasi-experimental study. Journal of Orthopaedic Surgery and Research. 2014;9(1):95.
44. Or C, Tao D. A 3-Month Randomized Controlled Pilot Trial of a Patient-Centered, Computer-Based Self-Monitoring System for the Care of Type 2 Diabetes Mellitus and Hypertension. Journal of Medical Systems. 2016;40(4):81.

For peer review only

45. Sahlen K-G, Boman K, Brannstrom M. A cost-effectiveness study of person-centered integrated heart failure and palliative home care: Based on a randomized controlled trial. Palliative Medicine. 2016;30(3):296-302.
46. Brannstrom M, Boman K. Effects of person-centred and integrated chronic heart failure and palliative home care. PREFER: a randomized controlled study. European Journal of Heart Failure. 2014;16(10):1142-51.
47. Slok AH, Kotz D, van Breukelen G, Chavannes NH, Rutten-van Molken MP, Kerstjens HA, et al. Effectiveness of the Assessment of Burden of COPD (ABC) tool on health-related quality of life in patients with COPD: a cluster randomised controlled trial in primary and hospital care. BMJ Open. 2016;6(7):e011519.
48. Windrum P, Garcia-Goni M, Coad H. The Impact of Patient-Centered versus Didactic Education Programs in Chronic Patients by Severity: The Case of Type 2 Diabetes Mellitus. Value in Health. 2016;19(4):353-62.
49. Yu DSF. Effects of a Health and Social Collaborative Case Management Model on Health Outcomes of Family Caregivers of Frail Older Adults: Preliminary Data from a Pilot Randomized Controlled Trial. Journal of the American Geriatrics Society. 2016;64(10):2144-8.
50. Hernandez C, Alonso A, Garcia-Aymerich J, Serra I, Marti D, Rodriguez-Roisin R, et al. Effectiveness of community-based integrated care in frail COPD patients: A randomised controlled trial. npj Primary Care Respiratory Medicine. 2015;25 (no pagination)(15022).
51. Kikkenborg Berg S, Stoier L, Moons P, Zwisler AD, Winkel P, Ulrich Pedersen P. Emotions and health: findings from a randomized clinical trial on psychoeducational nursing to patients with implantable cardioverter defibrillator. The Journal of cardiovascular nursing. 2015;30(3):197-204.
52. Larsson I, Fridlund B, Arvidsson B, Teleman A, Svedberg P, Bergman S. A nurse-led rheumatology clinic versus rheumatologist-led clinic in monitoring of patients with chronic inflammatory arthritis undergoing biological therapy: A cost comparison study in a randomised controlled trial. BMC Musculoskeletal Disorders. 2015;16 (1) (no pagination)(817).
53. Larsson I, Fridlund B, Arvidsson B, Teleman A, Bergman S. Treatment outcomes from a nurse-led rheumatology clinic in monitoring of anti-TNF therapy-a randomised controlled trial. Arthritis and Rheumatism. 2012;10):S667.
54. Lowther K, Selman L, Simms V, Gikaara N, Ahmed A, Ali Z, et al. Nurse-led palliative care for HIV-positive patients taking antiretroviral therapy in Kenya: a randomised controlled trial. Lancet HIV. 2015;2(8):e328-34.
55. Kelechi TJ, Mueller M, Spencer C, Rinard B, Loftis G. The effect of a nurse-directed intervention to reduce pain and improve behavioral and physical outcomes in patients with critically colonized/infected chronic leg ulcers. Journal of Wound, Ostomy, & Continence Nursing. 2014;41(2):111-21.
56. Young JM, Butow PN, Walsh J, Durcinoska I, Dobbins TA, Rodwell L, et al. Multicenter randomized trial of centralized nurse-led telephone-based care coordination to improve outcomes after surgical resection for colorectal cancer: the CONNECT intervention. Journal of Clinical Oncology. 2013;31(28):3585-91.
57. Chochinov HM, Kristjanson LJ, Breitbart W, McClement S, Hack TF, Hassard T, et al. Effect of dignity therapy on distress and end-of-life experience in terminally ill patients: a randomised controlled trial. Lancet Oncology. 2011;12(8):753-62.
58. Goelz T, Wuensch A, Stubenrauch S, Ihorst G, de Figueiredo M, Bertz H, et al. Specific training program improves oncologists' palliative care communication skills in a randomized controlled trial. Journal of Clinical Oncology. 2011;29(25):3402-7.

For peer review only

59. Murphy SL, Lyden AK, Smith DM, Dong Q, Koliba JF. Effects of a tailored activity pacing intervention on pain and fatigue for adults with osteoarthritis. American Journal of Occupational Therapy. 2010;64(6):869-76.
60. Wolff JL, Giovannetti ER, Boyd CM, Reider L, Palmer S, Scharfstein D, et al. Effects of guided care on family caregivers. The Gerontologist. 2010;50(4):459-70.
61. Dobscha SK, Corson K, Perrin NA, Hanson GC, Leibowitz RQ, Doak MN, et al. Collaborative care for chronic pain in primary care: a cluster randomized trial. JAMA. 2009;301(12):1242-52.
62. Machado LA, Azevedo DC, Capanema MB, Neto TN, Cerceau DM. Client-Centered Therapy vs Exercise Therapy for Chronic Low Back Pain: A Pilot Randomized Controlled Trial in Brazil. Pain Medicine. 2007;8(3):251-8.
63. Glasgow RE, Nutting PA, King DK, Nelson CC, Cutter G, Gaglio B, et al. Randomized effectiveness trial of a computer-assisted intervention to improve diabetes care. Diabetes Care. 2005;28(1):33-9.
64. Mills PD, Harvey PW. Beyond community-based diabetes management and the COAG coordinated care trial. Australian Journal of Rural Health. 2003;11(3):131-7.
65. Kennedy A, Nelson E, Reeves D, Richardson G, Roberts C, Robinson A, et al. A randomised controlled trial to assess the impact of a package comprising a patient-orientated, evidence-based self-help guidebook and patient-centred consultations on disease management and satisfaction in inflammatory bowel disease. Health Technology Assessment. 2003;7(28).
66. Martin IR, McNamara D, Sutherland FR, Tilyard MW, Taylor DR. Care plans for acutely deteriorating COPD: a randomized controlled trial. Chronic respiratory disease. 2004;1(4):191-5.
67. Alamo MM, Moral RR, Perula de Torres LA. Evaluation of a patient-centred approach in generalized musculoskeletal chronic pain/fibromyalgia patients in primary care. Patient Education & Counseling. 2002;48(1):23-31.
68. Sommers LS, Marton KI, Barbaccia JC, Randolph J. Physician, nurse, and social worker collaboration in primary care for chronically ill seniors. Archives of Internal Medicine. 2000;160(12):1825-33.
69. Gustafson DH, Hawkins RP, Boberg EW, Bricker E, Pingree S, Chan CL. The use and impact of a computer-based support system for people living with AIDS and HIV infection. Proc Annu Symp Comput Appl Med Care. 1994:604-8.
70. Kinmonth AL, Woodcock A, Griffin S, Spiegal N, Campbell MJ. Randomised controlled trial of patient centred care of diabetes in general practice: Impact on current wellbeing and future disease risk. British Medical Journal. 1998;317(7167):1202-8.
71. Landefeld CS, Palmer RM, Kresevic DM, Fortinsky RH, Kowal J. A randomized trial of care in a hospital medical unit especially designed to improve the functional outcomes of acutely ill older patients. New England Journal of Medicine. 1995;332(20):1338-44.

For peer review only

For peer review only

Supplementary file 3: Risk of Bias in the studies (Joanna Briggs Institute Critical Appraisal checklist)

Yes: means good and no risk of bias, No: means there was risk of bias, ITTA: Intention to treat analysis, IG: Intervention group, CG: control group

	Author	Random allocation	Allocation concealment	Baseline similarity/Comparable at entry	Blinding of participants	Blinding of those delivering treatment	Blinding of outcome assessors	Identical except intervention	Relatively complete follow-up achieved	Participants analysed in the groups originally randomised	Outcomes measures same between group	Outcomes assessed in reliable way	Statistical analysis appropriate	Total YES scores
1	Fortin et al 2021	Yes	Yes	Yes	No	No	Unclear	Yes	Yes	Yes	Yes	Yes	Yes	9/12
2	Yu et al 2020	Yes	Yes	No	No	No	Yes	Yes	No, 71%	Yes	Yes	Yes	Yes	8/12
3	de Batlle, 2020	Unclear	Unclear	No	No	Unclear	NA assessed through a web-based survey/mail	Yes	Yes, 87%	Yes, Modified ITTA	Yes	Yes	Yes	6/12
4	Mielenz et al 2020	Yes	Unclear	Yes	No	No	Yes	Yes	Yes, 97%	Yes, Modified	Yes	Yes	Yes	9/12
5	Bergsten et al 2019	Yes	Unclear	No	No	No	Yes	Yes	Yes, 83%	Yes	Yes	Yes	Yes	8/12
6	Berntsen et al (2019)	No	N/A	No	No	No	Unclear	Yes	Unclear	Yes	Yes	Yes	Yes	5/12
7	Berondonk (2019)	Yes	Yes	Yes	No	No	Unclear	Yes	Yes, 82%	Yes, Modified ITTA	Yes	Yes	Yes	9/12
8	Bokberg et al (2019)	No	N/A	No	No	No	Yes	Yes	No	Yes, Modified ITTA	Yes	Yes	Yes	6/12
9	Britt et al (2019)	No, patients recruited at separate hospitals. Those declining participation in IG were offered inclusion in CG	No	CG were younger, more likely to be married and more likely to live at home. CG were more likely to have a cardiovascular, and less likely to have dementia as a primary diagnosis.	No	No	No	Yes	No, 51% completed 12 months	Unclear	Yes	Yes	Yes	4/12

For peer review only

	Author	Random allocation	Allocation concealment	Baseline similarity/Comparable at entry	Blinding of participants	Blinding of those delivering treatment	Blinding of outcome assessors	Identical except intervention	Relatively complete follow-up achieved	Participants analysed in the groups originally randomised	Outcomes measures same between group	Outcomes assessed in reliable way	Statistical analysis appropriate	Total YES scores
10	Hedman, et al 2019 Bertilsson et al (2016), Guidenti et al (2015) and Bertilsson et al (2014) One study reporting three papers	Unclear	Unclear	Unclear	No	No	Unclear	Yes	Yes, 81%	Yes, Modified ITTA	Yes	Yes	Yes	6/12
11	Ohlen et al (2019)	No	N/A	Yes	No	No	N/A	Yes	Yes, 82%	Yes, ITTA	Yes	Yes	Yes	7/12
12	Pirhonen et al 2019 Pirhonen et al 2016, Fors et al (2017); Fors et al (2016a)a Fors et al (2016b) Wolf et al 2016 and Fors et al 2015 Seven papers one study	Yes	Yes	Yes	No	No	Not clear	Yes	Yes, 91%	Yes	Yes	Yes	Yes	9/12
13	Zakrisson et al (2019)	Yes	Yes	No	No	No	Yes	Yes	No	Yes	Yes	Yes	Yes	8/12
14	Arian et al (2018)	Yes	Unclear	Yes	No	No	Yes	Yes	No	Yes	Yes	Yes	Yes	8/12

For peer review only

	Author	Random allocation	Allocation concealment	Baseline similarity/Comparable at entry	Blinding of participants	Blinding of those delivering treatment	Blinding of outcome assessors	Identical except intervention	Relatively complete follow-up achieved	Participants analysed in the groups originally randomised	Outcomes measures same between group	Outcomes assessed in reliable way	Statistical analysis appropriate	Total YES scores
15	Eggers, 2018	Yes	Yes	Yes	No	No	Unclear	Yes	Yes, 86%	Yes, Modified ITTA	Yes	Yes	Yes	9/12
16	Fors et al (2018)	Yes	Not clear	Yes	No	No	N/A: patients self-completed and sent by post	Yes	Yes, 91%	Yes	Yes	Yes	Yes	8/12
17	Reed et al 2018)	Yes	Yes	Yes	Yes	No	Yes	Yes	Yes, 91%	Yes	Yes	Yes	Yes	11/12
18	Schäfer et al (2018)	Yes	Yes	No difference in characteristics of GPs and practices between groups. More female patients in the control group.	No	No	No	Yes	Yes, 93% completed	Yes, Modified ITTA	No	Yes	Yes	7/12
19	Thom et al 2018	Yes	Yes	Yes	No	No	Unclear	Yes	Yes, 82%	Yes	Yes	Yes	Yes	9/12
20	Armstrong et al (2017)	Unclear	Yes	Yes	No	No	Unclear	Yes	Yes, 93%	Yes	Yes	Yes	Yes	8/12
21	Feldthusen et al 2017	Yes	Yes	Yes	No	Yes	Yes	Yes	Yes, 96%	Yes	Yes	Yes	Yes	11/12
22	Hansson et al 2017<br><br>Gyllensten et al 2019	Yes	Yes	No	No	No	No	Yes	Yes, 92%	Yes	Yes	Yes	Yes	8/12
23	Ko et al (2017)	Yes	Yes	IG had higher FEV_1% of predicted	No	No	Yes	Yes	No, 79%	Yes	Yes	Yes	Yes	8/12
24	Low et al (2017)	Yes	Yes	Yes	No	No	Yes	Yes	Yes, 87%	Yes	Yes	Yes	Yes	10/12
25	Wichit et al (2017)	Yes	Yes	IG were older	Yes	No	Yes	Yes	Yes, 96%	Yes	Yes	Yes	Yes	10/12
26	Ericsson et al 2016	Yes	Yes	Yes	No	No	Yes	Yes	No, 70%	Yes, ITTA	Yes	Yes	Yes	9/12

For peer review only

	Author	Random allocation	Allocation concealment	Baseline similarity/Comparable at entry	Blinding of participants	Blinding of those delivering treatment	Blinding of outcome assessors	Identical except intervention	Relatively complete follow-up achieved	Participants analysed in the groups originally randomised	Outcomes measures same between group	Outcomes assessed in reliable way	Statistical analysis appropriate	Total YES scores
	Larsson et al 2015<br><br>Two papers one study													
27	Hansson et al 2016; Ulin et al 2016; Ekman et al (2012) and Dudas et al 2012 Four papers one study	No	N/A	No	No	No	Unclear	Yes	Yes, 80%	Yes, ITTA	Yes	Yes	Yes	6/12
28	Jutterström et al (2016)	Yes	Yes	No, Greater numbers in the Group intervention group. Group intervention had greater HbA1c than External control group, and lower total cholesterol than internal control. External control group were more likely to have diet and/or insulin treatment	No	No	No	Yes	Yes, 88%	Yes, Modified ITTA	Yes	Yes	Yes	8/12
29	Olsson et al 2016 and	No	N/A	No	No	No	Unclear	Yes	Yes, 99%	Yes	Yes	Yes	Yes	6/12

	Author	Random allocation	Allocation concealment	Baseline similarity/Comparable at entry	Blinding of participants	Blinding of those delivering treatment	Blinding of outcome assessors	Identical except intervention	Relatively complete follow-up achieved	Participants analysed in the groups originally randomised	Outcomes measures same between group	Outcomes assessed in reliable way	Statistical analysis appropriate	Total YES scores
	Olsson et al 2014<br><br>Two papers one study													
30	Or and Tao (2016)	Yes	Yes	Yes	No	No	No	Yes	Yes, 87%	Yes, Modified ITTA	No, IG self monitoring data captured on a tablet, CG from log book records.	Yes	Yes	8/12
31	Sahlen et al (2016); Brännstrom & Boman (2014) One study two papers	Unclear	Unclear	IG older	No	No	Unclear	Yes	Yes, 83%	Yes, Modified ITTA	Yes	Yes	Yes	6/12
32	Slok et al (2016)	Yes	Yes	IG more likely to be a current smoker	No	No	No	Yes	Yes, 82%	Yes, Modified ITTA	Yes	Yes	Yes	8/12
33	Windrum et al (2016)	Unclear	Unclear	Yes	No	Yes	Unclear	Yes	Unclear	Unclear	Unclear	Unclear	Yes	4/12
34	Yu (2016)	Yes	Unclear	Yes	No	No	Yes	Yes	Yes	Yes, Modified ITTA	Yes	Yes	Yes	9/12
35	Hernández et al (2015)	Yes	Yes	CG more likely to have had influenza and pneumococccal vaccines	No	No	Yes	Yes	No, 71%	Yes, Modified ITTA	Yes	Yes	Yes	8/12
36	Kikkenborg et al (2015)	Unclear	Unclear	Yes	No	No	Yes	Yes	Yes, 84%	Yes, Modified ITTA	Yes	Yes	Yes	8/12

	Author	Random allocation	Allocation concealment	Baseline similarity/Comparable at entry	Blinding of participants	Blinding of those delivering treatment	Blinding of outcome assessors	Identical except intervention	Relatively complete follow-up achieved	Participants analysed in the groups originally randomised	Outcomes measures same between group	Outcomes assessed in reliable way	Statistical analysis appropriate	Total YES scores
37	Larsson et al (2015) and 2013 One study two papers	Yes	Yes	Yes	No	No	No	Yes	Yes	Yes	Yes	Yes	Yes	9/12
38	Lowther et al (2015)	Yes	Yes	CG had been diagnosed with HIV for longer and been taking ART for longer than IG.	No	No	No	Yes	Yes, 95%	Yes, Modified ITTA	Yes	Yes	Yes	8/12
39	Kelechi et al (2014)	Yes	Not clear	Greater motivation in IG Patient baseline demographic characteristics not reported by group	No	No	Not clear	Yes	Yes, 88%	Yes, Modified ITTA	Yes	Not stated	Yes	6/12
40	Young et al (2013)	Yes	Yes	IG more likely to have private health insurance, were admitted to a private hospital and had a stoma created.	No	No	Unclear	Yes	Yes, 88%	Yes, Modified ITTA	Yes	Yes	Yes	8/12
41	Chochinov et al (2011)	Yes	Yes	Yes	No	No	Yes	Yes	No, 74%	Yes, Modified ITTA	Yes	Unclear	Yes	8/12
42	Goelz et al (2011)	Yes	Yes	No	No	Unclear	Unclear	Yes	Yes	Yes	Yes	Yes	Yes	8/12
43	Murphy et al (2010)	Yes	Unclear	Yes	No	No	Yes	Yes	No, 74%	Yes, Modified ITTA	Yes	Unclear	Yes	7/12
44	Wolff et al (2010)	Unclear, Cluster	Unclear	CG more likely to be female	Unclear	No	Yes	Yes	No, 69% of patients and	Yes, Modified ITTA	Yes	Yes	Yes	6/12

For peer review only

	Author	Random allocation	Allocation concealment	Baseline similarity/Comparable at entry	Blinding of participants	Blinding of those delivering treatment	Blinding of outcome assessors	Identical except intervention	Relatively complete follow-up achieved	Participants analysed in the groups originally randomised	Outcomes measures same between group	Outcomes assessed in reliable way	Statistical analysis appropriate	Total YES scores
		randomization		and less educated					64% of caregivers					
45	Dobscha et al (2009)	Unclear	Yes	Yes	Yes	Yes	Yes	Yes	Yes, 90%	Yes, ITTA	Yes	Yes	Yes	11/12
46	Machado et al (2007)	Yes	Yes	IG had longer duration of symptoms, more likely to be female and more likely to not be working due to lower back pain	No	No	Yes	Yes	Yes, 81%	Yes, Modified ITTA	Yes	Yes	Yes	9/12
47	Glasgow et al 2005	Yes	Yes	Yes	No	No	unclear	Yes	Yes, 83%	Yes	Yes	Yes	Yes	9/12
48	Mills and Harvey (2003)	Unclear	Unclear	No	No	No	Unclear	Yes	No	Yes, modified ITTA	Yes	Yes	Yes	5/12
49	Kennedy et al (2004)	Unclear	Unclear	IG more likely to be off work with long term sickness	No	No	Unclear	Differences in discharge policies between centres.	Yes, 87%	Yes, ITTA	Yes	Unclear	Yes	4/12
50	Martin et al, (2004),	Unclear	Unclear	CG were more likely to be male and have greater cigarette consumption.	No	No	Unclear	Yes	Yes, 83%	Yes, Modified ITTA	Yes	Yes	Yes	6/12
51	Alamo et al (2002)	Unclear	Unclear	IG more tender points and more likely to describe pain as never/hardly ever a problem.	No	No	Yes	Yes	No, 74%	Yes	Yes	Yes	Yes	6/12
52	Sommers et al (2000)	Yes	Unclear	No	No	No	Unclear	Yes	No	Yes, Modified ITTA	Yes	Yes	Yes	6/12

For peer review only

	Author	Random allocation	Allocation concealment	Baseline similarity/Comparable at entry	Blinding of participants	Blinding of those delivering treatment	Blinding of outcome assessors	Identical except intervention	Relatively complete follow-up achieved	Participants analysed in the groups originally randomised	Outcomes measures same between group	Outcomes assessed in reliable way	Statistical analysis appropriate	Total YES scores
53	Gustafson et al (1994)	Yes	Yes	Yes	No	No	Unclear	Yes	Yes, 84%	Yes, Modified ITTA	Yes	Unclear	Yes	8/12
54	Kinmonth et al (1998)	Yes	Yes	Yes	Unclear	No	Unclear	Yes	No, 69%	Yes, Modified ITTA	Yes	Yes	Yes	8/12
55	Landefeld et al 1995	Yes	Unclear	Yes	No	No	No	Yes	Yes	Yes, Modified ITTA	Yes	Yes	Yes	8/12

For peer review only

PRISMA 2009 Checklist

Section/topic	#	Checklist item	Reported on page #
**TITLE**			
Title	1	Identify the report as a systematic review, meta-analysis, or both.	1
**ABSTRACT**			
Structured summary	2	Provide a structured summary including, as applicable: background; objectives; data sources; study eligibility criteria, participants, and interventions; study appraisal and synthesis methods; results; limitations; conclusions and implications of key findings; systematic review registration number.	2
**INTRODUCTION**			
Rationale	3	Describe the rationale for the review in the context of what is already known.	6
Objectives	4	Provide an explicit statement of questions being addressed with reference to participants, interventions, comparisons, outcomes, and study design (PICOS).	9
**METHODS**			
Protocol and registration	5	Indicate if a review protocol exists, if and where it can be accessed (e.g., Web address), and, if available, provide registration information including registration number.	6
Eligibility criteria	6	Specify study characteristics (e.g., PICOS, length of follow-up) and report characteristics (e.g., years considered, language, publication status) used as criteria for eligibility, giving rationale.	8-9
Information sources	7	Describe all information sources (e.g., databases with dates of coverage, contact with study authors to identify additional studies) in the search and date last searched.	7
Search	8	Present full electronic search strategy for at least one database, including any limits used, such that it could be repeated.	Supplementary file 1
Study selection	9	State the process for selecting studies (i.e., screening, eligibility, included in systematic review, and, if applicable, included in the meta-analysis).	9-10
Data collection process	10	Describe method of data extraction from reports (e.g., piloted forms, independently, in duplicate) and any processes for obtaining and confirming data from investigators.	10
Data items	11	List and define all variables for which data were sought (e.g., PICOS, funding sources) and any assumptions and simplifications made.	10
Risk of bias in individual studies	12	Describe methods used for assessing risk of bias of individual studies (including specification of whether this was done at the study or outcome level), and how this information is to be used in any data synthesis.	10
Summary measures	13	State the principal summary measures (e.g., risk ratio, difference in means).	N/A
Synthesis of results	14	Describe the methods of handling data and combining results of studies, if done, including measures of consistency (e.g., I^2) for each meta-analysis.	10

For peer review only - http://bmjopen.bmj.com/site/about/guidelines.xhtml

PRISMA 2009 Checklist

Section/topic	#	Checklist item	Reported on page #
Risk of bias across studies	15	Specify any assessment of risk of bias that may affect the cumulative evidence (e.g., publication bias, selective reporting within studies).	N/A
Additional analyses	16	Describe methods of additional analyses (e.g., sensitivity or subgroup analyses, meta-regression), if done, indicating which were pre-specified.	N/A
**RESULTS**			
Study selection	17	Give numbers of studies screened, assessed for eligibility, and included in the review, with reasons for exclusions at each stage, ideally with a flow diagram.	12-13 Fig. 3
Study characteristics	18	For each study, present characteristics for which data were extracted (e.g., study size, PICOS, follow-up period) and provide the citations.	12-13
Risk of bias within studies	19	Present data on risk of bias of each study and, if available, any outcome level assessment (see item 12).	N/A
Results of individual studies	20	For all outcomes considered (benefits or harms), present, for each study: (a) simple summary data for each intervention group (b) effect estimates and confidence intervals, ideally with a forest plot.	Supplementary file 2
Synthesis of results	21	Present results of each meta-analysis done, including confidence intervals and measures of consistency.	18-26
Risk of bias across studies	22	Present results of any assessment of risk of bias across studies (see Item 15).	Supplementary file 3
Additional analysis	23	Give results of additional analyses, if done (e.g., sensitivity or subgroup analyses, meta-regression [see Item 16]).	N/A
**DISCUSSION**			
Summary of evidence	24	Summarize the main findings including the strength of evidence for each main outcome; consider their relevance to key groups (e.g., healthcare providers, users, and policy makers).	26
Limitations	25	Discuss limitations at study and outcome level (e.g., risk of bias), and at review-level (e.g., incomplete retrieval of identified research, reporting bias).	31-32
Conclusions	26	Provide a general interpretation of the results in the context of other evidence, and implications for future research.	32-33
**FUNDING**			
Funding	27	Describe sources of funding for the systematic review and other support (e.g., supply of data); role of funders for the systematic review.	34

From: Moher D, Liberati A, Tetzlaff J, Altman DG, The PRISMA Group (2009). Preferred Reporting Items for Systematic Reviews and Meta-Analyses: The PRISMA Statement. PLoS Med 6(7): e1000097. doi:10.1371/journal.pmed1000097

For peer review only - http://bmjopen.bmj.com/site/about/guidelines.xhtml

PRISMA 2009 Checklist

For more information, visit: www.prisma-statement.org.

Page 2 of 2

For peer review only
